# Associations between self-reported interoception and resting-state EEG markers in panic disorder: heartbeat-evoked potentials and spectral power

**DOI:** 10.1186/s40359-026-04736-7

**Published:** 2026-05-12

**Authors:** Ziheng Gao, Zhiwan Xiong, Meng Li, Yi Chang, Tommi Kärkkäinen, Fengyu Cong, Xiaoshuang Wang

**Affiliations:** 1 Central Hospital of Dalian University of Technology, Dalian, China; 2 Faculty of Information Technology, University of Jyvaskylä, Jyväskylä, Finland; 3 School of Biomedical Engineering, Faculty of Medicine, Dalian University of Technology, Dalian, China; 4 Department of Neurology and Psychiatry, First Affiliated Hospital, Dalian Medical University, Dalian, China; 5 Center for Mental Health Education, Nanchang University College of Science and Technology, Jiujiang, China; 6 Key Laboratory of Social Computing and Cognitive Intelligence, Ministry of Education, Dalian University of Technology, Dalian, China

**Keywords:** Panic disorder, Interoception, Resting-state EEG, Heartbeat-evoked potential, Spectral power

## Abstract

**Background:**

Panic disorder (PD) is associated with altered interoceptive experience, but it remains unclear how self-reported interoception relates to neurophysiological indices of bodily-signal processing. This study examined whether associations between self-reported interoception and resting-state electroencephalography (EEG) markers differ between participants with PD and healthy controls (HC).

**Methods:**

This analysis included 19 PD participants and 21 HCs from a previously published resting-state EEG dataset. Self-reported interoception was assessed using the Body Perception Questionnaire-Very Short Form (BPQ-VSF) and the Multidimensional Assessment of Interoceptive Awareness (MAIA). Resting-state EEG signals were recorded during eyes-closed and eyes-open conditions. Heartbeat-evoked potentials (HEP) and relative band power (RBP) were extracted to index cardiac-related cortical processing and frequency-specific resting-state activity. Associations between self-report measures and EEG indices were examined using Pearson correlations with 95% confidence intervals, Bayes factors, and false-discovery-rate (FDR) correction across scalp regions within predefined analysis families. Age-adjusted sensitivity analyses and group × self-report interaction models were used to assess robustness and formally test group differences in association patterns.

**Results:**

Compared with HCs, PD participants reported higher body-focused attention and lower scores on several adaptive interoceptive dimensions, including Not-Distracting, Not-Worrying, and Trusting. In the 235—301 ms HEP window, age-adjusted interaction models showed that BPQ-VSF HEP coupling differed between groups, especially under eyes-open conditions. Within-group analyses showed an exploratory opposite-direction BPQ-VSF HEP pattern, with positive associations in HC and negative associations in PD. The clearest FDR-corrected HEP association within panic disorder involved Not-Worrying under eyes closed. RBP analyses showed PD-concentrated $\theta$ (theta)- and $\beta$ (beta)-band association profiles. Trusting was positively associated with $\theta$ power in PD, and BPQ-VSF showed positive $\beta$-band associations under eyes closed that remained robust after age adjustment. Not-Distracting showed negative $\theta$*-*band associations in the primary correlation analyses, but these were attenuated after controlling for age.

**Conclusions:**

These findings provide preliminary FDR-controlled and age-informed evidence that PD involves altered, condition- and frequency-dependent mappings between subjective bodily experience and resting-state EEG markers. The clearest formal group-difference evidence was observed for BPQ-VSF HEP coupling, whereas PD-specific associations were most robust for Not-Worrying-HEP, Trusting-$\theta$ power, and BPQ-VSF $\beta$ power. Given the modest sample size and exploratory correlational design, replication in larger samples is needed.

**Supplementary Information:**

The online version contains supplementary material available at 10.1186/s40359-026-04736-7.

## Background

Panic disorder (PD) is characterized by recurrent unexpected panic attacks, anticipatory anxiety, and avoidance. A central clinical feature of PD is the tendency to rapidly detect, amplify, and catastrophically interpret bodily sensations such as palpitations, dyspnea, dizziness, and chest tightness. Interoception-the process by which the nervous system senses, integrates, and interprets signals originating from within the body-has therefore been considered highly relevant to the onset and maintenance of panic symptoms [1, 2].

However, interoception is not a unitary construct. Contemporary frameworks distinguish objective performance, self-reported interoceptive sensibility/attention, and higher-order awareness or appraisal, and emphasize that these dimensions can dissociate within the same individual [3, 4]. This distinction is especially important in anxiety research. Meta-analytic evidence indicates that anxiety is not reliably associated with greater cardiac interoceptive accuracy, whereas self-reported interoception is more consistently related to increased frequency, sensitivity, negative evaluation, and negative attention to bodily signals [5, 6]. Consistent with this view, people with panic attacks may show heightened worries and beliefs about bodily sensations without superior objective heartbeat detection performance [7]. Accordingly, the Body Perception Questionnaire-Very Short Form (BPQ-VSF) is useful for indexing attention to bodily sensations and symptom-focused body awareness, whereas the Multidimensional Assessment of Interoceptive Awareness (MAIA) is better suited to differentiating adaptive and maladaptive styles of bodily appraisal and regulation [8–10].

At the neural level, the heartbeat-evoked potential (HEP) is widely used as an electrophysiological index of cortical processing of cardiac signals. A recent meta-analysis concluded that HEP amplitude shows moderate associations with behavioral interoception and is sensitive to manipulations of attention, arousal, and clinical status, although methodological heterogeneity remains substantial across studies [11]. Resting-state work further suggests that HEP is modulated by eyes-closed (EC) and eyes-open (EO) states: in healthy individuals, HEP amplitudes are typically larger under eyes closed, whereas people with generalized anxiety disorder show altered state-dependent modulation, particularly under eyes open [12, 13]. Nevertheless, evidence regarding HEP characteristics in PD remains limited, and little is known about whether HEP covaries with multidimensional self-report indices of interoception in this population.

A complementary perspective comes from frequency-domain analysis of resting-state electroencephalography (EEG). In the present study, frequency-domain features were derived by estimating power spectral density (PSD) and extracting relative band power (RBP) across conventional frequency bands. RBP may capture oscillatory processes related to arousal, attention, and control. At the same time, large-scale reviews caution against assigning overly fixed psychological meanings to individual frequency bands or assuming disorder-specific signatures from isolated spectral findings, given the substantial overlap and heterogeneity across psychiatric conditions [14]. This caution is particularly relevant in panic research, where frequency-domain EEG evidence remains sparse. In our previous report based on the same resting-state EEG dataset, people with PD showed impaired HEP modulation and elevated beta-band power relative to healthy controls (HC), suggesting altered interoceptive regulation and cortical hyperarousal at the group level [15]. What remains unknown is whether these electrophysiological abnormalities are systematically related to self-reported interoceptive attention and beliefs.

The present study addressed this question by combining BPQ-VSF and MAIA measures with HEP and RBP indices obtained during resting-state EO and EC conditions. Rather than focusing only on group differences in neural activity, we examined how self-reported interoceptive experience was associated with electrophysiological markers within each group and formally tested whether key self-report-EEG association patterns differed between PD and HC. We hypothesized that the correspondence between subjective interoception and EEG indices would differ between PD and HC, and that these associations would be condition-dependent and frequency-specific. By testing these cross-domain association patterns, the study aimed to clarify whether PD is characterized not simply by stronger or weaker interoceptive processing, but by altered mappings between subjective bodily experience and electrophysiological markers.

## Methods

### Study design and participants

The parent study primarily reported between-group differences in HEP modulation and RBP between people with PD and HC, whereas the current secondary analysis focused on associations between self-reported interoceptive measures and electrophysiological indices, as well as formal group differences in key self-report–EEG association patterns [15].

A total of 21 people with PD and 22 HC were recruited from the First Affiliated Hospital of Dalian Medical University. Two participants with PD and one HC were excluded because of poor data quality, yielding a final sample of 19 people with PD and 21 HC. PD diagnoses were confirmed by qualified clinical assessors according to the Diagnostic and Statistical Manual of Mental Disorders, Fifth Edition (DSM-5) criteria [16]. Inclusion criteria for the PD group were age between 18 and 65 years, meeting DSM-5 diagnostic criteria for PD, and no pharmacological or psychological intervention within two weeks before the experiment. HC were matched to the PD group on age and sex and did not meet diagnostic criteria for panic disorder.

Participants in both groups were screened for recent medication use and treatment exposure. No participant reported current medication use or recent pharmacological or psychological intervention likely to affect EEG signals or interoceptive processing within two weeks before EEG recording. Exclusion criteria included pregnancy, other psychiatric disorders, malignant tumors, cerebral infarction, leukemia, or other major medical illnesses. The study was approved by the Ethics Committee of Dalian Medical University and was conducted in accordance with the Declaration of Helsinki. All participants provided written informed consent before participation.

### Clinical and interoceptive measures

All participants completed the Hamilton Anxiety Scale (HAMA) [17], the 17-item Hamilton Depression Scale (HAMD-17) [18], the BPQ-VSF [8], and the MAIA [10]. People with PD additionally completed the Panic Disorder Severity Scale (PDSS) [19] and the Panic-Associated Symptom Scale (PASS) [20].

The BPQ-VSF was used as an index of self-reported body awareness/interoceptive attention [8]. The MAIA was used to assess attentional, appraisal-related, and regulatory aspects of bodily-signal experience [10]. In the present manuscript, the MAIA version comprised 32 items and eight subscales: Noticing, Not-Distracting, Not-Worrying, Attention Regulation, Emotional Awareness, Self-Regulation, Body Listening, and Trusting [10].

### EEG acquisition procedure

Participants underwent resting-state EEG recording under 3-min EO and 3-min EC conditions in a sound-attenuated room. EEG data were acquired using a Neuroscan SynAmps2 amplifier with a 64-channel Ag/AgCl cap arranged according to the international 10—20 system [21]. Continuous EEG signals were recorded at a sampling rate of 500 Hz with an online bandwidth of 0.1—100 Hz. The nose tip served as the reference electrode. Horizontal electrooculogram electrodes were placed 1 cm lateral to the outer canthi of both eyes, and vertical electrooculogram electrodes were placed 1 cm above and below the left eye. Electrode impedances were maintained below 5 kΩ throughout the recording. Electrocardiographic (ECG) signals were recorded simultaneously for subsequent R-wave detection.

### EEG preprocessing and HEP extraction

HEP preprocessing was performed in EEGLAB [22] running in MATLAB 2023a. The preprocessing pipeline included a 47—53 Hz finite impulse response (FIR) notch filter to remove line noise, a 0.5 Hz FIR high-pass filter to reduce DC drift, and a 30 Hz FIR low-pass filter to attenuate electromyographic noise. Independent component analysis (ICA) was used to remove ocular artifacts [23]. R-waves were detected from the simultaneously recorded ECG using the Pan-Tompkins algorithm [24].

**Fig. 1:**
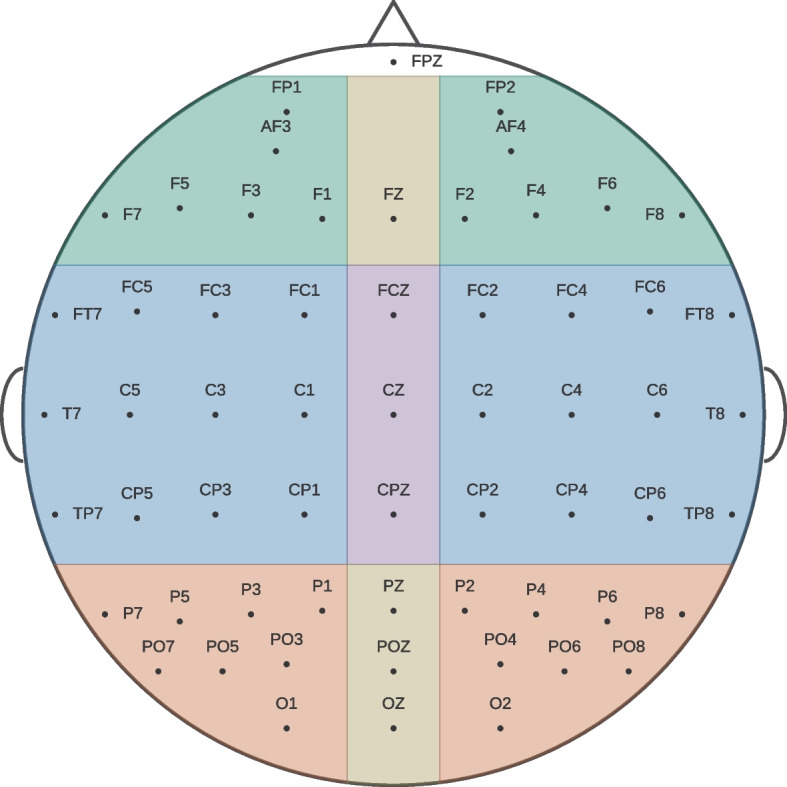
Scalp regions of interest used in the HEP and RBP analyses. The 64 EEG electrodes were grouped into nine scalp regions of interest (ROIs): LF (left frontal), RF (right frontal), LC (left central), RC (right central), LPO (left parieto-occipital), RPO (right parieto-occipital), CF (central frontal), CFP (central fronto-parietal), and CPO (central parieto-occipital). Mean regional values were used in the subsequent HEP and RBP analyses. These nine ROIs also served as the spatial units across which false discovery rate (FDR) correction was applied within each predefined analysis family. The electrode composition of each ROI is provided in Supplementary Table S1 [see Additional file 1]

EEG epochs were extracted from −100 to 700 ms relative to each R-wave, and baseline correction was applied using the −100 to 0 ms interval. Epochs exceeding 200 μV were rejected, and the remaining epochs were averaged to obtain HEP waveforms. To reduce channel-level redundancy and maintain consistency with the parent study, scalp electrodes were grouped into nine regions, and the mean potential within each region was used as the regional HEP amplitude. The electrodes included in each region are shown in Fig. 1. Based on the key time windows identified in the previous report using the same dataset, mean HEP amplitudes were extracted within two predefined windows, 235—301 ms and 263—381 ms, for subsequent correlation analyses [15].

### Power spectral density estimation and relative band power extraction

Power spectral density (PSD) was estimated using the Welch method [25]. To characterize frequency-specific resting-state neural oscillations, EEG signals were divided into four conventional bands: delta (0.5—4 Hz), theta (4—8 Hz), alpha (8—13 Hz), and beta (13—30 Hz) [15]. For each participant, condition, and scalp region, average band power (ABP) was calculated for each frequency band. RBP was then obtained by dividing band-specific ABP by the total power in the 0.5—30 Hz range, thereby reducing the influence of overall power fluctuations [26, 27]. To ensure spatial comparability with the HEP analyses, RBP values were summarized within the same nine scalp regions.

### Statistical analysis

All statistical analyses were conducted in R 4.5.3 [28]. Between-group differences in demographic, clinical, and questionnaire variables were examined using independent-samples *t* tests, Mann–Whitney *U* tests, or chi-square tests, as appropriate. Cohen's *d* or rank-biserial *r* was reported as the effect-size index [29, 30].

Given the fixed sample size and the secondary exploratory nature of the study, a sensitivity power analysis was conducted to contextualize the statistical sensitivity of the correlational analyses. With *α* = 0.05 and 80% power, the present sample could detect correlations of approximately *|r|*= 0.58 in the healthy-control group and *|r|*= 0.60 in the panic-disorder group. For age-adjusted partial correlations, the corresponding detectable effects were approximately *|r|*= 0.59 in the healthy-control group and *|r|*= 0.62 in the panic-disorder group. These estimates indicate that the study was primarily powered to detect medium-to-large associations.

Associations between self-report measures and electrophysiological indices were examined separately within the HC and PD groups using Pearson correlations. For each Pearson correlation, 95% confidence intervals (CIs) were computed using Fisher’s *z* transformation. Bayes factors for correlations were computed using the correlationBF function from the BayesFactor package in R with the default prior scale to quantify the relative evidence for a non-zero association versus the null hypothesis of no association [31]. Bayes factors were reported as BF_10_, where values greater than 3 were interpreted as moderate evidence for the alternative hypothesis, values greater than 10 as strong evidence, values below 1/3 as moderate evidence for the null hypothesis, and values between 1/3 and 3 as inconclusive.

Multiple-comparison correction was applied using the Benjamini–Hochberg FDR procedure within theoretically defined analysis families [32]. For HEP analyses, *p* values were adjusted separately within each group, predefined HEP time window, resting-state condition, and self-report construct across the nine scalp regions of interest. Although HEP correlations were computed for the two predefined windows identified in the parent study, the main text emphasizes the 235—301 ms window because it showed the clearest and most spatially coherent association patterns; the later 263—381 ms window is summarized separately. For RBP analyses, *p* values were adjusted separately within each group, resting-state condition, frequency band, and self-report construct across the nine scalp regions of interest. BPQ-VSF and each MAIA subscale were treated as separate self-report constructs because they index distinct dimensions of interoceptive experience. Results with *q* < 0.05 were considered FDR-corrected associations. Given the exploratory nature of the present secondary analysis, associations with 0.05 ≤ *q* < 0.10 were reported as exploratory FDR-level patterns when they showed directionally coherent distributions across scalp regions. Nominal associations that did not meet the exploratory FDR threshold are reported in the Supplementary Materials and were not interpreted as confirmatory findings.

The primary inferential analyses focused on the BPQ-VSF total score and the MAIA subscales that showed behavioral group differences between the panic-disorder and healthy-control groups: Not-Distracting, Not-Worrying, Emotional Awareness, and Trusting. These constructs were selected for primary interpretation because they were both theoretically relevant to panic-related interoceptive dysfunction and empirically differentiated the two groups at the questionnaire level. Other MAIA dimensions were not selected for primary interpretation because they did not show behavioral group differences.

To address the potential influence of age, age-adjusted sensitivity analyses were conducted using partial correlations controlling for age within each group. These analyses were used to evaluate whether the main self-report-EEG association patterns remained directionally consistent after accounting for age-related variability. In addition, formal between-group differences in association patterns were examined using linear models including self-report score, group, their interaction, and age as predictors. The group × self-report interaction term tested whether the association between a self-report construct and an EEG marker differed between panic-disorder and healthy-control participants after controlling for age. Interaction-model confidence intervals were computed for the interaction coefficients, and interaction *p* values were FDR-corrected across the nine scalp regions within the corresponding self-report construct, resting-state condition, and HEP time window or RBP frequency band.

## Results

### Demographic and clinical data

As shown in Table 1, the two groups did not differ significantly in age, sex, BMI, or years of education. Compared with HC, the PD group showed significantly higher HAMA, HAMD-17, and BPQ-VSF scores (all *p* < 0.001). Among the MAIA subscales, the PD group showed higher Emotional Awareness (*p* = 0.044), but lower Not-Distracting (*p* = 0.017), Not-Worrying (*p* < 0.001), and Trusting (*p* = 0.004). No significant between-group differences were observed for Noticing, Attention Regulation, Self-Regulation, or Body Listening.

**Table 1:** Demographic, clinical, and self-reported interoceptive characteristics of the study groups

Measure	PD (*n* = 19)	HC (*n* = 21)	Statistic	*p*	Effect size
*Demographics*					
Gender (F/M)	12/7	13/8	0.007	.935	
Age (years)	48.84 (14.1)	46 (11.2)	−0.709	.483	−0.222
BMI	22 (20.8, 24.9)	23.8 (21.2, 25.2)	−1.071	.285	−0.169
Education (years)	12 (9, 16)	15 (12, 16)	−0.963	.336	−0.152
*Clinical scales*					
HAMA	20 (18, 24)	4 (2, 6.5)	5.387	<.001	0.852
HAMD-17	19 (14, 21)	5 (4, 8.5)	4.850	<.001	0.767
PDSS	12.26 (3.59)	NA			
PASS	11.32 (2.93)	NA			
*Interoceptive measures*					
BPQ-VSF	24.68 (5.25)	17.38 (4.07)	−4.944	<.001	−1.549
MAIA					
Noticing	3.09 (0.95)	2.68 (1.27)	−1.156	.255	−0.369
Not-Distracting	2.32 (1.06)	3.30 (1.39)	2.504	.017	0.799
Not-Worrying	1.16 (0.88)	2.65 (1.18)	4.487	<.001	1.437
Attention Regulation	2.30 (1.11)	2.38 (1.26)	0.213	.833	0.068
Emotional Awareness	3 (2.60, 3.80)	2.20 (1.0, 3.1)	2.009	.044	0.318
Self-Regulation	1.91 (1.10)	2.6 (1.47)	1.660	.105	0.531
Body Listening	2.65 (1.09)	2.03 (1.44)	−1.517	.137	−0.486
Trusting	1.67 (0.33, 2.00)	3.33 (1.5, 4.5)	−2.858	.004	−0.452

Values are presented as mean (SD) for normally distributed variables and median (Q1, Q3) for non-normally distributed variables. Between-group comparisons were conducted using independent-samples *t* tests, Mann–Whitney *U* tests, or chi-square tests, as appropriate. Effect sizes are reported as Cohen’s *d* for parametric comparisons and rank-biserial *r* for nonparametric comparisons, where applicable. Group-difference confidence intervals for variables with between-group comparisons are provided in Supplementary Table S2. PDSS and PASS were assessed only in the PD group and were therefore not included in between-group comparisons

*HC* Healthy controls, *PD* Panic disorder, *BMI* Body mass index, *HAMA* Hamilton Anxiety Rating Scale, *HAMD-17* 17-item Hamilton Depression Rating Scale, *BPQ-VSF* Body Perception Questionnaire-Very Short Form, *MAIA* Multidimensional Assessment of Interoceptive Awareness, *PDSS* Panic Disorder Severity Scale, *PASS* Panic-Associated Symptom Scale, *NA* Not applicable

### Heartbeat-evoked potential correlations

Building on our previous report using the same resting-state EEG dataset, associations between self-reported interoceptive measures and HEP amplitudes were examined in two predefined time windows, 235—301 ms and 263—381 ms [15]. The main text focuses on the 235—301 ms interval because this window showed the clearest and most spatially coherent association patterns; the later 263—381 ms window is summarized briefly and reported in the Supplementary Materials.

In the 235—301 ms window, the clearest FDR-corrected within-group HEP association involved MAIA Not-Worrying in the PD group under EC. Higher Not-Worrying scores were positively associated with HEP amplitudes across all nine ROIs, surviving FDR correction at *q* < 0.05. Correlation coefficients ranged from* r* = 0.458 to *r* = 0.560 across ROIs, with BF_10_ values ranging from 1.73 to 5.14. The strongest association was observed in the right parieto-occipital region, *r* = 0.560, 95% CI [0.142, 0.809], *p* = 0.013, *q* = 0.046, BF_10_ = 5.14, followed by the right frontal region, *r* = 0.550, 95% CI [0.127, 0.803], *p* = 0.015, *q* = 0.046, BF_10_ = 4.51, and the left frontal region, *r* = 0.532, 95% CI [0.103, 0.794], *p* = 0.019, *q* = 0.046, BF_10_ = 3.67. No comparable FDR-corrected Not-Worrying-HEP pattern was observed in HC. Age-adjusted partial correlations in PD remained positive and moderate in magnitude across ROIs, although they did not reach the exploratory FDR threshold, indicating that this association pattern was directionally preserved but attenuated after controlling for age.

BPQ-VSF showed an exploratory opposite-direction HEP pattern under EO. In HC, BPQ-VSF scores were positively associated with HEP amplitudes across eight ROIs at the exploratory FDR level, with correlation coefficients ranging from *r* = 0.397 to *r* = 0.496. The strongest association was observed in the central fronto-parietal region, *r* = 0.496, 95% CI [0.082, 0.764], *p* = 0.022, *q* = 0.085, BF_10_ = 3.13. In PD, the pattern was reversed: BPQ-VSF scores were negatively associated with HEP amplitudes across seven ROIs at the exploratory FDR level, with correlation coefficients ranging from* r* = −0.511 to* r* = −0.424. The strongest association was again observed in the central fronto-parietal region, *r* = −0.511, 95% CI [−0.783, −0.073], *p* = 0.025, *q* = 0.091, BF_10_ = 2.90. The HC pattern remained at the exploratory FDR level after controlling for age, whereas the PD pattern remained directionally negative but did not meet the exploratory FDR threshold in the age-adjusted partial-correlation analyses.

Formal age-adjusted interaction models provided direct evidence that BPQ-VSF-HEP coupling differed between groups. Under EO, the group × BPQ-VSF interaction survived FDR correction across all nine ROIs, with negative interaction coefficients indicating a lower BPQ-VSF-HEP slope in PD than in HC. Interaction coefficients ranged from *β* = −0.271 to *β* = −0.385, with *q* values ranging from 0.007 to 0.018. The strongest interaction was observed in the central fronto-parietal region, *β* = −0.385, 95% CI [−0.602, −0.169], *p* < 0.001, *q* = 0.007. Under EC, group × BPQ-VSF interactions were also observed across most ROIs, with seven ROIs reaching *q* < 0.05 and one additional ROI reaching the exploratory *q* < 0.10 threshold. No MAIA dimension showed a comparable FDR-corrected group interaction for HEP. Thus, BPQ-VSF provided the clearest formal evidence for group-differentiated self-report-HEP coupling, whereas Not-Worrying provided the clearest FDR-corrected within-PD HEP association.

**Fig. 2:**
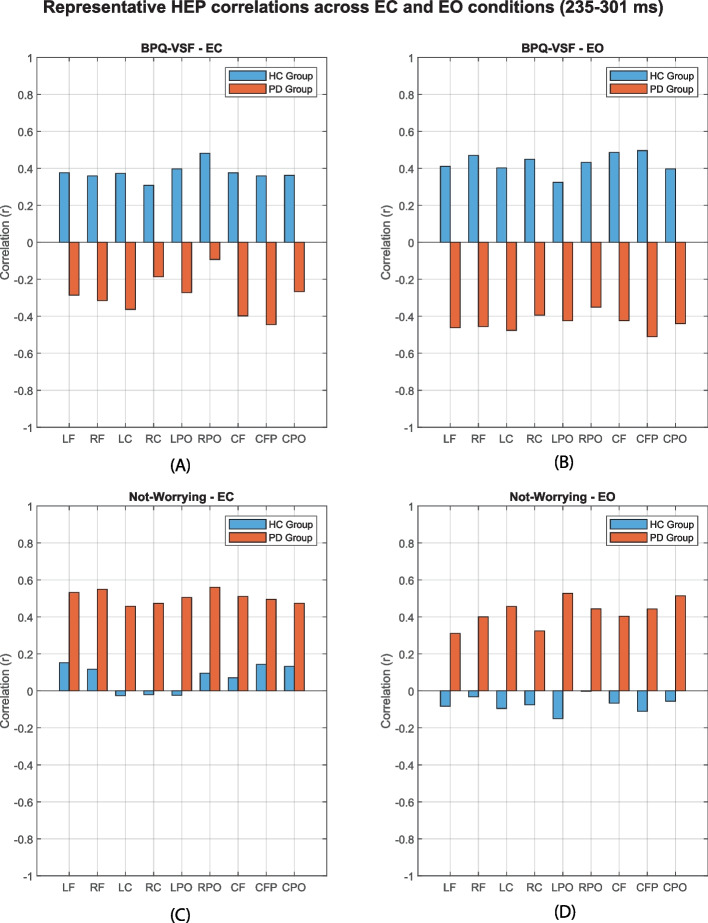
Core bar-plot results for associations between self-reported interoceptive measures and HEP amplitudes in the 235—301 ms time window. **A** BPQ-VSF-HEP associations under eyes closed (EC); (**B**) BPQ-VSF-HEP associations under eyes open (EO); (**C**) MAIA Not-Worrying-HEP associations under eyes closed (EC); (**D**) MAIA Not-Worrying-HEP associations under eyes open (EO). Bars represent Pearson correlation coefficients between self-reported interoceptive measures and mean HEP amplitudes across nine scalp ROIs in healthy controls (HC) and people with panic disorder (PD). Positive and negative values indicate positive and negative associations, respectively. BPQ-VSF = Body Perception Questionnaire-Very Short Form; HE*P* = heartbeat-evoked potential; MAIA = Multidimensional Assessment of Interoceptive Awareness; EC = eyes closed; EO = eyes open. Exact *r* values, 95% confidence intervals, *p* values, FDR-adjusted *q* values, Bayes factors, age-adjusted partial correlations, and interaction-model results are provided in the Supplementary Materials [see Additional file 1]

**Fig. 3:**
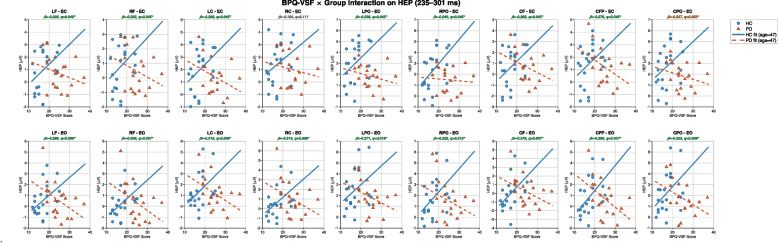
Age-adjusted group-differentiated BPQ-VSF–HEP coupling under eyes-closed and eyes-open conditions in the 235–301 ms time window. The upper row shows EC and the lower row shows EO. Each panel shows the association between BPQ-VSF scores and mean HEP amplitudes in one scalp ROI. Fitted lines represent group-specific model-predicted slopes from age-adjusted interaction models with age set to the sample mean. Panel titles report the group × BPQ-VSF interaction coefficient and FDR-adjusted *q* value. BPQ-VSF = Body Perception Questionnaire-Very Short Form; HEP = heartbeat-evoked potential; ROI = region of interest; EC = eyes closed; EO = eyes open

HEP findings for the remaining MAIA dimensions were more limited. Trusting and Emotional Awareness did not show stable FDR-corrected HEP associations. Not-Distracting showed a directionally coherent but statistically weaker pattern, with negative correlations in HC and positive correlations in PD across regions and conditions; these effects did not meet the FDR thresholds and are retained as supplementary descriptive findings. The later 263—381 ms window did not show comparably coherent patterns for the principal interpretation and is summarized in the Supplementary Materials [see Additional file 1] together with the complete HEP results (Figs. 2 and 3).

### Relative band power correlations

**Fig. 4:**
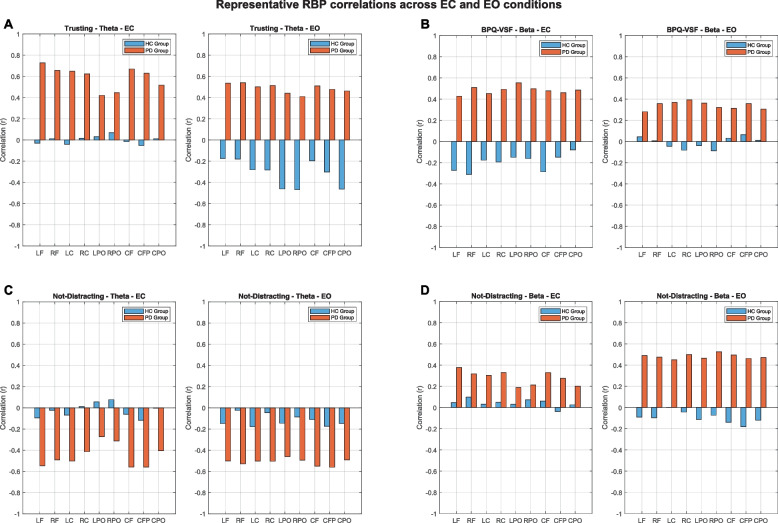
Principal relative-band-power association profiles retained in the main text. **A** MAIA Trusting-theta associations; (**B**) BPQ-VSF-beta associations; (**C**) MAIA Not-Distracting-theta associations; (**D**) MAIA Not-Distracting-beta associations. Bars represent Pearson correlation coefficients between self-reported interoceptive measures and relative band power across the nine scalp ROIs in healthy controls (HC) and participants with panic disorder (PD). Each panel shows both eyes-closed (EC) and eyes-open (EO) conditions. Positive and negative values indicate positive and negative associations, respectively. Age-adjusted partial-correlation and group-interaction results are reported in the text and Supplementary Materials [see Additional file 1]. BPQ-VSF = Body Perception Questionnaire-Very Short Form; MAIA = Multidimensional Assessment of Interoceptive Awareness; RBP = relative band power

RBP analyses showed band-specific self-report-EEG association profiles that were concentrated in the PD group. No HC RBP association showed comparable FDR-corrected or exploratory FDR-level patterns. Figure 4 displays the representative RBP patterns retained in the main text, and the complete RBP results are provided in the Supplementary Materials.

At the conventional *q* < 0.05 threshold, the strongest RBP findings involved theta-band power in PD. Trusting showed positive associations with theta power under EC across multiple ROIs. Seven ROIs survived FDR correction at *q* < 0.05, and all nine ROIs met the exploratory *q* < 0.10 threshold. Correlation coefficients ranged from *r* = 0.420 to *r* = 0.728 across ROIs, with BF_10_ values ranging from 1.27 to 91.44. The strongest association was observed in the left frontal region, *r* = 0.728, 95% CI [0.408, 0.888], *p* < 0.001, *q* = 0.004, BF_10_ = 91.44, followed by the central frontal region, *r* = 0.670, 95% CI [0.310, 0.862], *p* = 0.002, *q* = 0.006, BF_10_ = 27.34. Age-adjusted partial correlations remained positive and survived FDR correction across six ROIs, indicating that the Trusting-theta association was one of the most robust RBP patterns in PD.

Not-Distracting showed a widespread negative theta-band pattern in PD. Under EO, higher Not-Distracting scores were associated with lower theta power across all nine ROIs, with all ROIs surviving FDR correction at *q* < 0.05. Correlation coefficients ranged from *r* = −0.560 to *r* = −0.459, with BF_10_ values ranging from 1.75 to 5.08. The strongest associations were observed in the central fronto-parietal region, *r* = −0.560, 95% CI [−0.808, −0.141], *p* = 0.013, *q* = 0.037, BF_10_ = 5.08, and the central frontal region, *r* = −0.550, 95% CI [−0.803, −0.127], *p* = 0.015, *q* = 0.037, BF_10_ = 4.50. Under EC, negative Not-Distracting-theta associations were more focal, with three ROIs surviving *q* < 0.05 and five ROIs meeting *q* < 0.10. However, these Not-Distracting-theta patterns were attenuated after controlling for age and did not meet the exploratory FDR threshold in the age-adjusted partial-correlation analyses. Therefore, they were retained as FDR-corrected primary-correlation findings but interpreted cautiously in relation to age-adjusted robustness.

BPQ-VSF showed exploratory positive RBP associations in PD. Under EO, BPQ-VSF was positively associated with theta power across all nine ROIs at the exploratory *q* < 0.10 level. The strongest association was observed in the left frontal region, *r* = 0.507, 95% CI [0.069, 0.782], *p* = 0.027, *q* = 0.069, BF_10_ = 2.80. This theta-band pattern remained directionally positive after age adjustment but did not meet the exploratory FDR threshold. Under EC, BPQ-VSF was positively associated with beta power across all nine ROIs at the exploratory *q* < 0.10 level in the primary correlation analyses, with correlation coefficients ranging from *r* = 0.426 to *r* = 0.552. The strongest primary correlation was observed in the left parieto-occipital region, *r* = 0.552, 95% CI [0.131, 0.805], *p* = 0.014, *q* = 0.058, BF_10_ = 4.65. Importantly, after controlling for age, the BPQ-VSF-beta association under EC survived FDR correction across all nine ROIs, with partial correlations ranging from* r* = 0.477 to *r* = 0.600. The strongest age-adjusted association was again observed in the left parieto-occipital region, partial *r* = 0.600, 95% CI [0.184, 0.833], *p* = 0.009, *q* = 0.034. Thus, BPQ-VSF-beta under EC represented the most robust BPQ-VSF-related RBP pattern in PD.

Additional exploratory RBP patterns were observed for Trusting and Not-Distracting. Trusting was positively associated with alpha power under EO in PD across seven ROIs at the exploratory *q* < 0.10 level, with the strongest association in the left frontal region, *r* = 0.556, 95% CI [0.136, 0.807], *p* = 0.013, *q* = 0.081, BF_10_ = 4.87. This pattern did not remain FDR-level after controlling for age. Not-Distracting was positively associated with beta power under EO across all nine ROIs at the exploratory *q* < 0.10 level, with correlation coefficients ranging from *r* = 0.451 to *r* = 0.527. The strongest association was observed in the right parieto-occipital region, *r* = 0.527, 95% CI [0.095, 0.791], *p* = 0.021, *q* = 0.051, BF_10_ = 3.44. This beta-band pattern was also attenuated in the age-adjusted analyses.

Age-adjusted group-interaction models did not identify any RBP interaction that survived the conventional *q* < 0.05 threshold. However, exploratory group-differentiated RBP patterns were observed for BPQ-VSF-beta under EC and Trusting-theta under EC. For BPQ-VSF-beta, group × BPQ-VSF interactions reached the exploratory *q* < 0.10 threshold across eight ROIs, with positive interaction coefficients indicating stronger BPQ-VSF-beta coupling in PD than in HC. For Trusting-theta, group × Trusting interactions reached the exploratory *q* < 0.10 threshold across six ROIs under EC. These interaction results suggest possible group-differentiated RBP coupling, but they should be considered exploratory. Delta-band BPQ-VSF associations did not reach the FDR thresholds and were therefore not retained as principal RBP findings.

## Discussion

### Principal findings

The present study extended our previous report based on the same resting-state EEG dataset by examining how self-reported interoceptive experience maps onto electrophysiological indices in people with PD and HC [15]. After incorporating FDR correction, confidence intervals, Bayes factors, age-adjusted sensitivity analyses, and formal group-interaction models, four principal findings emerged.

First, the clearest formal evidence for altered self-report-EEG mapping was observed for BPQ-VSF-HEP coupling. In the 235—301 ms HEP window, BPQ-VSF showed an exploratory opposite-direction within-group pattern under EO, with positive associations in HC and negative associations in PD. More importantly, age-adjusted interaction models showed that the BPQ-VSF-HEP association differed between groups across scalp regions, especially under EO. Thus, the BPQ-VSF-HEP result is not only a descriptive contrast between separate within-group correlations, but provides direct evidence that body-focused attention maps differently onto cardiac-related cortical responses in PD and HC.

Second, the strongest FDR-corrected within-PD HEP association involved the MAIA Not-Worrying dimension under EC. Higher Not-Worrying scores were associated with larger HEP amplitudes across all scalp regions in PD. This finding suggests that, within PD, the tendency to experience bodily sensations in a less catastrophic or less worry-driven way is closely related to cortical processing of cardiac signals. Age-adjusted analyses preserved the positive direction of this pattern but attenuated its statistical strength, indicating that the Not-Worrying-HEP association should be interpreted as a robust primary-correlation finding that still requires confirmation in larger samples.

Third, the RBP findings were concentrated in the PD group and were most informative in theta- and beta-band profiles rather than across all frequency bands equally. Trusting showed the most stable MAIA-related RBP association: higher Trusting scores were positively associated with theta power in PD, and this pattern remained relatively robust after controlling for age. BPQ-VSF also showed a clinically relevant beta-band pattern in PD under EC; although this association was exploratory in the primary correlation analysis, it became FDR-corrected after age adjustment, suggesting that body-focused attention may be linked to beta-band resting-state profiles in PD. Not-Distracting showed a widespread negative theta-band association in the primary correlation analyses, but this pattern was attenuated after age adjustment and should therefore be interpreted cautiously.

Fourth, the overall pattern of findings supports a condition- and frequency-dependent account of self-report-EEG correspondence in PD. The strongest formal group-difference evidence was observed for BPQ-VSF-HEP coupling, whereas PD-specific associations were most robust for Not-Worrying-HEP, Trusting-theta power, and BPQ-VSF-beta power. Additional alpha- and beta-band patterns were retained as exploratory findings, whereas delta-band associations were not retained as principal results. Taken together, these findings suggest that PD is characterized less by a uniform increase or decrease in interoceptive processing than by altered mappings between subjective bodily experience and electrophysiological markers. These mappings appear to depend on the interoceptive construct being measured, the resting-state condition, and the electrophysiological feature under consideration.

### Altered coupling between self-report and electrophysiology in PD

The present findings are consistent with multidimensional accounts of interoception, which emphasize that self-report, behavioral performance, and neural indices need not converge linearly [1]. In the anxiety literature, meta-analytic evidence does not support a reliable association between anxiety and cardiac interoceptive accuracy; by contrast, anxiety is more consistently associated with self-reported interoceptive experience, especially perceived bodily sensitivity, negative evaluation of bodily signals, and negative attention toward bodily cues [5, 6]. Thus, the clinically relevant issue in PD may not be more accurate detection of bodily signals, but rather how bodily sensations are attended to, evaluated, and assigned threat value.

Within this framework, the BPQ-VSF-HEP finding provides the clearest evidence for altered coupling between subjective bodily focus and cardiac-related cortical responses. In the within-group analyses, BPQ-VSF showed an exploratory opposite-direction pattern under EO: higher BPQ-VSF scores were associated with larger HEP amplitudes in HC but smaller HEP amplitudes in PD. Importantly, the age-adjusted interaction models showed that the BPQ-VSF-HEP slopes differed between groups across scalp regions, especially under EO. This formal group-level test strengthens the interpretation that the BPQ-VSF-HEP pattern reflects altered self-report-HEP mapping in PD rather than merely separate within-group correlation trends.

BPQ-VSF indexes body-focused attention and symptom-related bodily awareness, whereas HEP reflects cortical responses time-locked to cardiac signals. In HC, the positive BPQ-VSF-HEP association suggests relative alignment between subjective bodily focus and cardiac-related cortical responses. In PD, however, the negative slope indicates that greater self-reported bodily focus does not straightforwardly correspond to larger HEP amplitudes. This pattern should not be interpreted as direct evidence of reduced neural processing efficiency. Rather, it suggests that in PD, heightened body-focused attention may be linked to a different organization of subjective and electrophysiological bodily-signal processing.

This interpretation is compatible with cognitive models of panic, in which panic symptoms are maintained not simply by stronger bodily signals, but by catastrophic interpretations of benign bodily changes [2]. It is also consistent with evidence that panic-related abnormalities may be more prominent at the level of metacognitive interoception and beliefs about bodily sensations than at the level of objective interoceptive sensitivity itself [7]. The present BPQ-VSF-HEP interaction therefore supports the view that interoceptive dysfunction in PD may involve a mismatch between subjective bodily salience and cardiac-related cortical responses.

At the same time, this interpretation should remain cautious. The within-group BPQ-VSF-HEP correlations were retained at the exploratory FDR level, and the sample size was modest. The strongest support for altered coupling comes from the age-adjusted group-interaction models rather than from confirmatory within-group correlations alone. Therefore, the BPQ-VSF-HEP pattern is best viewed as preliminary evidence for altered self-report-electrophysiology coupling in PD, requiring replication in larger samples and ideally in designs that combine resting-state measures with active interoceptive tasks.

### Why not-worrying, trusting, and not-distracting stood out

The MAIA findings further support the view that interoceptive dysfunction in PD is best understood at the level of specific appraisal and regulatory dimensions rather than as a single global bodily-awareness construct. Unlike BPQ-VSF, which mainly captures body-focused attention and symptom-related bodily awareness, MAIA separates how bodily sensations are noticed, evaluated, trusted, and regulated. This distinction is clinically relevant because anxiety and panic symptoms are not consistently associated with all self-report interoceptive dimensions equally, but are more closely linked to negative evaluation of bodily signals, negative attention toward bodily sensations, and difficulty relating to bodily experience in a non-threatening way [6].

Among the MAIA dimensions, Not-Worrying provided the clearest HEP-related pattern. In PD, higher Not-Worrying scores were associated with larger HEP amplitudes under EC across scalp regions. This finding suggests that the tendency to experience bodily sensations with less worry or catastrophic appraisal is linked to cardiac-related cortical processing in PD. The EC predominance of this association is theoretically meaningful: when external visual input is reduced, internally oriented bodily appraisal may become more closely coupled with cardiac-signal processing. However, because the age-adjusted sensitivity analyses attenuated the statistical strength of this pattern, the Not-Worrying-HEP association should be interpreted as a robust primary-correlation finding that remains preliminary with respect to age-adjusted stability.

Trusting showed the most stable MAIA-related RBP association. In PD, higher Trusting scores were positively associated with theta power under EC, and this association remained relatively robust after controlling for age. Trusting reflects the extent to which bodily sensations are experienced as safe, reliable, and tolerable rather than threatening or disruptive. The present finding therefore suggests that the subjective sense of bodily trust may be linked to resting-state theta-band profiles in PD. This does not imply that theta power has a fixed or exclusive psychological meaning; rather, it indicates that among the frequency-domain measures examined here, theta-band relative power showed the clearest association with bodily trust in the PD group.

Not-Distracting also emerged as a relevant dimension, although its interpretation requires more caution. In the primary correlation analyses, Not-Distracting showed widespread negative associations with theta power in PD, especially under EO, and exploratory positive associations with beta power. This dimension indexes how individuals respond when unpleasant bodily sensations arise, particularly whether they can remain with such sensations rather than urgently disengaging from them. The combined theta- and beta-band patterns suggest that responses to bodily discomfort may be related to broader resting-state spectral profiles in PD. However, because the Not-Distracting-related RBP patterns were attenuated after age adjustment, they should be considered exploratory and age-sensitive rather than definitive evidence for a stable oscillatory signature.

Taken together, these MAIA findings indicate that the self-report dimensions most relevant to PD-related EEG associations are not simple bodily noticing, but appraisal- and regulation-related dimensions: worrying less about bodily sensations, trusting the body, and responding less avoidantly to unpleasant bodily cues. The absence of stable EEG associations for some other MAIA dimensions, including Emotional Awareness despite its behavioral group difference, further suggests that not every questionnaire-level difference maps directly onto resting-state electrophysiological markers. This reinforces the importance of distinguishing between behavioral group differences in self-report scales and cross-domain self-report-EEG association patterns.

### State dependence and frequency specificity

The present findings also indicate that self-report-EEG correspondence in PD depends on resting-state condition. The BPQ-VSF-HEP pattern was most pronounced under EO, where the age-adjusted group × BPQ-VSF interaction survived FDR correction across scalp regions. EO may place greater demands on balancing external monitoring with internally salient bodily sensations. In this context, the altered BPQ-VSF-HEP mapping in PD may reflect a state in which body-focused attention competes with ongoing external sensory engagement. By contrast, the strongest Not-Worrying-HEP association appeared under EC, a condition in which reduced visual input may facilitate inwardly oriented bodily appraisal. Thus, the HEP findings suggest that subjective-neural correspondence is not fixed across resting states, but varies according to the attentional context in which bodily signals are processed.

This state-dependent interpretation is consistent with previous HEP work showing that HEP amplitudes are sensitive to attentional state, arousal, and experimental context [11]. Prior studies in generalized anxiety disorder reported altered EC-EO modulation of HEP and abnormal HEP responses during peripheral adrenergic stimulation [12, 13]. Although the present study did not include active interoceptive or pharmacological challenges, the pattern observed here suggests that resting-state condition may still modulate how self-reported bodily experience relates to cardiac-related cortical responses. The EO predominance of BPQ-VSF-HEP group differences and the EC predominance of Not-Worrying-HEP associations therefore point to complementary state-dependent aspects of interoceptive processing in PD.

The RBP findings further suggest frequency-specific association profiles, but the revised evidence is more selective than a broad cross-band account. The most stable RBP patterns were concentrated in theta and beta bands. Trusting showed a relatively robust positive association with theta power in PD, especially under EC, whereas BPQ-VSF showed a positive beta-band association under EC that became FDR-corrected after age adjustment. Not-Distracting showed a negative theta-band association in the primary correlation analyses and exploratory beta-band associations, but these patterns were more sensitive to age adjustment. Alpha-band associations involving Trusting were retained only as exploratory patterns, and delta-band BPQ-VSF associations were not retained as principal findings.

This more selective frequency pattern is important for interpretation. Current evidence does not support assigning each resting-state EEG band a fixed and exclusive psychological meaning, and large-scale reviews emphasize heterogeneity and overlap across psychiatric conditions [14]. Moreover, because RBP is a relative measure, changes in one band are not independent of the distribution of power across other bands. Accordingly, the present RBP findings are best interpreted as frequency-specific relative-power profiles rather than as direct markers of isolated cognitive or affective mechanisms. Within this cautious framework, theta-band associations may be most relevant to bodily trust and responses to bodily discomfort, whereas beta-band associations may be more closely linked to body-focused attention in PD.

These condition- and frequency-specific findings complement our previous group-level report based on the same dataset. The parent study emphasized impaired HEP modulation and elevated beta-band power in PD at the between-group level [15]. The present analysis adds a cross-domain perspective by showing how these electrophysiological indices relate to specific self-reported interoceptive constructs within each group and how some of these associations differ between groups. Therefore, the added value of the current study is not simply the identification of EEG abnormalities in PD, but the demonstration that subjective interoceptive experience and resting-state EEG markers are linked in a construct-, state-, and frequency-dependent manner.

### Clinical implications and limitations

The present findings have several clinical implications, although they should be considered preliminary. First, interoceptive abnormalities in PD may not be adequately characterized by self-report questionnaires or electrophysiological markers alone. Self-report measures capture body-focused attention, bodily worry, bodily trust, and responses to unpleasant bodily sensations, whereas EEG indices capture temporally and spectrally specific aspects of resting-state neural activity. Considering both levels together may help identify forms of subjective-neural mismatch that are not visible from either level in isolation. This is consistent with cognitive models of panic, which emphasize that panic-related distress is maintained not only by bodily arousal itself, but also by catastrophic interpretations and threat-related appraisal of bodily sensations [2].

Second, the present findings point to clinically relevant interoceptive dimensions that may be especially important for PD. The strongest formal group-difference evidence involved BPQ-VSF-HEP coupling, suggesting that body-focused attention may map differently onto cardiac-related cortical responses in PD and HC. Within the PD group, Not-Worrying-HEP, Trusting-theta power, and BPQ-VSF-beta power were among the most robust self-report-EEG association patterns. These findings suggest that future clinical work may benefit from distinguishing between heightened bodily attention, catastrophic or worry-driven appraisal, reduced bodily trust, and responses to bodily discomfort. Such distinctions are relevant for interventions targeting panic-related interoceptive processes, including cognitive restructuring of catastrophic bodily interpretations and interoceptive exposure. Existing component network meta-analytic evidence also suggests that interoceptive exposure is an important component of cognitive-behavioral therapy packages for panic disorder [33].

At the same time, the present data do not justify direct clinical or neuromodulatory recommendations. In particular, the RBP findings should not be interpreted as evidence for frequency-specific treatment targets. The results are better viewed as hypothesis-generating evidence that particular self-reported interoceptive dimensions may correspond to specific resting-state EEG profiles in PD. Future studies should test whether these self-report-EEG association patterns predict symptom severity, treatment response, or longitudinal change.

Several limitations should be acknowledged. First, the sample size was modest. The sensitivity power analysis indicated that the present study was primarily powered to detect medium-to-large correlations, meaning that smaller but potentially meaningful associations may have been missed. This limitation is particularly relevant for effects that were retained only at the exploratory *q* < 0.10 threshold or that were attenuated after age adjustment. Replication in larger independent samples is therefore essential.

Second, this was a secondary exploratory analysis with a correlational design. Although FDR correction, confidence intervals, Bayes factors, age-adjusted sensitivity analyses, and formal group-interaction models were added to improve statistical transparency and robustness, the results cannot establish causal relations between self-reported interoception and EEG markers. The *q* < 0.10 findings should be treated as exploratory FDR-level patterns rather than confirmatory effects.

Third, age was included as a covariate in sensitivity and interaction analyses, but the present study was not designed to investigate age-related mechanisms. Some association patterns remained stable after age adjustment, whereas others, particularly some Not-Distracting-related RBP findings, were attenuated. These results suggest that age-related variability should be considered in future work, but the current sample does not allow strong conclusions about age as a mechanistic factor.

Fourth, the study relied exclusively on resting-state EEG indices. Resting-state HEP and RBP are useful for characterizing baseline self-report-EEG association patterns, but they do not directly measure task-evoked interoceptive accuracy or active interoceptive attention. Future studies should combine resting-state recordings with experimentally accessible interoceptive tasks, such as heartbeat detection, heartbeat discrimination, heartbeat counting, or interoceptive attention paradigms. Such designs would clarify whether the altered self-report-EEG mappings observed here also appear during active bodily-signal monitoring.

Finally, the present analyses were conducted at the scalp ROI level and did not include source localization. Therefore, interpretations regarding specific neural generators, hemispheric lateralization, or circuit-level mechanisms should remain restrained. Future studies using larger samples, source-resolved EEG or multimodal neuroimaging, and longitudinal clinical designs would be valuable for determining whether altered self-report-EEG mappings represent stable markers of PD, treatment-sensitive mechanisms, or state-dependent features of panic-related bodily experience.

## Conclusions

By integrating self-reported interoceptive measures with resting-state HEP and RBP indices, the present study provides preliminary FDR-controlled and age-informed evidence that PD involves altered mappings between subjective bodily experience and electrophysiological markers. The clearest formal group-difference evidence was observed for BPQ-VSF-HEP coupling, particularly under EO, suggesting that body-focused attention relates differently to cardiac-related cortical responses in PD and HC. Within the PD group, the most robust association patterns involved Not-Worrying-HEP under EC, Trusting-theta power under EC, and BPQ-VSF-beta power under EC. Additional Not-Distracting-related theta and beta patterns were observed but were more sensitive to age adjustment and should be interpreted as exploratory. Overall, the findings suggest that interoceptive dysfunction in PD is not best characterized as a uniform increase or decrease in bodily-signal processing. Instead, PD may involve construct-specific, state-dependent, and frequency-specific self-report-EEG mappings. These results highlight the value of combining multidimensional self-report measures with electrophysiological indices when studying interoception in panic disorder. Given the modest sample size, exploratory correlational design, and reliance on resting-state EEG, replication in larger samples and extension to active interoceptive task paradigms are needed.

## Supplementary Information

Additional file 1: Supplementary Table S1. Electrode composition of the nine ROIs used in the HEP and RBP analyses. Supplementary Table S2. Group-difference confidence intervals for demographic, clinical, and questionnaire comparisons. Supplementary Table S3. Sensitivity power analysis for Pearson and age-adjusted partial correlation analyses. Supplementary Table S3. Sensitivity power analysis for Pearson and age-adjusted partial correlation analyses. Supplementary Table S5. HEP age-adjusted partial correlation and group-interaction results. Supplementary Table S6. RBP Pearson correlation results with FDR correction, confidence intervals, and Bayes factors. Supplementary Table S7. RBP age-adjusted partial correlation and group-interaction results. Supplementary Figure S1. Complete HEP correlation bar plots in the 235–301 ms time window. Supplementary Figure S2. Complete HEP correlation bar plots in the 263–381 ms time window. Supplementary Figure S2. Complete HEP correlation bar plots in the 263–381 ms time window.

## Data Availability

The datasets generated and analyzed during the current study are not publicly available due to participant privacy and the sensitivity of clinical and electrophysiological data, but are available from the corresponding author on reasonable request.

## Abbreviations

ABP: Average band power
BF_10_: Bayes factor in favor of the alternative hypothesis over the null hypothesis
BH: Benjamini–Hochberg
BMI: Body mass index
BPQ-VSF: Body Perception Questionnaire-Very Short Form
CBT: Cognitive behavioral therapy
CF: Central frontal
CFP: Central fronto-parietal
CI: Confidence interval
CPO: Central parieto-occipital
DSM-5: Diagnostic and Statistical Manual of Mental Disorders, Fifth Edition
EC: Eyes closed
ECG: Electrocardiography
EEG: Electroencephalography
EO: Eyes open
FDR: False discovery rate
FIR: Finite impulse response
GAD: Generalized anxiety disorder
HAMA: Hamilton Anxiety Rating Scale
HAMD-17: 17-Item Hamilton Depression Rating Scale
HC: Healthy controls
HEP: Heartbeat-evoked potential
ICA: Independent component analysis
LC: Left central
LF: Left frontal
LPO: Left parieto-occipital
MAIA: Multidimensional Assessment of Interoceptive Awareness
NA: Not applicable
PASS: Panic-Associated Symptom Scale
PD: Panic disorder
PDSS: Panic Disorder Severity Scale
PSD: Power spectral density
Q1: First quartile
Q3: Third quartile
RBP: Relative band power
RC: Right central
RF: Right frontal
ROI: Region of interest
RPO: Right parieto-occipital
SD: Standard deviation
