# Supplementary material for: Associations between self-reported interoception and resting-state EEG markers in panic disorder: heartbeat-evoked potentials and spectral power
Source: BMC Psychol. 2026 May 12;14:977. doi: 10.1186/s40359-026-04736-7 (PMC13334908; doi:10.1186/s40359-026-04736-7)
Supplement: Supplementary file 1 — Additional file 1: Supplementary Table S1. Electrode composition of the nine ROIs used in the HEP and RBP analyses. Supplementary Table S2. Group-difference confidence intervals for demographic, clinical, and questionnaire comparisons. Supplementary Table S3. Sensitivity power analysis for Pearson and age-adjusted partial correlation analyses. Supplementary Table S3. Sensitivity power analysis for Pearson and age-adjusted partial correlation analyses. Supplementary Table S5. HEP age-adjusted partial correlation and group-interaction results. Supplementary Table S6. RBP Pearson correlation results with FDR correction, confidence intervals, and Bayes factors. Supplementary Table S7. RBP age-adjusted partial correlation and group-interaction results. Supplementary Figure S1. Complete HEP correlation bar plots in the 235–301 ms time window. Supplementary Figure S2. Complete HEP correlation bar plots in the 263–381 ms time window. Supplementary Figure S2. Complete HEP correlation bar plots in the 263–381 ms time window. [file 40359_2026_4736_MOESM1_ESM.pdf]

Supplementary Materials

for

Associations Between Self-Reported Interoception and Resting-State EEG Markers in Panic Disorder: Heartbeat-Evoked Potentials and Spectral Power

Ziheng Gao[a,b,c], Zhiwan Xiong[d,e] , Meng Li[b], Yi Chang[d], Tommi Kärkkäinen[b], Fengyu Cong[a,b,c,f], Xiaoshuang Wang[a,c,*]

[a] Central Hospital of Dalian University of Technology, Dalian, China

[b] Faculty of Information Technology, University of Jyvaskylä, Jyväskylä, Finland

[c] School of Biomedical Engineering, Faculty of Medicine, Dalian University of Technology, Dalian, China

[d] Department of Neurology and Psychiatry, First Affiliated Hospital, Dalian Medical University, Dalian, China

[e] Center for Mental Health Education， Nanchang University College of Science and Technology, Jiujiang, China

[f] Key Laboratory of Social Computing and Cognitive Intelligence (Dalian University of Technology), Ministry of Education, Dalian, China

*Corresponding author: xswang@dlut.edu.cn (Xiaoshuang Wang)

Contents

Supplementary Table S1. Electrode composition of the nine ROIs used in the HEP and RBP analyses.

Supplementary Table S2. Group-difference confidence intervals for demographic, clinical, and questionnaire comparisons.

Supplementary Table S3. Sensitivity power analysis for Pearson and age-adjusted partial correlation analyses.

Supplementary Table S4. HEP Pearson correlation results with FDR correction, confidence intervals, and Bayes factors.

Supplementary Table S5. HEP age-adjusted partial correlation and group-interaction results.

Supplementary Table S6. RBP Pearson correlation results with FDR correction, confidence intervals, and Bayes factors.

Supplementary Table S7. RBP age-adjusted partial correlation and group-interaction results.

Supplementary Figure S1. Complete HEP correlation bar plots in the 235–301 ms time window.

Supplementary Figure S2. Complete HEP correlation bar plots in the 263–381 ms time window.

Supplementary Figure S3. Complete RBP correlation bar plots for the primary self-report constructs and frequency bands.

Supplementary Table S1

Supplementary Table S1 Electrode composition of the nine ROIs used in the HEP and RBP analyses

ROI	Region Name	Electrodes	N
LF	Left Frontal	FP1, AF3, F1, F3, F5, F7	6
RF	Right Frontal	FP2, AF4, F2, F4, F6, F8	6
CF	Central Frontal	FZ	1
LC	Left Central	FC1, FC3, FC5, FT7, C1, C3, C5, T7, CP1, CP3, CP5, TP7	12
RC	Right Central	FC2, FC4, FC6, FT8, C2, C4, C6, T8, CP2, CP4, CP6, TP8	12
CFP	Central Fronto-Parietal	FCZ, CZ, CPZ	3
LPO	Left Parieto-Occipital	P1, P3, P5, P7, PO3, PO5, PO7, O1	8
RPO	Right Parieto-Occipital	P2, P4, P6, P8, PO4, PO6, PO8, O2	8
CPO	Central Parieto-Occipital	PZ, POZ, OZ	3

Note. N = number of electrodes per region. LF = left frontal; RF = right frontal; CF = central frontal; LC = left central; RC = right central; CFP = central fronto-parietal; LPO = left parieto-occipital; RPO = right parieto-occipital; CPO = central parieto-occipital. Total: 59 electrodes across 9 ROIs (FPZ excluded as reference-adjacent midline electrode).

Supplementary Table S2

Supplementary Table S2. Group-difference confidence intervals for demographic, clinical, and questionnaire comparisons

Measure	PD value	HC value	Test	Statistic	*p*	Effect size	Difference type	Direction	Estimate	95% CI lower	95% CI upper
**Gender (F/M)**	12F / 7M	13F / 8M	Chi-square	0.007	.935	-	Categorical comparison	-	N/A	N/A	N/A
**Age (years)**	48.84 (14.1)	46.00 (11.2)	Welch's *t*-test	-0.709	.483	-0.222	Mean difference	HC - PD	-2.842	-11.083	5.399
**BMI**	22.00 (20.8, 24.9)	23.81 (21.2, 25.2)	Mann-Whitney *U*	-1.071	.285	-0.169	Hodges–Lehmann shift	HC - PD	0.970	0.160	1.900
**Education (years)**	12.00 (9, 16)	15.00 (12, 16)	Mann-Whitney *U*	-0.963	.336	-0.152	Hodges–Lehmann shift	HC - PD	0.000	0.000	3.000
**HAMA**	20.00 (18, 24)	4.00 (2, 6.5)	Mann-Whitney *U*	5.387	< .001	0.852	Hodges–Lehmann shift	HC - PD	-16.000	-17.000	-15.000
**HAMD-17**	19.00 (14, 21)	5.00 (4, 8.5)	Mann-Whitney *U*	4.850	< .001	0.767	Hodges–Lehmann shift	HC - PD	-12.000	-14.000	-10.000
**PDSS**	12.26 (3.59)	N/A	-	-	-	-	PD only	-	-	-	-
**PASS**	11.32 (2.93)	N/A	-	-	-	-	PD only	-	-	-	-
**BPQ-VSF**	24.68 (5.25)	17.38 (4.07)	Welch's *t*-test	-4.944	< .001	-1.549	Mean difference	HC - PD	-7.303	-10.344	-4.262
**Noticing**	3.09 (0.95)	2.68 (1.27)	Welch's *t*-test	-1.156	.255	-0.369	Mean difference	HC - PD	-0.414	-1.129	0.301
**Not-Distracting**	2.32 (1.06)	3.30 (1.39)	Welch's *t*-test	2.504	.017	0.799	Mean difference	HC - PD	0.986	0.199	1.773
**Not-Worrying**	1.16 (0.88)	2.65 (1.18)	Welch's *t*-	4.487	< .001	1.437	Mean	HC - PD	1.493	0.828	2.158

			test				difference				
**Attention Regulation**	2.30 (1.11)	2.38 (1.26)	Welch's *t*-test	0.213	.833	0.068	Mean difference	HC - PD	0.080	-0.677	0.838
**Emotional Awareness**	3.00 (2.60, 3.80)	2.20 (1.0, 3.1)	Mann-Whitney *U*	2.009	.044	0.318	Hodges–Lehmann shift	HC - PD	-0.800	-1.200	-0.400
**Self-Regulation**	1.91 (1.10)	2.60 (1.47)	Welch's *t*-test	1.660	.105	0.531	Mean difference	HC - PD	0.687	-0.140	1.514
**Body Listening**	2.65 (1.09)	2.03 (1.44)	Welch's *t*-test	-1.517	.137	-0.486	Mean difference	HC - PD	-0.617	-1.430	0.196
**Trusting**	1.67 (0.33, 2.00)	3.33 (1.5, 4.5)	Mann-Whitney *U*	-2.858	.004	-0.452	Hodges–Lehmann shift	HC - PD	2.000	1.000	2.333

Note. Group differences are expressed as PD - HC. For normally distributed variables, the group-difference estimate represents the mean difference. For non-normally distributed variables, the group-difference estimate represents the Hodges–Lehmann location shift. Effect sizes are reported as Cohen’s d for parametric comparisons and rank-biserial r for nonparametric comparisons. PDSS and PASS were assessed only in the PD group and were not included in between-group comparisons.

Supplementary Table S3

Supplementary Table S3. Sensitivity power analysis for Pearson and age-adjusted partial correlation analyses

Analysis	Group	n	Covariates	Alpha	Power	Minimum detectable |r|
Pearson correlation	HC	21	0	.05	.80	.579
	PD	19	0	.05	.80	.605
Age-adjusted partial correlation	HC	21	1	.05	.80	.591
	PD	19	1	.05	.80	.619

Note. Sensitivity power analysis was conducted to determine the minimum detectable correlation coefficient given the fixed sample size, $\alpha = .05$, and 80% power. For age-adjusted partial correlations, age was treated as one covariate. HC = healthy controls; PD = panic disorder.

Supplementary Table S4

Supplementary Table S4. HEP Pearson correlation results with FDR correction, confidence intervals, and Bayes factors.

FDR_family	FDR_ family_size	Region	*r*	CI_95_ lower	CI_95_ upper	*p*	*q*_FDR	BF_{10}	Evidence_level
BPQ-VSF | HC | EC | 235-301 ms	9	LF	0.376	-0.066	0.695	0.093	0.124	1.018	No association
BPQ-VSF | HC | EC | 235-301 ms	9	RF	0.359	-0.086	0.684	0.110	0.124	0.894	No association
BPQ-VSF | HC | EC | 235-301 ms	9	LC	0.372	-0.071	0.693	0.096	0.124	0.988	No association
BPQ-VSF | HC | EC | 235-301 ms	9	RC	0.309	-0.142	0.653	0.173	0.173	0.645	No association
BPQ-VSF | HC | EC | 235-301 ms	9	LPO	0.396	-0.042	0.707	0.075	0.124	1.192	No association
BPQ-VSF | HC | EC | 235-301 ms	9	RPO	0.482	0.063	0.756	0.027	0.124	2.669	Nominal $p < .05$ only
BPQ-VSF | HC | EC | 235-301 ms	9	CF	0.376	-0.067	0.695	0.093	0.124	1.012	No association
BPQ-VSF | HC | EC | 235-301 ms	9	CFP	0.359	-0.086	0.685	0.110	0.124	0.895	No association
BPQ-VSF | HC | EC | 235-301 ms	9	CPO	0.362	-0.082	0.687	0.106	0.124	0.918	No association
BPQ-VSF | PD | EC | 235-301 ms	9	LF	-0.286	-0.655	0.193	0.235	0.349	0.548	No association
BPQ-VSF | PD | EC | 235-301 ms	9	RF	-0.315	-0.673	0.163	0.189	0.349	0.634	No association
BPQ-VSF | PD | EC | 235-301 ms	9	LC	-0.363	-0.701	0.109	0.127	0.349	0.841	No association
BPQ-VSF | PD | EC | 235-301 ms	9	RC	-0.187	-0.591	0.292	0.444	0.499	0.373	No association
BPQ-VSF | PD | EC | 235-301 ms	9	LPO	-0.272	-0.646	0.208	0.260	0.349	0.512	No association
BPQ-VSF | PD | EC | 235-301 ms	9	RPO	-0.093	-0.525	0.377	0.705	0.705	0.303	No association
BPQ-VSF | PD | EC | 235-301 ms	9	CF	-0.398	-0.721	0.069	0.092	0.349	1.067	No association
BPQ-VSF | PD | EC | 235-301 ms	9	CFP	-0.444	-0.748	0.013	0.057	0.349	1.539	No association
BPQ-VSF | PD | EC | 235-301 ms	9	CPO	-0.266	-0.643	0.214	0.271	0.349	0.499	No association
BPQ-VSF | HC | EO | 235-301 ms	9	LF	0.411	-0.025	0.716	0.064	0.085	1.349	Exploratory FDR-level $.05 \leq q < .10$
BPQ-VSF | HC | EO | 235-301 ms	9	RF	0.470	0.048	0.749	0.032	0.085	2.350	Exploratory FDR-level $.05 \leq q < .10$
BPQ-VSF | HC | EO | 235-301 ms	9	LC	0.402	-0.035	0.711	0.071	0.085	1.252	Exploratory FDR-level $.05 \leq q < .10$

FDR_family	FDR_ family_size	Region	*r*	CI_95_ lower	CI_95_ upper	*p*	*q*_FDR	BF_{10}	Evidence_level
BPQ-VSF | HC | EO | 235-301 ms	9	RC	0.449	0.022	0.738	0.041	0.085	1.909	Exploratory FDR-level $.05 \leq q < .10$
BPQ-VSF | HC | EO | 235-301 ms	9	LPO	0.325	-0.124	0.663	0.151	0.151	0.711	No association
BPQ-VSF | HC | EO | 235-301 ms	9	RPO	0.432	0.000	0.728	0.051	0.085	1.621	Exploratory FDR-level $.05 \leq q < .10$
BPQ-VSF | HC | EO | 235-301 ms	9	CF	0.486	0.069	0.758	0.026	0.085	2.794	Exploratory FDR-level $.05 \leq q < .10$
BPQ-VSF | HC | EO | 235-301 ms	9	CFP	0.496	0.082	0.764	0.022	0.085	3.131	Exploratory FDR-level $.05 \leq q < .10$
BPQ-VSF | HC | EO | 235-301 ms	9	CPO	0.397	-0.042	0.707	0.075	0.085	1.193	Exploratory FDR-level $.05 \leq q < .10$
BPQ-VSF | PD | EO | 235-301 ms	9	LF	-0.462	-0.757	-0.010	0.046	0.091	1.801	Exploratory FDR-level $.05 \leq q < .10$
BPQ-VSF | PD | EO | 235-301 ms	9	RF	-0.455	-0.753	-0.001	0.050	0.091	1.690	Exploratory FDR-level $.05 \leq q < .10$
BPQ-VSF | PD | EO | 235-301 ms	9	LC	-0.476	-0.765	-0.028	0.039	0.091	2.055	Exploratory FDR-level $.05 \leq q < .10$
BPQ-VSF | PD | EO | 235-301 ms	9	RC	-0.394	-0.719	0.073	0.095	0.107	1.040	No association
BPQ-VSF | PD | EO | 235-301 ms	9	LPO	-0.424	-0.736	0.037	0.070	0.091	1.306	Exploratory FDR-level $.05 \leq q < .10$
BPQ-VSF | PD | EO | 235-301 ms	9	RPO	-0.351	-0.694	0.123	0.141	0.141	0.780	No association
BPQ-VSF | PD | EO | 235-301 ms	9	CF	-0.424	-0.736	0.038	0.071	0.091	1.300	Exploratory FDR-level $.05 \leq q < .10$
BPQ-VSF | PD | EO | 235-301 ms	9	CFP	-0.511	-0.783	-0.073	0.025	0.091	2.896	Exploratory FDR-level $.05 \leq q < .10$
BPQ-VSF | PD | EO | 235-301 ms	9	CPO	-0.439	-0.745	0.018	0.060	0.091	1.479	Exploratory FDR-level $.05 \leq q < .10$
Not-Distracting | HC | EC | 235-301 ms	9	LF	-0.238	-0.607	0.216	0.298	0.350	0.449	No association
Not-Distracting | HC | EC | 235-301 ms	9	RF	-0.288	-0.640	0.164	0.205	0.350	0.575	No association
Not-Distracting | HC | EC | 235-301 ms	9	LC	-0.279	-0.634	0.174	0.221	0.350	0.546	No association
Not-Distracting | HC | EC | 235-301 ms	9	RC	-0.257	-0.620	0.196	0.260	0.350	0.490	No association
Not-Distracting | HC | EC | 235-301 ms	9	LPO	-0.186	-0.572	0.267	0.420	0.420	0.367	No association

FDR_family	FDR_ family_size	Region	*r*	CI_95_ lower	CI_95_ upper	*p*	*q*_FDR	BF_{10}	Evidence_level
Not-Distracting | HC | EC | 235-301 ms	9	RPO	-0.371	-0.692	0.072	0.097	0.350	0.980	No association
Not-Distracting | HC | EC | 235-301 ms	9	CF	-0.267	-0.626	0.186	0.242	0.350	0.513	No association
Not-Distracting | HC | EC | 235-301 ms	9	CFP	-0.363	-0.687	0.081	0.105	0.350	0.924	No association
Not-Distracting | HC | EC | 235-301 ms	9	CPO	-0.232	-0.604	0.222	0.311	0.350	0.438	No association
Not-Distracting | PD | EC | 235-301 ms	9	LF	0.156	-0.321	0.570	0.522	0.555	0.343	No association
Not-Distracting | PD | EC | 235-301 ms	9	RF	0.247	-0.233	0.631	0.307	0.555	0.461	No association
Not-Distracting | PD | EC | 235-301 ms	9	LC	0.201	-0.279	0.600	0.410	0.555	0.390	No association
Not-Distracting | PD | EC | 235-301 ms	9	RC	0.240	-0.241	0.626	0.323	0.555	0.448	No association
Not-Distracting | PD | EC | 235-301 ms	9	LPO	0.243	-0.238	0.628	0.316	0.555	0.453	No association
Not-Distracting | PD | EC | 235-301 ms	9	RPO	0.165	-0.312	0.576	0.499	0.555	0.351	No association
Not-Distracting | PD | EC | 235-301 ms	9	CF	0.285	-0.195	0.654	0.237	0.555	0.544	No association
Not-Distracting | PD | EC | 235-301 ms	9	CFP	0.190	-0.290	0.593	0.437	0.555	0.376	No association
Not-Distracting | PD | EC | 235-301 ms	9	CPO	0.145	-0.331	0.562	0.555	0.555	0.334	No association
Not-Distracting | HC | EO | 235-301 ms	9	LF	-0.278	-0.634	0.175	0.222	0.252	0.544	No association
Not-Distracting | HC | EO | 235-301 ms	9	RF	-0.434	-0.729	-0.003	0.049	0.193	1.649	Nominal p < .05 only
Not-Distracting | HC | EO | 235-301 ms	9	LC	-0.323	-0.663	0.126	0.153	0.229	0.705	No association
Not-Distracting | HC | EO | 235-301 ms	9	RC	-0.398	-0.708	0.040	0.074	0.193	1.211	No association
Not-Distracting | HC | EO | 235-301 ms	9	LPO	-0.213	-0.591	0.240	0.353	0.353	0.405	No association
Not-Distracting | HC | EO | 235-301 ms	9	RPO	-0.364	-0.688	0.080	0.105	0.193	0.928	No association
Not-Distracting | HC | EO | 235-301 ms	9	CF	-0.362	-0.686	0.083	0.107	0.193	0.912	No association
Not-Distracting | HC | EO | 235-301 ms	9	CFP	-0.385	-0.701	0.055	0.084	0.193	1.092	No association
Not-Distracting | HC | EO | 235-301 ms	9	CPO	-0.277	-0.633	0.176	0.224	0.252	0.541	No association
Not-Distracting | PD | EO | 235-301 ms	9	LF	0.260	-0.220	0.639	0.283	0.443	0.486	No association
Not-Distracting | PD | EO | 235-301 ms	9	RF	0.329	-0.147	0.681	0.169	0.443	0.686	No association
Not-Distracting | PD | EO | 235-301 ms	9	LC	0.266	-0.214	0.642	0.271	0.443	0.499	No association
Not-Distracting | PD | EO | 235-301 ms	9	RC	0.272	-0.207	0.647	0.259	0.443	0.514	No association
Not-Distracting | PD | EO | 235-301 ms	9	LPO	0.177	-0.302	0.584	0.469	0.598	0.362	No association
Not-Distracting | PD | EO | 235-301 ms	9	RPO	0.127	-0.347	0.550	0.604	0.604	0.322	No association

FDR_family	FDR_ family_size	Region	*r*	CI_95_ lower	CI_95_ upper	*p*	*q*_FDR	BF_{10}	Evidence_level
Not-Distracting | PD | EO | 235-301 ms	9	CF	0.361	-0.112	0.700	0.129	0.443	0.830	No association
Not-Distracting | PD | EO | 235-301 ms	9	CFP	0.254	-0.227	0.635	0.295	0.443	0.473	No association
Not-Distracting | PD | EO | 235-301 ms	9	CPO	0.153	-0.323	0.568	0.531	0.598	0.341	No association
Not-Worrying | HC | EC | 235-301 ms	9	LF	0.153	-0.298	0.548	0.508	0.931	0.332	No association
Not-Worrying | HC | EC | 235-301 ms	9	RF	0.117	-0.331	0.523	0.612	0.931	0.305	No association
Not-Worrying | HC | EC | 235-301 ms	9	LC	-0.026	-0.452	0.411	0.912	0.931	0.272	No association
Not-Worrying | HC | EC | 235-301 ms	9	RC	-0.020	-0.448	0.415	0.931	0.931	0.271	No association
Not-Worrying | HC | EC | 235-301 ms	9	LPO	-0.024	-0.451	0.412	0.917	0.931	0.272	No association
Not-Worrying | HC | EC | 235-301 ms	9	RPO	0.096	-0.350	0.507	0.680	0.931	0.293	No association
Not-Worrying | HC | EC | 235-301 ms	9	CF	0.071	-0.372	0.488	0.760	0.931	0.282	No association
Not-Worrying | HC | EC | 235-301 ms	9	CFP	0.144	-0.307	0.542	0.535	0.931	0.324	No association
Not-Worrying | HC | EC | 235-301 ms	9	CPO	0.133	-0.317	0.534	0.567	0.931	0.315	No association
Not-Worrying | PD | EC | 235-301 ms	9	LF	0.532	0.103	0.794	0.019	0.046	3.666	FDR-corrected q < .05
Not-Worrying | PD | EC | 235-301 ms	9	RF	0.550	0.127	0.803	0.015	0.046	4.505	FDR-corrected q < .05
Not-Worrying | PD | EC | 235-301 ms	9	LC	0.458	0.004	0.755	0.049	0.049	1.729	FDR-corrected q < .05
Not-Worrying | PD | EC | 235-301 ms	9	RC	0.473	0.024	0.763	0.041	0.046	1.996	FDR-corrected q < .05
Not-Worrying | PD | EC | 235-301 ms	9	LPO	0.505	0.066	0.780	0.027	0.046	2.733	FDR-corrected q < .05
Not-Worrying | PD | EC | 235-301 ms	9	RPO	0.560	0.142	0.809	0.013	0.046	5.136	FDR-corrected q < .05
Not-Worrying | PD | EC | 235-301 ms	9	CF	0.511	0.073	0.783	0.025	0.046	2.894	FDR-corrected q < .05
Not-Worrying | PD | EC | 235-301 ms	9	CFP	0.495	0.053	0.775	0.031	0.046	2.469	FDR-corrected q < .05
Not-Worrying | PD | EC | 235-301 ms	9	CPO	0.474	0.025	0.764	0.040	0.046	2.006	FDR-corrected q < .05
Not-Worrying | HC | EO | 235-301 ms	9	LF	-0.083	-0.497	0.362	0.722	0.995	0.287	No association
Not-Worrying | HC | EO | 235-301 ms	9	RF	-0.032	-0.458	0.405	0.889	0.995	0.273	No association
Not-Worrying | HC | EO | 235-301 ms	9	LC	-0.095	-0.506	0.351	0.683	0.995	0.292	No association
Not-Worrying | HC | EO | 235-301 ms	9	RC	-0.076	-0.491	0.368	0.744	0.995	0.284	No association
Not-Worrying | HC | EO | 235-301 ms	9	LPO	-0.149	-0.546	0.302	0.518	0.995	0.329	No association
Not-Worrying | HC | EO | 235-301 ms	9	RPO	-0.002	-0.433	0.430	0.995	0.995	0.270	No association
Not-Worrying | HC | EO | 235-301 ms	9	CF	-0.066	-0.484	0.376	0.776	0.995	0.281	No association

FDR_family	FDR_ family_size	Region	*r*	CI_95_ lower	CI_95_ upper	*p*	*q*_FDR	BF_{10}	Evidence_level
Not-Worrying | HC | EO | 235-301 ms	9	CFP	-0.110	-0.517	0.337	0.634	0.995	0.301	No association
Not-Worrying | HC | EO | 235-301 ms	9	CPO	-0.056	-0.476	0.385	0.810	0.995	0.278	No association
Not-Worrying | PD | EO | 235-301 ms	9	LF	0.311	-0.167	0.670	0.196	0.196	0.620	No association
Not-Worrying | PD | EO | 235-301 ms	9	RF	0.401	-0.066	0.723	0.089	0.115	1.090	No association
Not-Worrying | PD | EO | 235-301 ms	9	LC	0.457	0.003	0.755	0.049	0.103	1.719	Nominal $p < .05$ only
Not-Worrying | PD | EO | 235-301 ms	9	RC	0.324	-0.152	0.679	0.175	0.196	0.668	No association
Not-Worrying | PD | EO | 235-301 ms	9	LPO	0.527	0.096	0.792	0.020	0.103	3.477	Nominal $p < .05$ only
Not-Worrying | PD | EO | 235-301 ms	9	RPO	0.444	-0.013	0.747	0.057	0.103	1.536	No association
Not-Worrying | PD | EO | 235-301 ms	9	CF	0.404	-0.061	0.725	0.086	0.115	1.119	No association
Not-Worrying | PD | EO | 235-301 ms	9	CFP	0.443	-0.014	0.747	0.057	0.103	1.529	No association
Not-Worrying | PD | EO | 235-301 ms	9	CPO	0.515	0.079	0.785	0.024	0.103	3.032	Nominal $p < .05$ only
Emotional Awareness | HC | EC | 235-301 ms	9	LF	-0.160	-0.553	0.292	0.488	0.984	0.339	No association
Emotional Awareness | HC | EC | 235-301 ms	9	RF	-0.103	-0.512	0.344	0.657	0.984	0.296	No association
Emotional Awareness | HC | EC | 235-301 ms	9	LC	-0.005	-0.435	0.428	0.984	0.984	0.270	No association
Emotional Awareness | HC | EC | 235-301 ms	9	RC	0.095	-0.351	0.506	0.681	0.984	0.293	No association
Emotional Awareness | HC | EC | 235-301 ms	9	LPO	0.039	-0.399	0.463	0.866	0.984	0.274	No association
Emotional Awareness | HC | EC | 235-301 ms	9	RPO	0.119	-0.330	0.524	0.608	0.984	0.306	No association
Emotional Awareness | HC | EC | 235-301 ms	9	CF	-0.019	-0.447	0.416	0.933	0.984	0.271	No association
Emotional Awareness | HC | EC | 235-301 ms	9	CFP	0.055	-0.386	0.476	0.812	0.984	0.278	No association
Emotional Awareness | HC | EC | 235-301 ms	9	CPO	0.053	-0.388	0.474	0.820	0.984	0.277	No association
Emotional Awareness | PD | EC | 235-301 ms	9	LF	-0.178	-0.585	0.301	0.467	0.956	0.363	No association

FDR_family	FDR_ family_size	Region	*r*	CI_95_ lower	CI_95_ upper	*p*	*q*_FDR	BF_{10}	Evidence_level
Emotional Awareness | PD | EC | 235-301 ms	9	RF	-0.114	-0.540	0.359	0.643	0.956	0.314	No association
Emotional Awareness | PD | EC | 235-301 ms	9	LC	-0.067	-0.506	0.399	0.785	0.956	0.294	No association
Emotional Awareness | PD | EC | 235-301 ms	9	RC	-0.148	-0.564	0.328	0.545	0.956	0.337	No association
Emotional Awareness | PD | EC | 235-301 ms	9	LPO	0.016	-0.442	0.467	0.949	0.956	0.284	No association
Emotional Awareness | PD | EC | 235-301 ms	9	RPO	0.018	-0.440	0.469	0.941	0.956	0.285	No association
Emotional Awareness | PD | EC | 235-301 ms	9	CF	-0.206	-0.603	0.274	0.398	0.956	0.396	No association
Emotional Awareness | PD | EC | 235-301 ms	9	CFP	0.014	-0.443	0.465	0.956	0.956	0.284	No association
Emotional Awareness | PD | EC | 235-301 ms	9	CPO	0.157	-0.320	0.571	0.520	0.956	0.344	No association
Emotional Awareness | HC | EO | 235-301 ms	9	LF	0.198	-0.255	0.580	0.389	0.389	0.383	No association
Emotional Awareness | HC | EO | 235-301 ms	9	RF	0.302	-0.149	0.649	0.183	0.206	0.621	No association
Emotional Awareness | HC | EO | 235-301 ms	9	LC	0.303	-0.148	0.650	0.182	0.206	0.624	No association
Emotional Awareness | HC | EO | 235-301 ms	9	RC	0.414	-0.021	0.718	0.062	0.201	1.385	No association
Emotional Awareness | HC | EO | 235-301 ms	9	LPO	0.376	-0.067	0.695	0.093	0.201	1.012	No association
Emotional Awareness | HC | EO | 235-301 ms	9	RPO	0.358	-0.088	0.684	0.111	0.201	0.887	No association
Emotional Awareness | HC | EO | 235-301 ms	9	CF	0.312	-0.138	0.656	0.168	0.206	0.659	No association
Emotional Awareness | HC | EO | 235-301 ms	9	CFP	0.430	-0.002	0.727	0.052	0.201	1.597	No association
Emotional Awareness | HC | EO | 235-301 ms	9	CPO	0.389	-0.052	0.703	0.082	0.201	1.121	No association

FDR_family	FDR_ family_size	Region	*r*	CI_95_ lower	CI_95_ upper	*p*	*q*_FDR	BF_{10}	Evidence_level
Emotional Awareness | PD | EO | 235-301 ms	9	LF	-0.077	-0.513	0.391	0.753	0.897	0.297	No association
Emotional Awareness | PD | EO | 235-301 ms	9	RF	-0.064	-0.504	0.402	0.794	0.897	0.293	No association
Emotional Awareness | PD | EO | 235-301 ms	9	LC	0.032	-0.429	0.479	0.897	0.897	0.286	No association
Emotional Awareness | PD | EO | 235-301 ms	9	RC	-0.033	-0.480	0.428	0.894	0.897	0.286	No association
Emotional Awareness | PD | EO | 235-301 ms	9	LPO	0.120	-0.354	0.545	0.625	0.897	0.317	No association
Emotional Awareness | PD | EO | 235-301 ms	9	RPO	0.041	-0.421	0.486	0.867	0.897	0.287	No association
Emotional Awareness | PD | EO | 235-301 ms	9	CF	-0.099	-0.529	0.372	0.687	0.897	0.306	No association
Emotional Awareness | PD | EO | 235-301 ms	9	CFP	0.109	-0.363	0.537	0.657	0.897	0.311	No association
Emotional Awareness | PD | EO | 235-301 ms	9	CPO	0.165	-0.313	0.576	0.501	0.897	0.351	No association
Trusting | HC | EC | 235-301 ms	9	LF	0.012	-0.422	0.442	0.958	0.975	0.271	No association
Trusting | HC | EC | 235-301 ms	9	RF	0.028	-0.408	0.455	0.902	0.975	0.272	No association
Trusting | HC | EC | 235-301 ms	9	LC	0.043	-0.396	0.466	0.852	0.975	0.275	No association
Trusting | HC | EC | 235-301 ms	9	RC	0.070	-0.373	0.487	0.762	0.975	0.282	No association
Trusting | HC | EC | 235-301 ms	9	LPO	-0.084	-0.498	0.361	0.717	0.975	0.288	No association
Trusting | HC | EC | 235-301 ms	9	RPO	-0.007	-0.438	0.426	0.975	0.975	0.270	No association
Trusting | HC | EC | 235-301 ms	9	CF	0.168	-0.284	0.559	0.467	0.975	0.346	No association
Trusting | HC | EC | 235-301 ms	9	CFP	0.106	-0.341	0.514	0.646	0.975	0.298	No association
Trusting | HC | EC | 235-301 ms	9	CPO	0.026	-0.410	0.453	0.911	0.975	0.272	No association
Trusting | PD | EC | 235-301 ms	9	LF	-0.020	-0.470	0.438	0.934	0.934	0.285	No association
Trusting | PD | EC | 235-301 ms	9	RF	-0.109	-0.537	0.363	0.656	0.934	0.311	No association
Trusting | PD | EC | 235-301 ms	9	LC	-0.060	-0.501	0.405	0.806	0.934	0.292	No association
Trusting | PD | EC | 235-301 ms	9	RC	-0.078	-0.514	0.390	0.750	0.934	0.298	No association

FDR_family	FDR_ family_size	Region	*r*	CI_95_ lower	CI_95_ upper	*p*	*q*_FDR	BF_{10}	Evidence_level
Trusting | PD | EC | 235-301 ms	9	LPO	-0.163	-0.575	0.315	0.505	0.934	0.349	No association
Trusting | PD | EC | 235-301 ms	9	RPO	-0.020	-0.470	0.438	0.934	0.934	0.285	No association
Trusting | PD | EC | 235-301 ms	9	CF	-0.268	-0.644	0.212	0.268	0.934	0.504	No association
Trusting | PD | EC | 235-301 ms	9	CFP	-0.062	-0.502	0.404	0.801	0.934	0.292	No association
Trusting | PD | EC | 235-301 ms	9	CPO	-0.110	-0.537	0.362	0.653	0.934	0.312	No association
Trusting | HC | EO | 235-301 ms	9	LF	-0.069	-0.487	0.373	0.765	0.959	0.282	No association
Trusting | HC | EO | 235-301 ms	9	RF	-0.059	-0.479	0.382	0.799	0.959	0.279	No association
Trusting | HC | EO | 235-301 ms	9	LC	-0.075	-0.491	0.368	0.746	0.959	0.284	No association
Trusting | HC | EO | 235-301 ms	9	RC	-0.068	-0.485	0.375	0.770	0.959	0.281	No association
Trusting | HC | EO | 235-301 ms	9	LPO	-0.053	-0.474	0.388	0.820	0.959	0.277	No association
Trusting | HC | EO | 235-301 ms	9	RPO	-0.020	-0.448	0.415	0.932	0.959	0.271	No association
Trusting | HC | EO | 235-301 ms	9	CF	-0.012	-0.441	0.422	0.959	0.959	0.271	No association
Trusting | HC | EO | 235-301 ms	9	CFP	-0.103	-0.512	0.344	0.656	0.959	0.297	No association
Trusting | HC | EO | 235-301 ms	9	CPO	0.018	-0.417	0.446	0.938	0.959	0.271	No association
Trusting | PD | EO | 235-301 ms	9	LF	-0.243	-0.628	0.238	0.316	0.949	0.453	No association
Trusting | PD | EO | 235-301 ms	9	RF	-0.300	-0.664	0.179	0.212	0.949	0.587	No association
Trusting | PD | EO | 235-301 ms	9	LC	-0.138	-0.557	0.337	0.574	0.951	0.329	No association
Trusting | PD | EO | 235-301 ms	9	RC	-0.147	-0.563	0.329	0.549	0.951	0.336	No association
Trusting | PD | EO | 235-301 ms	9	LPO	-0.001	-0.455	0.453	0.996	0.996	0.284	No association
Trusting | PD | EO | 235-301 ms	9	RPO	0.059	-0.406	0.500	0.811	0.996	0.291	No association
Trusting | PD | EO | 235-301 ms	9	CF	-0.348	-0.693	0.126	0.144	0.949	0.766	No association
Trusting | PD | EO | 235-301 ms	9	CFP	-0.117	-0.542	0.356	0.634	0.951	0.315	No association
Trusting | PD | EO | 235-301 ms	9	CPO	-0.006	-0.459	0.449	0.981	0.996	0.284	No association
BPQ-VSF | HC | EC | 263-381 ms	9	LF	0.122	-0.327	0.526	0.598	0.709	0.308	No association
BPQ-VSF | HC | EC | 263-381 ms	9	RF	0.087	-0.358	0.500	0.709	0.709	0.289	No association
BPQ-VSF | HC | EC | 263-381 ms	9	LC	0.097	-0.349	0.508	0.674	0.709	0.294	No association
BPQ-VSF | HC | EC | 263-381 ms	9	RC	0.140	-0.311	0.539	0.545	0.709	0.321	No association
BPQ-VSF | HC | EC | 263-381 ms	9	LPO	0.091	-0.355	0.503	0.695	0.709	0.291	No association

FDR_family	FDR_ family_size	Region	*r*	CI_95_ lower	CI_95_ upper	*p*	*q*_FDR	BF_{10}	Evidence_level
BPQ-VSF | HC | EC | 263-381 ms	9	RPO	0.256	-0.197	0.619	0.262	0.709	0.488	No association
BPQ-VSF | HC | EC | 263-381 ms	9	CF	0.151	-0.300	0.547	0.514	0.709	0.330	No association
BPQ-VSF | HC | EC | 263-381 ms	9	CFP	0.117	-0.331	0.522	0.613	0.709	0.305	No association
BPQ-VSF | HC | EC | 263-381 ms	9	CPO	0.099	-0.347	0.509	0.669	0.709	0.295	No association
BPQ-VSF | PD | EC | 263-381 ms	9	LF	-0.199	-0.599	0.281	0.415	0.727	0.387	No association
BPQ-VSF | PD | EC | 263-381 ms	9	RF	-0.238	-0.624	0.243	0.327	0.727	0.444	No association
BPQ-VSF | PD | EC | 263-381 ms	9	LC	-0.243	-0.628	0.238	0.316	0.727	0.453	No association
BPQ-VSF | PD | EC | 263-381 ms	9	RC	-0.141	-0.559	0.335	0.565	0.727	0.331	No association
BPQ-VSF | PD | EC | 263-381 ms	9	LPO	-0.103	-0.532	0.369	0.675	0.733	0.308	No association
BPQ-VSF | PD | EC | 263-381 ms	9	RPO	-0.084	-0.518	0.385	0.733	0.733	0.300	No association
BPQ-VSF | PD | EC | 263-381 ms	9	CF	-0.321	-0.677	0.156	0.180	0.727	0.657	No association
BPQ-VSF | PD | EC | 263-381 ms	9	CFP	-0.304	-0.666	0.174	0.206	0.727	0.599	No association
BPQ-VSF | PD | EC | 263-381 ms	9	CPO	-0.152	-0.567	0.324	0.534	0.727	0.340	No association
BPQ-VSF | HC | EO | 263-381 ms	9	LF	0.175	-0.278	0.564	0.448	0.532	0.354	No association
BPQ-VSF | HC | EO | 263-381 ms	9	RF	0.166	-0.286	0.558	0.473	0.532	0.344	No association
BPQ-VSF | HC | EO | 263-381 ms	9	LC	0.234	-0.220	0.604	0.308	0.462	0.440	No association
BPQ-VSF | HC | EO | 263-381 ms	9	RC	0.300	-0.151	0.648	0.186	0.425	0.613	No association
BPQ-VSF | HC | EO | 263-381 ms	9	LPO	0.139	-0.312	0.538	0.548	0.548	0.320	No association
BPQ-VSF | HC | EO | 263-381 ms	9	RPO	0.286	-0.166	0.639	0.209	0.425	0.567	No association
BPQ-VSF | HC | EO | 263-381 ms	9	CF	0.359	-0.086	0.685	0.110	0.425	0.897	No association
BPQ-VSF | HC | EO | 263-381 ms	9	CFP	0.419	-0.016	0.720	0.059	0.425	1.442	No association
BPQ-VSF | HC | EO | 263-381 ms	9	CPO	0.270	-0.183	0.629	0.236	0.425	0.522	No association
BPQ-VSF | PD | EO | 263-381 ms	9	LF	-0.408	-0.728	0.056	0.083	0.172	1.155	No association
BPQ-VSF | PD | EO | 263-381 ms	9	RF	-0.429	-0.739	0.032	0.067	0.172	1.354	No association
BPQ-VSF | PD | EO | 263-381 ms	9	LC	-0.374	-0.708	0.097	0.115	0.172	0.905	No association
BPQ-VSF | PD | EO | 263-381 ms	9	RC	-0.382	-0.713	0.087	0.107	0.172	0.955	No association
BPQ-VSF | PD | EO | 263-381 ms	9	LPO	-0.266	-0.643	0.214	0.271	0.271	0.500	No association
BPQ-VSF | PD | EO | 263-381 ms	9	RPO	-0.324	-0.679	0.152	0.176	0.198	0.668	No association

FDR_family	FDR_ family_size	Region	*r*	CI_95_ lower	CI_95_ upper	*p*	*q*_FDR	BF_{10}	Evidence_level
BPQ-VSF | PD | EO | 263-381 ms	9	CF	-0.380	-0.711	0.090	0.109	0.172	0.941	No association
BPQ-VSF | PD | EO | 263-381 ms	9	CFP	-0.425	-0.737	0.036	0.070	0.172	1.312	No association
BPQ-VSF | PD | EO | 263-381 ms	9	CPO	-0.331	-0.683	0.145	0.166	0.198	0.694	No association
Not-Distracting | HC | EC | 263-381 ms	9	LF	-0.273	-0.630	0.180	0.232	0.298	0.528	No association
Not-Distracting | HC | EC | 263-381 ms	9	RF	-0.282	-0.636	0.171	0.216	0.298	0.554	No association
Not-Distracting | HC | EC | 263-381 ms	9	LC	-0.322	-0.662	0.127	0.155	0.298	0.699	No association
Not-Distracting | HC | EC | 263-381 ms	9	RC	-0.289	-0.641	0.162	0.203	0.298	0.578	No association
Not-Distracting | HC | EC | 263-381 ms	9	LPO	-0.207	-0.586	0.247	0.368	0.414	0.395	No association
Not-Distracting | HC | EC | 263-381 ms	9	RPO	-0.341	-0.673	0.107	0.131	0.298	0.790	No association
Not-Distracting | HC | EC | 263-381 ms	9	CF	-0.359	-0.685	0.086	0.110	0.298	0.895	No association
Not-Distracting | HC | EC | 263-381 ms	9	CFP	-0.422	-0.722	0.012	0.057	0.298	1.482	No association
Not-Distracting | HC | EC | 263-381 ms	9	CPO	-0.176	-0.565	0.277	0.446	0.446	0.355	No association
Not-Distracting | PD | EC | 263-381 ms	9	LF	0.355	-0.118	0.697	0.135	0.146	0.802	No association
Not-Distracting | PD | EC | 263-381 ms	9	RF	0.400	-0.066	0.723	0.090	0.146	1.084	No association
Not-Distracting | PD | EC | 263-381 ms	9	LC	0.360	-0.113	0.700	0.130	0.146	0.824	No association
Not-Distracting | PD | EC | 263-381 ms	9	RC	0.425	-0.036	0.737	0.070	0.146	1.313	No association
Not-Distracting | PD | EC | 263-381 ms	9	LPO	0.383	-0.087	0.713	0.106	0.146	0.959	No association
Not-Distracting | PD | EC | 263-381 ms	9	RPO	0.350	-0.124	0.694	0.142	0.146	0.775	No association
Not-Distracting | PD | EC | 263-381 ms	9	CF	0.390	-0.078	0.717	0.099	0.146	1.009	No association
Not-Distracting | PD | EC | 263-381 ms	9	CFP	0.347	-0.127	0.692	0.146	0.146	0.761	No association
Not-Distracting | PD | EC | 263-381 ms	9	CPO	0.349	-0.125	0.694	0.143	0.146	0.773	No association
Not-Distracting | HC | EO | 263-381 ms	9	LF	-0.226	-0.600	0.228	0.324	0.382	0.427	No association
Not-Distracting | HC | EO | 263-381 ms	9	RF	-0.307	-0.652	0.143	0.175	0.331	0.640	No association
Not-Distracting | HC | EO | 263-381 ms	9	LC	-0.279	-0.634	0.174	0.221	0.331	0.546	No association
Not-Distracting | HC | EO | 263-381 ms	9	RC	-0.315	-0.657	0.135	0.164	0.331	0.670	No association
Not-Distracting | HC | EO | 263-381 ms	9	LPO	-0.218	-0.594	0.235	0.341	0.382	0.413	No association
Not-Distracting | HC | EO | 263-381 ms	9	RPO	-0.284	-0.638	0.168	0.212	0.331	0.561	No association
Not-Distracting | HC | EO | 263-381 ms	9	CF	-0.391	-0.704	0.049	0.080	0.331	1.139	No association

FDR_family	FDR_ family_size	Region	*r*	CI_95_ lower	CI_95_ upper	*p*	*q*_FDR	BF_{10}	Evidence_level
Not-Distracting | HC | EO | 263-381 ms	9	CFP	-0.379	-0.697	0.062	0.090	0.331	1.042	No association
Not-Distracting | HC | EO | 263-381 ms	9	CPO	-0.201	-0.582	0.252	0.382	0.382	0.387	No association
Not-Distracting | PD | EO | 263-381 ms	9	LF	0.351	-0.123	0.694	0.141	0.187	0.781	No association
Not-Distracting | PD | EO | 263-381 ms	9	RF	0.371	-0.100	0.706	0.118	0.187	0.886	No association
Not-Distracting | PD | EO | 263-381 ms	9	LC	0.373	-0.097	0.708	0.115	0.187	0.901	No association
Not-Distracting | PD | EO | 263-381 ms	9	RC	0.362	-0.110	0.701	0.128	0.187	0.837	No association
Not-Distracting | PD | EO | 263-381 ms	9	LPO	0.347	-0.127	0.692	0.145	0.187	0.763	No association
Not-Distracting | PD | EO | 263-381 ms	9	RPO	0.264	-0.216	0.641	0.275	0.275	0.495	No association
Not-Distracting | PD | EO | 263-381 ms	9	CF	0.394	-0.073	0.720	0.095	0.187	1.042	No association
Not-Distracting | PD | EO | 263-381 ms	9	CFP	0.350	-0.124	0.694	0.142	0.187	0.774	No association
Not-Distracting | PD | EO | 263-381 ms	9	CPO	0.331	-0.145	0.683	0.166	0.187	0.693	No association
Not-Worrying | HC | EC | 263-381 ms	9	LF	0.291	-0.161	0.642	0.200	0.817	0.584	No association
Not-Worrying | HC | EC | 263-381 ms	9	RF	0.185	-0.268	0.571	0.422	0.817	0.366	No association
Not-Worrying | HC | EC | 263-381 ms	9	LC	0.099	-0.347	0.509	0.669	0.860	0.294	No association
Not-Worrying | HC | EC | 263-381 ms	9	RC	0.005	-0.427	0.436	0.982	0.982	0.270	No association
Not-Worrying | HC | EC | 263-381 ms	9	LPO	0.040	-0.399	0.464	0.864	0.972	0.274	No association
Not-Worrying | HC | EC | 263-381 ms	9	RPO	0.165	-0.287	0.557	0.475	0.817	0.343	No association
Not-Worrying | HC | EC | 263-381 ms	9	CF	0.140	-0.310	0.539	0.545	0.817	0.321	No association
Not-Worrying | HC | EC | 263-381 ms	9	CFP	0.194	-0.260	0.577	0.400	0.817	0.377	No association
Not-Worrying | HC | EC | 263-381 ms	9	CPO	0.208	-0.246	0.587	0.366	0.817	0.397	No association
Not-Worrying | PD | EC | 263-381 ms	9	LF	0.458	0.004	0.755	0.049	0.220	1.731	Nominal *p* < .05 only
Not-Worrying | PD | EC | 263-381 ms	9	RF	0.474	0.026	0.764	0.040	0.220	2.016	Nominal *p* < .05 only
Not-Worrying | PD | EC | 263-381 ms	9	LC	0.283	-0.197	0.653	0.241	0.275	0.539	No association
Not-Worrying | PD | EC | 263-381 ms	9	RC	0.370	-0.102	0.705	0.119	0.275	0.880	No association
Not-Worrying | PD | EC | 263-381 ms	9	LPO	0.267	-0.213	0.643	0.269	0.275	0.502	No association
Not-Worrying | PD | EC | 263-381 ms	9	RPO	0.302	-0.176	0.665	0.209	0.275	0.593	No association
Not-Worrying | PD | EC | 263-381 ms	9	CF	0.353	-0.121	0.696	0.138	0.275	0.790	No association
Not-Worrying | PD | EC | 263-381 ms	9	CFP	0.312	-0.166	0.671	0.193	0.275	0.625	No association

FDR_family	FDR_ family_size	Region	*r*	CI_95_ lower	CI_95_ upper	*p*	*q*_FDR	BF_{10}	Evidence_level
Not-Worrying | PD | EC | 263-381 ms	9	CPO	0.264	-0.216	0.641	0.275	0.275	0.495	No association
Not-Worrying | HC | EO | 263-381 ms	9	LF	0.012	-0.422	0.441	0.960	0.977	0.271	No association
Not-Worrying | HC | EO | 263-381 ms	9	RF	0.007	-0.426	0.437	0.977	0.977	0.270	No association
Not-Worrying | HC | EO | 263-381 ms	9	LC	-0.009	-0.439	0.425	0.971	0.977	0.270	No association
Not-Worrying | HC | EO | 263-381 ms	9	RC	-0.027	-0.453	0.410	0.908	0.977	0.272	No association
Not-Worrying | HC | EO | 263-381 ms	9	LPO	-0.154	-0.549	0.297	0.505	0.977	0.333	No association
Not-Worrying | HC | EO | 263-381 ms	9	RPO	0.060	-0.382	0.479	0.797	0.977	0.279	No association
Not-Worrying | HC | EO | 263-381 ms	9	CF	-0.042	-0.465	0.397	0.857	0.977	0.274	No association
Not-Worrying | HC | EO | 263-381 ms	9	CFP	-0.161	-0.554	0.291	0.485	0.977	0.340	No association
Not-Worrying | HC | EO | 263-381 ms	9	CPO	-0.057	-0.477	0.384	0.805	0.977	0.278	No association
Not-Worrying | PD | EO | 263-381 ms	9	LF	0.228	-0.252	0.618	0.348	0.370	0.428	No association
Not-Worrying | PD | EO | 263-381 ms	9	RF	0.308	-0.170	0.669	0.199	0.370	0.612	No association
Not-Worrying | PD | EO | 263-381 ms	9	LC	0.276	-0.204	0.649	0.253	0.370	0.522	No association
Not-Worrying | PD | EO | 263-381 ms	9	RC	0.218	-0.262	0.612	0.370	0.370	0.413	No association
Not-Worrying | PD | EO | 263-381 ms	9	LPO	0.276	-0.204	0.649	0.253	0.370	0.523	No association
Not-Worrying | PD | EO | 263-381 ms	9	RPO	0.245	-0.235	0.629	0.312	0.370	0.457	No association
Not-Worrying | PD | EO | 263-381 ms	9	CF	0.266	-0.214	0.643	0.270	0.370	0.500	No association
Not-Worrying | PD | EO | 263-381 ms	9	CFP	0.276	-0.204	0.649	0.252	0.370	0.523	No association
Not-Worrying | PD | EO | 263-381 ms	9	CPO	0.283	-0.196	0.653	0.240	0.370	0.541	No association
Emotional Awareness | HC | EC | 263-381 ms	9	LF	-0.211	-0.589	0.243	0.358	0.910	0.402	No association
Emotional Awareness | HC | EC | 263-381 ms	9	RF	-0.150	-0.546	0.301	0.517	0.910	0.329	No association
Emotional Awareness | HC | EC | 263-381 ms	9	LC	-0.106	-0.514	0.341	0.646	0.910	0.298	No association
Emotional Awareness | HC | EC | 263-381 ms	9	RC	0.035	-0.403	0.460	0.880	0.910	0.273	No association
Emotional Awareness | HC | EC | 263-381 ms	9	LPO	-0.105	-0.513	0.342	0.651	0.910	0.297	No association

FDR_family	FDR_ family_size	Region	*r*	CI_95_ lower	CI_95_ upper	*p*	*q*_FDR	BF_{10}	Evidence_level
Emotional Awareness | HC | EC | 263-381 ms	9	RPO	-0.026	-0.453	0.410	0.910	0.910	0.272	No association
Emotional Awareness | HC | EC | 263-381 ms	9	CF	-0.048	-0.470	0.392	0.837	0.910	0.276	No association
Emotional Awareness | HC | EC | 263-381 ms	9	CFP	0.041	-0.398	0.464	0.860	0.910	0.274	No association
Emotional Awareness | HC | EC | 263-381 ms	9	CPO	-0.108	-0.516	0.339	0.641	0.910	0.299	No association
Emotional Awareness | PD | EC | 263-381 ms	9	LF	-0.223	-0.615	0.257	0.358	0.941	0.421	No association
Emotional Awareness | PD | EC | 263-381 ms	9	RF	-0.082	-0.517	0.386	0.737	0.941	0.299	No association
Emotional Awareness | PD | EC | 263-381 ms	9	LC	-0.127	-0.550	0.347	0.604	0.941	0.322	No association
Emotional Awareness | PD | EC | 263-381 ms	9	RC	-0.147	-0.563	0.329	0.548	0.941	0.336	No association
Emotional Awareness | PD | EC | 263-381 ms	9	LPO	-0.059	-0.500	0.406	0.809	0.941	0.292	No association
Emotional Awareness | PD | EC | 263-381 ms	9	RPO	-0.049	-0.492	0.414	0.842	0.941	0.289	No association
Emotional Awareness | PD | EC | 263-381 ms	9	CF	-0.161	-0.573	0.317	0.511	0.941	0.347	No association
Emotional Awareness | PD | EC | 263-381 ms	9	CFP	-0.097	-0.528	0.374	0.693	0.941	0.305	No association
Emotional Awareness | PD | EC | 263-381 ms	9	CPO	-0.018	-0.468	0.440	0.941	0.941	0.284	No association
Emotional Awareness | HC | EO | 263-381 ms	9	LF	0.192	-0.261	0.576	0.404	0.450	0.375	No association
Emotional Awareness | HC | EO | 263-381 ms	9	RF	0.253	-0.201	0.617	0.269	0.384	0.479	No association
Emotional Awareness | HC | EO | 263-381 ms	9	LC	0.238	-0.216	0.607	0.299	0.384	0.449	No association
Emotional Awareness | HC | EO | 263-381 ms	9	RC	0.302	-0.149	0.649	0.183	0.384	0.620	No association

FDR_family	FDR_ family_size	Region	*r*	CI_95_ lower	CI_95_ upper	*p*	*q*_FDR	BF_{10}	Evidence_level
Emotional Awareness | HC | EO | 263-381 ms	9	LPO	0.285	-0.168	0.638	0.211	0.384	0.563	No association
Emotional Awareness | HC | EO | 263-381 ms	9	RPO	0.174	-0.279	0.563	0.450	0.450	0.353	No association
Emotional Awareness | HC | EO | 263-381 ms	9	CF	0.320	-0.130	0.660	0.157	0.384	0.690	No association
Emotional Awareness | HC | EO | 263-381 ms	9	CFP	0.455	0.029	0.741	0.038	0.343	2.027	Nominal $p < .05$ only
Emotional Awareness | HC | EO | 263-381 ms	9	CPO	0.260	-0.193	0.622	0.255	0.384	0.497	No association
Emotional Awareness | PD | EO | 263-381 ms	9	LF	-0.067	-0.506	0.399	0.784	0.976	0.294	No association
Emotional Awareness | PD | EO | 263-381 ms	9	RF	0.030	-0.430	0.478	0.902	0.976	0.286	No association
Emotional Awareness | PD | EO | 263-381 ms	9	LC	-0.016	-0.467	0.442	0.949	0.976	0.284	No association
Emotional Awareness | PD | EO | 263-381 ms	9	RC	0.021	-0.437	0.471	0.932	0.976	0.285	No association
Emotional Awareness | PD | EO | 263-381 ms	9	LPO	0.036	-0.425	0.482	0.883	0.976	0.287	No association
Emotional Awareness | PD | EO | 263-381 ms	9	RPO	0.037	-0.424	0.483	0.880	0.976	0.287	No association
Emotional Awareness | PD | EO | 263-381 ms	9	CF	0.007	-0.448	0.460	0.976	0.976	0.284	No association
Emotional Awareness | PD | EO | 263-381 ms	9	CFP	0.061	-0.405	0.501	0.805	0.976	0.292	No association
Emotional Awareness | PD | EO | 263-381 ms	9	CPO	0.073	-0.394	0.510	0.767	0.976	0.296	No association
Trusting | HC | EC | 263-381 ms	9	LF	0.206	-0.248	0.585	0.371	0.418	0.393	No association
Trusting | HC | EC | 263-381 ms	9	RF	0.276	-0.177	0.632	0.226	0.396	0.537	No association
Trusting | HC | EC | 263-381 ms	9	LC	0.255	-0.198	0.619	0.264	0.396	0.485	No association
Trusting | HC | EC | 263-381 ms	9	RC	0.284	-0.168	0.638	0.212	0.396	0.562	No association
Trusting | HC | EC | 263-381 ms	9	LPO	0.151	-0.300	0.547	0.513	0.513	0.330	No association

FDR_family	FDR_ family_size	Region	*r*	CI_95_ lower	CI_95_ upper	*p*	*q*_FDR	BF_{10}	Evidence_level
Trusting | HC | EC | 263-381 ms	9	RPO	0.234	-0.220	0.604	0.308	0.396	0.440	No association
Trusting | HC | EC | 263-381 ms	9	CF	0.334	-0.115	0.669	0.139	0.396	0.753	No association
Trusting | HC | EC | 263-381 ms	9	CFP	0.349	-0.098	0.678	0.121	0.396	0.834	No association
Trusting | HC | EC | 263-381 ms	9	CPO	0.249	-0.205	0.614	0.277	0.396	0.470	No association
Trusting | PD | EC | 263-381 ms	9	LF	-0.070	-0.508	0.397	0.775	0.775	0.295	No association
Trusting | PD | EC | 263-381 ms	9	RF	-0.118	-0.543	0.355	0.629	0.708	0.316	No association
Trusting | PD | EC | 263-381 ms	9	LC	-0.188	-0.591	0.291	0.442	0.568	0.374	No association
Trusting | PD | EC | 263-381 ms	9	RC	-0.208	-0.605	0.272	0.394	0.568	0.398	No association
Trusting | PD | EC | 263-381 ms	9	LPO	-0.243	-0.628	0.237	0.315	0.568	0.454	No association
Trusting | PD | EC | 263-381 ms	9	RPO	-0.251	-0.633	0.230	0.301	0.568	0.468	No association
Trusting | PD | EC | 263-381 ms	9	CF	-0.262	-0.640	0.218	0.278	0.568	0.491	No association
Trusting | PD | EC | 263-381 ms	9	CFP	-0.234	-0.622	0.247	0.335	0.568	0.438	No association
Trusting | PD | EC | 263-381 ms	9	CPO	-0.260	-0.639	0.221	0.283	0.568	0.486	No association
Trusting | HC | EO | 263-381 ms	9	LF	0.119	-0.329	0.524	0.606	0.896	0.306	No association
Trusting | HC | EO | 263-381 ms	9	RF	0.182	-0.271	0.569	0.431	0.896	0.362	No association
Trusting | HC | EO | 263-381 ms	9	LC	0.044	-0.395	0.467	0.850	0.896	0.275	No association
Trusting | HC | EO | 263-381 ms	9	RC	0.030	-0.407	0.456	0.896	0.896	0.272	No association
Trusting | HC | EO | 263-381 ms	9	LPO	0.103	-0.344	0.512	0.658	0.896	0.296	No association
Trusting | HC | EO | 263-381 ms	9	RPO	0.091	-0.354	0.503	0.694	0.896	0.291	No association
Trusting | HC | EO | 263-381 ms	9	CF	0.105	-0.342	0.514	0.650	0.896	0.298	No association
Trusting | HC | EO | 263-381 ms	9	CFP	0.048	-0.392	0.470	0.837	0.896	0.276	No association
Trusting | HC | EO | 263-381 ms	9	CPO	0.146	-0.305	0.543	0.528	0.896	0.326	No association
Trusting | PD | EO | 263-381 ms	9	LF	-0.228	-0.618	0.253	0.349	0.492	0.427	No association
Trusting | PD | EO | 263-381 ms	9	RF	-0.227	-0.617	0.253	0.350	0.492	0.426	No association
Trusting | PD | EO | 263-381 ms	9	LC	-0.236	-0.623	0.245	0.332	0.492	0.441	No association
Trusting | PD | EO | 263-381 ms	9	RC	-0.213	-0.608	0.267	0.382	0.492	0.406	No association
Trusting | PD | EO | 263-381 ms	9	LPO	-0.187	-0.591	0.292	0.444	0.500	0.373	No association
Trusting | PD | EO | 263-381 ms	9	RPO	-0.154	-0.569	0.322	0.528	0.528	0.342	No association

FDR_family	FDR_ family_size	Region	*r*	CI_95_ lower	CI_95_ upper	*p*	*q*_FDR	BF_{10}	Evidence_level
Trusting | PD | EO | 263-381 ms	9	CF	-0.250	-0.632	0.231	0.303	0.492	0.466	No association
Trusting | PD | EO | 263-381 ms	9	CFP	-0.225	-0.616	0.255	0.355	0.492	0.423	No association
Trusting | PD | EO | 263-381 ms	9	CPO	-0.213	-0.608	0.267	0.382	0.492	0.405	No association

Note. Pearson correlations were computed within each group. FDR correction was applied across the nine ROIs within each group, HEP time window, resting-state condition, and self-report construct. *q*_FDR denotes the Benjamini–Hochberg FDR-adjusted *p* value. BF_{10} denotes the Bayes factor in favor of the alternative hypothesis of a non-zero correlation over the null hypothesis. Results with $q < .05$ were considered FDR-corrected associations, and results with $.05 \leqslant q < .10$ were considered exploratory FDR-level patterns.

Supplementary Table S5

Supplementary Table S5A. HEP age-adjusted partial correlation results.

FDR_family	FDR_family_size	Region	partial_*r*_controlling_age	CI_95_lower	CI_95_upper	*p*_partial	*q*_FDR_partial	Evidence_level
BPQ-VSF | HC | EC | 235-301 ms	9	LF	0.346	-0.114	0.684	0.135	0.198	No association
BPQ-VSF | HC | EC | 235-301 ms	9	RF	0.326	-0.136	0.672	0.161	0.198	No association
BPQ-VSF | HC | EC | 235-301 ms	9	LC	0.319	-0.143	0.668	0.170	0.198	No association
BPQ-VSF | HC | EC | 235-301 ms	9	RC	0.246	-0.221	0.621	0.296	0.296	No association
BPQ-VSF | HC | EC | 235-301 ms	9	LPO	0.333	-0.128	0.676	0.151	0.198	No association
BPQ-VSF | HC | EC | 235-301 ms	9	RPO	0.439	-0.004	0.738	0.053	0.198	No association
BPQ-VSF | HC | EC | 235-301 ms	9	CF	0.350	-0.109	0.686	0.130	0.198	No association
BPQ-VSF | HC | EC | 235-301 ms	9	CFP	0.347	-0.112	0.685	0.134	0.198	No association
BPQ-VSF | HC | EC | 235-301 ms	9	CPO	0.315	-0.148	0.665	0.176	0.198	No association
BPQ-VSF | PD | EC | 235-301 ms	9	LF	-0.257	-0.647	0.238	0.303	0.475	No association
BPQ-VSF | PD | EC | 235-301 ms	9	RF	-0.280	-0.660	0.215	0.261	0.475	No association
BPQ-VSF | PD | EC | 235-301 ms	9	LC	-0.333	-0.692	0.159	0.177	0.475	No association
BPQ-VSF | PD | EC | 235-301 ms	9	RC	-0.143	-0.572	0.347	0.572	0.643	No association
BPQ-VSF | PD | EC | 235-301 ms	9	LPO	-0.225	-0.626	0.270	0.369	0.475	No association
BPQ-VSF | PD | EC | 235-301 ms	9	RPO	-0.047	-0.503	0.429	0.852	0.852	No association
BPQ-VSF | PD | EC | 235-301 ms	9	CF	-0.367	-0.712	0.120	0.134	0.475	No association
BPQ-VSF | PD | EC | 235-301 ms	9	CFP	-0.419	-0.741	0.059	0.083	0.475	No association
BPQ-VSF | PD | EC | 235-301 ms	9	CPO	-0.227	-0.628	0.268	0.364	0.475	No association
BPQ-VSF | HC | EO | 235-301 ms	9	LF	0.430	-0.015	0.733	0.058	0.076	Exploratory FDR-level .05 ≤ *q* < .10
BPQ-VSF | HC | EO | 235-301 ms	9	RF	0.473	0.038	0.757	0.035	0.076	Exploratory FDR-level .05 ≤ *q* < .10
BPQ-VSF | HC | EO | 235-301 ms	9	LC	0.429	-0.017	0.732	0.059	0.076	Exploratory FDR-level .05 ≤ *q* < .10

FDR_family	FDR_family_size	Region	partial_*r*_controlling_age	CI_95_lower	CI_95_upper	*p*_partial	*q*_FDR_partial	Evidence_level
BPQ-VSF | HC | EO | 235-301 ms	9	RC	0.432	-0.012	0.734	0.057	0.076	Exploratory FDR-level .05 ≤ *q* < .10
BPQ-VSF | HC | EO | 235-301 ms	9	LPO	0.341	-0.119	0.681	0.141	0.141	No association
BPQ-VSF | HC | EO | 235-301 ms	9	RPO	0.470	0.034	0.755	0.037	0.076	Exploratory FDR-level .05 ≤ *q* < .10
BPQ-VSF | HC | EO | 235-301 ms	9	CF	0.471	0.036	0.756	0.036	0.076	Exploratory FDR-level .05 ≤ *q* < .10
BPQ-VSF | HC | EO | 235-301 ms	9	CFP	0.496	0.069	0.770	0.026	0.076	Exploratory FDR-level .05 ≤ *q* < .10
BPQ-VSF | HC | EO | 235-301 ms	9	CPO	0.410	-0.040	0.721	0.073	0.082	Exploratory FDR-level .05 ≤ *q* < .10
BPQ-VSF | PD | EO | 235-301 ms	9	LF	-0.438	-0.751	0.036	0.069	0.132	No association
BPQ-VSF | PD | EO | 235-301 ms	9	RF	-0.432	-0.748	0.043	0.073	0.132	No association
BPQ-VSF | PD | EO | 235-301 ms	9	LC	-0.454	-0.760	0.017	0.059	0.132	No association
BPQ-VSF | PD | EO | 235-301 ms	9	RC	-0.364	-0.710	0.124	0.138	0.155	No association
BPQ-VSF | PD | EO | 235-301 ms	9	LPO	-0.397	-0.729	0.085	0.102	0.132	No association
BPQ-VSF | PD | EO | 235-301 ms	9	RPO	-0.323	-0.686	0.169	0.191	0.191	No association
BPQ-VSF | PD | EO | 235-301 ms	9	CF	-0.398	-0.729	0.085	0.102	0.132	No association
BPQ-VSF | PD | EO | 235-301 ms	9	CFP	-0.491	-0.779	-0.031	0.039	0.132	Nominal *p* < .05 only
BPQ-VSF | PD | EO | 235-301 ms	9	CPO	-0.413	-0.738	0.067	0.088	0.132	No association
Not-Distracting | HC | EC | 235-301 ms	9	LF	-0.221	-0.605	0.245	0.348	0.420	No association
Not-Distracting | HC | EC | 235-301 ms	9	RF	-0.272	-0.638	0.193	0.245	0.420	No association
Not-Distracting | HC | EC | 235-301 ms	9	LC	-0.257	-0.628	0.210	0.275	0.420	No association
Not-Distracting | HC | EC | 235-301 ms	9	RC	-0.233	-0.612	0.234	0.323	0.420	No association
Not-Distracting | HC | EC | 235-301 ms	9	LPO	-0.155	-0.559	0.309	0.514	0.514	No association
Not-Distracting | HC | EC | 235-301 ms	9	RPO	-0.353	-0.688	0.106	0.127	0.420	No association

FDR_family	FDR_family_size	Region	partial_*r*_controlling_age	CI_95_lower	CI_95_upper	*p*_partial	*q*_FDR_partial	Evidence_level
Not-Distracting | HC | EC | 235-301 ms	9	CF	-0.252	-0.625	0.214	0.283	0.420	No association
Not-Distracting | HC | EC | 235-301 ms	9	CFP	-0.355	-0.689	0.104	0.124	0.420	No association
Not-Distracting | HC | EC | 235-301 ms	9	CPO	-0.210	-0.597	0.256	0.374	0.420	No association
Not-Distracting | PD | EC | 235-301 ms	9	LF	0.011	-0.458	0.475	0.966	0.993	No association
Not-Distracting | PD | EC | 235-301 ms	9	RF	0.058	-0.420	0.511	0.820	0.993	No association
Not-Distracting | PD | EC | 235-301 ms	9	LC	0.017	-0.453	0.480	0.945	0.993	No association
Not-Distracting | PD | EC | 235-301 ms	9	RC	0.053	-0.424	0.508	0.834	0.993	No association
Not-Distracting | PD | EC | 235-301 ms	9	LPO	-0.038	-0.496	0.436	0.880	0.993	No association
Not-Distracting | PD | EC | 235-301 ms	9	RPO	-0.021	-0.483	0.450	0.935	0.993	No association
Not-Distracting | PD | EC | 235-301 ms	9	CF	0.002	-0.465	0.469	0.993	0.993	No association
Not-Distracting | PD | EC | 235-301 ms	9	CFP	-0.004	-0.470	0.464	0.989	0.993	No association
Not-Distracting | PD | EC | 235-301 ms	9	CPO	-0.077	-0.525	0.404	0.762	0.993	No association
Not-Distracting | HC | EO | 235-301 ms	9	LF	-0.279	-0.642	0.186	0.233	0.269	No association
Not-Distracting | HC | EO | 235-301 ms	9	RF	-0.429	-0.733	0.016	0.059	0.235	No association
Not-Distracting | HC | EO | 235-301 ms	9	LC	-0.328	-0.673	0.134	0.158	0.237	No association
Not-Distracting | HC | EO | 235-301 ms	9	RC	-0.388	-0.709	0.066	0.091	0.235	No association
Not-Distracting | HC | EO | 235-301 ms	9	LPO	-0.215	-0.600	0.252	0.363	0.363	No association

FDR_family	FDR_family_size	Region	partial_*r*_controlling_age	CI_95_lower	CI_95_upper	*p*_partial	*q*_FDR_partial	Evidence_level
Not-Distracting | HC | EO | 235-301 ms	9	RPO	-0.373	-0.700	0.083	0.105	0.235	No association
Not-Distracting | HC | EO | 235-301 ms	9	CF	-0.350	-0.686	0.110	0.130	0.235	No association
Not-Distracting | HC | EO | 235-301 ms	9	CFP	-0.379	-0.703	0.077	0.100	0.235	No association
Not-Distracting | HC | EO | 235-301 ms	9	CPO	-0.276	-0.640	0.190	0.239	0.269	No association
Not-Distracting | PD | EO | 235-301 ms	9	LF	0.091	-0.392	0.535	0.719	0.914	No association
Not-Distracting | PD | EO | 235-301 ms	9	RF	0.070	-0.411	0.520	0.783	0.914	No association
Not-Distracting | PD | EO | 235-301 ms	9	LC	0.035	-0.439	0.494	0.891	0.914	No association
Not-Distracting | PD | EO | 235-301 ms	9	RC	0.065	-0.414	0.517	0.797	0.914	No association
Not-Distracting | PD | EO | 235-301 ms	9	LPO	-0.148	-0.575	0.343	0.558	0.914	No association
Not-Distracting | PD | EO | 235-301 ms	9	RPO	-0.047	-0.503	0.429	0.852	0.914	No association
Not-Distracting | PD | EO | 235-301 ms	9	CF	0.097	-0.387	0.540	0.701	0.914	No association
Not-Distracting | PD | EO | 235-301 ms	9	CFP	0.027	-0.445	0.488	0.914	0.914	No association
Not-Distracting | PD | EO | 235-301 ms	9	CPO	-0.130	-0.563	0.359	0.607	0.914	No association
Not-Worrying | HC | EC | 235-301 ms	9	LF	0.155	-0.309	0.559	0.514	0.931	No association
Not-Worrying | HC | EC | 235-301 ms	9	RF	0.119	-0.341	0.534	0.617	0.931	No association
Not-Worrying | HC | EC | 235-301 ms	9	LC	-0.026	-0.464	0.421	0.912	0.931	No association
Not-Worrying | HC | EC | 235-301 ms	9	RC	-0.021	-0.459	0.426	0.931	0.931	No association

FDR_family	FDR_family_size	Region	partial_*r*_controlling_age	CI_95_lower	CI_95_upper	*p*_partial	*q*_FDR_partial	Evidence_level
Not-Worrying | HC | EC | 235-301 ms	9	LPO	-0.025	-0.463	0.422	0.915	0.931	No association
Not-Worrying | HC | EC | 235-301 ms	9	RPO	0.099	-0.359	0.519	0.679	0.931	No association
Not-Worrying | HC | EC | 235-301 ms	9	CF	0.072	-0.383	0.498	0.764	0.931	No association
Not-Worrying | HC | EC | 235-301 ms	9	CFP	0.144	-0.319	0.552	0.544	0.931	No association
Not-Worrying | HC | EC | 235-301 ms	9	CPO	0.136	-0.326	0.546	0.568	0.931	No association
Not-Worrying | PD | EC | 235-301 ms	9	LF	0.492	0.033	0.780	0.038	0.108	Nominal $p < .05$ only
Not-Worrying | PD | EC | 235-301 ms	9	RF	0.489	0.028	0.778	0.040	0.108	Nominal $p < .05$ only
Not-Worrying | PD | EC | 235-301 ms	9	LC	0.391	-0.093	0.726	0.108	0.108	No association
Not-Worrying | PD | EC | 235-301 ms	9	RC	0.403	-0.078	0.732	0.097	0.108	No association
Not-Worrying | PD | EC | 235-301 ms	9	LPO	0.413	-0.066	0.738	0.088	0.108	No association
Not-Worrying | PD | EC | 235-301 ms	9	RPO	0.510	0.056	0.789	0.031	0.108	Nominal $p < .05$ only
Not-Worrying | PD | EC | 235-301 ms	9	CF	0.416	-0.064	0.739	0.086	0.108	No association
Not-Worrying | PD | EC | 235-301 ms	9	CFP	0.432	-0.044	0.748	0.074	0.108	No association
Not-Worrying | PD | EC | 235-301 ms	9	CPO	0.403	-0.078	0.732	0.097	0.108	No association
Not-Worrying | HC | EO | 235-301 ms	9	LF	-0.083	-0.507	0.374	0.729	0.995	No association
Not-Worrying | HC | EO | 235-301 ms	9	RF	-0.033	-0.468	0.416	0.892	0.995	No association
Not-Worrying | HC | EO | 235-301 ms	9	LC	-0.095	-0.516	0.363	0.691	0.995	No association

FDR_family	FDR_family_size	Region	partial_*r*_controlling_age	CI_95_lower	CI_95_upper	*p*_partial	*q*_FDR_partial	Evidence_level
Not-Worrying | HC | EO | 235-301 ms	9	RC	-0.076	-0.502	0.379	0.749	0.995	No association
Not-Worrying | HC | EO | 235-301 ms	9	LPO	-0.149	-0.555	0.314	0.530	0.995	No association
Not-Worrying | HC | EO | 235-301 ms	9	RPO	-0.002	-0.444	0.441	0.995	0.995	No association
Not-Worrying | HC | EO | 235-301 ms	9	CF	-0.067	-0.495	0.387	0.780	0.995	No association
Not-Worrying | HC | EO | 235-301 ms	9	CFP	-0.111	-0.527	0.349	0.642	0.995	No association
Not-Worrying | HC | EO | 235-301 ms	9	CPO	-0.056	-0.486	0.396	0.815	0.995	No association
Not-Worrying | PD | EO | 235-301 ms	9	LF	0.223	-0.272	0.625	0.373	0.376	No association
Not-Worrying | PD | EO | 235-301 ms	9	RF	0.284	-0.211	0.663	0.253	0.329	No association
Not-Worrying | PD | EO | 235-301 ms	9	LC	0.368	-0.120	0.712	0.133	0.267	No association
Not-Worrying | PD | EO | 235-301 ms	9	RC	0.222	-0.273	0.624	0.376	0.376	No association
Not-Worrying | PD | EO | 235-301 ms	9	LPO	0.437	-0.038	0.751	0.070	0.267	No association
Not-Worrying | PD | EO | 235-301 ms	9	RPO	0.387	-0.097	0.723	0.112	0.267	No association
Not-Worrying | PD | EO | 235-301 ms	9	CF	0.283	-0.212	0.662	0.256	0.329	No association
Not-Worrying | PD | EO | 235-301 ms	9	CFP	0.355	-0.134	0.705	0.148	0.267	No association
Not-Worrying | PD | EO | 235-301 ms	9	CPO	0.433	-0.042	0.749	0.073	0.267	No association
Emotional Awareness | HC | EC | 235-301 ms	9	LF	-0.203	-0.592	0.263	0.390	0.983	No association
Emotional Awareness | HC | EC | 235-301 ms	9	RF	-0.145	-0.552	0.318	0.542	0.983	No association

FDR_family	FDR_family_size	Region	partial_*r*_controlling_age	CI_95_lower	CI_95_upper	*p*_partial	*q*_FDR_partial	Evidence_level
Emotional Awareness | HC | EC | 235-301 ms	9	LC	-0.061	-0.491	0.392	0.797	0.983	No association
Emotional Awareness | HC | EC | 235-301 ms	9	RC	0.041	-0.409	0.475	0.863	0.983	No association
Emotional Awareness | HC | EC | 235-301 ms	9	LPO	-0.027	-0.464	0.421	0.910	0.983	No association
Emotional Awareness | HC | EC | 235-301 ms	9	RPO	0.070	-0.385	0.497	0.770	0.983	No association
Emotional Awareness | HC | EC | 235-301 ms	9	CF	-0.053	-0.484	0.399	0.824	0.983	No association
Emotional Awareness | HC | EC | 235-301 ms	9	CFP	0.035	-0.414	0.470	0.884	0.983	No association
Emotional Awareness | HC | EC | 235-301 ms	9	CPO	0.005	-0.438	0.447	0.983	0.983	No association
Emotional Awareness | PD | EC | 235-301 ms	9	LF	-0.103	-0.544	0.382	0.685	0.988	No association
Emotional Awareness | PD | EC | 235-301 ms	9	RF	0.003	-0.465	0.469	0.992	0.992	No association
Emotional Awareness | PD | EC | 235-301 ms	9	LC	0.044	-0.432	0.501	0.862	0.988	No association
Emotional Awareness | PD | EC | 235-301 ms	9	RC	-0.039	-0.497	0.436	0.878	0.988	No association
Emotional Awareness | PD | EC | 235-301 ms	9	LPO	0.200	-0.294	0.610	0.425	0.988	No association
Emotional Awareness | PD | EC | 235-301 ms	9	RPO	0.135	-0.354	0.566	0.593	0.988	No association
Emotional Awareness | PD | EC | 235-301 ms	9	CF	-0.054	-0.508	0.423	0.831	0.988	No association
Emotional Awareness | PD | EC | 235-301 ms	9	CFP	0.138	-0.352	0.568	0.585	0.988	No association
Emotional Awareness | PD | EC | 235-301 ms	9	CPO	0.307	-0.187	0.677	0.215	0.988	No association
Emotional Awareness | HC | EO | 235-301 ms	9	LF	0.201	-0.265	0.591	0.396	0.396	No association

FDR_family	FDR_family_size	Region	partial_*r*_controlling_age	CI_95_lower	CI_95_upper	*p*_partial	*q*_FDR_partial	Evidence_level
Emotional Awareness | HC | EO | 235-301 ms	9	RF	0.295	-0.170	0.652	0.207	0.237	No association
Emotional Awareness | HC | EO | 235-301 ms	9	LC	0.313	-0.150	0.664	0.179	0.237	No association
Emotional Awareness | HC | EO | 235-301 ms	9	RC	0.398	-0.054	0.715	0.082	0.185	No association
Emotional Awareness | HC | EO | 235-301 ms	9	LPO	0.384	-0.071	0.706	0.095	0.185	No association
Emotional Awareness | HC | EO | 235-301 ms	9	RPO	0.375	-0.081	0.701	0.103	0.185	No association
Emotional Awareness | HC | EO | 235-301 ms	9	CF	0.293	-0.172	0.651	0.211	0.237	No association
Emotional Awareness | HC | EO | 235-301 ms	9	CFP	0.423	-0.024	0.729	0.063	0.185	No association
Emotional Awareness | HC | EO | 235-301 ms	9	CPO	0.392	-0.061	0.711	0.087	0.185	No association
Emotional Awareness | PD | EO | 235-301 ms	9	LF	0.035	-0.439	0.494	0.889	0.889	No association
Emotional Awareness | PD | EO | 235-301 ms	9	RF	0.114	-0.373	0.551	0.654	0.841	No association
Emotional Awareness | PD | EO | 235-301 ms	9	LC	0.195	-0.299	0.607	0.438	0.841	No association
Emotional Awareness | PD | EO | 235-301 ms	9	RC	0.108	-0.378	0.547	0.669	0.841	No association
Emotional Awareness | PD | EO | 235-301 ms	9	LPO	0.334	-0.157	0.693	0.175	0.788	No association
Emotional Awareness | PD | EO | 235-301 ms	9	RPO	0.148	-0.343	0.575	0.559	0.841	No association
Emotional Awareness | PD | EO | 235-301 ms	9	CF	0.082	-0.400	0.528	0.747	0.841	No association
Emotional Awareness | PD | EO | 235-301 ms	9	CFP	0.278	-0.217	0.659	0.264	0.791	No association
Emotional Awareness | PD | EO | 235-301 ms	9	CPO	0.355	-0.134	0.705	0.149	0.788	No association

FDR_family	FDR_family_size	Region	partial_*r*_controlling_age	CI_95_lower	CI_95_upper	*p*_partial	*q*_FDR_partial	Evidence_level
Trusting | HC | EC | 235-301 ms	9	LF	0.104	-0.355	0.522	0.664	0.747	No association
Trusting | HC | EC | 235-301 ms	9	RF	0.124	-0.337	0.537	0.601	0.747	No association
Trusting | HC | EC | 235-301 ms	9	LC	0.187	-0.279	0.581	0.430	0.747	No association
Trusting | HC | EC | 235-301 ms	9	RC	0.227	-0.239	0.608	0.336	0.747	No association
Trusting | HC | EC | 235-301 ms	9	LPO	0.066	-0.387	0.494	0.781	0.781	No association
Trusting | HC | EC | 235-301 ms	9	RPO	0.126	-0.335	0.539	0.596	0.747	No association
Trusting | HC | EC | 235-301 ms	9	CF	0.272	-0.194	0.638	0.246	0.747	No association
Trusting | HC | EC | 235-301 ms	9	CFP	0.173	-0.291	0.572	0.465	0.747	No association
Trusting | HC | EC | 235-301 ms	9	CPO	0.152	-0.312	0.557	0.524	0.747	No association
Trusting | PD | EC | 235-301 ms	9	LF	0.122	-0.365	0.557	0.628	0.862	No association
Trusting | PD | EC | 235-301 ms	9	RF	0.073	-0.408	0.522	0.773	0.862	No association
Trusting | PD | EC | 235-301 ms	9	LC	0.116	-0.371	0.553	0.647	0.862	No association
Trusting | PD | EC | 235-301 ms	9	RC	0.107	-0.379	0.547	0.672	0.862	No association
Trusting | PD | EC | 235-301 ms	9	LPO	0.078	-0.404	0.526	0.758	0.862	No association
Trusting | PD | EC | 235-301 ms	9	RPO	0.157	-0.334	0.581	0.533	0.862	No association
Trusting | PD | EC | 235-301 ms	9	CF	-0.044	-0.501	0.432	0.862	0.862	No association
Trusting | PD | EC | 235-301 ms	9	CFP	0.119	-0.369	0.555	0.640	0.862	No association
Trusting | PD | EC | 235-301 ms	9	CPO	0.070	-0.411	0.520	0.783	0.862	No association
Trusting | HC | EO | 235-301 ms	9	LF	-0.073	-0.499	0.382	0.759	0.982	No association
Trusting | HC | EO | 235-301 ms	9	RF	-0.029	-0.466	0.419	0.902	0.982	No association
Trusting | HC | EO | 235-301 ms	9	LC	-0.092	-0.514	0.365	0.700	0.982	No association
Trusting | HC | EO | 235-301 ms	9	RC	-0.006	-0.447	0.438	0.982	0.982	No association
Trusting | HC | EO | 235-301 ms	9	LPO	-0.058	-0.488	0.394	0.807	0.982	No association
Trusting | HC | EO | 235-301 ms	9	RPO	-0.044	-0.477	0.406	0.854	0.982	No association
Trusting | HC | EO | 235-301 ms	9	CF	0.059	-0.394	0.489	0.804	0.982	No association
Trusting | HC | EO | 235-301 ms	9	CFP	-0.071	-0.498	0.383	0.765	0.982	No association
Trusting | HC | EO | 235-301 ms	9	CPO	0.035	-0.414	0.470	0.885	0.982	No association
Trusting | PD | EO | 235-301 ms	9	LF	-0.103	-0.544	0.382	0.684	0.849	No association

FDR_family	FDR_family_size	Region	partial_*r*_controlling_age	CI_95_lower	CI_95_upper	*p*_partial	*q*_FDR_partial	Evidence_level
Trusting | PD | EO | 235-301 ms	9	RF	-0.089	-0.534	0.394	0.725	0.849	No association
Trusting | PD | EO | 235-301 ms	9	LC	0.077	-0.404	0.525	0.761	0.849	No association
Trusting | PD | EO | 235-301 ms	9	RC	0.048	-0.428	0.504	0.849	0.849	No association
Trusting | PD | EO | 235-301 ms	9	LPO	0.301	-0.193	0.673	0.225	0.849	No association
Trusting | PD | EO | 235-301 ms	9	RPO	0.233	-0.262	0.631	0.352	0.849	No association
Trusting | PD | EO | 235-301 ms	9	CF	-0.139	-0.569	0.350	0.581	0.849	No association
Trusting | PD | EO | 235-301 ms	9	CFP	0.096	-0.389	0.538	0.706	0.849	No association
Trusting | PD | EO | 235-301 ms	9	CPO	0.251	-0.244	0.643	0.314	0.849	No association
BPQ-VSF | HC | EC | 263-381 ms	9	LF	0.093	-0.365	0.514	0.698	0.770	No association
BPQ-VSF | HC | EC | 263-381 ms	9	RF	0.092	-0.366	0.513	0.701	0.770	No association
BPQ-VSF | HC | EC | 263-381 ms	9	LC	0.075	-0.380	0.501	0.754	0.770	No association
BPQ-VSF | HC | EC | 263-381 ms	9	RC	0.123	-0.338	0.536	0.606	0.770	No association
BPQ-VSF | HC | EC | 263-381 ms	9	LPO	0.070	-0.385	0.497	0.770	0.770	No association
BPQ-VSF | HC | EC | 263-381 ms	9	RPO	0.235	-0.232	0.614	0.319	0.770	No association
BPQ-VSF | HC | EC | 263-381 ms	9	CF	0.128	-0.334	0.540	0.591	0.770	No association
BPQ-VSF | HC | EC | 263-381 ms	9	CFP	0.132	-0.330	0.542	0.580	0.770	No association
BPQ-VSF | HC | EC | 263-381 ms	9	CPO	0.113	-0.347	0.529	0.635	0.770	No association
BPQ-VSF | PD | EC | 263-381 ms	9	LF	-0.149	-0.576	0.342	0.555	0.940	No association
BPQ-VSF | PD | EC | 263-381 ms	9	RF	-0.188	-0.602	0.306	0.455	0.940	No association
BPQ-VSF | PD | EC | 263-381 ms	9	LC	-0.195	-0.607	0.299	0.438	0.940	No association
BPQ-VSF | PD | EC | 263-381 ms	9	RC	-0.075	-0.524	0.406	0.767	0.940	No association
BPQ-VSF | PD | EC | 263-381 ms	9	LPO	-0.032	-0.492	0.441	0.899	0.940	No association
BPQ-VSF | PD | EC | 263-381 ms	9	RPO	-0.019	-0.482	0.452	0.940	0.940	No association
BPQ-VSF | PD | EC | 263-381 ms	9	CF	-0.281	-0.661	0.214	0.259	0.940	No association
BPQ-VSF | PD | EC | 263-381 ms	9	CFP	-0.261	-0.649	0.234	0.296	0.940	No association
BPQ-VSF | PD | EC | 263-381 ms	9	CPO	-0.093	-0.536	0.391	0.715	0.940	No association
BPQ-VSF | HC | EO | 263-381 ms	9	LF	0.219	-0.248	0.603	0.354	0.354	No association
BPQ-VSF | HC | EO | 263-381 ms	9	RF	0.235	-0.232	0.613	0.320	0.354	No association

FDR_family	FDR_family_size	Region	partial_*r*_controlling_age	CI_95_lower	CI_95_upper	*p*_partial	*q*_FDR_partial	Evidence_level
BPQ-VSF | HC | EO | 263-381 ms	9	LC	0.338	-0.123	0.679	0.145	0.217	No association
BPQ-VSF | HC | EO | 263-381 ms	9	RC	0.369	-0.088	0.698	0.109	0.217	No association
BPQ-VSF | HC | EO | 263-381 ms	9	LPO	0.231	-0.236	0.611	0.327	0.354	No association
BPQ-VSF | HC | EO | 263-381 ms	9	RPO	0.377	-0.079	0.702	0.101	0.217	No association
BPQ-VSF | HC | EO | 263-381 ms	9	CF	0.358	-0.101	0.691	0.121	0.217	No association
BPQ-VSF | HC | EO | 263-381 ms	9	CFP	0.456	0.017	0.748	0.043	0.217	Nominal $p < .05$ only
BPQ-VSF | HC | EO | 263-381 ms	9	CPO	0.369	-0.088	0.697	0.110	0.217	No association
BPQ-VSF | PD | EO | 263-381 ms	9	LF	-0.379	-0.719	0.107	0.121	0.250	No association
BPQ-VSF | PD | EO | 263-381 ms	9	RF	-0.402	-0.732	0.080	0.098	0.250	No association
BPQ-VSF | PD | EO | 263-381 ms	9	LC	-0.340	-0.697	0.150	0.167	0.250	No association
BPQ-VSF | PD | EO | 263-381 ms	9	RC	-0.349	-0.702	0.140	0.155	0.250	No association
BPQ-VSF | PD | EO | 263-381 ms	9	LPO	-0.215	-0.619	0.280	0.393	0.393	No association
BPQ-VSF | PD | EO | 263-381 ms	9	RPO	-0.287	-0.665	0.207	0.248	0.279	No association
BPQ-VSF | PD | EO | 263-381 ms	9	CF	-0.348	-0.701	0.142	0.158	0.250	No association
BPQ-VSF | PD | EO | 263-381 ms	9	CFP	-0.398	-0.729	0.084	0.102	0.250	No association
BPQ-VSF | PD | EO | 263-381 ms	9	CPO	-0.291	-0.667	0.203	0.241	0.279	No association
Not-Distracting | HC | EC | 263-381 ms	9	LF	-0.262	-0.631	0.204	0.264	0.340	No association
Not-Distracting | HC | EC | 263-381 ms	9	RF	-0.284	-0.646	0.181	0.224	0.340	No association
Not-Distracting | HC | EC | 263-381 ms	9	LC	-0.315	-0.665	0.148	0.176	0.340	No association
Not-Distracting | HC | EC | 263-381 ms	9	RC	-0.283	-0.645	0.182	0.227	0.340	No association
Not-Distracting | HC | EC | 263-381 ms	9	LPO	-0.199	-0.590	0.267	0.401	0.446	No association
Not-Distracting | HC | EC | 263-381 ms	9	RPO	-0.331	-0.675	0.130	0.154	0.340	No association
Not-Distracting | HC | EC | 263-381 ms	9	CF	-0.351	-0.687	0.108	0.129	0.340	No association

FDR_family	FDR_family_size	Region	partial_*r*_controlling_age	CI_95_lower	CI_95_upper	*p*_partial	*q*_FDR_partial	Evidence_level
Not-Distracting | HC | EC | 263-381 ms	9	CFP	-0.429	-0.732	0.017	0.059	0.340	No association
Not-Distracting | HC | EC | 263-381 ms	9	CPO	-0.180	-0.577	0.285	0.446	0.446	No association
Not-Distracting | PD | EC | 263-381 ms	9	LF	0.165	-0.327	0.587	0.512	0.787	No association
Not-Distracting | PD | EC | 263-381 ms	9	RF	0.196	-0.298	0.607	0.435	0.787	No association
Not-Distracting | PD | EC | 263-381 ms	9	LC	0.151	-0.340	0.577	0.550	0.787	No association
Not-Distracting | PD | EC | 263-381 ms	9	RC	0.207	-0.288	0.614	0.410	0.787	No association
Not-Distracting | PD | EC | 263-381 ms	9	LPO	0.148	-0.343	0.575	0.559	0.787	No association
Not-Distracting | PD | EC | 263-381 ms	9	RPO	0.139	-0.350	0.569	0.581	0.787	No association
Not-Distracting | PD | EC | 263-381 ms	9	CF	0.049	-0.428	0.504	0.848	0.848	No association
Not-Distracting | PD | EC | 263-381 ms	9	CFP	0.098	-0.387	0.540	0.700	0.787	No association
Not-Distracting | PD | EC | 263-381 ms	9	CPO	0.126	-0.362	0.560	0.618	0.787	No association
Not-Distracting | HC | EO | 263-381 ms	9	LF	-0.242	-0.619	0.224	0.303	0.312	No association
Not-Distracting | HC | EO | 263-381 ms	9	RF	-0.337	-0.678	0.124	0.146	0.254	No association
Not-Distracting | HC | EO | 263-381 ms	9	LC	-0.323	-0.670	0.139	0.165	0.254	No association
Not-Distracting | HC | EO | 263-381 ms	9	RC	-0.341	-0.681	0.120	0.142	0.254	No association
Not-Distracting | HC | EO | 263-381 ms	9	LPO	-0.258	-0.629	0.208	0.272	0.312	No association
Not-Distracting | HC | EO | 263-381 ms	9	RPO	-0.320	-0.668	0.143	0.170	0.254	No association

FDR_family	FDR_family_size	Region	partial_*r*_controlling_age	CI_95_lower	CI_95_upper	*p*_partial	*q*_FDR_partial	Evidence_level
Not-Distracting | HC | EO | 263-381 ms	9	CF	-0.386	-0.708	0.068	0.092	0.254	No association
Not-Distracting | HC | EO | 263-381 ms	9	CFP	-0.389	-0.709	0.065	0.090	0.254	No association
Not-Distracting | HC | EO | 263-381 ms	9	CPO	-0.238	-0.616	0.228	0.312	0.312	No association
Not-Distracting | PD | EO | 263-381 ms	9	LF	0.172	-0.321	0.591	0.495	0.863	No association
Not-Distracting | PD | EO | 263-381 ms	9	RF	0.142	-0.348	0.571	0.575	0.863	No association
Not-Distracting | PD | EO | 263-381 ms	9	LC	0.130	-0.359	0.563	0.607	0.863	No association
Not-Distracting | PD | EO | 263-381 ms	9	RC	0.146	-0.345	0.574	0.564	0.863	No association
Not-Distracting | PD | EO | 263-381 ms	9	LPO	0.050	-0.427	0.505	0.843	0.863	No association
Not-Distracting | PD | EO | 263-381 ms	9	RPO	0.048	-0.428	0.504	0.850	0.863	No association
Not-Distracting | PD | EO | 263-381 ms	9	CF	0.138	-0.352	0.568	0.586	0.863	No association
Not-Distracting | PD | EO | 263-381 ms	9	CFP	0.100	-0.385	0.541	0.694	0.863	No association
Not-Distracting | PD | EO | 263-381 ms	9	CPO	0.044	-0.432	0.501	0.863	0.863	No association
Not-Worrying | HC | EC | 263-381 ms	9	LF	0.293	-0.172	0.651	0.210	0.831	No association
Not-Worrying | HC | EC | 263-381 ms	9	RF	0.185	-0.280	0.580	0.435	0.831	No association
Not-Worrying | HC | EC | 263-381 ms	9	LC	0.099	-0.359	0.519	0.676	0.870	No association
Not-Worrying | HC | EC | 263-381 ms	9	RC	0.005	-0.438	0.447	0.982	0.982	No association
Not-Worrying | HC | EC | 263-381 ms	9	LPO	0.040	-0.410	0.474	0.867	0.976	No association

FDR_family	FDR_family_size	Region	partial_*r*_controlling_age	CI_95_lower	CI_95_upper	*p*_partial	*q*_FDR_partial	Evidence_level
Not-Worrying | HC | EC | 263-381 ms	9	RPO	0.166	-0.299	0.567	0.485	0.831	No association
Not-Worrying | HC | EC | 263-381 ms	9	CF	0.141	-0.322	0.549	0.554	0.831	No association
Not-Worrying | HC | EC | 263-381 ms	9	CFP	0.194	-0.272	0.586	0.413	0.831	No association
Not-Worrying | HC | EC | 263-381 ms	9	CPO	0.208	-0.258	0.596	0.379	0.831	No association
Not-Worrying | PD | EC | 263-381 ms	9	LF	0.371	-0.116	0.714	0.130	0.584	No association
Not-Worrying | PD | EC | 263-381 ms	9	RF	0.381	-0.105	0.720	0.119	0.584	No association
Not-Worrying | PD | EC | 263-381 ms	9	LC	0.159	-0.333	0.583	0.529	0.624	No association
Not-Worrying | PD | EC | 263-381 ms	9	RC	0.247	-0.248	0.640	0.323	0.624	No association
Not-Worrying | PD | EC | 263-381 ms	9	LPO	0.124	-0.364	0.559	0.624	0.624	No association
Not-Worrying | PD | EC | 263-381 ms	9	RPO	0.182	-0.311	0.598	0.469	0.624	No association
Not-Worrying | PD | EC | 263-381 ms	9	CF	0.187	-0.306	0.602	0.456	0.624	No association
Not-Worrying | PD | EC | 263-381 ms	9	CFP	0.179	-0.314	0.596	0.478	0.624	No association
Not-Worrying | PD | EC | 263-381 ms	9	CPO	0.132	-0.357	0.564	0.602	0.624	No association
Not-Worrying | HC | EO | 263-381 ms	9	LF	0.012	-0.433	0.452	0.961	0.977	No association
Not-Worrying | HC | EO | 263-381 ms	9	RF	0.007	-0.437	0.448	0.977	0.977	No association
Not-Worrying | HC | EO | 263-381 ms	9	LC	-0.009	-0.450	0.435	0.971	0.977	No association
Not-Worrying | HC | EO | 263-381 ms	9	RC	-0.027	-0.464	0.420	0.909	0.977	No association

FDR_family	FDR_family_size	Region	partial_*r*_controlling_age	CI_95_lower	CI_95_upper	*p*_partial	*q*_FDR_partial	Evidence_level
Not-Worrying | HC | EO | 263-381 ms	9	LPO	-0.158	-0.562	0.306	0.505	0.977	No association
Not-Worrying | HC | EO | 263-381 ms	9	RPO	0.061	-0.392	0.490	0.798	0.977	No association
Not-Worrying | HC | EO | 263-381 ms	9	CF	-0.042	-0.476	0.408	0.860	0.977	No association
Not-Worrying | HC | EO | 263-381 ms	9	CFP	-0.161	-0.564	0.303	0.497	0.977	No association
Not-Worrying | HC | EO | 263-381 ms	9	CPO	-0.059	-0.489	0.394	0.806	0.977	No association
Not-Worrying | PD | EO | 263-381 ms	9	LF	0.110	-0.376	0.549	0.663	0.745	No association
Not-Worrying | PD | EO | 263-381 ms	9	RF	0.178	-0.315	0.595	0.480	0.745	No association
Not-Worrying | PD | EO | 263-381 ms	9	LC	0.133	-0.356	0.565	0.599	0.745	No association
Not-Worrying | PD | EO | 263-381 ms	9	RC	0.078	-0.403	0.526	0.757	0.757	No association
Not-Worrying | PD | EO | 263-381 ms	9	LPO	0.115	-0.372	0.552	0.650	0.745	No association
Not-Worrying | PD | EO | 263-381 ms	9	RPO	0.128	-0.360	0.561	0.613	0.745	No association
Not-Worrying | PD | EO | 263-381 ms	9	CF	0.111	-0.375	0.549	0.661	0.745	No association
Not-Worrying | PD | EO | 263-381 ms	9	CFP	0.135	-0.354	0.566	0.594	0.745	No association
Not-Worrying | PD | EO | 263-381 ms	9	CPO	0.131	-0.358	0.563	0.604	0.745	No association
Emotional Awareness | HC | EC | 263-381 ms	9	LF	-0.242	-0.618	0.225	0.305	0.935	No association
Emotional Awareness | HC | EC | 263-381 ms	9	RF	-0.153	-0.558	0.310	0.519	0.935	No association
Emotional Awareness | HC | EC | 263-381 ms	9	LC	-0.128	-0.540	0.334	0.591	0.935	No association

FDR_family	FDR_family_size	Region	partial_*r*_controlling_age	CI_95_lower	CI_95_upper	*p*_partial	*q*_FDR_partial	Evidence_level
Emotional Awareness | HC | EC | 263-381 ms	9	RC	0.020	-0.427	0.458	0.935	0.935	No association
Emotional Awareness | HC | EC | 263-381 ms	9	LPO	-0.125	-0.538	0.336	0.600	0.935	No association
Emotional Awareness | HC | EC | 263-381 ms	9	RPO	-0.051	-0.483	0.400	0.830	0.935	No association
Emotional Awareness | HC | EC | 263-381 ms	9	CF	-0.070	-0.497	0.384	0.769	0.935	No association
Emotional Awareness | HC | EC | 263-381 ms	9	CFP	0.047	-0.404	0.480	0.844	0.935	No association
Emotional Awareness | HC | EC | 263-381 ms	9	CPO	-0.105	-0.523	0.354	0.659	0.935	No association
Emotional Awareness | PD | EC | 263-381 ms	9	LF	-0.104	-0.544	0.381	0.681	0.956	No association
Emotional Awareness | PD | EC | 263-381 ms	9	RF	0.076	-0.405	0.525	0.763	0.956	No association
Emotional Awareness | PD | EC | 263-381 ms	9	LC	0.018	-0.453	0.481	0.943	0.956	No association
Emotional Awareness | PD | EC | 263-381 ms	9	RC	0.014	-0.456	0.478	0.956	0.956	No association
Emotional Awareness | PD | EC | 263-381 ms	9	LPO	0.118	-0.369	0.554	0.641	0.956	No association
Emotional Awareness | PD | EC | 263-381 ms	9	RPO	0.108	-0.378	0.547	0.670	0.956	No association
Emotional Awareness | PD | EC | 263-381 ms	9	CF	0.056	-0.422	0.510	0.825	0.956	No association
Emotional Awareness | PD | EC | 263-381 ms	9	CFP	0.073	-0.408	0.522	0.775	0.956	No association
Emotional Awareness | PD | EC | 263-381 ms	9	CPO	0.152	-0.339	0.578	0.548	0.956	No association
Emotional Awareness | HC | EO | 263-381 ms	9	LF	0.219	-0.247	0.603	0.353	0.353	No association
Emotional Awareness | HC | EO | 263-381 ms	9	RF	0.300	-0.164	0.655	0.199	0.256	No association

**FDR_family**	**FDR_family_size**	**Region**	**partial_*r*_controlling_age**	**CI_95_lower**	**CI_95_upper**	***p*_partial**	***q*_FDR_partial**	**Evidence_level**
Emotional Awareness | HC | EO | 263-381 ms	9	LC	0.306	-0.157	0.659	0.189	0.256	No association
Emotional Awareness | HC | EO | 263-381 ms	9	RC	0.345	-0.115	0.683	0.137	0.256	No association
Emotional Awareness | HC | EO | 263-381 ms	9	LPO	0.352	-0.107	0.688	0.128	0.256	No association
Emotional Awareness | HC | EO | 263-381 ms	9	RPO	0.227	-0.240	0.608	0.336	0.353	No association
Emotional Awareness | HC | EO | 263-381 ms	9	CF	0.314	-0.149	0.664	0.178	0.256	No association
Emotional Awareness | HC | EO | 263-381 ms	9	CFP	0.476	0.042	0.759	0.034	0.256	Nominal $p < .05$ only
Emotional Awareness | HC | EO | 263-381 ms	9	CPO	0.323	-0.139	0.670	0.165	0.256	No association
Emotional Awareness | PD | EO | 263-381 ms	9	LF	0.069	-0.411	0.519	0.785	0.785	No association
Emotional Awareness | PD | EO | 263-381 ms	9	RF	0.218	-0.277	0.622	0.384	0.553	No association
Emotional Awareness | PD | EO | 263-381 ms	9	LC	0.173	-0.319	0.592	0.491	0.553	No association
Emotional Awareness | PD | EO | 263-381 ms	9	RC	0.197	-0.298	0.608	0.434	0.553	No association
Emotional Awareness | PD | EO | 263-381 ms	9	LPO	0.265	-0.231	0.651	0.288	0.553	No association
Emotional Awareness | PD | EO | 263-381 ms	9	RPO	0.192	-0.302	0.605	0.445	0.553	No association
Emotional Awareness | PD | EO | 263-381 ms	9	CF	0.216	-0.279	0.621	0.388	0.553	No association
Emotional Awareness | PD | EO | 263-381 ms	9	CFP	0.263	-0.233	0.650	0.292	0.553	No association
Emotional Awareness | PD | EO | 263-381 ms	9	CPO	0.297	-0.197	0.671	0.231	0.553	No association
Trusting | HC | EC | 263-381 ms	9	LF	0.293	-0.172	0.651	0.211	0.271	No association

FDR_family	FDR_family_size	Region	partial_*r*_controlling_age	CI_95_lower	CI_95_upper	*p*_partial	*q*_FDR_partial	Evidence_level
Trusting | HC | EC | 263-381 ms	9	RF	0.312	-0.152	0.663	0.181	0.271	No association
Trusting | HC | EC | 263-381 ms	9	LC	0.335	-0.126	0.677	0.149	0.271	No association
Trusting | HC | EC | 263-381 ms	9	RC	0.363	-0.095	0.694	0.116	0.271	No association
Trusting | HC | EC | 263-381 ms	9	LPO	0.213	-0.253	0.599	0.366	0.366	No association
Trusting | HC | EC | 263-381 ms	9	RPO	0.325	-0.138	0.671	0.163	0.271	No association
Trusting | HC | EC | 263-381 ms	9	CF	0.430	-0.016	0.733	0.059	0.271	No association
Trusting | HC | EC | 263-381 ms	9	CFP	0.383	-0.072	0.706	0.096	0.271	No association
Trusting | HC | EC | 263-381 ms	9	CPO	0.268	-0.198	0.635	0.253	0.284	No association
Trusting | PD | EC | 263-381 ms	9	LF	0.156	-0.335	0.581	0.536	0.984	No association
Trusting | PD | EC | 263-381 ms	9	RF	0.123	-0.365	0.558	0.626	0.984	No association
Trusting | PD | EC | 263-381 ms	9	LC	0.025	-0.447	0.487	0.920	0.984	No association
Trusting | PD | EC | 263-381 ms	9	RC	0.031	-0.442	0.491	0.903	0.984	No association
Trusting | PD | EC | 263-381 ms	9	LPO	-0.017	-0.480	0.454	0.947	0.984	No association
Trusting | PD | EC | 263-381 ms	9	RPO	-0.056	-0.510	0.422	0.824	0.984	No association
Trusting | PD | EC | 263-381 ms	9	CF	0.049	-0.428	0.504	0.847	0.984	No association
Trusting | PD | EC | 263-381 ms	9	CFP	-0.005	-0.471	0.463	0.984	0.984	No association
Trusting | PD | EC | 263-381 ms	9	CPO	-0.058	-0.511	0.420	0.819	0.984	No association
Trusting | HC | EO | 263-381 ms	9	LF	0.084	-0.373	0.507	0.726	0.989	No association
Trusting | HC | EO | 263-381 ms	9	RF	0.118	-0.342	0.533	0.620	0.989	No association
Trusting | HC | EO | 263-381 ms	9	LC	-0.082	-0.506	0.374	0.732	0.989	No association
Trusting | HC | EO | 263-381 ms	9	RC	-0.043	-0.477	0.407	0.856	0.989	No association
Trusting | HC | EO | 263-381 ms	9	LPO	-0.008	-0.449	0.436	0.975	0.989	No association
Trusting | HC | EO | 263-381 ms	9	RPO	-0.003	-0.445	0.440	0.989	0.989	No association
Trusting | HC | EO | 263-381 ms	9	CF	0.154	-0.310	0.558	0.517	0.989	No association
Trusting | HC | EO | 263-381 ms	9	CFP	0.032	-0.416	0.468	0.894	0.989	No association
Trusting | HC | EO | 263-381 ms	9	CPO	0.049	-0.402	0.481	0.837	0.989	No association
Trusting | PD | EO | 263-381 ms	9	LF	-0.052	-0.506	0.425	0.838	0.998	No association
Trusting | PD | EO | 263-381 ms	9	RF	-0.004	-0.470	0.464	0.987	0.998	No association

FDR_family	FDR_family_size	Region	partial_*r*_controlling_age	CI_95_lower	CI_95_upper	*p*_partial	*q*_FDR_partial	Evidence_level
Trusting | PD | EO | 263-381 ms	9	LC	-0.003	-0.469	0.465	0.991	0.998	No association
Trusting | PD | EO | 263-381 ms	9	RC	0.001	-0.466	0.467	0.998	0.998	No association
Trusting | PD | EO | 263-381 ms	9	LPO	0.097	-0.387	0.540	0.700	0.998	No association
Trusting | PD | EO | 263-381 ms	9	RPO	0.043	-0.432	0.500	0.865	0.998	No association
Trusting | PD | EO | 263-381 ms	9	CF	-0.003	-0.469	0.465	0.991	0.998	No association
Trusting | PD | EO | 263-381 ms	9	CFP	0.008	-0.460	0.473	0.974	0.998	No association
Trusting | PD | EO | 263-381 ms	9	CPO	0.048	-0.429	0.504	0.850	0.998	No association

Note. Partial correlations were computed within each group while controlling for age. FDR correction was applied across the nine ROIs within each group, HEP time window, resting-state condition, and self-report construct.

Supplementary Table S5B. HEP age-adjusted group × self-report interaction results.

FDR_family	FDR_family_size	Region	Interaction_term	Interaction_beta	CI_95_lower	CI_95_upper	*p*_interaction	*q*_FDR_interaction	Age_beta	Age_*p*	Evidence_level
BPQ-VSF | EC | 235-301 ms	9	LF	BPQ-VSF x Group	-0.228	-0.440	-0.017	0.035	0.045	0.008	0.680	FDR-corrected $q < .05$
BPQ-VSF | EC | 235-301 ms	9	RF	BPQ-VSF x Group	-0.232	-0.445	-0.019	0.034	0.045	0.014	0.473	FDR-corrected $q < .05$
BPQ-VSF | EC | 235-301 ms	9	LC	BPQ-VSF x Group	-0.260	-0.484	-0.037	0.024	0.045	0.006	0.766	FDR-corrected $q < .05$
BPQ-VSF | EC | 235-301 ms	9	RC	BPQ-VSF x Group	-0.184	-0.412	0.044	0.111	0.111	0.006	0.765	No association
BPQ-VSF | EC | 235-301 ms	9	LPO	BPQ-VSF x Group	-0.238	-0.452	-0.025	0.030	0.045	0.006	0.754	FDR-corrected $q < .05$
BPQ-VSF | EC | 235-301 ms	9	RPO	BPQ-VSF x Group	-0.240	-0.452	-0.028	0.027	0.045	0.008	0.691	FDR-corrected $q < .05$
BPQ-VSF | EC | 235-301 ms	9	CF	BPQ-VSF x Group	-0.283	-0.505	-0.060	0.014	0.045	0.022	0.277	FDR-corrected $q < .05$
BPQ-VSF | EC | 235-301 ms	9	CFP	BPQ-VSF x Group	-0.276	-0.490	-0.062	0.013	0.045	0.016	0.413	FDR-corrected $q < .05$
BPQ-VSF | EC | 235-301 ms	9	CPO	BPQ-VSF x Group	-0.237	-0.468	-0.006	0.045	0.050	0.007	0.727	Exploratory FDR-level $.05 \leq q < .10$
BPQ-VSF | EO | 235-301 ms	9	LF	BPQ-VSF x Group	-0.298	-0.500	-0.096	0.005	0.009	0.024	0.209	FDR-corrected $q < .05$
BPQ-VSF | EO | 235-301 ms	9	RF	BPQ-VSF x Group	-0.308	-0.499	-0.117	0.002	0.007	0.033	0.066	FDR-corrected $q < .05$
BPQ-VSF | EO | 235-301 ms	9	LC	BPQ-VSF x Group	-0.310	-0.516	-0.105	0.004	0.009	0.030	0.123	FDR-corrected $q < .05$
BPQ-VSF | EO | 235-301 ms	9	RC	BPQ-VSF x Group	-0.314	-0.537	-0.091	0.007	0.009	0.025	0.236	FDR-corrected $q < .05$
BPQ-VSF | EO | 235-301 ms	9	LPO	BPQ-VSF x Group	-0.271	-0.494	-0.049	0.018	0.018	0.031	0.140	FDR-corrected $q < .05$
BPQ-VSF | EO | 235-301 ms	9	RPO	BPQ-VSF x Group	-0.322	-0.564	-0.080	0.011	0.012	0.029	0.201	FDR-corrected $q < .05$

FDR_family	FDR_family_size	Region	Interaction_term	Interaction_beta	CI_95_lower	CI_95_upper	*p*_interaction	*q*_FDR_interaction	Age_beta	Age_*p*	Evidence_level
BPQ-VSF | EO | 235-301 ms	9	CF	BPQ-VSF x Group	-0.376	-0.609	-0.143	0.002	0.007	0.032	0.141	FDR-corrected *q* < .05
BPQ-VSF | EO | 235-301 ms	9	CFP	BPQ-VSF x Group	-0.385	-0.602	-0.169	0.001	0.007	0.027	0.179	FDR-corrected *q* < .05
BPQ-VSF | EO | 235-301 ms	9	CPO	BPQ-VSF x Group	-0.325	-0.555	-0.095	0.007	0.009	0.030	0.166	FDR-corrected *q* < .05
Not-Distracting | EC | 235-301 ms	9	LF	Not-Distracting x Group	0.483	-0.429	1.395	0.290	0.310	-0.001	0.976	No association
Not-Distracting | EC | 235-301 ms	9	RF	Not-Distracting x Group	0.636	-0.268	1.540	0.162	0.282	0.004	0.869	No association
Not-Distracting | EC | 235-301 ms	9	LC	Not-Distracting x Group	0.649	-0.311	1.608	0.179	0.282	-0.004	0.857	No association
Not-Distracting | EC | 235-301 ms	9	RC	Not-Distracting x Group	0.676	-0.270	1.622	0.156	0.282	-0.006	0.787	No association
Not-Distracting | EC | 235-301 ms	9	LPO	Not-Distracting x Group	0.547	-0.381	1.475	0.240	0.308	-0.008	0.742	No association
Not-Distracting | EC | 235-301 ms	9	RPO	Not-Distracting x Group	0.708	-0.200	1.615	0.123	0.282	-0.005	0.819	No association
Not-Distracting | EC | 235-301 ms	9	CF	Not-Distracting x Group	0.641	-0.328	1.609	0.188	0.282	0.012	0.623	No association
Not-Distracting | EC | 235-301 ms	9	CFP	Not-Distracting x Group	0.614	-0.306	1.534	0.184	0.282	0.011	0.625	No association
Not-Distracting | EC | 235-301 ms	9	CPO	Not-Distracting x Group	0.503	-0.487	1.493	0.310	0.310	-0.002	0.943	No association
Not-Distracting | EO | 235-301 ms	9	LF	Not-Distracting x Group	0.577	-0.329	1.484	0.204	0.307	0.015	0.503	No association
Not-Distracting | EO | 235-301 ms	9	RF	Not-Distracting x Group	0.764	-0.086	1.615	0.077	0.296	0.023	0.280	No association
Not-Distracting | EO | 235-301 ms	9	LC	Not-Distracting x Group	0.593	-0.322	1.509	0.197	0.307	0.023	0.320	No association
Not-Distracting | EO | 235-301 ms	9	RC	Not-Distracting x Group	0.805	-0.165	1.775	0.101	0.296	0.013	0.578	No association

FDR_family	FDR_family_size	Region	Interaction_term	Interaction_beta	CI_95_lower	CI_95_upper	*p*_interaction	*q*_FDR_interaction	Age_beta	Age_*p*	Evidence_level
Not-Distracting | EO | 235-301 ms	9	LPO	Not-Distracting x Group	0.312	-0.663	1.286	0.520	0.520	0.028	0.248	No association
Not-Distracting | EO | 235-301 ms	9	RPO	Not-Distracting x Group	0.501	-0.552	1.553	0.341	0.438	0.026	0.329	No association
Not-Distracting | EO | 235-301 ms	9	CF	Not-Distracting x Group	0.959	-0.076	1.995	0.068	0.296	0.015	0.547	No association
Not-Distracting | EO | 235-301 ms	9	CFP	Not-Distracting x Group	0.754	-0.238	1.746	0.132	0.296	0.018	0.471	No association
Not-Distracting | EO | 235-301 ms	9	CPO	Not-Distracting x Group	0.408	-0.616	1.432	0.424	0.477	0.027	0.299	No association
Not-Worrying | EC | 235-301 ms	9	LF	Not-Worrying x Group	0.616	-0.387	1.618	0.221	0.290	-0.008	0.675	No association
Not-Worrying | EC | 235-301 ms	9	RF	Not-Worrying x Group	0.742	-0.259	1.742	0.141	0.290	-0.002	0.906	No association
Not-Worrying | EC | 235-301 ms	9	LC	Not-Worrying x Group	0.812	-0.278	1.901	0.139	0.290	-0.009	0.683	No association
Not-Worrying | EC | 235-301 ms	9	RC	Not-Worrying x Group	0.806	-0.266	1.878	0.136	0.290	-0.009	0.668	No association
Not-Worrying | EC | 235-301 ms	9	LPO	Not-Worrying x Group	0.707	-0.343	1.757	0.180	0.290	-0.010	0.640	No association
Not-Worrying | EC | 235-301 ms	9	RPO	Not-Worrying x Group	0.669	-0.375	1.712	0.202	0.290	-0.013	0.523	No association
Not-Worrying | EC | 235-301 ms	9	CF	Not-Worrying x Group	0.666	-0.432	1.765	0.226	0.290	0.007	0.756	No association
Not-Worrying | EC | 235-301 ms	9	CFP	Not-Worrying x Group	0.593	-0.453	1.640	0.258	0.290	0.002	0.921	No association
Not-Worrying | EC | 235-301 ms	9	CPO	Not-Worrying x Group	0.497	-0.617	1.611	0.371	0.371	-0.008	0.702	No association
Not-Worrying | EO | 235-301 ms	9	LF	Not-Worrying x Group	0.592	-0.455	1.638	0.259	0.259	0.014	0.499	No association
Not-Worrying | EO | 235-301 ms	9	RF	Not-Worrying x Group	0.705	-0.288	1.698	0.158	0.249	0.021	0.290	No association

FDR_family	FDR_family_size	Region	Interaction_term	Interaction_beta	CI_95_lower	CI_95_upper	*p*_interaction	*q*_FDR_interaction	Age_beta	Age_*p*	Evidence_level
Not-Worrying | EO | 235-301 ms	9	LC	Not-Worrying x Group	0.814	-0.237	1.865	0.125	0.249	0.016	0.440	No association
Not-Worrying | EO | 235-301 ms	9	RC	Not-Worrying x Group	0.695	-0.445	1.835	0.224	0.252	0.012	0.588	No association
Not-Worrying | EO | 235-301 ms	9	LPO	Not-Worrying x Group	0.963	-0.124	2.050	0.081	0.249	0.016	0.451	No association
Not-Worrying | EO | 235-301 ms	9	RPO	Not-Worrying x Group	0.838	-0.369	2.044	0.168	0.249	0.011	0.637	No association
Not-Worrying | EO | 235-301 ms	9	CF	Not-Worrying x Group	0.799	-0.424	2.022	0.193	0.249	0.015	0.528	No association
Not-Worrying | EO | 235-301 ms	9	CFP	Not-Worrying x Group	0.954	-0.191	2.100	0.099	0.249	0.011	0.626	No association
Not-Worrying | EO | 235-301 ms	9	CPO	Not-Worrying x Group	0.915	-0.241	2.071	0.117	0.249	0.013	0.571	No association
Emotional Awareness | EC | 235-301 ms	9	LF	Emotional Awareness x Group	-0.020	-0.882	0.841	0.962	0.986	-0.003	0.889	No association
Emotional Awareness | EC | 235-301 ms	9	RF	Emotional Awareness x Group	-0.008	-0.881	0.866	0.986	0.986	0.007	0.754	No association
Emotional Awareness | EC | 235-301 ms	9	LC	Emotional Awareness x Group	-0.079	-1.008	0.849	0.864	0.986	0.000	0.994	No association
Emotional Awareness | EC | 235-301 ms	9	RC	Emotional Awareness x Group	-0.309	-1.218	0.601	0.496	0.986	0.000	0.988	No association
Emotional Awareness | EC | 235-301 ms	9	LPO	Emotional Awareness x Group	-0.038	-0.926	0.850	0.931	0.986	0.000	0.997	No association
Emotional Awareness | EC | 235-301 ms	9	RPO	Emotional Awareness x Group	-0.143	-1.039	0.753	0.748	0.986	0.000	0.992	No association

FDR_family	FDR_family_size	Region	Interaction_term	Interaction_beta	CI_95_lower	CI_95_upper	*p*_interaction	*q*_FDR_interaction	Age_beta	Age_*p*	Evidence_level
Emotional Awareness | EC | 235-301 ms	9	CF	Emotional Awareness x Group	-0.201	-1.135	0.733	0.665	0.986	0.014	0.543	No association
Emotional Awareness | EC | 235-301 ms	9	CFP	Emotional Awareness x Group	-0.010	-0.911	0.891	0.983	0.986	0.015	0.492	No association
Emotional Awareness | EC | 235-301 ms	9	CPO	Emotional Awareness x Group	0.116	-0.828	1.061	0.804	0.986	0.005	0.819	No association
Emotional Awareness | EO | 235-301 ms	9	LF	Emotional Awareness x Group	-0.263	-1.126	0.599	0.539	0.643	0.023	0.274	No association
Emotional Awareness | EO | 235-301 ms	9	RF	Emotional Awareness x Group	-0.288	-1.110	0.534	0.482	0.643	0.034	0.092	No association
Emotional Awareness | EO | 235-301 ms	9	LC	Emotional Awareness x Group	-0.235	-1.092	0.622	0.582	0.643	0.033	0.122	No association
Emotional Awareness | EO | 235-301 ms	9	RC	Emotional Awareness x Group	-0.448	-1.359	0.463	0.325	0.643	0.027	0.233	No association
Emotional Awareness | EO | 235-301 ms	9	LPO	Emotional Awareness x Group	-0.261	-1.124	0.603	0.544	0.643	0.037	0.082	No association
Emotional Awareness | EO | 235-301 ms	9	RPO	Emotional Awareness x Group	-0.324	-1.304	0.655	0.506	0.643	0.031	0.192	No association
Emotional Awareness | EO | 235-301 ms	9	CF	Emotional Awareness x Group	-0.512	-1.504	0.481	0.302	0.643	0.029	0.228	No association
Emotional Awareness | EO | 235-301 ms	9	CFP	Emotional Awareness x Group	-0.285	-1.194	0.625	0.530	0.643	0.033	0.139	No association

FDR_family	FDR_family_size	Region	Interaction_term	Interaction_beta	CI_95_lower	CI_95_upper	*p*_interaction	*q*_FDR_interaction	Age_beta	Age_*p*	Evidence_level
Emotional Awareness | EO | 235-301 ms	9	CPO	Emotional Awareness x Group	-0.211	-1.124	0.703	0.643	0.643	0.037	0.103	No association
Trusting | EC | 235-301 ms	9	LF	Trusting x Group	-0.010	-0.786	0.766	0.979	0.979	0.003	0.914	No association
Trusting | EC | 235-301 ms	9	RF	Trusting x Group	-0.077	-0.856	0.701	0.841	0.979	0.007	0.751	No association
Trusting | EC | 235-301 ms	9	LC	Trusting x Group	-0.121	-0.945	0.703	0.768	0.979	-0.002	0.935	No association
Trusting | EC | 235-301 ms	9	RC	Trusting x Group	-0.180	-0.991	0.631	0.655	0.979	-0.004	0.867	No association
Trusting | EC | 235-301 ms	9	LPO	Trusting x Group	-0.055	-0.840	0.730	0.888	0.979	-0.002	0.920	No association
Trusting | EC | 235-301 ms	9	RPO	Trusting x Group	-0.037	-0.836	0.763	0.927	0.979	-0.003	0.890	No association
Trusting | EC | 235-301 ms	9	CF	Trusting x Group	-0.403	-1.224	0.418	0.326	0.979	0.005	0.837	No association
Trusting | EC | 235-301 ms	9	CFP	Trusting x Group	-0.082	-0.883	0.719	0.836	0.979	0.010	0.670	No association
Trusting | EC | 235-301 ms	9	CPO	Trusting x Group	-0.155	-0.996	0.686	0.711	0.979	-0.003	0.890	No association
Trusting | EO | 235-301 ms	9	LF	Trusting x Group	-0.077	-0.844	0.690	0.840	0.919	0.018	0.428	No association
Trusting | EO | 235-301 ms	9	RF	Trusting x Group	-0.118	-0.854	0.618	0.747	0.919	0.026	0.237	No association
Trusting | EO | 235-301 ms	9	LC	Trusting x Group	0.169	-0.617	0.956	0.664	0.919	0.030	0.204	No association
Trusting | EO | 235-301 ms	9	RC	Trusting x Group	0.043	-0.801	0.887	0.919	0.919	0.021	0.398	No association
Trusting | EO | 235-301 ms	9	LPO	Trusting x Group	0.353	-0.464	1.170	0.386	0.919	0.037	0.142	No association
Trusting | EO | 235-301 ms	9	RPO	Trusting x Group	0.369	-0.535	1.272	0.413	0.919	0.033	0.222	No association

FDR_family	FDR_family_size	Region	Interaction_term	Interaction_beta	CI_95_lower	CI_95_upper	*p*_interaction	*q*_FDR_interaction	Age_beta	Age_*p*	Evidence_level
Trusting | EO | 235-301 ms	9	CF	Trusting x Group	-0.281	-1.180	0.618	0.529	0.919	0.016	0.549	No association
Trusting | EO | 235-301 ms	9	CFP	Trusting x Group	0.178	-0.683	1.039	0.677	0.919	0.027	0.299	No association
Trusting | EO | 235-301 ms	9	CPO	Trusting x Group	0.225	-0.649	1.098	0.605	0.919	0.031	0.245	No association
BPQ-VSF | EC | 263-381 ms	9	LF	BPQ-VSF x Group	-0.100	-0.301	0.100	0.318	0.423	0.017	0.353	No association
BPQ-VSF | EC | 263-381 ms	9	RF	BPQ-VSF x Group	-0.103	-0.300	0.094	0.298	0.423	0.025	0.172	No association
BPQ-VSF | EC | 263-381 ms	9	LC	BPQ-VSF x Group	-0.126	-0.349	0.096	0.257	0.423	0.027	0.188	No association
BPQ-VSF | EC | 263-381 ms	9	RC	BPQ-VSF x Group	-0.094	-0.290	0.102	0.339	0.423	0.027	0.141	No association
BPQ-VSF | EC | 263-381 ms	9	LPO	BPQ-VSF x Group	-0.078	-0.304	0.148	0.487	0.487	0.032	0.130	No association
BPQ-VSF | EC | 263-381 ms	9	RPO	BPQ-VSF x Group	-0.121	-0.331	0.089	0.251	0.423	0.026	0.192	No association
BPQ-VSF | EC | 263-381 ms	9	CF	BPQ-VSF x Group	-0.170	-0.393	0.053	0.130	0.423	0.034	0.105	No association
BPQ-VSF | EC | 263-381 ms	9	CFP	BPQ-VSF x Group	-0.159	-0.379	0.061	0.151	0.423	0.035	0.086	No association
BPQ-VSF | EC | 263-381 ms	9	CPO	BPQ-VSF x Group	-0.100	-0.328	0.127	0.376	0.423	0.035	0.103	No association
BPQ-VSF | EO | 263-381 ms	9	LF	BPQ-VSF x Group	-0.220	-0.441	0.001	0.051	0.058	0.033	0.111	Exploratory FDR-level $.05 \leq q < .10$
BPQ-VSF | EO | 263-381 ms	9	RF	BPQ-VSF x Group	-0.218	-0.423	-0.013	0.038	0.053	0.041	0.037	Exploratory FDR-level $.05 \leq q < .10$
BPQ-VSF | EO | 263-381 ms	9	LC	BPQ-VSF x Group	-0.231	-0.440	-0.021	0.032	0.053	0.049	0.015	Exploratory FDR-level $.05 \leq q < .10$
BPQ-VSF | EO | 263-381 ms	9	RC	BPQ-VSF x Group	-0.261	-0.482	-0.040	0.022	0.053	0.044	0.037	Exploratory FDR-level $.05 \leq q < .10$

FDR_family	FDR_family_size	Region	Interaction_term	Interaction_beta	CI_95_lower	CI_95_upper	*p*_interaction	*q*_FDR_interaction	Age_beta	Age_*p*	Evidence_level
BPQ-VSF | EO | 263-381 ms	9	LPO	BPQ-VSF x Group	-0.163	-0.378	0.051	0.131	0.131	0.053	0.010	No association
BPQ-VSF | EO | 263-381 ms	9	RPO	BPQ-VSF x Group	-0.257	-0.503	-0.011	0.041	0.053	0.047	0.044	Exploratory FDR-level $.05 \leq q < .10$
BPQ-VSF | EO | 263-381 ms	9	CF	BPQ-VSF x Group	-0.306	-0.549	-0.064	0.015	0.053	0.039	0.086	Exploratory FDR-level $.05 \leq q < .10$
BPQ-VSF | EO | 263-381 ms	9	CFP	BPQ-VSF x Group	-0.311	-0.522	-0.101	0.005	0.044	0.042	0.036	FDR-corrected $q < .05$
BPQ-VSF | EO | 263-381 ms	9	CPO	BPQ-VSF x Group	-0.238	-0.456	-0.020	0.033	0.053	0.054	0.010	Exploratory FDR-level $.05 \leq q < .10$
Not-Distracting | EC | 263-381 ms	9	LF	Not-Distracting x Group	0.675	-0.129	1.480	0.097	0.125	0.007	0.721	No association
Not-Distracting | EC | 263-381 ms	9	RF	Not-Distracting x Group	0.686	-0.102	1.475	0.086	0.125	0.016	0.426	No association
Not-Distracting | EC | 263-381 ms	9	LC	Not-Distracting x Group	0.794	-0.100	1.689	0.080	0.125	0.016	0.472	No association
Not-Distracting | EC | 263-381 ms	9	RC	Not-Distracting x Group	0.758	-0.018	1.535	0.055	0.125	0.013	0.489	No association
Not-Distracting | EC | 263-381 ms	9	LPO	Not-Distracting x Group	0.715	-0.190	1.619	0.118	0.133	0.018	0.429	No association
Not-Distracting | EC | 263-381 ms	9	RPO	Not-Distracting x Group	0.763	-0.081	1.608	0.075	0.125	0.012	0.573	No association
Not-Distracting | EC | 263-381 ms	9	CF	Not-Distracting x Group	0.810	-0.086	1.707	0.075	0.125	0.025	0.256	No association
Not-Distracting | EC | 263-381 ms	9	CFP	Not-Distracting x Group	0.792	-0.093	1.676	0.078	0.125	0.028	0.200	No association
Not-Distracting | EC | 263-381 ms	9	CPO	Not-Distracting x Group	0.603	-0.323	1.528	0.195	0.195	0.023	0.313	No association
Not-Distracting | EO | 263-381 ms	9	LF	Not-Distracting x Group	0.681	-0.264	1.626	0.152	0.239	0.023	0.320	No association
Not-Distracting | EO | 263-381 ms	9	RF	Not-Distracting x Group	0.680	-0.195	1.554	0.124	0.239	0.033	0.129	No association

FDR_family	FDR_family_size	Region	Interaction_term	Interaction_beta	CI_95_lower	CI_95_upper	*p*_interaction	*q*_FDR_interaction	Age_beta	Age_*p*	Evidence_level
Not-Distracting | EO | 263-381 ms	9	LC	Not-Distracting x Group	0.634	-0.261	1.528	0.159	0.239	0.041	0.072	No association
Not-Distracting | EO | 263-381 ms	9	RC	Not-Distracting x Group	0.744	-0.202	1.691	0.119	0.239	0.033	0.167	No association
Not-Distracting | EO | 263-381 ms	9	LPO	Not-Distracting x Group	0.495	-0.399	1.389	0.269	0.308	0.046	0.042	No association
Not-Distracting | EO | 263-381 ms	9	RPO	Not-Distracting x Group	0.543	-0.509	1.594	0.302	0.308	0.041	0.122	No association
Not-Distracting | EO | 263-381 ms	9	CF	Not-Distracting x Group	1.021	0.002	2.041	0.050	0.239	0.024	0.344	Nominal $p < .05$ only
Not-Distracting | EO | 263-381 ms	9	CFP	Not-Distracting x Group	0.781	-0.147	1.709	0.096	0.239	0.031	0.186	No association
Not-Distracting | EO | 263-381 ms	9	CPO	Not-Distracting x Group	0.479	-0.462	1.421	0.308	0.308	0.046	0.053	No association
Not-Worrying | EC | 263-381 ms	9	LF	Not-Worrying x Group	0.326	-0.571	1.224	0.465	0.690	0.007	0.681	No association
Not-Worrying | EC | 263-381 ms	9	RF	Not-Worrying x Group	0.449	-0.447	1.345	0.316	0.690	0.016	0.375	No association
Not-Worrying | EC | 263-381 ms	9	LC	Not-Worrying x Group	0.363	-0.690	1.416	0.489	0.690	0.021	0.311	No association
Not-Worrying | EC | 263-381 ms	9	RC	Not-Worrying x Group	0.517	-0.397	1.430	0.259	0.690	0.018	0.322	No association
Not-Worrying | EC | 263-381 ms	9	LPO	Not-Worrying x Group	0.355	-0.704	1.415	0.501	0.690	0.025	0.242	No association
Not-Worrying | EC | 263-381 ms	9	RPO	Not-Worrying x Group	0.261	-0.733	1.254	0.598	0.690	0.015	0.447	No association
Not-Worrying | EC | 263-381 ms	9	CF	Not-Worrying x Group	0.287	-0.777	1.352	0.587	0.690	0.026	0.217	No association
Not-Worrying | EC | 263-381 ms	9	CFP	Not-Worrying x Group	0.262	-0.783	1.308	0.614	0.690	0.029	0.164	No association
Not-Worrying | EC | 263-381 ms	9	CPO	Not-Worrying x Group	0.146	-0.918	1.211	0.782	0.782	0.028	0.187	No association

FDR_family	FDR_family_size	Region	Interaction_term	Interaction_beta	CI_95_lower	CI_95_upper	*p*_interaction	*q*_FDR_interaction	Age_beta	Age_*p*	Evidence_level
Not-Worrying | EO | 263-381 ms	9	LF	Not-Worrying x Group	0.312	-0.795	1.418	0.571	0.631	0.030	0.181	No association
Not-Worrying | EO | 263-381 ms	9	RF	Not-Worrying x Group	0.425	-0.601	1.450	0.406	0.631	0.035	0.086	No association
Not-Worrying | EO | 263-381 ms	9	LC	Not-Worrying x Group	0.345	-0.706	1.395	0.510	0.631	0.043	0.043	No association
Not-Worrying | EO | 263-381 ms	9	RC	Not-Worrying x Group	0.299	-0.822	1.421	0.591	0.631	0.038	0.091	No association
Not-Worrying | EO | 263-381 ms	9	LPO	Not-Worrying x Group	0.491	-0.541	1.523	0.341	0.631	0.047	0.026	No association
Not-Worrying | EO | 263-381 ms	9	RPO	Not-Worrying x Group	0.292	-0.931	1.514	0.631	0.631	0.039	0.111	No association
Not-Worrying | EO | 263-381 ms	9	CF	Not-Worrying x Group	0.469	-0.766	1.704	0.446	0.631	0.029	0.235	No association
Not-Worrying | EO | 263-381 ms	9	CFP	Not-Worrying x Group	0.594	-0.502	1.690	0.279	0.631	0.033	0.133	No association
Not-Worrying | EO | 263-381 ms	9	CPO	Not-Worrying x Group	0.429	-0.658	1.515	0.429	0.631	0.046	0.037	No association
Emotional Awareness | EC | 263-381 ms	9	LF	Emotional Awareness x Group	-0.034	-0.807	0.738	0.929	0.972	0.010	0.581	No association
Emotional Awareness | EC | 263-381 ms	9	RF	Emotional Awareness x Group	0.108	-0.663	0.879	0.778	0.972	0.023	0.216	No association
Emotional Awareness | EC | 263-381 ms	9	LC	Emotional Awareness x Group	-0.047	-0.923	0.828	0.913	0.972	0.025	0.239	No association
Emotional Awareness | EC | 263-381 ms	9	RC	Emotional Awareness x Group	-0.167	-0.932	0.599	0.661	0.972	0.024	0.197	No association
Emotional Awareness | EC | 263-381 ms	9	LPO	Emotional Awareness x Group	0.086	-0.791	0.963	0.843	0.972	0.030	0.163	No association

FDR_family	FDR_family_size	Region	Interaction_term	Interaction_beta	CI_95_lower	CI_95_upper	*p*_interaction	*q*_FDR_interaction	Age_beta	Age_*p*	Evidence_level
Emotional Awareness | EC | 263-381 ms	9	RPO	Emotional Awareness x Group	0.014	-0.818	0.847	0.972	0.972	0.022	0.290	No association
Emotional Awareness | EC | 263-381 ms	9	CF	Emotional Awareness x Group	-0.089	-0.979	0.800	0.839	0.972	0.031	0.156	No association
Emotional Awareness | EC | 263-381 ms	9	CFP	Emotional Awareness x Group	-0.102	-0.977	0.773	0.814	0.972	0.037	0.091	No association
Emotional Awareness | EC | 263-381 ms	9	CPO	Emotional Awareness x Group	0.172	-0.715	1.058	0.697	0.972	0.035	0.114	No association
Emotional Awareness | EO | 263-381 ms	9	LF	Emotional Awareness x Group	-0.204	-1.104	0.697	0.649	0.966	0.037	0.094	No association
Emotional Awareness | EO | 263-381 ms	9	RF	Emotional Awareness x Group	-0.054	-0.879	0.771	0.894	0.966	0.049	0.019	No association
Emotional Awareness | EO | 263-381 ms	9	LC	Emotional Awareness x Group	-0.114	-0.956	0.728	0.785	0.966	0.055	0.011	No association
Emotional Awareness | EO | 263-381 ms	9	RC	Emotional Awareness x Group	-0.125	-1.015	0.765	0.777	0.966	0.050	0.026	No association
Emotional Awareness | EO | 263-381 ms	9	LPO	Emotional Awareness x Group	-0.045	-0.862	0.773	0.913	0.966	0.061	0.004	No association
Emotional Awareness | EO | 263-381 ms	9	RPO	Emotional Awareness x Group	0.021	-0.967	1.009	0.966	0.966	0.052	0.036	No association
Emotional Awareness | EO | 263-381 ms	9	CF	Emotional Awareness x Group	-0.278	-1.258	0.703	0.569	0.966	0.043	0.076	No association

FDR_family	FDR_family_size	Region	Interaction_term	Interaction_beta	CI_95_lower	CI_95_upper	*p*_interaction	*q*_FDR_interaction	Age_beta	Age_*p*	Evidence_level
Emotional Awareness | EO | 263-381 ms	9	CFP	Emotional Awareness x Group	-0.210	-1.061	0.641	0.619	0.966	0.050	0.021	No association
Emotional Awareness | EO | 263-381 ms	9	CPO	Emotional Awareness x Group	0.033	-0.824	0.891	0.938	0.966	0.061	0.005	No association
Trusting | EC | 263-381 ms	9	LF	Trusting x Group	-0.131	-0.825	0.563	0.704	0.704	0.012	0.557	No association
Trusting | EC | 263-381 ms	9	RF	Trusting x Group	-0.172	-0.852	0.508	0.611	0.687	0.019	0.344	No association
Trusting | EC | 263-381 ms	9	LC	Trusting x Group	-0.270	-1.043	0.504	0.484	0.687	0.019	0.406	No association
Trusting | EC | 263-381 ms	9	RC	Trusting x Group	-0.289	-0.963	0.385	0.390	0.687	0.016	0.422	No association
Trusting | EC | 263-381 ms	9	LPO	Trusting x Group	-0.239	-1.014	0.535	0.534	0.687	0.023	0.327	No association
Trusting | EC | 263-381 ms	9	RPO	Trusting x Group	-0.385	-1.112	0.342	0.290	0.687	0.009	0.663	No association
Trusting | EC | 263-381 ms	9	CF	Trusting x Group	-0.445	-1.218	0.328	0.250	0.687	0.019	0.407	No association
Trusting | EC | 263-381 ms	9	CFP	Trusting x Group	-0.368	-1.136	0.399	0.337	0.687	0.024	0.291	No association
Trusting | EC | 263-381 ms	9	CPO	Trusting x Group	-0.343	-1.123	0.437	0.379	0.687	0.023	0.330	No association
Trusting | EO | 263-381 ms	9	LF	Trusting x Group	-0.166	-0.973	0.640	0.678	0.991	0.029	0.235	No association
Trusting | EO | 263-381 ms	9	RF	Trusting x Group	-0.121	-0.876	0.634	0.746	0.991	0.038	0.103	No association
Trusting | EO | 263-381 ms	9	LC	Trusting x Group	0.052	-0.713	0.818	0.891	0.991	0.049	0.038	No association
Trusting | EO | 263-381 ms	9	RC	Trusting x Group	0.020	-0.795	0.835	0.960	0.991	0.042	0.090	No association

FDR_family	FDR_family_size	Region	Interaction_term	Interaction_beta	CI_95_lower	CI_95_upper	*p*_interaction	*q*_FDR_interaction	Age_beta	Age_*p*	Evidence_level
Trusting | EO | 263-381 ms	9	LPO	Trusting x Group	0.118	-0.640	0.875	0.755	0.991	0.055	0.020	No association
Trusting | EO | 263-381 ms	9	RPO	Trusting x Group	0.060	-0.835	0.955	0.893	0.991	0.046	0.094	No association
Trusting | EO | 263-381 ms	9	CF	Trusting x Group	-0.215	-1.117	0.687	0.631	0.991	0.028	0.299	No association
Trusting | EO | 263-381 ms	9	CFP	Trusting x Group	-0.043	-0.849	0.763	0.914	0.991	0.037	0.129	No association
Trusting | EO | 263-381 ms	9	CPO	Trusting x Group	0.004	-0.792	0.801	0.991	0.991	0.051	0.038	No association

Note. Interaction models included self-report score, group, self-report × group interaction, and age as predictors. The interaction term tested whether the self-report–HEP association differed between PD and HC after controlling for age. FDR correction was applied across the nine ROIs within each self-report construct, resting-state condition, and HEP time window.

Supplementary Table S6

Supplementary Table S6. RBP Pearson correlation results with FDR correction, confidence intervals, and Bayes factors.

FDR_family	FDR_family_size	Region	*r*	CI_95_lower	CI_95_upper	*p*	*q*_FDR	BF_{10}	Evidence_level
BPQ-VSF | HC | EC | delta	9	LF	0.169	-0.284	0.560	0.465	0.965	0.347	No association
BPQ-VSF | HC | EC | delta	9	RF	0.175	-0.277	0.564	0.447	0.965	0.354	No association
BPQ-VSF | HC | EC | delta	9	LC	0.082	-0.363	0.496	0.725	0.965	0.286	No association
BPQ-VSF | HC | EC | delta	9	RC	0.115	-0.333	0.521	0.620	0.965	0.303	No association
BPQ-VSF | HC | EC | delta	9	LPO	0.014	-0.420	0.443	0.951	0.965	0.271	No association
BPQ-VSF | HC | EC | delta	9	RPO	0.076	-0.368	0.491	0.744	0.965	0.284	No association
BPQ-VSF | HC | EC | delta	9	CF	0.169	-0.283	0.560	0.464	0.965	0.348	No association
BPQ-VSF | HC | EC | delta	9	CFP	0.058	-0.383	0.478	0.802	0.965	0.278	No association
BPQ-VSF | HC | EC | delta	9	CPO	-0.010	-0.440	0.423	0.965	0.965	0.271	No association
BPQ-VSF | PD | EC | delta	9	LF	-0.353	-0.696	0.121	0.138	0.241	0.791	No association
BPQ-VSF | PD | EC | delta	9	RF	-0.440	-0.745	0.018	0.060	0.241	1.480	No association
BPQ-VSF | PD | EC | delta	9	LC	-0.357	-0.698	0.116	0.134	0.241	0.810	No association
BPQ-VSF | PD | EC | delta	9	RC	-0.411	-0.729	0.054	0.081	0.241	1.175	No association
BPQ-VSF | PD | EC | delta	9	LPO	-0.289	-0.657	0.190	0.230	0.241	0.555	No association
BPQ-VSF | PD | EC | delta	9	RPO	-0.296	-0.661	0.183	0.219	0.241	0.574	No association
BPQ-VSF | PD | EC | delta	9	CF	-0.388	-0.716	0.080	0.100	0.241	1.000	No association
BPQ-VSF | PD | EC | delta	9	CFP	-0.323	-0.678	0.154	0.177	0.241	0.663	No association
BPQ-VSF | PD | EC | delta	9	CPO	-0.283	-0.653	0.197	0.241	0.241	0.540	No association
BPQ-VSF | HC | EO | delta	9	LF	-0.010	-0.440	0.423	0.964	0.981	0.271	No association
BPQ-VSF | HC | EO | delta	9	RF	0.039	-0.400	0.463	0.867	0.981	0.274	No association
BPQ-VSF | HC | EO | delta	9	LC	0.021	-0.414	0.449	0.927	0.981	0.271	No association
BPQ-VSF | HC | EO | delta	9	RC	0.077	-0.367	0.493	0.739	0.981	0.285	No association
BPQ-VSF | HC | EO | delta	9	LPO	0.052	-0.389	0.473	0.824	0.981	0.277	No association

FDR_family	FDR_family_size	Region	*r*	CI_95_lower	CI_95_upper	*p*	*q*_FDR	BF_{10}	Evidence_level
BPQ-VSF | HC | EO | delta	9	RPO	0.092	-0.354	0.504	0.692	0.981	0.291	No association
BPQ-VSF | HC | EO | delta	9	CF	-0.040	-0.464	0.399	0.863	0.981	0.274	No association
BPQ-VSF | HC | EO | delta	9	CFP	-0.102	-0.511	0.345	0.661	0.981	0.296	No association
BPQ-VSF | HC | EO | delta	9	CPO	-0.006	-0.436	0.427	0.981	0.981	0.270	No association
BPQ-VSF | PD | EO | delta	9	LF	-0.455	-0.754	-0.001	0.050	0.113	1.694	No association
BPQ-VSF | PD | EO | delta	9	RF	-0.526	-0.791	-0.094	0.021	0.113	3.403	Nominal $p < .05$ only
BPQ-VSF | PD | EO | delta	9	LC	-0.430	-0.740	0.030	0.066	0.119	1.368	No association
BPQ-VSF | PD | EO | delta	9	RC	-0.479	-0.767	-0.032	0.038	0.113	2.111	Nominal $p < .05$ only
BPQ-VSF | PD | EO | delta	9	LPO	-0.359	-0.699	0.114	0.131	0.169	0.820	No association
BPQ-VSF | PD | EO | delta	9	RPO	-0.334	-0.685	0.141	0.162	0.182	0.706	No association
BPQ-VSF | PD | EO | delta	9	CF	-0.489	-0.772	-0.044	0.034	0.113	2.315	Nominal $p < .05$ only
BPQ-VSF | PD | EO | delta	9	CFP	-0.404	-0.725	0.062	0.087	0.130	1.116	No association
BPQ-VSF | PD | EO | delta	9	CPO	-0.279	-0.651	0.200	0.247	0.247	0.531	No association
BPQ-VSF | HC | EC | theta	9	LF	-0.128	-0.530	0.322	0.581	0.613	0.312	No association
BPQ-VSF | HC | EC | theta	9	RF	-0.171	-0.561	0.282	0.460	0.613	0.349	No association
BPQ-VSF | HC | EC | theta	9	LC	-0.162	-0.555	0.290	0.482	0.613	0.341	No association
BPQ-VSF | HC | EC | theta	9	RC	-0.272	-0.630	0.181	0.233	0.613	0.526	No association
BPQ-VSF | HC | EC | theta	9	LPO	-0.301	-0.648	0.150	0.185	0.613	0.616	No association
BPQ-VSF | HC | EC | theta	9	RPO	-0.355	-0.682	0.091	0.115	0.613	0.869	No association
BPQ-VSF | HC | EC | theta	9	CF	-0.164	-0.557	0.288	0.476	0.613	0.343	No association
BPQ-VSF | HC | EC | theta	9	CFP	-0.117	-0.522	0.331	0.613	0.613	0.305	No association
BPQ-VSF | HC | EC | theta	9	CPO	-0.204	-0.584	0.250	0.375	0.613	0.391	No association
BPQ-VSF | PD | EC | theta	9	LF	0.423	-0.039	0.736	0.071	0.219	1.290	No association
BPQ-VSF | PD | EC | theta	9	RF	0.460	0.008	0.756	0.047	0.219	1.774	Nominal $p < .05$ only
BPQ-VSF | PD | EC | theta	9	LC	0.298	-0.181	0.663	0.215	0.433	0.581	No association
BPQ-VSF | PD | EC | theta	9	RC	0.228	-0.252	0.618	0.348	0.453	0.428	No association
BPQ-VSF | PD | EC | theta	9	LPO	0.283	-0.197	0.653	0.241	0.433	0.540	No association
BPQ-VSF | PD | EC | theta	9	RPO	0.207	-0.273	0.604	0.395	0.453	0.398	No association

FDR_family	FDR_family_size	Region	*r*	CI_95_lower	CI_95_upper	*p*	*q*_FDR	BF_{10}	Evidence_level
BPQ-VSF | PD | EC | theta	9	CF	0.421	-0.041	0.735	0.073	0.219	1.270	No association
BPQ-VSF | PD | EC | theta	9	CFP	0.204	-0.276	0.602	0.402	0.453	0.394	No association
BPQ-VSF | PD | EC | theta	9	CPO	0.146	-0.330	0.563	0.551	0.551	0.335	No association
BPQ-VSF | HC | EO | theta	9	LF	0.001	-0.431	0.433	0.995	0.995	0.270	No association
BPQ-VSF | HC | EO | theta	9	RF	-0.085	-0.498	0.360	0.714	0.995	0.288	No association
BPQ-VSF | HC | EO | theta	9	LC	0.028	-0.409	0.454	0.906	0.995	0.272	No association
BPQ-VSF | HC | EO | theta	9	RC	-0.112	-0.519	0.336	0.628	0.995	0.302	No association
BPQ-VSF | HC | EO | theta	9	LPO	0.019	-0.416	0.447	0.935	0.995	0.271	No association
BPQ-VSF | HC | EO | theta	9	RPO	-0.044	-0.467	0.395	0.850	0.995	0.275	No association
BPQ-VSF | HC | EO | theta	9	CF	0.048	-0.392	0.470	0.838	0.995	0.276	No association
BPQ-VSF | HC | EO | theta	9	CFP	0.104	-0.343	0.513	0.653	0.995	0.297	No association
BPQ-VSF | HC | EO | theta	9	CPO	0.058	-0.383	0.478	0.802	0.995	0.278	No association
BPQ-VSF | PD | EO | theta	9	LF	0.507	0.069	0.782	0.027	0.069	2.800	Exploratory FDR-level .05 ≤ *q* < .10
BPQ-VSF | PD | EO | theta	9	RF	0.469	0.019	0.761	0.043	0.069	1.919	Exploratory FDR-level .05 ≤ *q* < .10
BPQ-VSF | PD | EO | theta	9	LC	0.462	0.010	0.757	0.046	0.069	1.805	Exploratory FDR-level .05 ≤ *q* < .10
BPQ-VSF | PD | EO | theta	9	RC	0.426	-0.035	0.738	0.069	0.077	1.327	Exploratory FDR-level .05 ≤ *q* < .10
BPQ-VSF | PD | EO | theta	9	LPO	0.449	-0.006	0.750	0.054	0.069	1.606	Exploratory FDR-level .05 ≤ *q* < .10
BPQ-VSF | PD | EO | theta	9	RPO	0.474	0.026	0.764	0.040	0.069	2.016	Exploratory FDR-level .05 ≤ *q* < .10
BPQ-VSF | PD | EO | theta	9	CF	0.457	0.003	0.754	0.049	0.069	1.715	Exploratory FDR-level .05 ≤ *q* < .10
BPQ-VSF | PD | EO | theta	9	CFP	0.392	-0.075	0.718	0.097	0.097	1.027	Exploratory FDR-level .05 ≤ *q* < .10
BPQ-VSF | PD | EO | theta	9	CPO	0.460	0.008	0.756	0.047	0.069	1.771	Exploratory FDR-level .05 ≤ *q* < .10
BPQ-VSF | HC | EC | alpha	9	LF	-0.019	-0.447	0.416	0.935	0.997	0.271	No association

FDR_family	FDR_family_size	Region	*r*	CI_95_lower	CI_95_upper	*p*	*q*_FDR	BF_{10}	Evidence_level
BPQ-VSF | HC | EC | alpha	9	RF	-0.014	-0.443	0.420	0.951	0.997	0.271	No association
BPQ-VSF | HC | EC | alpha	9	LC	0.016	-0.418	0.445	0.945	0.997	0.271	No association
BPQ-VSF | HC | EC | alpha	9	RC	0.005	-0.427	0.436	0.981	0.997	0.270	No association
BPQ-VSF | HC | EC | alpha	9	LPO	0.093	-0.353	0.505	0.688	0.997	0.292	No association
BPQ-VSF | HC | EC | alpha	9	RPO	0.030	-0.407	0.455	0.899	0.997	0.272	No association
BPQ-VSF | HC | EC | alpha	9	CF	-0.001	-0.432	0.431	0.997	0.997	0.270	No association
BPQ-VSF | HC | EC | alpha	9	CFP	0.016	-0.419	0.445	0.945	0.997	0.271	No association
BPQ-VSF | HC | EC | alpha	9	CPO	0.072	-0.371	0.489	0.755	0.997	0.283	No association
BPQ-VSF | PD | EC | alpha	9	LF	0.041	-0.421	0.487	0.866	0.866	0.288	No association
BPQ-VSF | PD | EC | alpha	9	RF	0.125	-0.349	0.548	0.611	0.866	0.320	No association
BPQ-VSF | PD | EC | alpha	9	LC	0.152	-0.325	0.567	0.534	0.866	0.340	No association
BPQ-VSF | PD | EC | alpha	9	RC	0.243	-0.237	0.628	0.316	0.866	0.454	No association
BPQ-VSF | PD | EC | alpha	9	LPO	0.094	-0.377	0.525	0.703	0.866	0.304	No association
BPQ-VSF | PD | EC | alpha	9	RPO	0.159	-0.318	0.572	0.515	0.866	0.346	No association
BPQ-VSF | PD | EC | alpha	9	CF	0.057	-0.408	0.498	0.817	0.866	0.291	No association
BPQ-VSF | PD | EC | alpha	9	CFP	0.144	-0.332	0.561	0.557	0.866	0.333	No association
BPQ-VSF | PD | EC | alpha	9	CPO	0.160	-0.317	0.572	0.513	0.866	0.346	No association
BPQ-VSF | HC | EO | alpha	9	LF	-0.076	-0.491	0.368	0.745	0.978	0.284	No association
BPQ-VSF | HC | EO | alpha	9	RF	-0.075	-0.491	0.368	0.746	0.978	0.284	No association
BPQ-VSF | HC | EO | alpha	9	LC	-0.016	-0.445	0.418	0.944	0.978	0.271	No association
BPQ-VSF | HC | EO | alpha	9	RC	-0.021	-0.448	0.415	0.929	0.978	0.271	No association
BPQ-VSF | HC | EO | alpha	9	LPO	-0.064	-0.483	0.378	0.781	0.978	0.280	No association
BPQ-VSF | HC | EO | alpha	9	RPO	-0.069	-0.486	0.374	0.766	0.978	0.282	No association
BPQ-VSF | HC | EO | alpha	9	CF	0.006	-0.427	0.437	0.978	0.978	0.270	No association
BPQ-VSF | HC | EO | alpha	9	CFP	0.066	-0.377	0.484	0.777	0.978	0.281	No association
BPQ-VSF | HC | EO | alpha	9	CPO	-0.029	-0.455	0.408	0.900	0.978	0.272	No association
BPQ-VSF | PD | EO | alpha	9	LF	0.137	-0.339	0.556	0.577	0.840	0.328	No association
BPQ-VSF | PD | EO | alpha	9	RF	0.286	-0.193	0.655	0.235	0.840	0.548	No association

**FDR_family**	**FDR_family_size**	**Region**	***r***	**CI_95_lower**	**CI_95_upper**	***p***	***q*_FDR**	**BF_{10}**	**Evidence_level**
BPQ-VSF | PD | EO | alpha	9	LC	0.118	-0.355	0.543	0.630	0.840	0.316	No association
BPQ-VSF | PD | EO | alpha	9	RC	0.219	-0.261	0.612	0.367	0.840	0.415	No association
BPQ-VSF | PD | EO | alpha	9	LPO	0.109	-0.364	0.536	0.658	0.840	0.311	No association
BPQ-VSF | PD | EO | alpha	9	RPO	0.098	-0.373	0.528	0.691	0.840	0.305	No association
BPQ-VSF | PD | EO | alpha	9	CF	0.180	-0.299	0.586	0.462	0.840	0.365	No association
BPQ-VSF | PD | EO | alpha	9	CFP	0.079	-0.389	0.515	0.747	0.840	0.298	No association
BPQ-VSF | PD | EO | alpha	9	CPO	0.038	-0.424	0.484	0.878	0.878	0.287	No association
BPQ-VSF | HC | EC | beta	9	LF	-0.271	-0.629	0.182	0.234	0.585	0.525	No association
BPQ-VSF | HC | EC | beta	9	RF	-0.311	-0.655	0.140	0.170	0.585	0.653	No association
BPQ-VSF | HC | EC | beta	9	LC	-0.176	-0.565	0.277	0.445	0.585	0.355	No association
BPQ-VSF | HC | EC | beta	9	RC	-0.193	-0.577	0.260	0.402	0.585	0.376	No association
BPQ-VSF | HC | EC | beta	9	LPO	-0.150	-0.546	0.301	0.517	0.585	0.329	No association
BPQ-VSF | HC | EC | beta	9	RPO	-0.161	-0.554	0.291	0.485	0.585	0.340	No association
BPQ-VSF | HC | EC | beta	9	CF	-0.286	-0.639	0.166	0.209	0.585	0.567	No association
BPQ-VSF | HC | EC | beta	9	CFP	-0.149	-0.545	0.302	0.520	0.585	0.328	No association
BPQ-VSF | HC | EC | beta	9	CPO	-0.080	-0.494	0.365	0.731	0.731	0.286	No association
BPQ-VSF | PD | EC | beta	9	LF	0.426	-0.035	0.737	0.069	0.069	1.321	Exploratory FDR-level .05 ≤ *q* < .10
BPQ-VSF | PD | EC | beta	9	RF	0.510	0.072	0.783	0.026	0.058	2.861	Exploratory FDR-level .05 ≤ *q* < .10
BPQ-VSF | PD | EC | beta	9	LC	0.451	-0.004	0.751	0.053	0.059	1.628	Exploratory FDR-level .05 ≤ *q* < .10
BPQ-VSF | PD | EC | beta	9	RC	0.491	0.048	0.773	0.033	0.058	2.375	Exploratory FDR-level .05 ≤ *q* < .10
BPQ-VSF | PD | EC | beta	9	LPO	0.552	0.131	0.805	0.014	0.058	4.648	Exploratory FDR-level .05 ≤ *q* < .10
BPQ-VSF | PD | EC | beta	9	RPO	0.497	0.056	0.776	0.030	0.058	2.519	Exploratory FDR-level .05 ≤ *q* < .10
BPQ-VSF | PD | EC | beta	9	CF	0.477	0.029	0.765	0.039	0.058	2.070	Exploratory FDR-level .05 ≤ *q* < .10

FDR_family	FDR_family_size	Region	*r*	CI_95_lower	CI_95_upper	*p*	*q*_FDR	BF_{10}	Evidence_level
BPQ-VSF | PD | EC | beta	9	CFP	0.460	0.007	0.756	0.047	0.059	1.769	Exploratory FDR-level .05 ≤ *q* < .10
BPQ-VSF | PD | EC | beta	9	CPO	0.485	0.040	0.770	0.035	0.058	2.234	Exploratory FDR-level .05 ≤ *q* < .10
BPQ-VSF | HC | EO | beta	9	LF	0.043	-0.396	0.466	0.852	0.980	0.275	No association
BPQ-VSF | HC | EO | beta	9	RF	0.006	-0.427	0.437	0.980	0.980	0.270	No association
BPQ-VSF | HC | EO | beta	9	LC	-0.045	-0.467	0.395	0.847	0.980	0.275	No association
BPQ-VSF | HC | EO | beta	9	RC	-0.082	-0.496	0.362	0.723	0.980	0.287	No association
BPQ-VSF | HC | EO | beta	9	LPO	-0.040	-0.463	0.399	0.864	0.980	0.274	No association
BPQ-VSF | HC | EO | beta	9	RPO	-0.088	-0.501	0.357	0.703	0.980	0.289	No association
BPQ-VSF | HC | EO | beta	9	CF	0.031	-0.406	0.457	0.893	0.980	0.273	No association
BPQ-VSF | HC | EO | beta	9	CFP	0.064	-0.378	0.482	0.783	0.980	0.280	No association
BPQ-VSF | HC | EO | beta	9	CPO	0.012	-0.422	0.441	0.960	0.980	0.271	No association
BPQ-VSF | PD | EO | beta	9	LF	0.280	-0.199	0.652	0.245	0.245	0.533	No association
BPQ-VSF | PD | EO | beta	9	RF	0.357	-0.117	0.698	0.134	0.230	0.808	No association
BPQ-VSF | PD | EO | beta	9	LC	0.368	-0.104	0.704	0.121	0.230	0.868	No association
BPQ-VSF | PD | EO | beta	9	RC	0.392	-0.076	0.718	0.097	0.230	1.024	No association
BPQ-VSF | PD | EO | beta	9	LPO	0.362	-0.111	0.701	0.128	0.230	0.835	No association
BPQ-VSF | PD | EO | beta	9	RPO	0.321	-0.156	0.677	0.180	0.230	0.656	No association
BPQ-VSF | PD | EO | beta	9	CF	0.313	-0.164	0.672	0.191	0.230	0.629	No association
BPQ-VSF | PD | EO | beta	9	CFP	0.357	-0.117	0.698	0.134	0.230	0.808	No association
BPQ-VSF | PD | EO | beta	9	CPO	0.305	-0.174	0.667	0.205	0.230	0.601	No association
Not-Distracting | HC | EC | delta	9	LF	0.023	-0.413	0.450	0.921	0.994	0.272	No association
Not-Distracting | HC | EC | delta	9	RF	-0.012	-0.441	0.422	0.960	0.994	0.271	No association
Not-Distracting | HC | EC | delta	9	LC	0.089	-0.356	0.502	0.700	0.994	0.290	No association
Not-Distracting | HC | EC | delta	9	RC	0.033	-0.405	0.458	0.888	0.994	0.273	No association
Not-Distracting | HC | EC | delta	9	LPO	0.103	-0.344	0.512	0.657	0.994	0.297	No association
Not-Distracting | HC | EC | delta	9	RPO	0.070	-0.373	0.487	0.764	0.994	0.282	No association

FDR_family	FDR_family_size	Region	*r*	CI_95_lower	CI_95_upper	*p*	*q*_FDR	BF_{10}	Evidence_level
Not-Distracting | HC | EC | delta	9	CF	0.002	-0.430	0.433	0.994	0.994	0.270	No association
Not-Distracting | HC | EC | delta	9	CFP	0.103	-0.344	0.512	0.656	0.994	0.297	No association
Not-Distracting | HC | EC | delta	9	CPO	0.122	-0.327	0.526	0.599	0.994	0.308	No association
Not-Distracting | PD | EC | delta	9	LF	-0.028	-0.476	0.432	0.910	0.996	0.285	No association
Not-Distracting | PD | EC | delta	9	RF	-0.036	-0.482	0.425	0.883	0.996	0.287	No association
Not-Distracting | PD | EC | delta	9	LC	0.019	-0.439	0.469	0.937	0.996	0.285	No association
Not-Distracting | PD | EC | delta	9	RC	-0.016	-0.467	0.441	0.947	0.996	0.284	No association
Not-Distracting | PD | EC | delta	9	LPO	0.047	-0.416	0.490	0.850	0.996	0.289	No association
Not-Distracting | PD | EC | delta	9	RPO	-0.015	-0.466	0.442	0.952	0.996	0.284	No association
Not-Distracting | PD | EC | delta	9	CF	-0.035	-0.482	0.426	0.886	0.996	0.287	No association
Not-Distracting | PD | EC | delta	9	CFP	0.001	-0.453	0.455	0.996	0.996	0.284	No association
Not-Distracting | PD | EC | delta	9	CPO	0.030	-0.430	0.477	0.904	0.996	0.286	No association
Not-Distracting | HC | EO | delta	9	LF	0.124	-0.325	0.528	0.591	0.789	0.309	No association
Not-Distracting | HC | EO | delta	9	RF	0.086	-0.359	0.499	0.712	0.789	0.288	No association
Not-Distracting | HC | EO | delta	9	LC	0.136	-0.314	0.536	0.557	0.789	0.318	No association
Not-Distracting | HC | EO | delta	9	RC	0.105	-0.343	0.513	0.652	0.789	0.297	No association
Not-Distracting | HC | EO | delta	9	LPO	0.109	-0.339	0.516	0.638	0.789	0.300	No association
Not-Distracting | HC | EO | delta	9	RPO	0.062	-0.380	0.481	0.789	0.789	0.279	No association
Not-Distracting | HC | EO | delta	9	CF	0.135	-0.315	0.535	0.561	0.789	0.317	No association
Not-Distracting | HC | EO | delta	9	CFP	0.226	-0.228	0.599	0.325	0.789	0.426	No association
Not-Distracting | HC | EO | delta	9	CPO	0.108	-0.340	0.515	0.642	0.789	0.299	No association
Not-Distracting | PD | EO | delta	9	LF	-0.187	-0.591	0.292	0.443	0.683	0.374	No association
Not-Distracting | PD | EO | delta	9	RF	-0.131	-0.552	0.344	0.593	0.683	0.324	No association
Not-Distracting | PD | EO | delta	9	LC	-0.136	-0.556	0.339	0.579	0.683	0.328	No association
Not-Distracting | PD | EO | delta	9	RC	-0.171	-0.580	0.307	0.484	0.683	0.357	No association
Not-Distracting | PD | EO | delta	9	LPO	-0.158	-0.571	0.319	0.518	0.683	0.345	No association
Not-Distracting | PD | EO | delta	9	RPO	-0.188	-0.592	0.291	0.440	0.683	0.375	No association
Not-Distracting | PD | EO | delta	9	CF	-0.144	-0.562	0.332	0.556	0.683	0.334	No association

FDR_family	FDR_family_size	Region	*r*	CI_95_lower	CI_95_upper	*p*	*q*_FDR	BF_{10}	Evidence_level
Not-Distracting | PD | EO | delta	9	CFP	-0.097	-0.528	0.373	0.692	0.692	0.305	No association
Not-Distracting | PD | EO | delta	9	CPO	-0.126	-0.549	0.348	0.607	0.683	0.321	No association
Not-Distracting | HC | EC | theta	9	LF	-0.095	-0.506	0.351	0.683	0.989	0.292	No association
Not-Distracting | HC | EC | theta	9	RF	-0.023	-0.450	0.413	0.921	0.989	0.272	No association
Not-Distracting | HC | EC | theta	9	LC	-0.068	-0.486	0.375	0.769	0.989	0.281	No association
Not-Distracting | HC | EC | theta	9	RC	0.012	-0.422	0.442	0.957	0.989	0.271	No association
Not-Distracting | HC | EC | theta	9	LPO	0.057	-0.384	0.477	0.805	0.989	0.278	No association
Not-Distracting | HC | EC | theta	9	RPO	0.078	-0.366	0.493	0.738	0.989	0.285	No association
Not-Distracting | HC | EC | theta	9	CF	-0.061	-0.480	0.381	0.793	0.989	0.279	No association
Not-Distracting | HC | EC | theta	9	CFP	-0.118	-0.523	0.331	0.612	0.989	0.305	No association
Not-Distracting | HC | EC | theta	9	CPO	-0.003	-0.434	0.429	0.989	0.989	0.270	No association
Not-Distracting | PD | EC | theta	9	LF	-0.546	-0.801	-0.122	0.016	0.047	4.294	FDR-corrected $q < .05$
Not-Distracting | PD | EC | theta	9	RF	-0.491	-0.773	-0.047	0.033	0.059	2.357	Exploratory FDR-level $.05 \leq q < .10$
Not-Distracting | PD | EC | theta	9	LC	-0.501	-0.778	-0.061	0.029	0.059	2.618	Exploratory FDR-level $.05 \leq q < .10$
Not-Distracting | PD | EC | theta	9	RC	-0.413	-0.730	0.051	0.079	0.111	1.196	No association
Not-Distracting | PD | EC | theta	9	LPO	-0.272	-0.646	0.208	0.261	0.261	0.512	No association
Not-Distracting | PD | EC | theta	9	RPO	-0.311	-0.671	0.166	0.194	0.219	0.623	No association
Not-Distracting | PD | EC | theta	9	CF	-0.558	-0.808	-0.139	0.013	0.047	4.993	FDR-corrected $q < .05$
Not-Distracting | PD | EC | theta	9	CFP	-0.559	-0.808	-0.141	0.013	0.047	5.059	FDR-corrected $q < .05$
Not-Distracting | PD | EC | theta	9	CPO	-0.404	-0.725	0.061	0.086	0.111	1.119	No association
Not-Distracting | HC | EO | theta	9	LF	-0.146	-0.544	0.305	0.526	0.918	0.326	No association
Not-Distracting | HC | EO | theta	9	RF	-0.024	-0.451	0.412	0.918	0.918	0.272	No association
Not-Distracting | HC | EO | theta	9	LC	-0.177	-0.565	0.276	0.443	0.918	0.356	No association
Not-Distracting | HC | EO | theta	9	RC	-0.046	-0.468	0.393	0.843	0.918	0.275	No association
Not-Distracting | HC | EO | theta	9	LPO	-0.145	-0.543	0.306	0.531	0.918	0.325	No association
Not-Distracting | HC | EO | theta	9	RPO	-0.085	-0.498	0.360	0.714	0.918	0.288	No association

FDR_family	FDR_family_size	Region	*r*	CI_95_lower	CI_95_upper	*p*	*q*_FDR	BF_{10}	Evidence_level
Not-Distracting | HC | EO | theta	9	CF	-0.109	-0.516	0.339	0.638	0.918	0.300	No association
Not-Distracting | HC | EO | theta	9	CFP	-0.174	-0.563	0.279	0.451	0.918	0.353	No association
Not-Distracting | HC | EO | theta	9	CPO	-0.147	-0.544	0.304	0.525	0.918	0.327	No association
Not-Distracting | PD | EO | theta	9	LF	-0.500	-0.778	-0.059	0.029	0.037	2.589	FDR-corrected $q < .05$
Not-Distracting | PD | EO | theta	9	RF	-0.527	-0.792	-0.096	0.020	0.037	3.468	FDR-corrected $q < .05$
Not-Distracting | PD | EO | theta	9	LC	-0.502	-0.779	-0.062	0.029	0.037	2.639	FDR-corrected $q < .05$
Not-Distracting | PD | EO | theta	9	RC	-0.503	-0.779	-0.063	0.028	0.037	2.677	FDR-corrected $q < .05$
Not-Distracting | PD | EO | theta	9	LPO	-0.459	-0.756	-0.006	0.048	0.048	1.750	FDR-corrected $q < .05$
Not-Distracting | PD | EO | theta	9	RPO	-0.493	-0.774	-0.050	0.032	0.037	2.413	FDR-corrected $q < .05$
Not-Distracting | PD | EO | theta	9	CF	-0.550	-0.803	-0.127	0.015	0.037	4.495	FDR-corrected $q < .05$
Not-Distracting | PD | EO | theta	9	CFP	-0.560	-0.808	-0.141	0.013	0.037	5.081	FDR-corrected $q < .05$
Not-Distracting | PD | EO | theta	9	CPO	-0.491	-0.773	-0.048	0.033	0.037	2.371	FDR-corrected $q < .05$
Not-Distracting | HC | EC | alpha	9	LF	-0.038	-0.462	0.401	0.872	0.932	0.274	No association
Not-Distracting | HC | EC | alpha	9	RF	-0.040	-0.463	0.399	0.864	0.932	0.274	No association
Not-Distracting | HC | EC | alpha	9	LC	-0.124	-0.527	0.325	0.592	0.932	0.309	No association
Not-Distracting | HC | EC | alpha	9	RC	-0.074	-0.490	0.369	0.749	0.932	0.284	No association
Not-Distracting | HC | EC | alpha	9	LPO	-0.144	-0.542	0.307	0.535	0.932	0.324	No association
Not-Distracting | HC | EC | alpha	9	RPO	-0.125	-0.528	0.324	0.590	0.932	0.310	No association
Not-Distracting | HC | EC | alpha	9	CF	-0.020	-0.448	0.416	0.932	0.932	0.271	No association
Not-Distracting | HC | EC | alpha	9	CFP	-0.084	-0.497	0.361	0.719	0.932	0.287	No association
Not-Distracting | HC | EC | alpha	9	CPO	-0.151	-0.547	0.301	0.515	0.932	0.330	No association
Not-Distracting | PD | EC | alpha	9	LF	-0.079	-0.515	0.389	0.749	0.990	0.298	No association
Not-Distracting | PD | EC | alpha	9	RF	0.003	-0.452	0.457	0.990	0.990	0.284	No association
Not-Distracting | PD | EC | alpha	9	LC	-0.084	-0.519	0.385	0.732	0.990	0.300	No association
Not-Distracting | PD | EC | alpha	9	RC	-0.025	-0.474	0.434	0.918	0.990	0.285	No association
Not-Distracting | PD | EC | alpha	9	LPO	-0.075	-0.512	0.392	0.760	0.990	0.296	No association
Not-Distracting | PD | EC | alpha	9	RPO	0.015	-0.443	0.466	0.953	0.990	0.284	No association
Not-Distracting | PD | EC | alpha	9	CF	0.015	-0.442	0.466	0.950	0.990	0.284	No association

FDR_family	FDR_family_size	Region	*r*	CI_95_lower	CI_95_upper	*p*	*q*_FDR	BF_{10}	Evidence_level
Not-Distracting | PD | EC | alpha	9	CFP	0.011	-0.445	0.463	0.964	0.990	0.284	No association
Not-Distracting | PD | EC | alpha	9	CPO	-0.030	-0.478	0.430	0.902	0.990	0.286	No association
Not-Distracting | HC | EO | alpha	9	LF	-0.092	-0.504	0.353	0.691	0.928	0.291	No association
Not-Distracting | HC | EO | alpha	9	RF	-0.074	-0.490	0.369	0.750	0.928	0.284	No association
Not-Distracting | HC | EO | alpha	9	LC	-0.208	-0.587	0.246	0.366	0.928	0.396	No association
Not-Distracting | HC | EO | alpha	9	RC	-0.152	-0.548	0.300	0.511	0.928	0.331	No association
Not-Distracting | HC | EO | alpha	9	LPO	-0.037	-0.461	0.401	0.873	0.928	0.274	No association
Not-Distracting | HC | EO | alpha	9	RPO	-0.021	-0.449	0.414	0.928	0.928	0.271	No association
Not-Distracting | HC | EO | alpha	9	CF	-0.058	-0.477	0.384	0.804	0.928	0.278	No association
Not-Distracting | HC | EO | alpha	9	CFP	-0.169	-0.560	0.283	0.464	0.928	0.347	No association
Not-Distracting | HC | EO | alpha	9	CPO	-0.032	-0.457	0.405	0.891	0.928	0.273	No association
Not-Distracting | PD | EO | alpha	9	LF	-0.293	-0.660	0.186	0.223	0.809	0.567	No association
Not-Distracting | PD | EO | alpha	9	RF	-0.258	-0.638	0.222	0.286	0.809	0.483	No association
Not-Distracting | PD | EO | alpha	9	LC	-0.179	-0.586	0.300	0.464	0.809	0.365	No association
Not-Distracting | PD | EO | alpha	9	RC	-0.150	-0.566	0.326	0.539	0.809	0.338	No association
Not-Distracting | PD | EO | alpha	9	LPO	-0.008	-0.461	0.447	0.973	0.982	0.284	No association
Not-Distracting | PD | EO | alpha	9	RPO	0.006	-0.450	0.459	0.982	0.982	0.284	No association
Not-Distracting | PD | EO | alpha	9	CF	-0.253	-0.634	0.227	0.296	0.809	0.473	No association
Not-Distracting | PD | EO | alpha	9	CFP	-0.218	-0.611	0.262	0.371	0.809	0.413	No association
Not-Distracting | PD | EO | alpha	9	CPO	-0.039	-0.485	0.423	0.874	0.982	0.287	No association
Not-Distracting | HC | EC | beta	9	LF	0.047	-0.393	0.469	0.840	0.912	0.276	No association
Not-Distracting | HC | EC | beta	9	RF	0.098	-0.348	0.508	0.673	0.912	0.294	No association
Not-Distracting | HC | EC | beta	9	LC	0.032	-0.405	0.457	0.890	0.912	0.273	No association
Not-Distracting | HC | EC | beta	9	RC	0.049	-0.391	0.471	0.834	0.912	0.276	No association
Not-Distracting | HC | EC | beta	9	LPO	0.030	-0.407	0.456	0.897	0.912	0.272	No association
Not-Distracting | HC | EC | beta	9	RPO	0.074	-0.369	0.490	0.750	0.912	0.284	No association
Not-Distracting | HC | EC | beta	9	CF	0.061	-0.381	0.480	0.794	0.912	0.279	No association
Not-Distracting | HC | EC | beta	9	CFP	-0.038	-0.462	0.401	0.872	0.912	0.274	No association

FDR_family	FDR_family_size	Region	*r*	CI_95_lower	CI_95_upper	*p*	*q*_FDR	BF_{10}	Evidence_level
Not-Distracting | HC | EC | beta	9	CPO	0.026	-0.411	0.452	0.912	0.912	0.272	No association
Not-Distracting | PD | EC | beta	9	LF	0.377	-0.093	0.710	0.112	0.370	0.923	No association
Not-Distracting | PD | EC | beta	9	RF	0.318	-0.159	0.675	0.185	0.370	0.644	No association
Not-Distracting | PD | EC | beta	9	LC	0.304	-0.174	0.666	0.206	0.370	0.599	No association
Not-Distracting | PD | EC | beta	9	RC	0.331	-0.145	0.682	0.167	0.370	0.692	No association
Not-Distracting | PD | EC | beta	9	LPO	0.189	-0.290	0.592	0.439	0.439	0.375	No association
Not-Distracting | PD | EC | beta	9	RPO	0.213	-0.267	0.608	0.382	0.439	0.406	No association
Not-Distracting | PD | EC | beta	9	CF	0.330	-0.147	0.682	0.168	0.370	0.688	No association
Not-Distracting | PD | EC | beta	9	CFP	0.276	-0.204	0.649	0.253	0.379	0.522	No association
Not-Distracting | PD | EC | beta	9	CPO	0.202	-0.278	0.601	0.407	0.439	0.391	No association
Not-Distracting | HC | EO | beta	9	LF	-0.090	-0.502	0.355	0.698	0.964	0.290	No association
Not-Distracting | HC | EO | beta	9	RF	-0.096	-0.506	0.350	0.680	0.964	0.293	No association
Not-Distracting | HC | EO | beta	9	LC	0.001	-0.431	0.432	0.998	0.998	0.270	No association
Not-Distracting | HC | EO | beta	9	RC	-0.042	-0.465	0.397	0.857	0.964	0.274	No association
Not-Distracting | HC | EO | beta	9	LPO	-0.111	-0.518	0.337	0.632	0.964	0.301	No association
Not-Distracting | HC | EO | beta	9	RPO	-0.072	-0.489	0.371	0.756	0.964	0.283	No association
Not-Distracting | HC | EO | beta	9	CF	-0.139	-0.538	0.311	0.548	0.964	0.320	No association
Not-Distracting | HC | EO | beta	9	CFP	-0.181	-0.568	0.272	0.432	0.964	0.361	No association
Not-Distracting | HC | EO | beta	9	CPO	-0.119	-0.524	0.329	0.607	0.964	0.306	No association
Not-Distracting | PD | EO | beta	9	LF	0.491	0.047	0.773	0.033	0.051	2.362	Exploratory FDR-level .05 ≤ *q* < .10
Not-Distracting | PD | EO | beta	9	RF	0.476	0.027	0.765	0.040	0.051	2.040	Exploratory FDR-level .05 ≤ *q* < .10
Not-Distracting | PD | EO | beta	9	LC	0.451	-0.004	0.751	0.053	0.053	1.630	Exploratory FDR-level .05 ≤ *q* < .10
Not-Distracting | PD | EO | beta	9	RC	0.500	0.059	0.777	0.029	0.051	2.584	Exploratory FDR-level .05 ≤ *q* < .10
Not-Distracting | PD | EO | beta	9	LPO	0.466	0.015	0.759	0.044	0.051	1.863	Exploratory FDR-level .05 ≤ *q* < .10

FDR_family	FDR_family_size	Region	*r*	CI_95_lower	CI_95_upper	*p*	*q*_FDR	BF_{10}	Evidence_level
Not-Distracting | PD | EO | beta	9	RPO	0.527	0.095	0.791	0.021	0.051	3.442	Exploratory FDR-level .05 ≤ *q* < .10
Not-Distracting | PD | EO | beta	9	CF	0.496	0.054	0.776	0.031	0.051	2.492	Exploratory FDR-level .05 ≤ *q* < .10
Not-Distracting | PD | EO | beta	9	CFP	0.464	0.012	0.758	0.046	0.051	1.824	Exploratory FDR-level .05 ≤ *q* < .10
Not-Distracting | PD | EO | beta	9	CPO	0.472	0.023	0.763	0.041	0.051	1.973	Exploratory FDR-level .05 ≤ *q* < .10
Not-Worrying | HC | EC | delta	9	LF	-0.202	-0.583	0.252	0.381	0.824	0.387	No association
Not-Worrying | HC | EC | delta	9	RF	-0.102	-0.511	0.345	0.661	0.824	0.296	No association
Not-Worrying | HC | EC | delta	9	LC	-0.148	-0.545	0.303	0.521	0.824	0.328	No association
Not-Worrying | HC | EC | delta	9	RC	-0.123	-0.527	0.326	0.594	0.824	0.309	No association
Not-Worrying | HC | EC | delta	9	LPO	-0.136	-0.536	0.314	0.556	0.824	0.318	No association
Not-Worrying | HC | EC | delta	9	RPO	-0.149	-0.546	0.302	0.519	0.824	0.329	No association
Not-Worrying | HC | EC | delta	9	CF	-0.041	-0.464	0.398	0.861	0.861	0.274	No association
Not-Worrying | HC | EC | delta	9	CFP	-0.079	-0.494	0.365	0.733	0.824	0.285	No association
Not-Worrying | HC | EC | delta	9	CPO	-0.113	-0.519	0.335	0.627	0.824	0.302	No association
Not-Worrying | PD | EC | delta	9	LF	0.017	-0.440	0.468	0.944	0.992	0.284	No association
Not-Worrying | PD | EC | delta	9	RF	0.025	-0.435	0.473	0.921	0.992	0.285	No association
Not-Worrying | PD | EC | delta	9	LC	0.007	-0.448	0.460	0.977	0.992	0.284	No association
Not-Worrying | PD | EC | delta	9	RC	0.054	-0.410	0.496	0.826	0.992	0.290	No association
Not-Worrying | PD | EC | delta	9	LPO	-0.117	-0.542	0.356	0.634	0.992	0.315	No association
Not-Worrying | PD | EC | delta	9	RPO	-0.130	-0.552	0.345	0.596	0.992	0.324	No association
Not-Worrying | PD | EC | delta	9	CF	0.003	-0.452	0.456	0.992	0.992	0.284	No association
Not-Worrying | PD | EC | delta	9	CFP	0.047	-0.416	0.491	0.849	0.992	0.289	No association
Not-Worrying | PD | EC | delta	9	CPO	-0.095	-0.526	0.376	0.700	0.992	0.304	No association
Not-Worrying | HC | EO | delta	9	LF	-0.124	-0.527	0.325	0.593	0.787	0.309	No association
Not-Worrying | HC | EO | delta	9	RF	-0.147	-0.544	0.304	0.525	0.787	0.327	No association
Not-Worrying | HC | EO | delta	9	LC	-0.133	-0.534	0.317	0.565	0.787	0.316	No association

FDR_family	FDR_family_size	Region	*r*	CI_95_lower	CI_95_upper	*p*	*q*_FDR	BF_{10}	Evidence_level
Not-Worrying | HC | EO | delta	9	RC	-0.168	-0.559	0.284	0.465	0.787	0.347	No association
Not-Worrying | HC | EO | delta	9	LPO	-0.172	-0.562	0.281	0.457	0.787	0.351	No association
Not-Worrying | HC | EO | delta	9	RPO	-0.175	-0.564	0.278	0.448	0.787	0.354	No association
Not-Worrying | HC | EO | delta	9	CF	0.011	-0.423	0.440	0.963	0.963	0.271	No association
Not-Worrying | HC | EO | delta	9	CFP	-0.027	-0.453	0.410	0.908	0.963	0.272	No association
Not-Worrying | HC | EO | delta	9	CPO	-0.117	-0.523	0.331	0.612	0.787	0.305	No association
Not-Worrying | PD | EO | delta	9	LF	-0.116	-0.542	0.357	0.635	0.770	0.315	No association
Not-Worrying | PD | EO | delta	9	RF	-0.062	-0.502	0.403	0.800	0.800	0.292	No association
Not-Worrying | PD | EO | delta	9	LC	-0.148	-0.564	0.329	0.546	0.770	0.336	No association
Not-Worrying | PD | EO | delta	9	RC	-0.108	-0.536	0.364	0.659	0.770	0.311	No association
Not-Worrying | PD | EO | delta	9	LPO	-0.151	-0.566	0.326	0.537	0.770	0.339	No association
Not-Worrying | PD | EO | delta	9	RPO	-0.155	-0.569	0.322	0.528	0.770	0.342	No association
Not-Worrying | PD | EO | delta	9	CF	-0.100	-0.530	0.371	0.683	0.770	0.307	No association
Not-Worrying | PD | EO | delta	9	CFP	-0.100	-0.530	0.371	0.684	0.770	0.307	No association
Not-Worrying | PD | EO | delta	9	CPO	-0.174	-0.582	0.304	0.476	0.770	0.360	No association
Not-Worrying | HC | EC | theta	9	LF	0.393	-0.046	0.705	0.078	0.205	1.161	No association
Not-Worrying | HC | EC | theta	9	RF	0.306	-0.145	0.651	0.178	0.229	0.633	No association
Not-Worrying | HC | EC | theta	9	LC	0.358	-0.087	0.684	0.111	0.205	0.888	No association
Not-Worrying | HC | EC | theta	9	RC	0.357	-0.088	0.684	0.112	0.205	0.884	No association
Not-Worrying | HC | EC | theta	9	LPO	0.279	-0.173	0.634	0.220	0.248	0.547	No association
Not-Worrying | HC | EC | theta	9	RPO	0.355	-0.090	0.682	0.114	0.205	0.873	No association
Not-Worrying | HC | EC | theta	9	CF	0.245	-0.209	0.612	0.284	0.284	0.463	No association
Not-Worrying | HC | EC | theta	9	CFP	0.314	-0.136	0.657	0.166	0.229	0.665	No association
Not-Worrying | HC | EC | theta	9	CPO	0.392	-0.048	0.704	0.079	0.205	1.149	No association
Not-Worrying | PD | EC | theta	9	LF	0.134	-0.341	0.555	0.583	0.583	0.327	No association
Not-Worrying | PD | EC | theta	9	RF	0.173	-0.305	0.582	0.478	0.583	0.359	No association
Not-Worrying | PD | EC | theta	9	LC	0.248	-0.233	0.631	0.306	0.551	0.462	No association
Not-Worrying | PD | EC | theta	9	RC	0.334	-0.142	0.684	0.162	0.551	0.706	No association

FDR_family	FDR_family_size	Region	*r*	CI_95_lower	CI_95_upper	*p*	*q*_FDR	BF_{10}	Evidence_level
Not-Worrying | PD | EC | theta	9	LPO	0.261	-0.220	0.639	0.281	0.551	0.488	No association
Not-Worrying | PD | EC | theta	9	RPO	0.209	-0.271	0.606	0.390	0.583	0.401	No association
Not-Worrying | PD | EC | theta	9	CF	0.151	-0.325	0.567	0.536	0.583	0.339	No association
Not-Worrying | PD | EC | theta	9	CFP	0.285	-0.195	0.654	0.237	0.551	0.545	No association
Not-Worrying | PD | EC | theta	9	CPO	0.307	-0.171	0.668	0.202	0.551	0.607	No association
Not-Worrying | HC | EO | theta	9	LF	0.270	-0.183	0.628	0.237	0.467	0.520	No association
Not-Worrying | HC | EO | theta	9	RF	0.188	-0.266	0.573	0.415	0.467	0.369	No association
Not-Worrying | HC | EO | theta	9	LC	0.322	-0.127	0.662	0.154	0.467	0.701	No association
Not-Worrying | HC | EO | theta	9	RC	0.298	-0.154	0.646	0.190	0.467	0.604	No association
Not-Worrying | HC | EO | theta	9	LPO	0.240	-0.213	0.609	0.294	0.467	0.453	No association
Not-Worrying | HC | EO | theta	9	RPO	0.248	-0.206	0.614	0.279	0.467	0.468	No association
Not-Worrying | HC | EO | theta	9	CF	0.103	-0.344	0.512	0.656	0.656	0.297	No association
Not-Worrying | HC | EO | theta	9	CFP	0.212	-0.242	0.590	0.356	0.467	0.403	No association
Not-Worrying | HC | EO | theta	9	CPO	0.207	-0.247	0.586	0.367	0.467	0.396	No association
Not-Worrying | PD | EO | theta	9	LF	-0.174	-0.582	0.304	0.477	0.899	0.360	No association
Not-Worrying | PD | EO | theta	9	RF	-0.098	-0.529	0.373	0.690	0.899	0.306	No association
Not-Worrying | PD | EO | theta	9	LC	-0.036	-0.482	0.425	0.884	0.899	0.287	No association
Not-Worrying | PD | EO | theta	9	RC	0.038	-0.424	0.484	0.877	0.899	0.287	No association
Not-Worrying | PD | EO | theta	9	LPO	0.117	-0.356	0.542	0.634	0.899	0.315	No association
Not-Worrying | PD | EO | theta	9	RPO	0.052	-0.412	0.495	0.832	0.899	0.290	No association
Not-Worrying | PD | EO | theta	9	CF	-0.121	-0.545	0.352	0.621	0.899	0.318	No association
Not-Worrying | PD | EO | theta	9	CFP	0.031	-0.429	0.479	0.899	0.899	0.286	No association
Not-Worrying | PD | EO | theta	9	CPO	0.123	-0.351	0.547	0.616	0.899	0.319	No association
Not-Worrying | HC | EC | alpha	9	LF	0.034	-0.404	0.459	0.885	0.971	0.273	No association
Not-Worrying | HC | EC | alpha	9	RF	-0.061	-0.480	0.381	0.793	0.971	0.279	No association
Not-Worrying | HC | EC | alpha	9	LC	-0.008	-0.439	0.425	0.971	0.971	0.270	No association
Not-Worrying | HC | EC | alpha	9	RC	-0.043	-0.466	0.396	0.852	0.971	0.275	No association
Not-Worrying | HC | EC | alpha	9	LPO	-0.019	-0.447	0.416	0.936	0.971	0.271	No association

FDR_family	FDR_family_size	Region	*r*	CI_95_lower	CI_95_upper	*p*	*q*_FDR	BF_{10}	Evidence_level
Not-Worrying | HC | EC | alpha	9	RPO	-0.010	-0.440	0.424	0.967	0.971	0.270	No association
Not-Worrying | HC | EC | alpha	9	CF	-0.083	-0.497	0.362	0.721	0.971	0.287	No association
Not-Worrying | HC | EC | alpha	9	CFP	-0.045	-0.468	0.394	0.846	0.971	0.275	No association
Not-Worrying | HC | EC | alpha	9	CPO	-0.055	-0.476	0.386	0.812	0.971	0.278	No association
Not-Worrying | PD | EC | alpha	9	LF	-0.119	-0.544	0.355	0.629	0.797	0.317	No association
Not-Worrying | PD | EC | alpha	9	RF	-0.114	-0.540	0.359	0.642	0.797	0.314	No association
Not-Worrying | PD | EC | alpha	9	LC	-0.079	-0.515	0.389	0.747	0.797	0.298	No association
Not-Worrying | PD | EC | alpha	9	RC	-0.171	-0.580	0.307	0.483	0.797	0.357	No association
Not-Worrying | PD | EC | alpha	9	LPO	0.115	-0.358	0.541	0.639	0.797	0.314	No association
Not-Worrying | PD | EC | alpha	9	RPO	0.126	-0.348	0.549	0.607	0.797	0.321	No association
Not-Worrying | PD | EC | alpha	9	CF	-0.063	-0.503	0.402	0.797	0.797	0.293	No association
Not-Worrying | PD | EC | alpha	9	CFP	-0.111	-0.538	0.362	0.652	0.797	0.312	No association
Not-Worrying | PD | EC | alpha	9	CPO	0.079	-0.389	0.515	0.748	0.797	0.298	No association
Not-Worrying | HC | EO | alpha	9	LF	0.128	-0.322	0.530	0.581	0.961	0.312	No association
Not-Worrying | HC | EO | alpha	9	RF	0.066	-0.377	0.484	0.777	0.961	0.281	No association
Not-Worrying | HC | EO | alpha	9	LC	0.072	-0.371	0.488	0.757	0.961	0.283	No association
Not-Worrying | HC | EO | alpha	9	RC	0.073	-0.370	0.490	0.752	0.961	0.283	No association
Not-Worrying | HC | EO | alpha	9	LPO	0.100	-0.346	0.510	0.665	0.961	0.295	No association
Not-Worrying | HC | EO | alpha	9	RPO	0.093	-0.353	0.504	0.690	0.961	0.291	No association
Not-Worrying | HC | EO | alpha	9	CF	0.011	-0.422	0.441	0.961	0.961	0.271	No association
Not-Worrying | HC | EO | alpha	9	CFP	-0.014	-0.443	0.420	0.950	0.961	0.271	No association
Not-Worrying | HC | EO | alpha	9	CPO	0.059	-0.383	0.478	0.801	0.961	0.278	No association
Not-Worrying | PD | EO | alpha	9	LF	-0.036	-0.483	0.425	0.882	0.975	0.287	No association
Not-Worrying | PD | EO | alpha	9	RF	-0.125	-0.548	0.349	0.611	0.975	0.320	No association
Not-Worrying | PD | EO | alpha	9	LC	0.033	-0.428	0.480	0.894	0.975	0.286	No association
Not-Worrying | PD | EO | alpha	9	RC	-0.049	-0.492	0.414	0.842	0.975	0.289	No association
Not-Worrying | PD | EO | alpha	9	LPO	0.008	-0.448	0.460	0.975	0.975	0.284	No association
Not-Worrying | PD | EO | alpha	9	RPO	0.035	-0.426	0.481	0.888	0.975	0.286	No association

FDR_family	FDR_family_size	Region	*r*	CI_95_lower	CI_95_upper	*p*	*q*_FDR	BF_{10}	Evidence_level
Not-Worrying | PD | EO | alpha	9	CF	-0.120	-0.544	0.354	0.626	0.975	0.317	No association
Not-Worrying | PD | EO | alpha	9	CFP	-0.062	-0.502	0.403	0.799	0.975	0.292	No association
Not-Worrying | PD | EO | alpha	9	CPO	0.054	-0.410	0.496	0.825	0.975	0.290	No association
Not-Worrying | HC | EC | beta	9	LF	0.167	-0.285	0.559	0.468	0.608	0.346	No association
Not-Worrying | HC | EC | beta	9	RF	0.145	-0.306	0.543	0.531	0.608	0.325	No association
Not-Worrying | HC | EC | beta	9	LC	0.226	-0.228	0.599	0.324	0.584	0.426	No association
Not-Worrying | HC | EC | beta	9	RC	0.233	-0.221	0.604	0.310	0.584	0.438	No association
Not-Worrying | HC | EC | beta	9	LPO	0.351	-0.095	0.680	0.118	0.369	0.849	No association
Not-Worrying | HC | EC | beta	9	RPO	0.347	-0.099	0.677	0.123	0.369	0.824	No association
Not-Worrying | HC | EC | beta	9	CF	0.048	-0.392	0.470	0.835	0.835	0.276	No association
Not-Worrying | HC | EC | beta	9	CFP	0.142	-0.309	0.540	0.541	0.608	0.322	No association
Not-Worrying | HC | EC | beta	9	CPO	0.361	-0.084	0.686	0.108	0.369	0.909	No association
Not-Worrying | PD | EC | beta	9	LF	0.054	-0.410	0.496	0.826	0.985	0.290	No association
Not-Worrying | PD | EC | beta	9	RF	0.014	-0.443	0.465	0.956	0.985	0.284	No association
Not-Worrying | PD | EC | beta	9	LC	0.038	-0.424	0.484	0.879	0.985	0.287	No association
Not-Worrying | PD | EC | beta	9	RC	0.052	-0.412	0.495	0.832	0.985	0.290	No association
Not-Worrying | PD | EC | beta	9	LPO	-0.056	-0.498	0.409	0.820	0.985	0.291	No association
Not-Worrying | PD | EC | beta	9	RPO	-0.094	-0.525	0.377	0.703	0.985	0.304	No association
Not-Worrying | PD | EC | beta	9	CF	0.005	-0.450	0.458	0.985	0.985	0.284	No association
Not-Worrying | PD | EC | beta	9	CFP	-0.022	-0.472	0.436	0.928	0.985	0.285	No association
Not-Worrying | PD | EC | beta	9	CPO	-0.055	-0.497	0.409	0.823	0.985	0.290	No association
Not-Worrying | HC | EO | beta	9	LF	0.023	-0.413	0.450	0.922	0.925	0.271	No association
Not-Worrying | HC | EO | beta	9	RF	0.131	-0.319	0.533	0.571	0.925	0.314	No association
Not-Worrying | HC | EO | beta	9	LC	0.022	-0.414	0.449	0.925	0.925	0.271	No association
Not-Worrying | HC | EO | beta	9	RC	0.104	-0.343	0.513	0.653	0.925	0.297	No association
Not-Worrying | HC | EO | beta	9	LPO	0.103	-0.344	0.512	0.656	0.925	0.297	No association
Not-Worrying | HC | EO | beta	9	RPO	0.129	-0.321	0.531	0.579	0.925	0.312	No association
Not-Worrying | HC | EO | beta	9	CF	-0.080	-0.494	0.365	0.732	0.925	0.286	No association

FDR_family	FDR_family_size	Region	*r*	CI_95_lower	CI_95_upper	*p*	*q*_FDR	BF_{10}	Evidence_level
Not-Worrying | HC | EO | beta	9	CFP	-0.058	-0.477	0.384	0.804	0.925	0.278	No association
Not-Worrying | HC | EO | beta	9	CPO	0.059	-0.383	0.478	0.801	0.925	0.278	No association
Not-Worrying | PD | EO | beta	9	LF	0.208	-0.271	0.605	0.392	0.501	0.400	No association
Not-Worrying | PD | EO | beta	9	RF	0.164	-0.313	0.576	0.501	0.501	0.350	No association
Not-Worrying | PD | EO | beta	9	LC	0.193	-0.286	0.595	0.428	0.501	0.380	No association
Not-Worrying | PD | EO | beta	9	RC	0.174	-0.305	0.582	0.477	0.501	0.359	No association
Not-Worrying | PD | EO | beta	9	LPO	0.224	-0.256	0.616	0.357	0.501	0.422	No association
Not-Worrying | PD | EO | beta	9	RPO	0.221	-0.259	0.614	0.362	0.501	0.418	No association
Not-Worrying | PD | EO | beta	9	CF	0.215	-0.265	0.610	0.377	0.501	0.409	No association
Not-Worrying | PD | EO | beta	9	CFP	0.168	-0.310	0.578	0.492	0.501	0.354	No association
Not-Worrying | PD | EO | beta	9	CPO	0.217	-0.263	0.611	0.371	0.501	0.412	No association
Emotional Awareness | HC | EC | delta	9	LF	-0.068	-0.485	0.375	0.771	0.976	0.281	No association
Emotional Awareness | HC | EC | delta	9	RF	-0.081	-0.495	0.364	0.728	0.976	0.286	No association
Emotional Awareness | HC | EC | delta	9	LC	-0.042	-0.465	0.397	0.856	0.976	0.274	No association
Emotional Awareness | HC | EC | delta	9	RC	-0.007	-0.437	0.426	0.976	0.976	0.270	No association
Emotional Awareness | HC | EC | delta	9	LPO	-0.066	-0.484	0.377	0.777	0.976	0.281	No association
Emotional Awareness | HC | EC | delta	9	RPO	-0.042	-0.465	0.397	0.856	0.976	0.274	No association
Emotional Awareness | HC | EC | delta	9	CF	-0.091	-0.503	0.355	0.694	0.976	0.291	No association
Emotional Awareness | HC | EC | delta	9	CFP	-0.031	-0.457	0.406	0.894	0.976	0.273	No association
Emotional Awareness | HC | EC | delta	9	CPO	-0.080	-0.495	0.364	0.730	0.976	0.286	No association
Emotional Awareness | PD | EC | delta	9	LF	-0.261	-0.639	0.219	0.281	0.378	0.489	No association

FDR_family	FDR_family_size	Region	*r*	CI_95_lower	CI_95_upper	*p*	*q*_FDR	BF_{10}	Evidence_level
Emotional Awareness | PD | EC | delta	9	RF	-0.218	-0.612	0.262	0.370	0.378	0.413	No association
Emotional Awareness | PD | EC | delta	9	LC	-0.327	-0.680	0.149	0.172	0.378	0.678	No association
Emotional Awareness | PD | EC | delta	9	RC	-0.274	-0.648	0.206	0.257	0.378	0.517	No association
Emotional Awareness | PD | EC | delta	9	LPO	-0.279	-0.651	0.201	0.248	0.378	0.530	No association
Emotional Awareness | PD | EC | delta	9	RPO	-0.270	-0.645	0.210	0.263	0.378	0.509	No association
Emotional Awareness | PD | EC | delta	9	CF	-0.214	-0.609	0.266	0.378	0.378	0.408	No association
Emotional Awareness | PD | EC | delta	9	CFP	-0.314	-0.672	0.163	0.190	0.378	0.632	No association
Emotional Awareness | PD | EC | delta	9	CPO	-0.245	-0.629	0.236	0.312	0.378	0.457	No association
Emotional Awareness | HC | EO | delta	9	LF	-0.207	-0.586	0.246	0.367	0.683	0.396	No association
Emotional Awareness | HC | EO | delta	9	RF	-0.119	-0.524	0.329	0.607	0.683	0.306	No association
Emotional Awareness | HC | EO | delta	9	LC	-0.176	-0.565	0.277	0.445	0.683	0.355	No association
Emotional Awareness | HC | EO | delta	9	RC	-0.092	-0.504	0.353	0.691	0.691	0.291	No association
Emotional Awareness | HC | EO | delta	9	LPO	-0.168	-0.559	0.284	0.467	0.683	0.346	No association
Emotional Awareness | HC | EO | delta	9	RPO	-0.141	-0.540	0.310	0.542	0.683	0.322	No association
Emotional Awareness | HC | EO | delta	9	CF	-0.256	-0.619	0.198	0.263	0.683	0.486	No association
Emotional Awareness | HC | EO | delta	9	CFP	-0.211	-0.589	0.243	0.358	0.683	0.402	No association
Emotional Awareness | HC | EO | delta	9	CPO	-0.215	-0.592	0.239	0.350	0.683	0.407	No association

FDR_family	FDR_family_size	Region	*r*	CI_95_lower	CI_95_upper	*p*	*q*_FDR	BF_{10}	Evidence_level
Emotional Awareness | PD | EO | delta	9	LF	-0.137	-0.556	0.338	0.577	0.956	0.328	No association
Emotional Awareness | PD | EO | delta	9	RF	-0.193	-0.595	0.286	0.429	0.956	0.380	No association
Emotional Awareness | PD | EO | delta	9	LC	-0.116	-0.541	0.357	0.637	0.956	0.315	No association
Emotional Awareness | PD | EO | delta	9	RC	-0.173	-0.581	0.306	0.480	0.956	0.358	No association
Emotional Awareness | PD | EO | delta	9	LPO	-0.013	-0.464	0.444	0.958	0.958	0.284	No association
Emotional Awareness | PD | EO | delta	9	RPO	-0.037	-0.483	0.425	0.882	0.958	0.287	No association
Emotional Awareness | PD | EO | delta	9	CF	-0.156	-0.570	0.321	0.523	0.956	0.343	No association
Emotional Awareness | PD | EO | delta	9	CFP	-0.196	-0.597	0.283	0.420	0.956	0.384	No association
Emotional Awareness | PD | EO | delta	9	CPO	-0.034	-0.480	0.427	0.891	0.958	0.286	No association
Emotional Awareness | HC | EC | theta	9	LF	-0.185	-0.571	0.268	0.422	0.543	0.366	No association
Emotional Awareness | HC | EC | theta	9	RF	-0.120	-0.525	0.328	0.604	0.626	0.307	No association
Emotional Awareness | HC | EC | theta	9	LC	-0.331	-0.667	0.118	0.143	0.228	0.739	No association
Emotional Awareness | HC | EC | theta	9	RC	-0.325	-0.664	0.124	0.150	0.228	0.714	No association
Emotional Awareness | HC | EC | theta	9	LPO	-0.443	-0.734	-0.014	0.044	0.156	1.796	Nominal $p < .05$ only
Emotional Awareness | HC | EC | theta	9	RPO	-0.429	-0.726	0.003	0.052	0.156	1.584	No association
Emotional Awareness | HC | EC | theta	9	CF	-0.113	-0.519	0.335	0.626	0.626	0.302	No association
Emotional Awareness | HC | EC | theta	9	CFP	-0.324	-0.663	0.125	0.152	0.228	0.708	No association

FDR_family	FDR_family_size	Region	*r*	CI_95_lower	CI_95_upper	*p*	*q*_FDR	BF_{10}	Evidence_level
Emotional Awareness | HC | EC | theta	9	CPO	-0.479	-0.755	-0.059	0.028	0.156	2.588	Nominal *p* < .05 only
Emotional Awareness | PD | EC | theta	9	LF	0.262	-0.218	0.640	0.279	0.590	0.490	No association
Emotional Awareness | PD | EC | theta	9	RF	0.250	-0.230	0.632	0.302	0.590	0.466	No association
Emotional Awareness | PD | EC | theta	9	LC	0.196	-0.283	0.597	0.421	0.590	0.384	No association
Emotional Awareness | PD | EC | theta	9	RC	0.193	-0.286	0.595	0.429	0.590	0.380	No association
Emotional Awareness | PD | EC | theta	9	LPO	0.110	-0.363	0.537	0.655	0.706	0.311	No association
Emotional Awareness | PD | EC | theta	9	RPO	0.093	-0.377	0.525	0.706	0.706	0.303	No association
Emotional Awareness | PD | EC | theta	9	CF	0.286	-0.193	0.655	0.235	0.590	0.548	No association
Emotional Awareness | PD | EC | theta	9	CFP	0.248	-0.232	0.631	0.305	0.590	0.464	No association
Emotional Awareness | PD | EC | theta	9	CPO	0.181	-0.298	0.587	0.459	0.590	0.367	No association
Emotional Awareness | HC | EO | theta	9	LF	0.060	-0.382	0.479	0.797	0.910	0.279	No association
Emotional Awareness | HC | EO | theta	9	RF	0.128	-0.322	0.530	0.581	0.910	0.312	No association
Emotional Awareness | HC | EO | theta	9	LC	-0.113	-0.519	0.335	0.627	0.910	0.302	No association
Emotional Awareness | HC | EO | theta	9	RC	-0.100	-0.510	0.347	0.667	0.910	0.295	No association
Emotional Awareness | HC | EO | theta	9	LPO	-0.077	-0.492	0.367	0.740	0.910	0.285	No association
Emotional Awareness | HC | EO | theta	9	RPO	-0.094	-0.505	0.352	0.686	0.910	0.292	No association
Emotional Awareness | HC | EO | theta	9	CF	0.234	-0.220	0.604	0.308	0.910	0.440	No association

FDR_family	FDR_family_size	Region	*r*	CI_95_lower	CI_95_upper	*p*	*q*_FDR	BF_{10}	Evidence_level
Emotional Awareness | HC | EO | theta	9	CFP	-0.005	-0.435	0.428	0.984	0.984	0.270	No association
Emotional Awareness | HC | EO | theta	9	CPO	-0.056	-0.476	0.385	0.809	0.910	0.278	No association
Emotional Awareness | PD | EO | theta	9	LF	0.041	-0.421	0.486	0.868	0.968	0.287	No association
Emotional Awareness | PD | EO | theta	9	RF	0.114	-0.359	0.540	0.643	0.968	0.314	No association
Emotional Awareness | PD | EO | theta	9	LC	0.019	-0.439	0.469	0.940	0.968	0.285	No association
Emotional Awareness | PD | EO | theta	9	RC	0.023	-0.436	0.472	0.926	0.968	0.285	No association
Emotional Awareness | PD | EO | theta	9	LPO	-0.010	-0.462	0.446	0.968	0.968	0.284	No association
Emotional Awareness | PD | EO | theta	9	RPO	-0.023	-0.472	0.436	0.925	0.968	0.285	No association
Emotional Awareness | PD | EO | theta	9	CF	0.125	-0.349	0.548	0.610	0.968	0.320	No association
Emotional Awareness | PD | EO | theta	9	CFP	0.045	-0.418	0.489	0.855	0.968	0.288	No association
Emotional Awareness | PD | EO | theta	9	CPO	-0.022	-0.471	0.437	0.929	0.968	0.285	No association
Emotional Awareness | HC | EC | alpha	9	LF	0.184	-0.269	0.571	0.424	0.715	0.365	No association
Emotional Awareness | HC | EC | alpha	9	RF	0.223	-0.231	0.597	0.331	0.715	0.421	No association
Emotional Awareness | HC | EC | alpha	9	LC	0.110	-0.338	0.517	0.636	0.715	0.300	No association
Emotional Awareness | HC | EC | alpha	9	RC	0.077	-0.367	0.492	0.741	0.741	0.285	No association
Emotional Awareness | HC | EC | alpha	9	LPO	0.168	-0.284	0.559	0.466	0.715	0.347	No association
Emotional Awareness | HC | EC | alpha	9	RPO	0.137	-0.314	0.537	0.555	0.715	0.318	No association

FDR_family	FDR_family_size	Region	*r*	CI_95_lower	CI_95_upper	*p*	*q*_FDR	BF_{10}	Evidence_level
Emotional Awareness | HC | EC | alpha	9	CF	0.228	-0.226	0.601	0.320	0.715	0.430	No association
Emotional Awareness | HC | EC | alpha	9	CFP	0.121	-0.328	0.525	0.602	0.715	0.307	No association
Emotional Awareness | HC | EC | alpha	9	CPO	0.203	-0.251	0.583	0.378	0.715	0.389	No association
Emotional Awareness | PD | EC | alpha	9	LF	0.072	-0.395	0.509	0.770	0.987	0.295	No association
Emotional Awareness | PD | EC | alpha	9	RF	0.034	-0.427	0.481	0.891	0.987	0.286	No association
Emotional Awareness | PD | EC | alpha	9	LC	0.250	-0.230	0.632	0.302	0.792	0.466	No association
Emotional Awareness | PD | EC | alpha	9	RC	0.178	-0.301	0.585	0.467	0.792	0.363	No association
Emotional Awareness | PD | EC | alpha	9	LPO	0.194	-0.285	0.596	0.425	0.792	0.382	No association
Emotional Awareness | PD | EC | alpha	9	RPO	0.195	-0.284	0.597	0.423	0.792	0.383	No association
Emotional Awareness | PD | EC | alpha	9	CF	-0.004	-0.457	0.451	0.987	0.987	0.284	No association
Emotional Awareness | PD | EC | alpha	9	CFP	0.190	-0.289	0.593	0.436	0.792	0.377	No association
Emotional Awareness | PD | EC | alpha	9	CPO	0.154	-0.322	0.569	0.528	0.792	0.342	No association
Emotional Awareness | HC | EO | alpha	9	LF	0.174	-0.279	0.563	0.451	0.636	0.353	No association
Emotional Awareness | HC | EO | alpha	9	RF	0.179	-0.274	0.567	0.438	0.636	0.358	No association
Emotional Awareness | HC | EO | alpha	9	LC	0.120	-0.329	0.524	0.605	0.636	0.306	No association
Emotional Awareness | HC | EO | alpha	9	RC	0.110	-0.338	0.517	0.636	0.636	0.300	No association
Emotional Awareness | HC | EO | alpha	9	LPO	0.118	-0.331	0.523	0.611	0.636	0.305	No association

FDR_family	FDR_family_size	Region	*r*	CI_95_lower	CI_95_upper	*p*	*q*_FDR	BF_{10}	Evidence_level
Emotional Awareness | HC | EO | alpha	9	RPO	0.131	-0.319	0.533	0.572	0.636	0.314	No association
Emotional Awareness | HC | EO | alpha	9	CF	0.218	-0.236	0.594	0.342	0.636	0.413	No association
Emotional Awareness | HC | EO | alpha	9	CFP	0.191	-0.263	0.575	0.408	0.636	0.373	No association
Emotional Awareness | HC | EO | alpha	9	CPO	0.194	-0.259	0.577	0.399	0.636	0.377	No association
Emotional Awareness | PD | EO | alpha	9	LF	0.171	-0.307	0.580	0.485	0.759	0.356	No association
Emotional Awareness | PD | EO | alpha	9	RF	0.248	-0.233	0.631	0.306	0.759	0.462	No association
Emotional Awareness | PD | EO | alpha	9	LC	0.163	-0.315	0.574	0.506	0.759	0.349	No association
Emotional Awareness | PD | EO | alpha	9	RC	0.225	-0.256	0.616	0.355	0.759	0.423	No association
Emotional Awareness | PD | EO | alpha	9	LPO	-0.022	-0.472	0.436	0.928	0.971	0.285	No association
Emotional Awareness | PD | EO | alpha	9	RPO	0.009	-0.447	0.461	0.971	0.971	0.284	No association
Emotional Awareness | PD | EO | alpha	9	CF	0.173	-0.305	0.582	0.478	0.759	0.359	No association
Emotional Awareness | PD | EO | alpha	9	CFP	0.244	-0.236	0.629	0.313	0.759	0.456	No association
Emotional Awareness | PD | EO | alpha	9	CPO	0.020	-0.439	0.470	0.937	0.971	0.285	No association
Emotional Awareness | HC | EC | beta	9	LF	0.018	-0.417	0.446	0.940	0.994	0.271	No association
Emotional Awareness | HC | EC | beta	9	RF	-0.051	-0.473	0.389	0.825	0.994	0.277	No association
Emotional Awareness | HC | EC | beta	9	LC	0.068	-0.375	0.485	0.770	0.994	0.281	No association
Emotional Awareness | HC | EC | beta	9	RC	0.030	-0.407	0.456	0.897	0.994	0.272	No association

FDR_family	FDR_family_size	Region	*r*	CI_95_lower	CI_95_upper	*p*	*q*_FDR	BF_{10}	Evidence_level
Emotional Awareness | HC | EC | beta	9	LPO	0.002	-0.430	0.434	0.992	0.994	0.270	No association
Emotional Awareness | HC | EC | beta	9	RPO	-0.012	-0.441	0.422	0.960	0.994	0.271	No association
Emotional Awareness | HC | EC | beta	9	CF	-0.038	-0.462	0.401	0.872	0.994	0.274	No association
Emotional Awareness | HC | EC | beta	9	CFP	-0.002	-0.433	0.430	0.994	0.994	0.270	No association
Emotional Awareness | HC | EC | beta	9	CPO	-0.062	-0.481	0.380	0.791	0.994	0.279	No association
Emotional Awareness | PD | EC | beta	9	LF	0.284	-0.196	0.654	0.239	0.322	0.541	No association
Emotional Awareness | PD | EC | beta	9	RF	0.274	-0.206	0.647	0.257	0.322	0.517	No association
Emotional Awareness | PD | EC | beta	9	LC	0.235	-0.246	0.623	0.333	0.333	0.439	No association
Emotional Awareness | PD | EC | beta	9	RC	0.258	-0.222	0.638	0.286	0.322	0.483	No association
Emotional Awareness | PD | EC | beta	9	LPO	0.270	-0.210	0.645	0.264	0.322	0.507	No association
Emotional Awareness | PD | EC | beta	9	RPO	0.290	-0.189	0.658	0.228	0.322	0.559	No association
Emotional Awareness | PD | EC | beta	9	CF	0.280	-0.199	0.651	0.245	0.322	0.533	No association
Emotional Awareness | PD | EC | beta	9	CFP	0.298	-0.181	0.663	0.215	0.322	0.581	No association
Emotional Awareness | PD | EC | beta	9	CPO	0.317	-0.161	0.674	0.187	0.322	0.640	No association
Emotional Awareness | HC | EO | beta	9	LF	0.216	-0.238	0.592	0.348	0.609	0.409	No association
Emotional Awareness | HC | EO | beta	9	RF	0.049	-0.391	0.471	0.832	0.832	0.276	No association
Emotional Awareness | HC | EO | beta	9	LC	0.266	-0.187	0.626	0.244	0.609	0.511	No association

FDR_family	FDR_family_size	Region	*r*	CI_95_lower	CI_95_upper	*p*	*q*_FDR	BF_{10}	Evidence_level
Emotional Awareness | HC | EO | beta	9	RC	0.134	-0.316	0.535	0.563	0.633	0.316	No association
Emotional Awareness | HC | EO | beta	9	LPO	0.245	-0.209	0.612	0.285	0.609	0.462	No association
Emotional Awareness | HC | EO | beta	9	RPO	0.165	-0.287	0.557	0.474	0.609	0.344	No association
Emotional Awareness | HC | EO | beta	9	CF	0.191	-0.262	0.575	0.407	0.609	0.373	No association
Emotional Awareness | HC | EO | beta	9	CFP	0.233	-0.221	0.604	0.310	0.609	0.438	No association
Emotional Awareness | HC | EO | beta	9	CPO	0.245	-0.209	0.612	0.285	0.609	0.463	No association
Emotional Awareness | PD | EO | beta	9	LF	0.087	-0.383	0.520	0.725	0.838	0.301	No association
Emotional Awareness | PD | EO | beta	9	RF	0.109	-0.363	0.537	0.657	0.838	0.311	No association
Emotional Awareness | PD | EO | beta	9	LC	0.055	-0.409	0.497	0.822	0.838	0.291	No association
Emotional Awareness | PD | EO | beta	9	RC	0.091	-0.379	0.523	0.711	0.838	0.303	No association
Emotional Awareness | PD | EO | beta	9	LPO	0.059	-0.406	0.500	0.810	0.838	0.292	No association
Emotional Awareness | PD | EO | beta	9	RPO	0.069	-0.398	0.507	0.780	0.838	0.294	No association
Emotional Awareness | PD | EO | beta	9	CF	0.069	-0.398	0.507	0.779	0.838	0.294	No association
Emotional Awareness | PD | EO | beta	9	CFP	0.097	-0.373	0.528	0.692	0.838	0.305	No association
Emotional Awareness | PD | EO | beta	9	CPO	0.050	-0.413	0.493	0.838	0.838	0.289	No association
Trusting | HC | EC | delta	9	LF	-0.089	-0.501	0.357	0.702	0.982	0.290	No association
Trusting | HC | EC | delta	9	RF	-0.116	-0.521	0.333	0.617	0.982	0.304	No association
Trusting | HC | EC | delta	9	LC	-0.048	-0.470	0.392	0.837	0.982	0.276	No association

FDR_family	FDR_family_size	Region	*r*	CI_95_lower	CI_95_upper	*p*	*q*_FDR	BF_{10}	Evidence_level
Trusting | HC | EC | delta	9	RC	-0.074	-0.490	0.369	0.750	0.982	0.283	No association
Trusting | HC | EC | delta	9	LPO	0.039	-0.400	0.463	0.868	0.982	0.274	No association
Trusting | HC | EC | delta	9	RPO	-0.005	-0.436	0.427	0.982	0.982	0.270	No association
Trusting | HC | EC | delta	9	CF	-0.116	-0.521	0.332	0.617	0.982	0.304	No association
Trusting | HC | EC | delta	9	CFP	-0.072	-0.488	0.371	0.757	0.982	0.283	No association
Trusting | HC | EC | delta	9	CPO	0.032	-0.406	0.457	0.892	0.982	0.273	No association
Trusting | PD | EC | delta	9	LF	-0.267	-0.643	0.213	0.270	0.270	0.501	No association
Trusting | PD | EC | delta	9	RF	-0.272	-0.646	0.208	0.261	0.270	0.512	No association
Trusting | PD | EC | delta	9	LC	-0.311	-0.671	0.167	0.195	0.270	0.622	No association
Trusting | PD | EC | delta	9	RC	-0.276	-0.649	0.204	0.252	0.270	0.523	No association
Trusting | PD | EC | delta	9	LPO	-0.346	-0.691	0.129	0.147	0.270	0.756	No association
Trusting | PD | EC | delta	9	RPO	-0.281	-0.652	0.199	0.244	0.270	0.535	No association
Trusting | PD | EC | delta	9	CF	-0.282	-0.653	0.197	0.242	0.270	0.538	No association
Trusting | PD | EC | delta	9	CFP	-0.289	-0.657	0.191	0.231	0.270	0.555	No association
Trusting | PD | EC | delta	9	CPO	-0.327	-0.680	0.149	0.172	0.270	0.678	No association
Trusting | HC | EO | delta	9	LF	0.063	-0.379	0.482	0.786	0.810	0.280	No association
Trusting | HC | EO | delta	9	RF	0.068	-0.374	0.486	0.768	0.810	0.281	No association
Trusting | HC | EO | delta	9	LC	0.116	-0.333	0.521	0.618	0.810	0.304	No association
Trusting | HC | EO | delta	9	RC	0.081	-0.364	0.495	0.728	0.810	0.286	No association
Trusting | HC | EO | delta	9	LPO	0.080	-0.364	0.495	0.730	0.810	0.286	No association
Trusting | HC | EO | delta	9	RPO	0.057	-0.384	0.477	0.806	0.810	0.278	No association
Trusting | HC | EO | delta	9	CF	0.056	-0.385	0.476	0.810	0.810	0.278	No association
Trusting | HC | EO | delta	9	CFP	0.073	-0.370	0.490	0.752	0.810	0.283	No association
Trusting | HC | EO | delta	9	CPO	0.138	-0.312	0.538	0.551	0.810	0.319	No association
Trusting | PD | EO | delta	9	LF	-0.158	-0.571	0.319	0.519	0.519	0.345	No association
Trusting | PD | EO | delta	9	RF	-0.200	-0.599	0.280	0.413	0.480	0.388	No association
Trusting | PD | EO | delta	9	LC	-0.283	-0.653	0.197	0.241	0.480	0.539	No association
Trusting | PD | EO | delta	9	RC	-0.238	-0.625	0.242	0.326	0.480	0.445	No association

FDR_family	FDR_family_size	Region	*r*	CI_95_lower	CI_95_upper	*p*	*q*_FDR	BF_{10}	Evidence_level
Trusting | PD | EO | delta	9	LPO	-0.302	-0.665	0.176	0.209	0.480	0.593	No association
Trusting | PD | EO | delta	9	RPO	-0.268	-0.644	0.212	0.266	0.480	0.505	No association
Trusting | PD | EO | delta	9	CF	-0.194	-0.595	0.286	0.427	0.480	0.381	No association
Trusting | PD | EO | delta	9	CFP	-0.293	-0.660	0.186	0.223	0.480	0.567	No association
Trusting | PD | EO | delta	9	CPO	-0.342	-0.689	0.133	0.152	0.480	0.739	No association
Trusting | HC | EC | theta	9	LF	-0.031	-0.457	0.406	0.894	0.962	0.273	No association
Trusting | HC | EC | theta	9	RF	0.011	-0.423	0.441	0.962	0.962	0.271	No association
Trusting | HC | EC | theta	9	LC	-0.040	-0.464	0.398	0.862	0.962	0.274	No association
Trusting | HC | EC | theta	9	RC	0.017	-0.418	0.445	0.943	0.962	0.271	No association
Trusting | HC | EC | theta	9	LPO	0.031	-0.406	0.457	0.894	0.962	0.273	No association
Trusting | HC | EC | theta	9	RPO	0.069	-0.374	0.486	0.767	0.962	0.282	No association
Trusting | HC | EC | theta	9	CF	-0.015	-0.444	0.420	0.950	0.962	0.271	No association
Trusting | HC | EC | theta	9	CFP	-0.053	-0.474	0.388	0.821	0.962	0.277	No association
Trusting | HC | EC | theta	9	CPO	0.011	-0.422	0.441	0.961	0.962	0.271	No association
Trusting | PD | EC | theta	9	LF	0.728	0.408	0.888	0.000	0.004	91.437	FDR-corrected q < .05
Trusting | PD | EC | theta	9	RF	0.655	0.287	0.855	0.002	0.006	21.087	FDR-corrected q < .05
Trusting | PD | EC | theta	9	LC	0.648	0.274	0.851	0.003	0.006	18.404	FDR-corrected q < .05
Trusting | PD | EC | theta	9	RC	0.623	0.236	0.840	0.004	0.007	12.413	FDR-corrected q < .05
Trusting | PD | EC | theta	9	LPO	0.420	-0.042	0.734	0.073	0.073	1.267	Exploratory FDR-level .05 ≤ q < .10
Trusting | PD | EC | theta	9	RPO	0.446	-0.010	0.749	0.055	0.062	1.569	Exploratory FDR-level .05 ≤ q < .10
Trusting | PD | EC | theta	9	CF	0.670	0.310	0.862	0.002	0.006	27.337	FDR-corrected q < .05
Trusting | PD | EC | theta	9	CFP	0.629	0.245	0.843	0.004	0.007	13.614	FDR-corrected q < .05
Trusting | PD | EC | theta	9	CPO	0.516	0.081	0.786	0.024	0.030	3.081	FDR-corrected q < .05
Trusting | HC | EO | theta	9	LF	-0.174	-0.564	0.278	0.450	0.450	0.353	No association
Trusting | HC | EO | theta	9	RF	-0.180	-0.567	0.273	0.435	0.450	0.360	No association
Trusting | HC | EO | theta	9	LC	-0.281	-0.636	0.171	0.217	0.326	0.552	No association

FDR_family	FDR_family_size	Region	*r*	CI_95_lower	CI_95_upper	*p*	*q*_FDR	BF_{10}	Evidence_level
Trusting | HC | EO | theta	9	RC	-0.283	-0.637	0.169	0.214	0.326	0.558	No association
Trusting | HC | EO | theta	9	LPO	-0.462	-0.745	-0.038	0.035	0.105	2.164	Nominal $p < .05$ only
Trusting | HC | EO | theta	9	RPO	-0.468	-0.748	-0.045	0.033	0.105	2.300	Nominal $p < .05$ only
Trusting | HC | EO | theta	9	CF	-0.197	-0.579	0.257	0.393	0.450	0.381	No association
Trusting | HC | EO | theta	9	CFP	-0.302	-0.649	0.149	0.183	0.326	0.621	No association
Trusting | HC | EO | theta	9	CPO	-0.464	-0.746	-0.040	0.034	0.105	2.205	Nominal $p < .05$ only
Trusting | PD | EO | theta	9	LF	0.535	0.106	0.796	0.018	0.052	3.780	Exploratory FDR-level $.05 \leq q < .10$
Trusting | PD | EO | theta	9	RF	0.540	0.114	0.798	0.017	0.052	4.011	Exploratory FDR-level $.05 \leq q < .10$
Trusting | PD | EO | theta	9	LC	0.501	0.061	0.778	0.029	0.052	2.619	Exploratory FDR-level $.05 \leq q < .10$
Trusting | PD | EO | theta	9	RC	0.513	0.077	0.785	0.025	0.052	2.977	Exploratory FDR-level $.05 \leq q < .10$
Trusting | PD | EO | theta	9	LPO	0.440	-0.018	0.745	0.059	0.067	1.486	Exploratory FDR-level $.05 \leq q < .10$
Trusting | PD | EO | theta	9	RPO	0.410	-0.055	0.728	0.082	0.082	1.167	Exploratory FDR-level $.05 \leq q < .10$
Trusting | PD | EO | theta	9	CF	0.510	0.073	0.783	0.026	0.052	2.876	Exploratory FDR-level $.05 \leq q < .10$
Trusting | PD | EO | theta	9	CFP	0.477	0.029	0.766	0.039	0.058	2.072	Exploratory FDR-level $.05 \leq q < .10$
Trusting | PD | EO | theta	9	CPO	0.462	0.010	0.757	0.046	0.060	1.802	Exploratory FDR-level $.05 \leq q < .10$
Trusting | HC | EC | alpha	9	LF	-0.016	-0.445	0.418	0.943	0.998	0.271	No association
Trusting | HC | EC | alpha	9	RF	0.001	-0.431	0.433	0.996	0.998	0.270	No association
Trusting | HC | EC | alpha	9	LC	0.035	-0.403	0.460	0.881	0.998	0.273	No association
Trusting | HC | EC | alpha	9	RC	0.016	-0.418	0.445	0.944	0.998	0.271	No association
Trusting | HC | EC | alpha	9	LPO	-0.063	-0.482	0.379	0.785	0.998	0.280	No association
Trusting | HC | EC | alpha	9	RPO	-0.016	-0.445	0.419	0.945	0.998	0.271	No association
Trusting | HC | EC | alpha	9	CF	-0.001	-0.432	0.431	0.998	0.998	0.270	No association

**FDR_family**	**FDR_family_size**	**Region**	***r***	**CI_95_lower**	**CI_95_upper**	***p***	***q*_FDR**	**BF_{10}**	**Evidence_level**
Trusting | HC | EC | alpha	9	CFP	0.022	-0.413	0.450	0.924	0.998	0.271	No association
Trusting | HC | EC | alpha	9	CPO	-0.042	-0.465	0.397	0.858	0.998	0.274	No association
Trusting | PD | EC | alpha	9	LF	0.175	-0.304	0.583	0.475	0.601	0.360	No association
Trusting | PD | EC | alpha	9	RF	0.128	-0.346	0.550	0.601	0.601	0.322	No association
Trusting | PD | EC | alpha	9	LC	0.198	-0.281	0.599	0.416	0.601	0.387	No association
Trusting | PD | EC | alpha	9	RC	0.162	-0.315	0.574	0.508	0.601	0.348	No association
Trusting | PD | EC | alpha	9	LPO	0.253	-0.228	0.634	0.296	0.601	0.472	No association
Trusting | PD | EC | alpha	9	RPO	0.192	-0.287	0.594	0.431	0.601	0.379	No association
Trusting | PD | EC | alpha	9	CF	0.132	-0.343	0.553	0.589	0.601	0.325	No association
Trusting | PD | EC | alpha	9	CFP	0.164	-0.313	0.576	0.502	0.601	0.350	No association
Trusting | PD | EC | alpha	9	CPO	0.237	-0.244	0.624	0.329	0.601	0.442	No association
Trusting | HC | EO | alpha	9	LF	0.085	-0.360	0.499	0.713	0.910	0.288	No association
Trusting | HC | EO | alpha	9	RF	-0.026	-0.453	0.410	0.910	0.910	0.272	No association
Trusting | HC | EO | alpha	9	LC	0.071	-0.372	0.487	0.761	0.910	0.282	No association
Trusting | HC | EO | alpha	9	RC	-0.032	-0.457	0.405	0.891	0.910	0.273	No association
Trusting | HC | EO | alpha	9	LPO	0.102	-0.345	0.511	0.661	0.910	0.296	No association
Trusting | HC | EO | alpha	9	RPO	0.096	-0.350	0.507	0.679	0.910	0.293	No association
Trusting | HC | EO | alpha	9	CF	0.049	-0.391	0.471	0.833	0.910	0.276	No association
Trusting | HC | EO | alpha	9	CFP	0.031	-0.406	0.457	0.892	0.910	0.273	No association
Trusting | HC | EO | alpha	9	CPO	0.040	-0.399	0.463	0.865	0.910	0.274	No association
Trusting | PD | EO | alpha	9	LF	0.556	0.136	0.807	0.013	0.081	4.867	Exploratory FDR-level .05 ≤ *q* < .10
Trusting | PD | EO | alpha	9	RF	0.495	0.053	0.775	0.031	0.081	2.465	Exploratory FDR-level .05 ≤ *q* < .10
Trusting | PD | EO | alpha	9	LC	0.468	0.018	0.761	0.043	0.081	1.900	Exploratory FDR-level .05 ≤ *q* < .10
Trusting | PD | EO | alpha	9	RC	0.465	0.013	0.759	0.045	0.081	1.846	Exploratory FDR-level .05 ≤ *q* < .10
Trusting | PD | EO | alpha	9	LPO	0.358	-0.115	0.699	0.132	0.139	0.816	No association

FDR_family	FDR_family_size	Region	*r*	CI_95_lower	CI_95_upper	*p*	*q*_FDR	BF_{10}	Evidence_level
Trusting | PD | EO | alpha	9	RPO	0.352	-0.122	0.695	0.139	0.139	0.786	No association
Trusting | PD | EO | alpha	9	CF	0.441	-0.017	0.746	0.059	0.087	1.495	Exploratory FDR-level .05 ≤ *q* < .10
Trusting | PD | EO | alpha	9	CFP	0.509	0.072	0.783	0.026	0.081	2.856	Exploratory FDR-level .05 ≤ *q* < .10
Trusting | PD | EO | alpha	9	CPO	0.428	-0.033	0.739	0.068	0.087	1.345	Exploratory FDR-level .05 ≤ *q* < .10
Trusting | HC | EC | beta	9	LF	0.224	-0.229	0.598	0.328	0.862	0.423	No association
Trusting | HC | EC | beta	9	RF	0.274	-0.179	0.631	0.229	0.862	0.532	No association
Trusting | HC | EC | beta	9	LC	0.083	-0.362	0.497	0.720	0.928	0.287	No association
Trusting | HC | EC | beta	9	RC	0.164	-0.289	0.556	0.479	0.862	0.342	No association
Trusting | HC | EC | beta	9	LPO	0.044	-0.395	0.467	0.850	0.928	0.275	No association
Trusting | HC | EC | beta	9	RPO	0.037	-0.401	0.461	0.874	0.928	0.273	No association
Trusting | HC | EC | beta	9	CF	0.268	-0.185	0.627	0.240	0.862	0.516	No association
Trusting | HC | EC | beta	9	CFP	0.190	-0.264	0.574	0.410	0.862	0.371	No association
Trusting | HC | EC | beta	9	CPO	0.021	-0.414	0.449	0.928	0.928	0.271	No association
Trusting | PD | EC | beta	9	LF	-0.026	-0.475	0.433	0.916	0.916	0.285	No association
Trusting | PD | EC | beta	9	RF	0.059	-0.406	0.500	0.810	0.916	0.292	No association
Trusting | PD | EC | beta	9	LC	0.118	-0.355	0.543	0.631	0.916	0.316	No association
Trusting | PD | EC | beta	9	RC	0.073	-0.394	0.511	0.765	0.916	0.296	No association
Trusting | PD | EC | beta	9	LPO	0.194	-0.286	0.595	0.427	0.916	0.381	No association
Trusting | PD | EC | beta	9	RPO	0.130	-0.344	0.552	0.595	0.916	0.324	No association
Trusting | PD | EC | beta	9	CF	0.039	-0.423	0.484	0.875	0.916	0.287	No association
Trusting | PD | EC | beta	9	CFP	0.087	-0.383	0.520	0.724	0.916	0.301	No association
Trusting | PD | EC | beta	9	CPO	0.185	-0.294	0.589	0.449	0.916	0.371	No association
Trusting | HC | EO | beta	9	LF	-0.066	-0.484	0.376	0.775	0.984	0.281	No association
Trusting | HC | EO | beta	9	RF	-0.023	-0.450	0.413	0.921	0.984	0.272	No association
Trusting | HC | EO | beta	9	LC	-0.117	-0.522	0.332	0.615	0.984	0.304	No association
Trusting | HC | EO | beta	9	RC	0.019	-0.416	0.447	0.935	0.984	0.271	No association

FDR_family	FDR_family_size	Region	*r*	CI_95_lower	CI_95_upper	*p*	*q*_FDR	BF_{10}	Evidence_level
Trusting | HC | EO | beta	9	LPO	-0.075	-0.491	0.369	0.747	0.984	0.284	No association
Trusting | HC | EO | beta	9	RPO	-0.052	-0.473	0.389	0.824	0.984	0.277	No association
Trusting | HC | EO | beta	9	CF	-0.023	-0.450	0.413	0.921	0.984	0.272	No association
Trusting | HC | EO | beta	9	CFP	0.005	-0.428	0.436	0.984	0.984	0.270	No association
Trusting | HC | EO | beta	9	CPO	-0.094	-0.505	0.352	0.685	0.984	0.292	No association
Trusting | PD | EO | beta	9	LF	-0.200	-0.600	0.280	0.412	0.861	0.389	No association
Trusting | PD | EO | beta	9	RF	-0.151	-0.566	0.326	0.538	0.861	0.339	No association
Trusting | PD | EO | beta	9	LC	-0.060	-0.500	0.406	0.809	0.861	0.292	No association
Trusting | PD | EO | beta	9	RC	-0.135	-0.555	0.340	0.582	0.861	0.327	No association
Trusting | PD | EO | beta	9	LPO	-0.043	-0.488	0.419	0.861	0.861	0.288	No association
Trusting | PD | EO | beta	9	RPO	-0.154	-0.569	0.323	0.529	0.861	0.342	No association
Trusting | PD | EO | beta	9	CF	-0.150	-0.566	0.326	0.539	0.861	0.338	No association
Trusting | PD | EO | beta	9	CFP	-0.093	-0.525	0.377	0.704	0.861	0.304	No association
Trusting | PD | EO | beta	9	CPO	-0.069	-0.508	0.397	0.778	0.861	0.295	No association

Note. Pearson correlations were computed within each group. FDR correction was applied across the nine ROIs within each group, resting-state condition, frequency band, and self-report construct. *q*_FDR denotes the Benjamini–Hochberg FDR-adjusted *p* value. BF_{10} denotes the Bayes factor in favor of the alternative hypothesis of a non-zero correlation over the null hypothesis. Results with $q < .05$ were considered FDR-corrected associations, and results with $.05 \leqslant q < .10$ were considered exploratory FDR-level patterns.

Supplementary Table S7

Supplementary Table S7A. RBP age-adjusted partial correlations

FDR_family	FDR_family_size	Region	partial_*r*_controlling_age	CI_95_lower	CI_95_upper	*p*_partial	*q*_FDR_partial	Evidence_level
BPQ-VSF | HC | EC | delta	9	LF	0.096	-0.362	0.516	0.688	0.979	No association
BPQ-VSF | HC | EC | delta	9	RF	0.109	-0.351	0.526	0.648	0.979	No association
BPQ-VSF | HC | EC | delta	9	LC	0.009	-0.436	0.449	0.971	0.979	No association
BPQ-VSF | HC | EC | delta	9	RC	0.034	-0.415	0.470	0.886	0.979	No association
BPQ-VSF | HC | EC | delta	9	LPO	-0.049	-0.481	0.402	0.837	0.979	No association
BPQ-VSF | HC | EC | delta	9	RPO	0.011	-0.433	0.452	0.962	0.979	No association
BPQ-VSF | HC | EC | delta	9	CF	0.101	-0.358	0.520	0.672	0.979	No association
BPQ-VSF | HC | EC | delta	9	CFP	0.006	-0.438	0.447	0.979	0.979	No association
BPQ-VSF | HC | EC | delta	9	CPO	-0.070	-0.497	0.384	0.769	0.979	No association
BPQ-VSF | PD | EC | delta	9	LF	-0.323	-0.686	0.169	0.191	0.337	No association
BPQ-VSF | PD | EC | delta	9	RF	-0.414	-0.738	0.065	0.087	0.337	No association
BPQ-VSF | PD | EC | delta	9	LC	-0.325	-0.687	0.168	0.189	0.337	No association
BPQ-VSF | PD | EC | delta	9	RC	-0.382	-0.720	0.104	0.118	0.337	No association
BPQ-VSF | PD | EC | delta	9	LPO	-0.245	-0.639	0.251	0.328	0.337	No association
BPQ-VSF | PD | EC | delta	9	RPO	-0.253	-0.644	0.242	0.311	0.337	No association
BPQ-VSF | PD | EC | delta	9	CF	-0.361	-0.708	0.128	0.142	0.337	No association
BPQ-VSF | PD | EC | delta	9	CFP	-0.290	-0.666	0.205	0.244	0.337	No association
BPQ-VSF | PD | EC | delta	9	CPO	-0.240	-0.636	0.255	0.337	0.337	No association
BPQ-VSF | HC | EO | delta	9	LF	-0.085	-0.508	0.372	0.722	0.990	No association
BPQ-VSF | HC | EO | delta	9	RF	-0.031	-0.467	0.417	0.897	0.990	No association
BPQ-VSF | HC | EO | delta	9	LC	-0.042	-0.475	0.408	0.861	0.990	No association
BPQ-VSF | HC | EO | delta	9	RC	-0.003	-0.445	0.440	0.990	0.990	No association
BPQ-VSF | HC | EO | delta	9	LPO	-0.016	-0.455	0.430	0.947	0.990	No association

FDR_family	FDR_family_size	Region	partial_*r*_controlling_age	CI_95_lower	CI_95_upper	*p*_partial	*q*_FDR_partial	Evidence_level
BPQ-VSF | HC | EO | delta	9	RPO	0.031	-0.417	0.467	0.898	0.990	No association
BPQ-VSF | HC | EO | delta	9	CF	-0.114	-0.530	0.346	0.632	0.990	No association
BPQ-VSF | HC | EO | delta	9	CFP	-0.169	-0.569	0.296	0.476	0.990	No association
BPQ-VSF | HC | EO | delta	9	CPO	-0.064	-0.492	0.390	0.790	0.990	No association
BPQ-VSF | PD | EO | delta	9	LF	-0.462	-0.764	0.006	0.053	0.120	No association
BPQ-VSF | PD | EO | delta	9	RF	-0.530	-0.799	-0.084	0.024	0.120	Nominal $p < .05$ only
BPQ-VSF | PD | EO | delta	9	LC	-0.430	-0.747	0.047	0.075	0.135	No association
BPQ-VSF | PD | EO | delta	9	RC	-0.476	-0.771	-0.012	0.046	0.120	Nominal $p < .05$ only
BPQ-VSF | PD | EO | delta	9	LPO	-0.344	-0.699	0.146	0.162	0.209	No association
BPQ-VSF | PD | EO | delta	9	RPO	-0.319	-0.684	0.174	0.197	0.221	No association
BPQ-VSF | PD | EO | delta	9	CF	-0.498	-0.783	-0.041	0.035	0.120	Nominal $p < .05$ only
BPQ-VSF | PD | EO | delta	9	CFP	-0.400	-0.731	0.082	0.100	0.150	No association
BPQ-VSF | PD | EO | delta	9	CPO	-0.264	-0.651	0.231	0.290	0.290	No association
BPQ-VSF | HC | EC | theta	9	LF	-0.116	-0.531	0.344	0.626	0.720	No association
BPQ-VSF | HC | EC | theta	9	RF	-0.159	-0.562	0.305	0.503	0.720	No association
BPQ-VSF | HC | EC | theta	9	LC	-0.111	-0.528	0.348	0.640	0.720	No association
BPQ-VSF | HC | EC | theta	9	RC	-0.214	-0.599	0.253	0.366	0.720	No association
BPQ-VSF | HC | EC | theta	9	LPO	-0.243	-0.619	0.223	0.301	0.720	No association
BPQ-VSF | HC | EC | theta	9	RPO	-0.272	-0.638	0.194	0.246	0.720	No association
BPQ-VSF | HC | EC | theta	9	CF	-0.166	-0.567	0.299	0.485	0.720	No association
BPQ-VSF | HC | EC | theta	9	CFP	-0.081	-0.506	0.375	0.733	0.733	No association
BPQ-VSF | HC | EC | theta	9	CPO	-0.116	-0.531	0.344	0.626	0.720	No association
BPQ-VSF | PD | EC | theta	9	LF	0.395	-0.088	0.728	0.105	0.322	No association
BPQ-VSF | PD | EC | theta	9	RF	0.436	-0.038	0.750	0.070	0.322	No association
BPQ-VSF | PD | EC | theta	9	LC	0.254	-0.242	0.644	0.309	0.561	No association
BPQ-VSF | PD | EC | theta	9	RC	0.184	-0.309	0.599	0.465	0.623	No association
BPQ-VSF | PD | EC | theta	9	LPO	0.253	-0.243	0.644	0.312	0.561	No association
BPQ-VSF | PD | EC | theta	9	RPO	0.173	-0.320	0.592	0.492	0.623	No association

FDR_family	FDR_family_size	Region	partial_*r*_controlling_age	CI_95_lower	CI_95_upper	*p*_partial	*q*_FDR_partial	Evidence_level
BPQ-VSF | PD | EC | theta	9	CF	0.392	-0.091	0.726	0.107	0.322	No association
BPQ-VSF | PD | EC | theta	9	CFP	0.150	-0.341	0.576	0.553	0.623	No association
BPQ-VSF | PD | EC | theta	9	CPO	0.099	-0.386	0.541	0.696	0.696	No association
BPQ-VSF | HC | EO | theta	9	LF	-0.029	-0.465	0.419	0.905	0.977	No association
BPQ-VSF | HC | EO | theta	9	RF	-0.116	-0.531	0.344	0.627	0.977	No association
BPQ-VSF | HC | EO | theta	9	LC	-0.016	-0.455	0.429	0.946	0.977	No association
BPQ-VSF | HC | EO | theta	9	RC	-0.157	-0.561	0.307	0.508	0.977	No association
BPQ-VSF | HC | EO | theta	9	LPO	-0.070	-0.497	0.384	0.768	0.977	No association
BPQ-VSF | HC | EO | theta	9	RPO	-0.134	-0.544	0.328	0.574	0.977	No association
BPQ-VSF | HC | EO | theta	9	CF	0.007	-0.437	0.448	0.977	0.977	No association
BPQ-VSF | HC | EO | theta	9	CFP	0.046	-0.405	0.479	0.848	0.977	No association
BPQ-VSF | HC | EO | theta	9	CPO	-0.008	-0.449	0.436	0.972	0.977	No association
BPQ-VSF | PD | EO | theta	9	LF	0.496	0.037	0.782	0.036	0.103	Nominal p < .05 only
BPQ-VSF | PD | EO | theta	9	RF	0.453	-0.018	0.759	0.059	0.103	No association
BPQ-VSF | PD | EO | theta	9	LC	0.452	-0.019	0.758	0.060	0.103	No association
BPQ-VSF | PD | EO | theta	9	RC	0.405	-0.077	0.733	0.096	0.108	No association
BPQ-VSF | PD | EO | theta	9	LPO	0.424	-0.054	0.743	0.080	0.103	No association
BPQ-VSF | PD | EO | theta	9	RPO	0.452	-0.019	0.759	0.060	0.103	No association
BPQ-VSF | PD | EO | theta	9	CF	0.439	-0.035	0.752	0.068	0.103	No association
BPQ-VSF | PD | EO | theta	9	CFP	0.364	-0.124	0.710	0.137	0.137	No association
BPQ-VSF | PD | EO | theta	9	CPO	0.436	-0.038	0.750	0.070	0.103	No association
BPQ-VSF | HC | EC | alpha	9	LF	-0.032	-0.468	0.416	0.894	0.982	No association
BPQ-VSF | HC | EC | alpha	9	RF	-0.026	-0.463	0.421	0.914	0.982	No association
BPQ-VSF | HC | EC | alpha	9	LC	0.023	-0.424	0.461	0.922	0.982	No association
BPQ-VSF | HC | EC | alpha	9	RC	0.013	-0.432	0.453	0.958	0.982	No association
BPQ-VSF | HC | EC | alpha	9	LPO	0.099	-0.360	0.519	0.679	0.982	No association
BPQ-VSF | HC | EC | alpha	9	RPO	0.036	-0.413	0.471	0.881	0.982	No association
BPQ-VSF | HC | EC | alpha	9	CF	-0.019	-0.457	0.427	0.937	0.982	No association

FDR_family	FDR_family_size	Region	partial_*r*_controlling_age	CI_95_lower	CI_95_upper	*p*_partial	*q*_FDR_partial	Evidence_level
BPQ-VSF | HC | EC | alpha	9	CFP	-0.005	-0.447	0.438	0.982	0.982	No association
BPQ-VSF | HC | EC | alpha	9	CPO	0.073	-0.382	0.499	0.760	0.982	No association
BPQ-VSF | PD | EC | alpha	9	LF	-0.042	-0.499	0.433	0.868	0.957	No association
BPQ-VSF | PD | EC | alpha	9	RF	0.055	-0.422	0.509	0.827	0.957	No association
BPQ-VSF | PD | EC | alpha	9	LC	0.086	-0.397	0.532	0.734	0.957	No association
BPQ-VSF | PD | EC | alpha	9	RC	0.190	-0.304	0.603	0.450	0.957	No association
BPQ-VSF | PD | EC | alpha	9	LPO	0.014	-0.456	0.477	0.957	0.957	No association
BPQ-VSF | PD | EC | alpha	9	RPO	0.096	-0.388	0.539	0.705	0.957	No association
BPQ-VSF | PD | EC | alpha	9	CF	-0.017	-0.480	0.454	0.948	0.957	No association
BPQ-VSF | PD | EC | alpha	9	CFP	0.089	-0.394	0.534	0.724	0.957	No association
BPQ-VSF | PD | EC | alpha	9	CPO	0.098	-0.386	0.540	0.698	0.957	No association
BPQ-VSF | HC | EO | alpha	9	LF	-0.013	-0.453	0.432	0.957	0.998	No association
BPQ-VSF | HC | EO | alpha	9	RF	-0.051	-0.483	0.400	0.829	0.998	No association
BPQ-VSF | HC | EO | alpha	9	LC	0.038	-0.412	0.472	0.875	0.998	No association
BPQ-VSF | HC | EO | alpha	9	RC	0.001	-0.442	0.443	0.998	0.998	No association
BPQ-VSF | HC | EO | alpha	9	LPO	-0.017	-0.456	0.429	0.942	0.998	No association
BPQ-VSF | HC | EO | alpha	9	RPO	-0.036	-0.471	0.413	0.881	0.998	No association
BPQ-VSF | HC | EO | alpha	9	CF	0.054	-0.398	0.485	0.821	0.998	No association
BPQ-VSF | HC | EO | alpha	9	CFP	0.095	-0.362	0.516	0.689	0.998	No association
BPQ-VSF | HC | EO | alpha	9	CPO	-0.003	-0.445	0.440	0.990	0.998	No association
BPQ-VSF | PD | EO | alpha	9	LF	0.082	-0.400	0.529	0.746	0.939	No association
BPQ-VSF | PD | EO | alpha	9	RF	0.243	-0.252	0.638	0.331	0.939	No association
BPQ-VSF | PD | EO | alpha	9	LC	0.064	-0.416	0.515	0.802	0.939	No association
BPQ-VSF | PD | EO | alpha	9	RC	0.171	-0.321	0.591	0.497	0.939	No association
BPQ-VSF | PD | EO | alpha	9	LPO	0.053	-0.424	0.507	0.835	0.939	No association
BPQ-VSF | PD | EO | alpha	9	RPO	0.041	-0.434	0.498	0.872	0.939	No association
BPQ-VSF | PD | EO | alpha	9	CF	0.125	-0.363	0.559	0.621	0.939	No association
BPQ-VSF | PD | EO | alpha	9	CFP	0.021	-0.450	0.483	0.935	0.939	No association

FDR_family	FDR_family_size	Region	partial_*r*_controlling_age	CI_95_lower	CI_95_upper	*p*_partial	*q*_FDR_partial	Evidence_level
BPQ-VSF | PD | EO | alpha	9	CPO	-0.019	-0.482	0.451	0.939	0.939	No association
BPQ-VSF | HC | EC | beta	9	LF	-0.122	-0.535	0.339	0.610	0.980	No association
BPQ-VSF | HC | EC | beta	9	RF	-0.162	-0.564	0.302	0.494	0.980	No association
BPQ-VSF | HC | EC | beta	9	LC	-0.023	-0.461	0.424	0.924	0.980	No association
BPQ-VSF | HC | EC | beta	9	RC	-0.018	-0.457	0.428	0.939	0.980	No association
BPQ-VSF | HC | EC | beta	9	LPO	0.019	-0.427	0.457	0.938	0.980	No association
BPQ-VSF | HC | EC | beta	9	RPO	-0.006	-0.447	0.438	0.980	0.980	No association
BPQ-VSF | HC | EC | beta	9	CF	-0.127	-0.539	0.335	0.594	0.980	No association
BPQ-VSF | HC | EC | beta	9	CFP	0.039	-0.410	0.474	0.869	0.980	No association
BPQ-VSF | HC | EC | beta	9	CPO	0.104	-0.355	0.522	0.663	0.980	No association
BPQ-VSF | PD | EC | beta	9	LF	0.477	0.013	0.772	0.045	0.045	FDR-corrected $q < .05$
BPQ-VSF | PD | EC | beta	9	RF	0.558	0.123	0.813	0.016	0.034	FDR-corrected $q < .05$
BPQ-VSF | PD | EC | beta	9	LC	0.510	0.057	0.789	0.031	0.034	FDR-corrected $q < .05$
BPQ-VSF | PD | EC | beta	9	RC	0.547	0.107	0.807	0.019	0.034	FDR-corrected $q < .05$
BPQ-VSF | PD | EC | beta	9	LPO	0.600	0.184	0.833	0.009	0.034	FDR-corrected $q < .05$
BPQ-VSF | PD | EC | beta	9	RPO	0.564	0.132	0.816	0.015	0.034	FDR-corrected $q < .05$
BPQ-VSF | PD | EC | beta	9	CF	0.531	0.085	0.800	0.023	0.034	FDR-corrected $q < .05$
BPQ-VSF | PD | EC | beta	9	CFP	0.511	0.058	0.790	0.030	0.034	FDR-corrected $q < .05$
BPQ-VSF | PD | EC | beta	9	CPO	0.533	0.087	0.800	0.023	0.034	FDR-corrected $q < .05$
BPQ-VSF | HC | EO | beta	9	LF	0.143	-0.320	0.550	0.549	0.854	No association
BPQ-VSF | HC | EO | beta	9	RF	0.135	-0.327	0.545	0.570	0.854	No association
BPQ-VSF | HC | EO | beta	9	LC	0.044	-0.406	0.477	0.854	0.854	No association
BPQ-VSF | HC | EO | beta	9	RC	0.094	-0.364	0.515	0.693	0.854	No association
BPQ-VSF | HC | EO | beta	9	LPO	0.098	-0.360	0.518	0.681	0.854	No association
BPQ-VSF | HC | EO | beta	9	RPO	0.062	-0.391	0.491	0.796	0.854	No association
BPQ-VSF | HC | EO | beta	9	CF	0.155	-0.309	0.559	0.515	0.854	No association
BPQ-VSF | HC | EO | beta	9	CFP	0.202	-0.264	0.591	0.394	0.854	No association
BPQ-VSF | HC | EO | beta	9	CPO	0.161	-0.303	0.564	0.497	0.854	No association

FDR_family	FDR_family_size	Region	partial_*r*_controlling_age	CI_95_lower	CI_95_upper	*p*_partial	*q*_FDR_partial	Evidence_level
BPQ-VSF | PD | EO | beta	9	LF	0.352	-0.137	0.703	0.152	0.152	No association
BPQ-VSF | PD | EO | beta	9	RF	0.443	-0.030	0.754	0.065	0.106	No association
BPQ-VSF | PD | EO | beta	9	LC	0.456	-0.014	0.761	0.057	0.106	No association
BPQ-VSF | PD | EO | beta	9	RC	0.478	0.015	0.773	0.045	0.106	Nominal p < .05 only
BPQ-VSF | PD | EO | beta	9	LPO	0.446	-0.026	0.756	0.063	0.106	No association
BPQ-VSF | PD | EO | beta	9	RPO	0.435	-0.040	0.750	0.071	0.106	No association
BPQ-VSF | PD | EO | beta	9	CF	0.414	-0.065	0.738	0.087	0.112	No association
BPQ-VSF | PD | EO | beta	9	CFP	0.444	-0.029	0.754	0.065	0.106	No association
BPQ-VSF | PD | EO | beta	9	CPO	0.390	-0.094	0.725	0.109	0.123	No association
Not-Distracting | HC | EC | delta	9	LF	0.059	-0.394	0.489	0.806	0.933	No association
Not-Distracting | HC | EC | delta	9	RF	0.020	-0.426	0.458	0.933	0.933	No association
Not-Distracting | HC | EC | delta	9	LC	0.124	-0.337	0.537	0.602	0.933	No association
Not-Distracting | HC | EC | delta	9	RC	0.070	-0.384	0.497	0.769	0.933	No association
Not-Distracting | HC | EC | delta	9	LPO	0.131	-0.331	0.542	0.581	0.933	No association
Not-Distracting | HC | EC | delta	9	RPO	0.100	-0.359	0.519	0.676	0.933	No association
Not-Distracting | HC | EC | delta	9	CF	0.034	-0.415	0.470	0.886	0.933	No association
Not-Distracting | HC | EC | delta	9	CFP	0.128	-0.334	0.540	0.592	0.933	No association
Not-Distracting | HC | EC | delta	9	CPO	0.148	-0.315	0.554	0.533	0.933	No association
Not-Distracting | PD | EC | delta	9	LF	-0.277	-0.659	0.218	0.266	0.316	No association
Not-Distracting | PD | EC | delta	9	RF	-0.297	-0.671	0.197	0.231	0.316	No association
Not-Distracting | PD | EC | delta	9	LC	-0.250	-0.642	0.245	0.316	0.316	No association
Not-Distracting | PD | EC | delta	9	RC	-0.326	-0.688	0.166	0.187	0.316	No association
Not-Distracting | PD | EC | delta	9	LPO	-0.309	-0.678	0.184	0.212	0.316	No association
Not-Distracting | PD | EC | delta	9	RPO	-0.378	-0.718	0.108	0.122	0.316	No association
Not-Distracting | PD | EC | delta	9	CF	-0.291	-0.667	0.204	0.242	0.316	No association
Not-Distracting | PD | EC | delta	9	CFP	-0.258	-0.647	0.238	0.302	0.316	No association
Not-Distracting | PD | EC | delta	9	CPO	-0.299	-0.672	0.195	0.227	0.316	No association
Not-Distracting | HC | EO | delta	9	LF	0.157	-0.306	0.561	0.507	0.701	No association

FDR_family	FDR_family_size	Region	partial_*r*_controlling_age	CI_95_lower	CI_95_upper	*p*_partial	*q*_FDR_partial	Evidence_level
Not-Distracting | HC | EO | delta	9	RF	0.117	-0.343	0.532	0.623	0.701	No association
Not-Distracting | HC | EO | delta	9	LC	0.165	-0.299	0.566	0.487	0.701	No association
Not-Distracting | HC | EO | delta	9	RC	0.143	-0.319	0.551	0.547	0.701	No association
Not-Distracting | HC | EO | delta	9	LPO	0.141	-0.322	0.549	0.555	0.701	No association
Not-Distracting | HC | EO | delta	9	RPO	0.091	-0.366	0.513	0.704	0.704	No association
Not-Distracting | HC | EO | delta	9	CF	0.167	-0.298	0.567	0.482	0.701	No association
Not-Distracting | HC | EO | delta	9	CFP	0.255	-0.211	0.627	0.278	0.701	No association
Not-Distracting | HC | EO | delta	9	CPO	0.133	-0.329	0.544	0.576	0.701	No association
Not-Distracting | PD | EO | delta	9	LF	-0.234	-0.632	0.261	0.350	0.501	No association
Not-Distracting | PD | EO | delta	9	RF	-0.178	-0.596	0.315	0.479	0.501	No association
Not-Distracting | PD | EO | delta	9	LC	-0.205	-0.613	0.290	0.415	0.501	No association
Not-Distracting | PD | EO | delta	9	RC	-0.268	-0.653	0.227	0.282	0.501	No association
Not-Distracting | PD | EO | delta	9	LPO	-0.313	-0.680	0.180	0.206	0.501	No association
Not-Distracting | PD | EO | delta	9	RPO	-0.348	-0.701	0.142	0.157	0.501	No association
Not-Distracting | PD | EO | delta	9	CF	-0.171	-0.591	0.321	0.497	0.501	No association
Not-Distracting | PD | EO | delta	9	CFP	-0.170	-0.590	0.323	0.501	0.501	No association
Not-Distracting | PD | EO | delta	9	CPO	-0.262	-0.649	0.233	0.294	0.501	No association
Not-Distracting | HC | EC | theta	9	LF	-0.103	-0.522	0.355	0.665	0.936	No association
Not-Distracting | HC | EC | theta	9	RF	-0.032	-0.468	0.417	0.894	0.936	No association
Not-Distracting | HC | EC | theta	9	LC	-0.095	-0.516	0.363	0.691	0.936	No association
Not-Distracting | HC | EC | theta	9	RC	-0.019	-0.458	0.427	0.936	0.936	No association
Not-Distracting | HC | EC | theta	9	LPO	0.027	-0.421	0.464	0.911	0.936	No association
Not-Distracting | HC | EC | theta	9	RPO	0.033	-0.416	0.469	0.890	0.936	No association
Not-Distracting | HC | EC | theta	9	CF	-0.065	-0.493	0.389	0.787	0.936	No association
Not-Distracting | HC | EC | theta	9	CFP	-0.137	-0.546	0.326	0.566	0.936	No association
Not-Distracting | HC | EC | theta	9	CPO	-0.047	-0.480	0.404	0.844	0.936	No association
Not-Distracting | PD | EC | theta	9	LF	-0.396	-0.728	0.087	0.104	0.313	No association
Not-Distracting | PD | EC | theta	9	RF	-0.340	-0.696	0.151	0.167	0.365	No association

FDR_family	FDR_family_size	Region	partial_*r*_controlling_age	CI_95_lower	CI_95_upper	*p*_partial	*q*_FDR_partial	Evidence_level
Not-Distracting | PD | EC | theta	9	LC	-0.315	-0.682	0.178	0.203	0.365	No association
Not-Distracting | PD | EC | theta	9	RC	-0.266	-0.652	0.230	0.286	0.368	No association
Not-Distracting | PD | EC | theta	9	LPO	-0.151	-0.577	0.340	0.550	0.550	No association
Not-Distracting | PD | EC | theta	9	RPO	-0.202	-0.611	0.292	0.421	0.474	No association
Not-Distracting | PD | EC | theta	9	CF	-0.422	-0.743	0.056	0.081	0.313	No association
Not-Distracting | PD | EC | theta	9	CFP	-0.415	-0.739	0.064	0.087	0.313	No association
Not-Distracting | PD | EC | theta	9	CPO	-0.272	-0.656	0.223	0.274	0.368	No association
Not-Distracting | HC | EO | theta	9	LF	-0.136	-0.546	0.326	0.566	0.953	No association
Not-Distracting | HC | EO | theta	9	RF	-0.014	-0.454	0.431	0.953	0.953	No association
Not-Distracting | HC | EO | theta	9	LC	-0.162	-0.564	0.302	0.495	0.953	No association
Not-Distracting | HC | EO | theta	9	RC	-0.032	-0.468	0.416	0.894	0.953	No association
Not-Distracting | HC | EO | theta	9	LPO	-0.115	-0.531	0.345	0.628	0.953	No association
Not-Distracting | HC | EO | theta	9	RPO	-0.055	-0.485	0.398	0.819	0.953	No association
Not-Distracting | HC | EO | theta	9	CF	-0.094	-0.515	0.364	0.694	0.953	No association
Not-Distracting | HC | EO | theta	9	CFP	-0.153	-0.558	0.311	0.520	0.953	No association
Not-Distracting | HC | EO | theta	9	CPO	-0.124	-0.537	0.337	0.604	0.953	No association
Not-Distracting | PD | EO | theta	9	LF	-0.291	-0.667	0.204	0.242	0.311	No association
Not-Distracting | PD | EO | theta	9	RF	-0.320	-0.685	0.173	0.195	0.311	No association
Not-Distracting | PD | EO | theta	9	LC	-0.245	-0.639	0.250	0.327	0.327	No association
Not-Distracting | PD | EO | theta	9	RC	-0.268	-0.653	0.228	0.283	0.318	No association
Not-Distracting | PD | EO | theta	9	LPO	-0.299	-0.672	0.195	0.228	0.311	No association
Not-Distracting | PD | EO | theta	9	RPO	-0.340	-0.696	0.151	0.167	0.311	No association
Not-Distracting | PD | EO | theta	9	CF	-0.347	-0.700	0.144	0.159	0.311	No association
Not-Distracting | PD | EO | theta	9	CFP	-0.351	-0.703	0.139	0.153	0.311	No association
Not-Distracting | PD | EO | theta	9	CPO	-0.331	-0.691	0.161	0.180	0.311	No association
Not-Distracting | HC | EC | alpha	9	LF	-0.033	-0.469	0.415	0.889	0.957	No association
Not-Distracting | HC | EC | alpha	9	RF	-0.036	-0.471	0.413	0.881	0.957	No association
Not-Distracting | HC | EC | alpha	9	LC	-0.128	-0.540	0.334	0.592	0.957	No association

FDR_family	FDR_family_size	Region	partial_*r*_controlling_age	CI_95_lower	CI_95_upper	*p*_partial	*q*_FDR_partial	Evidence_level
Not-Distracting | HC | EC | alpha	9	RC	-0.078	-0.503	0.378	0.745	0.957	No association
Not-Distracting | HC | EC | alpha	9	LPO	-0.145	-0.552	0.318	0.542	0.957	No association
Not-Distracting | HC | EC | alpha	9	RPO	-0.128	-0.540	0.334	0.592	0.957	No association
Not-Distracting | HC | EC | alpha	9	CF	-0.013	-0.453	0.432	0.957	0.957	No association
Not-Distracting | HC | EC | alpha	9	CFP	-0.076	-0.501	0.379	0.751	0.957	No association
Not-Distracting | HC | EC | alpha	9	CPO	-0.151	-0.556	0.313	0.526	0.957	No association
Not-Distracting | PD | EC | alpha	9	LF	0.308	-0.186	0.677	0.214	0.238	No association
Not-Distracting | PD | EC | alpha	9	RF	0.415	-0.064	0.739	0.087	0.226	No association
Not-Distracting | PD | EC | alpha	9	LC	0.293	-0.201	0.668	0.238	0.238	No association
Not-Distracting | PD | EC | alpha	9	RC	0.383	-0.102	0.721	0.117	0.226	No association
Not-Distracting | PD | EC | alpha	9	LPO	0.338	-0.153	0.695	0.170	0.226	No association
Not-Distracting | PD | EC | alpha	9	RPO	0.422	-0.056	0.742	0.081	0.226	No association
Not-Distracting | PD | EC | alpha	9	CF	0.400	-0.082	0.730	0.100	0.226	No association
Not-Distracting | PD | EC | alpha	9	CFP	0.334	-0.158	0.693	0.176	0.226	No association
Not-Distracting | PD | EC | alpha	9	CPO	0.343	-0.148	0.698	0.164	0.226	No association
Not-Distracting | HC | EO | alpha	9	LF	-0.122	-0.536	0.339	0.608	0.880	No association
Not-Distracting | HC | EO | alpha	9	RF	-0.086	-0.509	0.371	0.718	0.880	No association
Not-Distracting | HC | EO | alpha	9	LC	-0.234	-0.613	0.233	0.321	0.880	No association
Not-Distracting | HC | EO | alpha	9	RC	-0.162	-0.564	0.302	0.494	0.880	No association
Not-Distracting | HC | EO | alpha	9	LPO	-0.058	-0.488	0.395	0.808	0.880	No association
Not-Distracting | HC | EO | alpha	9	RPO	-0.036	-0.471	0.413	0.880	0.880	No association
Not-Distracting | HC | EO | alpha	9	CF	-0.077	-0.503	0.378	0.746	0.880	No association
Not-Distracting | HC | EO | alpha	9	CFP	-0.181	-0.577	0.284	0.445	0.880	No association
Not-Distracting | HC | EO | alpha	9	CPO	-0.043	-0.477	0.407	0.857	0.880	No association
Not-Distracting | PD | EO | alpha	9	LF	-0.086	-0.532	0.397	0.734	0.989	No association
Not-Distracting | PD | EO | alpha	9	RF	-0.010	-0.475	0.459	0.967	0.989	No association
Not-Distracting | PD | EO | alpha	9	LC	0.061	-0.418	0.513	0.810	0.989	No association
Not-Distracting | PD | EO | alpha	9	RC	0.119	-0.369	0.555	0.639	0.989	No association

FDR_family	FDR_family_size	Region	partial_*r*_controlling_age	CI_95_lower	CI_95_upper	*p*_partial	*q*_FDR_partial	Evidence_level
Not-Distracting | PD | EO | alpha	9	LPO	0.294	-0.201	0.669	0.237	0.989	No association
Not-Distracting | PD | EO | alpha	9	RPO	0.314	-0.179	0.681	0.204	0.989	No association
Not-Distracting | PD | EO | alpha	9	CF	-0.004	-0.470	0.464	0.989	0.989	No association
Not-Distracting | PD | EO | alpha	9	CFP	0.011	-0.459	0.475	0.967	0.989	No association
Not-Distracting | PD | EO | alpha	9	CPO	0.230	-0.265	0.629	0.359	0.989	No association
Not-Distracting | HC | EC | beta	9	LF	-0.030	-0.466	0.418	0.899	0.962	No association
Not-Distracting | HC | EC | beta	9	RF	0.027	-0.421	0.464	0.911	0.962	No association
Not-Distracting | HC | EC | beta	9	LC	-0.038	-0.472	0.412	0.874	0.962	No association
Not-Distracting | HC | EC | beta	9	RC	-0.030	-0.467	0.418	0.899	0.962	No association
Not-Distracting | HC | EC | beta	9	LPO	-0.045	-0.478	0.406	0.851	0.962	No association
Not-Distracting | HC | EC | beta	9	RPO	0.011	-0.433	0.452	0.962	0.962	No association
Not-Distracting | HC | EC | beta	9	CF	-0.022	-0.460	0.425	0.928	0.962	No association
Not-Distracting | HC | EC | beta	9	CFP	-0.136	-0.546	0.326	0.567	0.962	No association
Not-Distracting | HC | EC | beta	9	CPO	-0.049	-0.481	0.402	0.837	0.962	No association
Not-Distracting | PD | EC | beta	9	LF	0.323	-0.170	0.686	0.191	0.654	No association
Not-Distracting | PD | EC | beta	9	RF	0.262	-0.234	0.649	0.295	0.654	No association
Not-Distracting | PD | EC | beta	9	LC	0.209	-0.286	0.616	0.405	0.654	No association
Not-Distracting | PD | EC | beta	9	RC	0.258	-0.237	0.647	0.301	0.654	No association
Not-Distracting | PD | EC | beta	9	LPO	0.101	-0.384	0.542	0.691	0.764	No association
Not-Distracting | PD | EC | beta	9	RPO	0.076	-0.405	0.524	0.764	0.764	No association
Not-Distracting | PD | EC | beta	9	CF	0.259	-0.236	0.648	0.299	0.654	No association
Not-Distracting | PD | EC | beta	9	CFP	0.196	-0.298	0.607	0.436	0.654	No association
Not-Distracting | PD | EC | beta	9	CPO	0.112	-0.374	0.550	0.658	0.764	No association
Not-Distracting | HC | EO | beta	9	LF	-0.132	-0.543	0.330	0.580	0.673	No association
Not-Distracting | HC | EO | beta	9	RF	-0.154	-0.558	0.310	0.517	0.673	No association
Not-Distracting | HC | EO | beta	9	LC	-0.036	-0.471	0.413	0.880	0.880	No association
Not-Distracting | HC | EO | beta	9	RC	-0.125	-0.538	0.336	0.599	0.673	No association
Not-Distracting | HC | EO | beta	9	LPO	-0.178	-0.575	0.287	0.452	0.673	No association

FDR_family	FDR_family_size	Region	partial_*r*_controlling_age	CI_95_lower	CI_95_upper	*p*_partial	*q*_FDR_partial	Evidence_level
Not-Distracting | HC | EO | beta	9	RPO	-0.147	-0.553	0.316	0.538	0.673	No association
Not-Distracting | HC | EO | beta	9	CF	-0.196	-0.588	0.270	0.408	0.673	No association
Not-Distracting | HC | EO | beta	9	CFP	-0.247	-0.622	0.220	0.294	0.673	No association
Not-Distracting | HC | EO | beta	9	CPO	-0.190	-0.583	0.276	0.423	0.673	No association
Not-Distracting | PD | EO | beta	9	LF	0.403	-0.078	0.732	0.097	0.180	No association
Not-Distracting | PD | EO | beta	9	RF	0.362	-0.126	0.709	0.140	0.180	No association
Not-Distracting | PD | EO | beta	9	LC	0.327	-0.165	0.689	0.185	0.185	No association
Not-Distracting | PD | EO | beta	9	RC	0.401	-0.082	0.731	0.100	0.180	No association
Not-Distracting | PD | EO | beta	9	LPO	0.354	-0.135	0.704	0.150	0.180	No association
Not-Distracting | PD | EO | beta	9	RPO	0.375	-0.111	0.717	0.125	0.180	No association
Not-Distracting | PD | EO | beta	9	CF	0.356	-0.133	0.705	0.147	0.180	No association
Not-Distracting | PD | EO | beta	9	CFP	0.345	-0.145	0.700	0.160	0.180	No association
Not-Distracting | PD | EO | beta	9	CPO	0.351	-0.138	0.703	0.153	0.180	No association
Not-Worrying | HC | EC | delta	9	LF	-0.208	-0.596	0.258	0.378	0.828	No association
Not-Worrying | HC | EC | delta	9	RF	-0.105	-0.523	0.354	0.660	0.828	No association
Not-Worrying | HC | EC | delta	9	LC	-0.152	-0.557	0.311	0.521	0.828	No association
Not-Worrying | HC | EC | delta	9	RC	-0.128	-0.540	0.334	0.591	0.828	No association
Not-Worrying | HC | EC | delta	9	LPO	-0.139	-0.548	0.324	0.560	0.828	No association
Not-Worrying | HC | EC | delta	9	RPO	-0.152	-0.557	0.311	0.522	0.828	No association
Not-Worrying | HC | EC | delta	9	CF	-0.042	-0.476	0.408	0.861	0.861	No association
Not-Worrying | HC | EC | delta	9	CFP	-0.080	-0.505	0.375	0.736	0.828	No association
Not-Worrying | HC | EC | delta	9	CPO	-0.114	-0.530	0.346	0.631	0.828	No association
Not-Worrying | PD | EC | delta	9	LF	-0.099	-0.541	0.385	0.695	0.765	No association
Not-Worrying | PD | EC | delta	9	RF	-0.096	-0.539	0.389	0.706	0.765	No association
Not-Worrying | PD | EC | delta	9	LC	-0.129	-0.562	0.360	0.610	0.765	No association
Not-Worrying | PD | EC | delta	9	RC	-0.088	-0.533	0.395	0.727	0.765	No association
Not-Worrying | PD | EC | delta	9	LPO	-0.325	-0.687	0.167	0.189	0.765	No association
Not-Worrying | PD | EC | delta	9	RPO	-0.330	-0.691	0.161	0.180	0.765	No association

FDR_family	FDR_family_size	Region	partial_*r*_controlling_age	CI_95_lower	CI_95_upper	*p*_partial	*q*_FDR_partial	Evidence_level
Not-Worrying | PD | EC | delta	9	CF	-0.118	-0.554	0.369	0.641	0.765	No association
Not-Worrying | PD | EC | delta	9	CFP	-0.076	-0.524	0.405	0.765	0.765	No association
Not-Worrying | PD | EC | delta	9	CPO	-0.280	-0.660	0.215	0.261	0.765	No association
Not-Worrying | HC | EO | delta	9	LF	-0.127	-0.539	0.335	0.594	0.793	No association
Not-Worrying | HC | EO | delta	9	RF	-0.150	-0.556	0.313	0.527	0.793	No association
Not-Worrying | HC | EO | delta	9	LC	-0.136	-0.545	0.326	0.569	0.793	No association
Not-Worrying | HC | EO | delta	9	RC	-0.174	-0.573	0.291	0.463	0.793	No association
Not-Worrying | HC | EO | delta	9	LPO	-0.176	-0.574	0.289	0.459	0.793	No association
Not-Worrying | HC | EO | delta	9	RPO	-0.179	-0.576	0.286	0.451	0.793	No association
Not-Worrying | HC | EO | delta	9	CF	0.011	-0.434	0.451	0.963	0.963	No association
Not-Worrying | HC | EO | delta	9	CFP	-0.027	-0.464	0.420	0.909	0.963	No association
Not-Worrying | HC | EO | delta	9	CPO	-0.119	-0.534	0.341	0.617	0.793	No association
Not-Worrying | PD | EO | delta	9	LF	-0.123	-0.558	0.365	0.627	0.774	No association
Not-Worrying | PD | EO | delta	9	RF	-0.073	-0.522	0.408	0.774	0.774	No association
Not-Worrying | PD | EO | delta	9	LC	-0.174	-0.593	0.318	0.489	0.774	No association
Not-Worrying | PD | EO | delta	9	RC	-0.141	-0.571	0.349	0.576	0.774	No association
Not-Worrying | PD | EO | delta	9	LPO	-0.218	-0.622	0.277	0.385	0.774	No association
Not-Worrying | PD | EO | delta	9	RPO	-0.220	-0.623	0.275	0.380	0.774	No association
Not-Worrying | PD | EO | delta	9	CF	-0.102	-0.543	0.383	0.688	0.774	No association
Not-Worrying | PD | EO | delta	9	CFP	-0.130	-0.563	0.359	0.608	0.774	No association
Not-Worrying | PD | EO | delta	9	CPO	-0.238	-0.635	0.257	0.341	0.774	No association
Not-Worrying | HC | EC | theta	9	LF	0.394	-0.059	0.712	0.086	0.206	No association
Not-Worrying | HC | EC | theta	9	RF	0.306	-0.158	0.659	0.189	0.243	No association
Not-Worrying | HC | EC | theta	9	LC	0.364	-0.094	0.695	0.115	0.206	No association
Not-Worrying | HC | EC | theta	9	RC	0.368	-0.089	0.697	0.111	0.206	No association
Not-Worrying | HC | EC | theta	9	LPO	0.288	-0.177	0.648	0.218	0.246	No association
Not-Worrying | HC | EC | theta	9	RPO	0.381	-0.074	0.705	0.098	0.206	No association
Not-Worrying | HC | EC | theta	9	CF	0.245	-0.221	0.620	0.297	0.297	No association

FDR_family	FDR_family_size	Region	partial_*r*_controlling_age	CI_95_lower	CI_95_upper	*p*_partial	*q*_FDR_partial	Evidence_level
Not-Worrying | HC | EC | theta	9	CFP	0.316	-0.147	0.666	0.174	0.243	No association
Not-Worrying | HC | EC | theta	9	CPO	0.413	-0.036	0.723	0.071	0.206	No association
Not-Worrying | PD | EC | theta	9	LF	0.344	-0.146	0.699	0.162	0.174	No association
Not-Worrying | PD | EC | theta	9	RF	0.371	-0.116	0.714	0.130	0.174	No association
Not-Worrying | PD | EC | theta	9	LC	0.497	0.039	0.782	0.036	0.096	Exploratory FDR-level .05 ≤ *q* < .10
Not-Worrying | PD | EC | theta	9	RC	0.529	0.083	0.799	0.024	0.096	Exploratory FDR-level .05 ≤ *q* < .10
Not-Worrying | PD | EC | theta	9	LPO	0.391	-0.092	0.726	0.108	0.174	No association
Not-Worrying | PD | EC | theta	9	RPO	0.335	-0.156	0.693	0.174	0.174	No association
Not-Worrying | PD | EC | theta	9	CF	0.356	-0.133	0.706	0.147	0.174	No association
Not-Worrying | PD | EC | theta	9	CFP	0.522	0.073	0.795	0.026	0.096	Exploratory FDR-level .05 ≤ *q* < .10
Not-Worrying | PD | EC | theta	9	CPO	0.483	0.020	0.775	0.043	0.096	Exploratory FDR-level .05 ≤ *q* < .10
Not-Worrying | HC | EO | theta	9	LF	0.271	-0.195	0.637	0.248	0.476	No association
Not-Worrying | HC | EO | theta	9	RF	0.188	-0.277	0.582	0.427	0.480	No association
Not-Worrying | HC | EO | theta	9	LC	0.325	-0.137	0.671	0.162	0.476	No association
Not-Worrying | HC | EO | theta	9	RC	0.299	-0.165	0.655	0.200	0.476	No association
Not-Worrying | HC | EO | theta	9	LPO	0.249	-0.217	0.623	0.289	0.476	No association
Not-Worrying | HC | EO | theta	9	RPO	0.256	-0.211	0.627	0.277	0.476	No association
Not-Worrying | HC | EO | theta	9	CF	0.104	-0.355	0.523	0.662	0.662	No association
Not-Worrying | HC | EO | theta	9	CFP	0.216	-0.250	0.601	0.360	0.476	No association
Not-Worrying | HC | EO | theta	9	CPO	0.212	-0.255	0.598	0.370	0.476	No association
Not-Worrying | PD | EO | theta	9	LF	0.003	-0.465	0.469	0.991	0.991	No association
Not-Worrying | PD | EO | theta	9	RF	0.104	-0.381	0.544	0.681	0.848	No association
Not-Worrying | PD | EO | theta	9	LC	0.212	-0.283	0.618	0.399	0.599	No association
Not-Worrying | PD | EO | theta	9	RC	0.288	-0.207	0.665	0.247	0.585	No association
Not-Worrying | PD | EO | theta	9	LPO	0.303	-0.191	0.674	0.222	0.585	No association

FDR_family	FDR_family_size	Region	partial_*r*_controlling_age	CI_95_lower	CI_95_upper	*p*_partial	*q*_FDR_partial	Evidence_level
Not-Worrying | PD | EO | theta	9	RPO	0.232	-0.264	0.630	0.355	0.599	No association
Not-Worrying | PD | EO | theta	9	CF	0.080	-0.402	0.527	0.753	0.848	No association
Not-Worrying | PD | EO | theta	9	CFP	0.280	-0.215	0.661	0.260	0.585	No association
Not-Worrying | PD | EO | theta	9	CPO	0.320	-0.172	0.685	0.195	0.585	No association
Not-Worrying | HC | EC | alpha	9	LF	0.034	-0.415	0.469	0.888	0.972	No association
Not-Worrying | HC | EC | alpha	9	RF	-0.061	-0.490	0.392	0.798	0.972	No association
Not-Worrying | HC | EC | alpha	9	LC	-0.008	-0.449	0.436	0.972	0.972	No association
Not-Worrying | HC | EC | alpha	9	RC	-0.043	-0.477	0.407	0.856	0.972	No association
Not-Worrying | HC | EC | alpha	9	LPO	-0.019	-0.457	0.427	0.938	0.972	No association
Not-Worrying | HC | EC | alpha	9	RPO	-0.010	-0.450	0.435	0.968	0.972	No association
Not-Worrying | HC | EC | alpha	9	CF	-0.083	-0.507	0.373	0.728	0.972	No association
Not-Worrying | HC | EC | alpha	9	CFP	-0.045	-0.478	0.406	0.850	0.972	No association
Not-Worrying | HC | EC | alpha	9	CPO	-0.055	-0.486	0.397	0.817	0.972	No association
Not-Worrying | PD | EC | alpha	9	LF	0.064	-0.415	0.516	0.799	0.998	No association
Not-Worrying | PD | EC | alpha	9	RF	0.066	-0.414	0.517	0.796	0.998	No association
Not-Worrying | PD | EC | alpha	9	LC	0.109	-0.377	0.548	0.666	0.998	No association
Not-Worrying | PD | EC | alpha	9	RC	-0.001	-0.467	0.466	0.998	0.998	No association
Not-Worrying | PD | EC | alpha	9	LPO	0.365	-0.123	0.711	0.137	0.692	No association
Not-Worrying | PD | EC | alpha	9	RPO	0.351	-0.139	0.702	0.154	0.692	No association
Not-Worrying | PD | EC | alpha	9	CF	0.112	-0.374	0.550	0.658	0.998	No association
Not-Worrying | PD | EC | alpha	9	CFP	0.030	-0.443	0.490	0.907	0.998	No association
Not-Worrying | PD | EC | alpha	9	CPO	0.286	-0.209	0.664	0.251	0.752	No association
Not-Worrying | HC | EO | alpha	9	LF	0.130	-0.331	0.542	0.584	0.961	No association
Not-Worrying | HC | EO | alpha	9	RF	0.066	-0.388	0.494	0.782	0.961	No association
Not-Worrying | HC | EO | alpha	9	LC	0.073	-0.382	0.499	0.760	0.961	No association
Not-Worrying | HC | EO | alpha	9	RC	0.074	-0.381	0.500	0.758	0.961	No association
Not-Worrying | HC | EO | alpha	9	LPO	0.101	-0.357	0.521	0.670	0.961	No association
Not-Worrying | HC | EO | alpha	9	RPO	0.093	-0.364	0.514	0.696	0.961	No association

FDR_family	FDR_family_size	Region	partial_*r*_controlling_age	CI_95_lower	CI_95_upper	*p*_partial	*q*_FDR_partial	Evidence_level
Not-Worrying | HC | EO | alpha	9	CF	0.012	-0.433	0.452	0.961	0.961	No association
Not-Worrying | HC | EO | alpha	9	CFP	-0.014	-0.454	0.431	0.952	0.961	No association
Not-Worrying | HC | EO | alpha	9	CPO	0.059	-0.394	0.489	0.806	0.961	No association
Not-Worrying | PD | EO | alpha	9	LF	0.114	-0.372	0.552	0.652	0.917	No association
Not-Worrying | PD | EO | alpha	9	RF	0.026	-0.446	0.487	0.917	0.917	No association
Not-Worrying | PD | EO | alpha	9	LC	0.189	-0.305	0.603	0.452	0.917	No association
Not-Worrying | PD | EO | alpha	9	RC	0.104	-0.381	0.545	0.680	0.917	No association
Not-Worrying | PD | EO | alpha	9	LPO	0.160	-0.331	0.584	0.525	0.917	No association
Not-Worrying | PD | EO | alpha	9	RPO	0.192	-0.302	0.605	0.445	0.917	No association
Not-Worrying | PD | EO | alpha	9	CF	0.032	-0.441	0.492	0.899	0.917	No association
Not-Worrying | PD | EO | alpha	9	CFP	0.081	-0.401	0.528	0.750	0.917	No association
Not-Worrying | PD | EO | alpha	9	CPO	0.202	-0.292	0.611	0.421	0.917	No association
Not-Worrying | HC | EC | beta	9	LF	0.200	-0.266	0.591	0.397	0.529	No association
Not-Worrying | HC | EC | beta	9	RF	0.179	-0.286	0.576	0.451	0.529	No association
Not-Worrying | HC | EC | beta	9	LC	0.260	-0.206	0.630	0.268	0.483	No association
Not-Worrying | HC | EC | beta	9	RC	0.281	-0.184	0.644	0.229	0.483	No association
Not-Worrying | HC | EC | beta	9	LPO	0.411	-0.038	0.722	0.072	0.245	No association
Not-Worrying | HC | EC | beta	9	RPO	0.399	-0.053	0.715	0.082	0.245	No association
Not-Worrying | HC | EC | beta	9	CF	0.060	-0.393	0.490	0.801	0.801	No association
Not-Worrying | HC | EC | beta	9	CFP	0.171	-0.293	0.571	0.470	0.529	No association
Not-Worrying | HC | EC | beta	9	CPO	0.421	-0.026	0.728	0.064	0.245	No association
Not-Worrying | PD | EC | beta	9	LF	-0.024	-0.485	0.448	0.926	0.926	No association
Not-Worrying | PD | EC | beta	9	RF	-0.059	-0.512	0.419	0.815	0.926	No association
Not-Worrying | PD | EC | beta	9	LC	-0.052	-0.506	0.425	0.838	0.926	No association
Not-Worrying | PD | EC | beta	9	RC	-0.028	-0.488	0.445	0.912	0.926	No association
Not-Worrying | PD | EC | beta	9	LPO	-0.132	-0.564	0.357	0.601	0.926	No association
Not-Worrying | PD | EC | beta	9	RPO	-0.205	-0.613	0.290	0.416	0.926	No association
Not-Worrying | PD | EC | beta	9	CF	-0.079	-0.526	0.403	0.756	0.926	No association

FDR_family	FDR_family_size	Region	partial_*r*_controlling_age	CI_95_lower	CI_95_upper	*p*_partial	*q*_FDR_partial	Evidence_level
Not-Worrying | PD | EC | beta	9	CFP	-0.105	-0.545	0.381	0.679	0.926	No association
Not-Worrying | PD | EC | beta	9	CPO	-0.134	-0.566	0.355	0.595	0.926	No association
Not-Worrying | HC | EO | beta	9	LF	0.024	-0.423	0.461	0.921	0.925	No association
Not-Worrying | HC | EO | beta	9	RF	0.140	-0.322	0.549	0.555	0.925	No association
Not-Worrying | HC | EO | beta	9	LC	0.023	-0.424	0.461	0.925	0.925	No association
Not-Worrying | HC | EO | beta	9	RC	0.121	-0.340	0.535	0.613	0.925	No association
Not-Worrying | HC | EO | beta	9	LPO	0.112	-0.347	0.529	0.637	0.925	No association
Not-Worrying | HC | EO | beta	9	RPO	0.143	-0.319	0.551	0.546	0.925	No association
Not-Worrying | HC | EO | beta	9	CF	-0.085	-0.508	0.372	0.723	0.925	No association
Not-Worrying | HC | EO | beta	9	CFP	-0.062	-0.491	0.391	0.796	0.925	No association
Not-Worrying | HC | EO | beta	9	CPO	0.064	-0.390	0.493	0.789	0.925	No association
Not-Worrying | PD | EO | beta	9	LF	0.110	-0.376	0.549	0.664	0.848	No association
Not-Worrying | PD | EO | beta	9	RF	0.049	-0.428	0.504	0.848	0.848	No association
Not-Worrying | PD | EO | beta	9	LC	0.081	-0.401	0.528	0.751	0.848	No association
Not-Worrying | PD | EO | beta	9	RC	0.062	-0.417	0.514	0.806	0.848	No association
Not-Worrying | PD | EO | beta	9	LPO	0.119	-0.368	0.555	0.638	0.848	No association
Not-Worrying | PD | EO | beta	9	RPO	0.082	-0.400	0.529	0.745	0.848	No association
Not-Worrying | PD | EO | beta	9	CF	0.087	-0.396	0.533	0.730	0.848	No association
Not-Worrying | PD | EO | beta	9	CFP	0.052	-0.425	0.507	0.836	0.848	No association
Not-Worrying | PD | EO | beta	9	CPO	0.106	-0.380	0.546	0.676	0.848	No association
Emotional Awareness | HC | EC | delta	9	LF	-0.129	-0.541	0.332	0.587	0.778	No association
Emotional Awareness | HC | EC | delta	9	RF	-0.138	-0.547	0.324	0.561	0.778	No association
Emotional Awareness | HC | EC | delta	9	LC	-0.097	-0.517	0.361	0.685	0.778	No association
Emotional Awareness | HC | EC | delta	9	RC	-0.067	-0.495	0.387	0.778	0.778	No association

FDR_family	FDR_family_size	Region	partial_*r*_controlling_age	CI_95_lower	CI_95_upper	*p*_partial	*q*_FDR_partial	Evidence_level
Emotional Awareness | HC | EC | delta	9	LPO	-0.111	-0.527	0.349	0.642	0.778	No association
Emotional Awareness | HC | EC | delta	9	RPO	-0.090	-0.512	0.367	0.705	0.778	No association
Emotional Awareness | HC | EC | delta	9	CF	-0.150	-0.556	0.313	0.527	0.778	No association
Emotional Awareness | HC | EC | delta	9	CFP	-0.069	-0.496	0.385	0.773	0.778	No association
Emotional Awareness | HC | EC | delta	9	CPO	-0.122	-0.536	0.339	0.608	0.778	No association
Emotional Awareness | PD | EC | delta	9	LF	-0.177	-0.595	0.316	0.482	0.627	No association
Emotional Awareness | PD | EC | delta	9	RF	-0.126	-0.560	0.362	0.619	0.627	No association
Emotional Awareness | PD | EC | delta	9	LC	-0.238	-0.635	0.257	0.341	0.627	No association
Emotional Awareness | PD | EC | delta	9	RC	-0.169	-0.589	0.324	0.503	0.627	No association
Emotional Awareness | PD | EC | delta	9	LPO	-0.151	-0.577	0.339	0.549	0.627	No association
Emotional Awareness | PD | EC | delta	9	RPO	-0.147	-0.574	0.344	0.561	0.627	No association
Emotional Awareness | PD | EC | delta	9	CF	-0.124	-0.558	0.364	0.625	0.627	No association
Emotional Awareness | PD | EC | delta	9	CFP	-0.230	-0.629	0.266	0.359	0.627	No association
Emotional Awareness | PD | EC | delta	9	CPO	-0.123	-0.558	0.365	0.627	0.627	No association
Emotional Awareness | HC | EO | delta	9	LF	-0.266	-0.634	0.201	0.258	0.514	No association
Emotional Awareness | HC | EO | delta	9	RF	-0.173	-0.571	0.292	0.467	0.514	No association
Emotional Awareness | HC | EO | delta	9	LC	-0.226	-0.608	0.241	0.339	0.514	No association

FDR_family	FDR_family_size	Region	partial_*r*_controlling_age	CI_95_lower	CI_95_upper	*p*_partial	*q*_FDR_partial	Evidence_level
Emotional Awareness | HC | EO | delta	9	RC	-0.155	-0.559	0.309	0.514	0.514	No association
Emotional Awareness | HC | EO | delta	9	LPO	-0.223	-0.605	0.244	0.346	0.514	No association
Emotional Awareness | HC | EO | delta	9	RPO	-0.192	-0.585	0.274	0.418	0.514	No association
Emotional Awareness | HC | EO | delta	9	CF	-0.314	-0.664	0.149	0.178	0.514	No association
Emotional Awareness | HC | EO | delta	9	CFP	-0.259	-0.629	0.207	0.270	0.514	No association
Emotional Awareness | HC | EO | delta	9	CPO	-0.261	-0.631	0.205	0.267	0.514	No association
Emotional Awareness | PD | EO | delta	9	LF	-0.148	-0.575	0.343	0.558	0.964	No association
Emotional Awareness | PD | EO | delta	9	RF	-0.200	-0.610	0.294	0.425	0.964	No association
Emotional Awareness | PD | EO | delta	9	LC	-0.108	-0.548	0.378	0.669	0.964	No association
Emotional Awareness | PD | EO | delta	9	RC	-0.161	-0.584	0.331	0.524	0.964	No association
Emotional Awareness | PD | EO | delta	9	LPO	0.038	-0.436	0.496	0.880	0.964	No association
Emotional Awareness | PD | EO | delta	9	RPO	0.012	-0.458	0.476	0.964	0.964	No association
Emotional Awareness | PD | EO | delta	9	CF	-0.172	-0.592	0.320	0.494	0.964	No association
Emotional Awareness | PD | EO | delta	9	CFP	-0.188	-0.602	0.305	0.454	0.964	No association
Emotional Awareness | PD | EO | delta	9	CPO	0.012	-0.458	0.476	0.964	0.964	No association
Emotional Awareness | HC | EC | theta	9	LF	-0.177	-0.575	0.288	0.455	0.584	No association
Emotional Awareness | HC | EC | theta	9	RF	-0.109	-0.526	0.350	0.646	0.646	No association

FDR_family	FDR_family_size	Region	partial_*r*_controlling_age	CI_95_lower	CI_95_upper	*p*_partial	*q*_FDR_partial	Evidence_level
Emotional Awareness | HC | EC | theta	9	LC	-0.303	-0.658	0.161	0.193	0.323	No association
Emotional Awareness | HC | EC | theta	9	RC	-0.290	-0.649	0.175	0.216	0.323	No association
Emotional Awareness | HC | EC | theta	9	LPO	-0.413	-0.723	0.037	0.071	0.277	No association
Emotional Awareness | HC | EC | theta	9	RPO	-0.387	-0.708	0.068	0.092	0.277	No association
Emotional Awareness | HC | EC | theta	9	CF	-0.111	-0.527	0.349	0.642	0.646	No association
Emotional Awareness | HC | EC | theta	9	CFP	-0.306	-0.659	0.158	0.189	0.323	No association
Emotional Awareness | HC | EC | theta	9	CPO	-0.444	-0.741	-0.002	0.050	0.277	Nominal $p < .05$ only
Emotional Awareness | PD | EC | theta	9	LF	0.132	-0.357	0.564	0.601	0.988	No association
Emotional Awareness | PD | EC | theta	9	RF	0.129	-0.359	0.562	0.610	0.988	No association
Emotional Awareness | PD | EC | theta	9	LC	0.045	-0.431	0.501	0.860	0.988	No association
Emotional Awareness | PD | EC | theta	9	RC	0.080	-0.402	0.527	0.753	0.988	No association
Emotional Awareness | PD | EC | theta	9	LPO	0.023	-0.449	0.485	0.928	0.988	No association
Emotional Awareness | PD | EC | theta	9	RPO	0.004	-0.464	0.470	0.988	0.988	No association
Emotional Awareness | PD | EC | theta	9	CF	0.165	-0.327	0.587	0.512	0.988	No association
Emotional Awareness | PD | EC | theta	9	CFP	0.117	-0.370	0.553	0.645	0.988	No association
Emotional Awareness | PD | EC | theta	9	CPO	0.075	-0.406	0.524	0.767	0.988	No association
Emotional Awareness | HC | EO | theta	9	LF	0.042	-0.408	0.476	0.861	0.861	No association

FDR_family	FDR_family_size	Region	partial_*r*_controlling_age	CI_95_lower	CI_95_upper	*p*_partial	*q*_FDR_partial	Evidence_level
Emotional Awareness | HC | EO | theta	9	RF	0.114	-0.346	0.530	0.631	0.847	No association
Emotional Awareness | HC | EO | theta	9	LC	-0.146	-0.553	0.317	0.538	0.847	No association
Emotional Awareness | HC | EO | theta	9	RC	-0.128	-0.540	0.334	0.592	0.847	No association
Emotional Awareness | HC | EO | theta	9	LPO	-0.141	-0.550	0.321	0.552	0.847	No association
Emotional Awareness | HC | EO | theta	9	RPO	-0.155	-0.559	0.308	0.513	0.847	No association
Emotional Awareness | HC | EO | theta	9	CF	0.213	-0.254	0.599	0.368	0.847	No association
Emotional Awareness | HC | EO | theta	9	CFP	-0.048	-0.480	0.403	0.841	0.861	No association
Emotional Awareness | HC | EO | theta	9	CPO	-0.105	-0.523	0.354	0.659	0.847	No association
Emotional Awareness | PD | EO | theta	9	LF	-0.158	-0.582	0.334	0.532	0.684	No association
Emotional Awareness | PD | EO | theta	9	RF	-0.076	-0.524	0.405	0.764	0.792	No association
Emotional Awareness | PD | EO | theta	9	LC	-0.224	-0.625	0.271	0.372	0.684	No association
Emotional Awareness | PD | EO | theta	9	RC	-0.202	-0.611	0.293	0.423	0.684	No association
Emotional Awareness | PD | EO | theta	9	LPO	-0.171	-0.591	0.321	0.497	0.684	No association
Emotional Awareness | PD | EO | theta	9	RPO	-0.191	-0.604	0.303	0.448	0.684	No association
Emotional Awareness | PD | EO | theta	9	CF	-0.067	-0.518	0.413	0.792	0.792	No association
Emotional Awareness | PD | EO | theta	9	CFP	-0.175	-0.593	0.318	0.488	0.684	No association
Emotional Awareness | PD | EO | theta	9	CPO	-0.194	-0.606	0.300	0.440	0.684	No association

FDR_family	FDR_family_size	Region	partial_*r*_controlling_age	CI_95_lower	CI_95_upper	*p*_partial	*q*_FDR_partial	Evidence_level
Emotional Awareness | HC | EC | alpha	9	LF	0.181	-0.284	0.577	0.445	0.727	No association
Emotional Awareness | HC | EC | alpha	9	RF	0.221	-0.245	0.605	0.348	0.727	No association
Emotional Awareness | HC | EC | alpha	9	LC	0.117	-0.344	0.532	0.625	0.727	No association
Emotional Awareness | HC | EC | alpha	9	RC	0.083	-0.373	0.507	0.728	0.728	No association
Emotional Awareness | HC | EC | alpha	9	LPO	0.172	-0.293	0.571	0.468	0.727	No association
Emotional Awareness | HC | EC | alpha	9	RPO	0.143	-0.320	0.551	0.548	0.727	No association
Emotional Awareness | HC | EC | alpha	9	CF	0.222	-0.244	0.605	0.346	0.727	No association
Emotional Awareness | HC | EC | alpha	9	CFP	0.109	-0.350	0.527	0.646	0.727	No association
Emotional Awareness | HC | EC | alpha	9	CPO	0.205	-0.261	0.594	0.385	0.727	No association
Emotional Awareness | PD | EC | alpha	9	LF	-0.114	-0.551	0.373	0.654	1.000	No association
Emotional Awareness | PD | EC | alpha	9	RF	-0.155	-0.580	0.336	0.539	1.000	No association
Emotional Awareness | PD | EC | alpha	9	LC	0.104	-0.381	0.545	0.680	1.000	No association
Emotional Awareness | PD | EC | alpha	9	RC	0.015	-0.455	0.479	0.952	1.000	No association
Emotional Awareness | PD | EC | alpha	9	LPO	0.025	-0.447	0.486	0.921	1.000	No association
Emotional Awareness | PD | EC | alpha	9	RPO	0.043	-0.432	0.500	0.864	1.000	No association
Emotional Awareness | PD | EC | alpha	9	CF	-0.185	-0.600	0.309	0.463	1.000	No association
Emotional Awareness | PD | EC | alpha	9	CFP	0.067	-0.413	0.518	0.791	1.000	No association

FDR_family	FDR_family_size	Region	partial_*r*_controlling_age	CI_95_lower	CI_95_upper	*p*_partial	*q*_FDR_partial	Evidence_level
Emotional Awareness | PD | EC | alpha	9	CPO	0.000	-0.467	0.467	1.000	1.000	No association
Emotional Awareness | HC | EO | alpha	9	LF	0.227	-0.240	0.608	0.336	0.576	No association
Emotional Awareness | HC | EO | alpha	9	RF	0.202	-0.264	0.592	0.393	0.576	No association
Emotional Awareness | HC | EO | alpha	9	LC	0.160	-0.304	0.563	0.499	0.576	No association
Emotional Awareness | HC | EO | alpha	9	RC	0.127	-0.334	0.539	0.593	0.593	No association
Emotional Awareness | HC | EO | alpha	9	LPO	0.156	-0.308	0.560	0.512	0.576	No association
Emotional Awareness | HC | EO | alpha	9	RPO	0.160	-0.304	0.562	0.502	0.576	No association
Emotional Awareness | HC | EO | alpha	9	CF	0.256	-0.210	0.628	0.275	0.576	No association
Emotional Awareness | HC | EO | alpha	9	CFP	0.213	-0.254	0.599	0.368	0.576	No association
Emotional Awareness | HC | EO | alpha	9	CPO	0.218	-0.249	0.602	0.357	0.576	No association
Emotional Awareness | PD | EO | alpha	9	LF	0.046	-0.430	0.502	0.856	0.886	No association
Emotional Awareness | PD | EO | alpha	9	RF	0.123	-0.364	0.558	0.625	0.886	No association
Emotional Awareness | PD | EO | alpha	9	LC	0.041	-0.434	0.498	0.872	0.886	No association
Emotional Awareness | PD | EO | alpha	9	RC	0.105	-0.381	0.545	0.679	0.886	No association
Emotional Awareness | PD | EO | alpha	9	LPO	-0.171	-0.591	0.322	0.497	0.886	No association
Emotional Awareness | PD | EO | alpha	9	RPO	-0.136	-0.567	0.353	0.590	0.886	No association
Emotional Awareness | PD | EO | alpha	9	CF	0.037	-0.438	0.495	0.886	0.886	No association

FDR_family	FDR_family_size	Region	partial_*r*_controlling_age	CI_95_lower	CI_95_upper	*p*_partial	*q*_FDR_partial	Evidence_level
Emotional Awareness | PD | EO | alpha	9	CFP	0.134	-0.355	0.565	0.597	0.886	No association
Emotional Awareness | PD | EO | alpha	9	CPO	-0.112	-0.550	0.374	0.657	0.886	No association
Emotional Awareness | HC | EC | beta	9	LF	0.166	-0.298	0.567	0.483	0.781	No association
Emotional Awareness | HC | EC | beta	9	RF	0.094	-0.364	0.515	0.695	0.781	No association
Emotional Awareness | HC | EC | beta	9	LC	0.205	-0.261	0.594	0.386	0.781	No association
Emotional Awareness | HC | EC | beta	9	RC	0.187	-0.279	0.581	0.431	0.781	No association
Emotional Awareness | HC | EC | beta	9	LPO	0.136	-0.326	0.546	0.568	0.781	No association
Emotional Awareness | HC | EC | beta	9	RPO	0.110	-0.349	0.527	0.643	0.781	No association
Emotional Awareness | HC | EC | beta	9	CF	0.115	-0.345	0.530	0.629	0.781	No association
Emotional Awareness | HC | EC | beta	9	CFP	0.148	-0.315	0.554	0.533	0.781	No association
Emotional Awareness | HC | EC | beta	9	CPO	0.058	-0.394	0.488	0.807	0.807	No association
Emotional Awareness | PD | EC | beta	9	LF	0.389	-0.095	0.724	0.110	0.158	No association
Emotional Awareness | PD | EC | beta	9	RF	0.369	-0.118	0.713	0.132	0.158	No association
Emotional Awareness | PD | EC | beta	9	LC	0.347	-0.143	0.701	0.158	0.158	No association
Emotional Awareness | PD | EC | beta	9	RC	0.364	-0.124	0.710	0.137	0.158	No association
Emotional Awareness | PD | EC | beta	9	LPO	0.361	-0.128	0.708	0.141	0.158	No association
Emotional Awareness | PD | EC | beta	9	RPO	0.417	-0.062	0.740	0.085	0.158	No association

FDR_family	FDR_family_size	Region	partial_*r*_controlling_age	CI_95_lower	CI_95_upper	*p*_partial	*q*_FDR_partial	Evidence_level
Emotional Awareness | PD | EC | beta	9	CF	0.387	-0.098	0.723	0.113	0.158	No association
Emotional Awareness | PD | EC | beta	9	CFP	0.402	-0.079	0.732	0.098	0.158	No association
Emotional Awareness | PD | EC | beta	9	CPO	0.415	-0.064	0.739	0.087	0.158	No association
Emotional Awareness | HC | EO | beta	9	LF	0.292	-0.173	0.650	0.212	0.250	No association
Emotional Awareness | HC | EO | beta	9	RF	0.138	-0.324	0.547	0.562	0.562	No association
Emotional Awareness | HC | EO | beta	9	LC	0.343	-0.117	0.682	0.138	0.250	No association
Emotional Awareness | HC | EO | beta	9	RC	0.286	-0.180	0.646	0.222	0.250	No association
Emotional Awareness | HC | EO | beta	9	LPO	0.366	-0.091	0.696	0.112	0.250	No association
Emotional Awareness | HC | EO | beta	9	RPO	0.297	-0.167	0.654	0.203	0.250	No association
Emotional Awareness | HC | EO | beta	9	CF	0.286	-0.179	0.647	0.221	0.250	No association
Emotional Awareness | HC | EO | beta	9	CFP	0.340	-0.120	0.680	0.142	0.250	No association
Emotional Awareness | HC | EO | beta	9	CPO	0.371	-0.086	0.699	0.107	0.250	No association
Emotional Awareness | PD | EO | beta	9	LF	0.217	-0.278	0.621	0.387	0.440	No association
Emotional Awareness | PD | EO | beta	9	RF	0.257	-0.239	0.646	0.304	0.440	No association
Emotional Awareness | PD | EO | beta	9	LC	0.197	-0.297	0.608	0.433	0.440	No association
Emotional Awareness | PD | EO | beta	9	RC	0.232	-0.263	0.631	0.354	0.440	No association
Emotional Awareness | PD | EO | beta	9	LPO	0.197	-0.297	0.608	0.434	0.440	No association

FDR_family	FDR_family_size	Region	partial_*r*_controlling_age	CI_95_lower	CI_95_upper	*p*_partial	*q*_FDR_partial	Evidence_level
Emotional Awareness | PD | EO | beta	9	RPO	0.253	-0.243	0.644	0.312	0.440	No association
Emotional Awareness | PD | EO | beta	9	CF	0.236	-0.260	0.633	0.346	0.440	No association
Emotional Awareness | PD | EO | beta	9	CFP	0.244	-0.252	0.638	0.329	0.440	No association
Emotional Awareness | PD | EO | beta	9	CPO	0.194	-0.300	0.606	0.440	0.440	No association
Trusting | HC | EC | delta	9	LF	0.036	-0.413	0.471	0.882	0.985	No association
Trusting | HC | EC | delta	9	RF	-0.006	-0.447	0.438	0.980	0.985	No association
Trusting | HC | EC | delta	9	LC	0.071	-0.384	0.498	0.767	0.985	No association
Trusting | HC | EC | delta	9	RC	0.058	-0.395	0.488	0.809	0.985	No association
Trusting | HC | EC | delta	9	LPO	0.146	-0.317	0.553	0.538	0.985	No association
Trusting | HC | EC | delta	9	RPO	0.105	-0.354	0.523	0.659	0.985	No association
Trusting | HC | EC | delta	9	CF	-0.004	-0.446	0.439	0.985	0.985	No association
Trusting | HC | EC | delta	9	CFP	0.006	-0.437	0.448	0.978	0.985	No association
Trusting | HC | EC | delta	9	CPO	0.130	-0.332	0.541	0.585	0.985	No association
Trusting | PD | EC | delta	9	LF	-0.143	-0.572	0.347	0.572	0.726	No association
Trusting | PD | EC | delta	9	RF	-0.143	-0.572	0.347	0.571	0.726	No association
Trusting | PD | EC | delta	9	LC	-0.175	-0.593	0.318	0.489	0.726	No association
Trusting | PD | EC | delta	9	RC	-0.115	-0.552	0.372	0.650	0.726	No association
Trusting | PD | EC | delta	9	LPO	-0.162	-0.585	0.330	0.521	0.726	No association
Trusting | PD | EC | delta	9	RPO	-0.089	-0.533	0.395	0.726	0.726	No association
Trusting | PD | EC | delta	9	CF	-0.160	-0.583	0.332	0.527	0.726	No association
Trusting | PD | EC | delta	9	CFP	-0.157	-0.582	0.334	0.533	0.726	No association
Trusting | PD | EC | delta	9	CPO	-0.157	-0.581	0.334	0.533	0.726	No association
Trusting | HC | EO | delta	9	LF	0.190	-0.276	0.583	0.423	0.464	No association
Trusting | HC | EO | delta	9	RF	0.194	-0.271	0.587	0.411	0.464	No association
Trusting | HC | EO | delta	9	LC	0.235	-0.231	0.614	0.318	0.464	No association

FDR_family	FDR_family_size	Region	partial_*r*_controlling_age	CI_95_lower	CI_95_upper	*p*_partial	*q*_FDR_partial	Evidence_level
Trusting | HC | EO | delta	9	RC	0.232	-0.234	0.612	0.324	0.464	No association
Trusting | HC | EO | delta	9	LPO	0.206	-0.261	0.594	0.384	0.464	No association
Trusting | HC | EO | delta	9	RPO	0.174	-0.291	0.572	0.464	0.464	No association
Trusting | HC | EO | delta	9	CF	0.177	-0.288	0.574	0.456	0.464	No association
Trusting | HC | EO | delta	9	CFP	0.180	-0.286	0.576	0.449	0.464	No association
Trusting | HC | EO | delta	9	CPO	0.250	-0.217	0.623	0.289	0.464	No association
Trusting | PD | EO | delta	9	LF	-0.186	-0.601	0.307	0.459	0.459	No association
Trusting | PD | EO | delta	9	RF	-0.223	-0.625	0.272	0.373	0.420	No association
Trusting | PD | EO | delta	9	LC	-0.305	-0.676	0.189	0.218	0.420	No association
Trusting | PD | EO | delta	9	RC	-0.240	-0.635	0.256	0.338	0.420	No association
Trusting | PD | EO | delta	9	LPO	-0.272	-0.655	0.224	0.276	0.420	No association
Trusting | PD | EO | delta	9	RPO	-0.234	-0.632	0.261	0.350	0.420	No association
Trusting | PD | EO | delta	9	CF	-0.234	-0.632	0.261	0.350	0.420	No association
Trusting | PD | EO | delta	9	CFP	-0.307	-0.677	0.187	0.215	0.420	No association
Trusting | PD | EO | delta	9	CPO	-0.325	-0.688	0.167	0.188	0.420	No association
Trusting | HC | EC | theta	9	LF	-0.065	-0.493	0.389	0.785	0.930	No association
Trusting | HC | EC | theta	9	RF	-0.021	-0.459	0.425	0.930	0.930	No association
Trusting | HC | EC | theta	9	LC	-0.146	-0.553	0.317	0.540	0.930	No association
Trusting | HC | EC | theta	9	RC	-0.109	-0.526	0.350	0.647	0.930	No association
Trusting | HC | EC | theta	9	LPO	-0.096	-0.517	0.362	0.687	0.930	No association
Trusting | HC | EC | theta	9	RPO	-0.120	-0.534	0.340	0.613	0.930	No association
Trusting | HC | EC | theta	9	CF	-0.029	-0.465	0.419	0.905	0.930	No association
Trusting | HC | EC | theta	9	CFP	-0.128	-0.540	0.333	0.590	0.930	No association
Trusting | HC | EC | theta	9	CPO	-0.162	-0.564	0.303	0.496	0.930	No association
Trusting | PD | EC | theta	9	LF	0.655	0.271	0.859	0.003	0.029	FDR-corrected $q < .05$
Trusting | PD | EC | theta	9	RF	0.574	0.146	0.821	0.013	0.036	FDR-corrected $q < .05$
Trusting | PD | EC | theta	9	LC	0.543	0.102	0.806	0.020	0.036	FDR-corrected $q < .05$
Trusting | PD | EC | theta	9	RC	0.552	0.114	0.810	0.018	0.036	FDR-corrected $q < .05$

FDR_family	FDR_family_size	Region	partial_*r*_controlling_age	CI_95_lower	CI_95_upper	*p*_partial	*q*_FDR_partial	Evidence_level
Trusting | PD | EC | theta	9	LPO	0.350	-0.140	0.702	0.155	0.155	No association
Trusting | PD | EC | theta	9	RPO	0.380	-0.105	0.719	0.120	0.135	No association
Trusting | PD | EC | theta	9	CF	0.586	0.163	0.827	0.011	0.036	FDR-corrected q < .05
Trusting | PD | EC | theta	9	CFP	0.529	0.082	0.799	0.024	0.036	FDR-corrected q < .05
Trusting | PD | EC | theta	9	CPO	0.430	-0.046	0.747	0.075	0.096	Exploratory FDR-level .05 ≤ q < .10
Trusting | HC | EO | theta	9	LF	-0.151	-0.556	0.313	0.526	0.526	No association
Trusting | HC | EO | theta	9	RF	-0.163	-0.565	0.301	0.491	0.526	No association
Trusting | HC | EO | theta	9	LC	-0.249	-0.623	0.217	0.289	0.448	No association
Trusting | HC | EO | theta	9	RC	-0.262	-0.632	0.204	0.264	0.448	No association
Trusting | HC | EO | theta	9	LPO	-0.397	-0.714	0.055	0.083	0.248	No association
Trusting | HC | EO | theta	9	RPO	-0.411	-0.722	0.039	0.072	0.248	No association
Trusting | HC | EO | theta	9	CF	-0.155	-0.559	0.308	0.513	0.526	No association
Trusting | HC | EO | theta	9	CFP	-0.244	-0.620	0.222	0.299	0.448	No association
Trusting | HC | EO | theta	9	CPO	-0.424	-0.730	0.023	0.062	0.248	No association
Trusting | PD | EO | theta	9	LF	0.382	-0.104	0.720	0.118	0.282	No association
Trusting | PD | EO | theta	9	RF	0.382	-0.103	0.720	0.118	0.282	No association
Trusting | PD | EO | theta	9	LC	0.306	-0.188	0.676	0.217	0.282	No association
Trusting | PD | EO | theta	9	RC	0.335	-0.156	0.694	0.174	0.282	No association
Trusting | PD | EO | theta	9	LPO	0.303	-0.191	0.674	0.222	0.282	No association
Trusting | PD | EO | theta	9	RPO	0.260	-0.236	0.648	0.298	0.298	No association
Trusting | PD | EO | theta	9	CF	0.337	-0.154	0.695	0.171	0.282	No association
Trusting | PD | EO | theta	9	CFP	0.286	-0.209	0.664	0.251	0.282	No association
Trusting | PD | EO | theta	9	CPO	0.322	-0.171	0.686	0.193	0.282	No association
Trusting | HC | EC | alpha	9	LF	0.000	-0.443	0.443	1.000	1.000	No association
Trusting | HC | EC | alpha	9	RF	0.018	-0.428	0.457	0.939	1.000	No association
Trusting | HC | EC | alpha	9	LC	0.029	-0.419	0.466	0.902	1.000	No association
Trusting | HC | EC | alpha	9	RC	0.008	-0.436	0.449	0.974	1.000	No association

FDR_family	FDR_family_size	Region	partial_*r*_controlling_age	CI_95_lower	CI_95_upper	*p*_partial	*q*_FDR_partial	Evidence_level
Trusting | HC | EC | alpha	9	LPO	-0.072	-0.499	0.383	0.763	1.000	No association
Trusting | HC | EC | alpha	9	RPO	-0.025	-0.463	0.422	0.915	1.000	No association
Trusting | HC | EC | alpha	9	CF	0.027	-0.420	0.464	0.909	1.000	No association
Trusting | HC | EC | alpha	9	CFP	0.060	-0.393	0.490	0.801	1.000	No association
Trusting | HC | EC | alpha	9	CPO	-0.042	-0.475	0.408	0.861	1.000	No association
Trusting | PD | EC | alpha	9	LF	-0.088	-0.533	0.395	0.728	0.999	No association
Trusting | PD | EC | alpha	9	RF	-0.142	-0.571	0.348	0.573	0.999	No association
Trusting | PD | EC | alpha	9	LC	-0.052	-0.507	0.425	0.837	0.999	No association
Trusting | PD | EC | alpha	9	RC	-0.104	-0.544	0.382	0.682	0.999	No association
Trusting | PD | EC | alpha	9	LPO	0.000	-0.467	0.467	0.999	0.999	No association
Trusting | PD | EC | alpha	9	RPO	-0.052	-0.507	0.425	0.837	0.999	No association
Trusting | PD | EC | alpha	9	CF	-0.115	-0.552	0.372	0.649	0.999	No association
Trusting | PD | EC | alpha	9	CFP	-0.035	-0.494	0.439	0.892	0.999	No association
Trusting | PD | EC | alpha	9	CPO	0.015	-0.455	0.479	0.953	0.999	No association
Trusting | HC | EO | alpha	9	LF	-0.010	-0.450	0.435	0.967	0.997	No association
Trusting | HC | EO | alpha	9	RF	-0.074	-0.500	0.381	0.756	0.997	No association
Trusting | HC | EO | alpha	9	LC	-0.006	-0.447	0.438	0.980	0.997	No association
Trusting | HC | EO | alpha	9	RC	-0.071	-0.498	0.383	0.765	0.997	No association
Trusting | HC | EO | alpha	9	LPO	0.035	-0.414	0.470	0.883	0.997	No association
Trusting | HC | EO | alpha	9	RPO	0.050	-0.402	0.482	0.835	0.997	No association
Trusting | HC | EO | alpha	9	CF	-0.019	-0.457	0.427	0.937	0.997	No association
Trusting | HC | EO | alpha	9	CFP	-0.005	-0.447	0.438	0.983	0.997	No association
Trusting | HC | EO | alpha	9	CPO	0.001	-0.442	0.443	0.997	0.997	No association
Trusting | PD | EO | alpha	9	LF	0.458	-0.011	0.762	0.056	0.297	No association
Trusting | PD | EO | alpha	9	RF	0.366	-0.121	0.711	0.135	0.297	No association
Trusting | PD | EO | alpha	9	LC	0.353	-0.136	0.704	0.150	0.297	No association
Trusting | PD | EO | alpha	9	RC	0.339	-0.152	0.696	0.169	0.297	No association
Trusting | PD | EO | alpha	9	LPO	0.216	-0.279	0.620	0.390	0.409	No association

FDR_family	FDR_family_size	Region	partial_*r*_controlling_age	CI_95_lower	CI_95_upper	*p*_partial	*q*_FDR_partial	Evidence_level
Trusting | PD | EO | alpha	9	RPO	0.208	-0.287	0.615	0.409	0.409	No association
Trusting | PD | EO | alpha	9	CF	0.297	-0.197	0.671	0.231	0.297	No association
Trusting | PD | EO | alpha	9	CFP	0.403	-0.078	0.732	0.097	0.297	No association
Trusting | PD | EO | alpha	9	CPO	0.315	-0.178	0.682	0.203	0.297	No association
Trusting | HC | EC | beta	9	LF	-0.045	-0.478	0.405	0.850	0.999	No association
Trusting | HC | EC | beta	9	RF	0.000	-0.443	0.442	0.999	0.999	No association
Trusting | HC | EC | beta	9	LC	-0.193	-0.586	0.273	0.415	0.933	No association
Trusting | HC | EC | beta	9	RC	-0.137	-0.546	0.326	0.566	0.999	No association
Trusting | HC | EC | beta	9	LPO	-0.263	-0.632	0.203	0.262	0.856	No association
Trusting | HC | EC | beta	9	RPO	-0.251	-0.624	0.215	0.285	0.856	No association
Trusting | HC | EC | beta	9	CF	-0.015	-0.455	0.430	0.949	0.999	No association
Trusting | HC | EC | beta	9	CFP	-0.103	-0.521	0.356	0.667	0.999	No association
Trusting | HC | EC | beta	9	CPO	-0.291	-0.650	0.174	0.213	0.856	No association
Trusting | PD | EC | beta	9	LF	0.095	-0.389	0.538	0.706	0.706	No association
Trusting | PD | EC | beta	9	RF	0.183	-0.310	0.599	0.467	0.551	No association
Trusting | PD | EC | beta	9	LC	0.283	-0.212	0.663	0.255	0.551	No association
Trusting | PD | EC | beta	9	RC	0.218	-0.277	0.621	0.386	0.551	No association
Trusting | PD | EC | beta	9	LPO	0.337	-0.154	0.695	0.171	0.551	No association
Trusting | PD | EC | beta	9	RPO	0.310	-0.183	0.679	0.211	0.551	No association
Trusting | PD | EC | beta	9	CF	0.174	-0.319	0.593	0.490	0.551	No association
Trusting | PD | EC | beta	9	CFP	0.226	-0.269	0.627	0.368	0.551	No association
Trusting | PD | EC | beta	9	CPO	0.331	-0.160	0.691	0.179	0.551	No association
Trusting | HC | EO | beta	9	LF	-0.228	-0.609	0.238	0.333	0.401	No association
Trusting | HC | EO | beta	9	RF	-0.231	-0.611	0.236	0.328	0.401	No association
Trusting | HC | EO | beta	9	LC	-0.284	-0.645	0.181	0.225	0.401	No association
Trusting | HC | EO | beta	9	RC	-0.283	-0.645	0.183	0.227	0.401	No association
Trusting | HC | EO | beta	9	LPO	-0.319	-0.667	0.144	0.170	0.401	No association
Trusting | HC | EO | beta	9	RPO	-0.327	-0.672	0.135	0.159	0.401	No association

FDR_family	FDR_family_size	Region	partial_*r*_controlling_age	CI_95_lower	CI_95_upper	*p*_partial	*q*_FDR_partial	Evidence_level
Trusting | HC | EO | beta	9	CF	-0.217	-0.602	0.250	0.359	0.401	No association
Trusting | HC | EO | beta	9	CFP	-0.199	-0.590	0.267	0.401	0.401	No association
Trusting | HC | EO | beta	9	CPO	-0.350	-0.686	0.109	0.130	0.401	No association
Trusting | PD | EO | beta	9	LF	-0.053	-0.508	0.424	0.833	0.921	No association
Trusting | PD | EO | beta	9	RF	0.025	-0.447	0.486	0.921	0.921	No association
Trusting | PD | EO | beta	9	LC	0.140	-0.350	0.569	0.581	0.921	No association
Trusting | PD | EO | beta	9	RC	0.040	-0.435	0.497	0.876	0.921	No association
Trusting | PD | EO | beta	9	LPO	0.153	-0.338	0.579	0.543	0.921	No association
Trusting | PD | EO | beta	9	RPO	0.076	-0.405	0.524	0.764	0.921	No association
Trusting | PD | EO | beta	9	CF	0.058	-0.420	0.511	0.818	0.921	No association
Trusting | PD | EO | beta	9	CFP	0.097	-0.388	0.539	0.703	0.921	No association
Trusting | PD | EO | beta	9	CPO	0.132	-0.357	0.564	0.603	0.921	No association

Note. Partial correlations were computed within each group while controlling for age. FDR correction was applied across the nine ROIs within each group, resting-state condition, frequency band, and self-report construct.

Supplementary Table S7B. RBP age-adjusted group × self-report interaction results. RBP Pearson correlation results with FDR correction, confidence intervals, and Bayes factors.

FDR_family	FDR_family_size	Region	Interaction_term	Interaction_beta	CI_95_lower	CI_95_upper	*p*_interaction	*q*_FDR_interaction	Age_beta	Age_*p*	Evidence_level
BPQ-VSF | EC | delta	9	LF	BPQ-VSF x Group	-0.022	-0.050	0.006	0.126	0.283	0.001	0.795	No association
BPQ-VSF | EC | delta	9	RF	BPQ-VSF x Group	-0.025	-0.053	0.002	0.070	0.283	0.001	0.771	No association
BPQ-VSF | EC | delta	9	LC	BPQ-VSF x Group	-0.020	-0.052	0.012	0.213	0.351	0.001	0.741	No association
BPQ-VSF | EC | delta	9	RC	BPQ-VSF x Group	-0.024	-0.055	0.007	0.122	0.283	0.001	0.687	No association
BPQ-VSF | EC | delta	9	LPO	BPQ-VSF x Group	-0.015	-0.051	0.020	0.383	0.427	0.002	0.454	No association
BPQ-VSF | EC | delta	9	RPO	BPQ-VSF x Group	-0.019	-0.053	0.015	0.273	0.351	0.002	0.475	No association
BPQ-VSF | EC | delta	9	CF	BPQ-VSF x Group	-0.022	-0.049	0.005	0.105	0.283	0.001	0.796	No association
BPQ-VSF | EC | delta	9	CFP	BPQ-VSF x Group	-0.016	-0.046	0.013	0.270	0.351	0.001	0.630	No association
BPQ-VSF | EC | delta	9	CPO	BPQ-VSF x Group	-0.013	-0.047	0.020	0.427	0.427	0.002	0.478	No association
BPQ-VSF | EO | delta	9	LF	BPQ-VSF x Group	-0.012	-0.033	0.010	0.289	0.390	-0.002	0.352	No association
BPQ-VSF | EO | delta	9	RF	BPQ-VSF x Group	-0.015	-0.036	0.006	0.161	0.390	-0.002	0.387	No association
BPQ-VSF | EO | delta	9	LC	BPQ-VSF x Group	-0.012	-0.034	0.009	0.253	0.390	-0.001	0.517	No association
BPQ-VSF | EO | delta	9	RC	BPQ-VSF x Group	-0.015	-0.035	0.006	0.151	0.390	-0.001	0.449	No association
BPQ-VSF | EO | delta	9	LPO	BPQ-VSF x Group	-0.012	-0.035	0.011	0.303	0.390	-0.001	0.730	No association
BPQ-VSF | EO | delta	9	RPO	BPQ-VSF x Group	-0.012	-0.035	0.011	0.285	0.390	-0.001	0.753	No association

FDR_family	FDR_family_size	Region	Interaction_term	Interaction_beta	CI_95_lower	CI_95_upper	*p*_interaction	*q*_FDR_interaction	Age_beta	Age_*p*	Evidence_level
BPQ-VSF | EO | delta	9	CF	BPQ-VSF x Group	-0.010	-0.029	0.009	0.279	0.390	-0.002	0.321	No association
BPQ-VSF | EO | delta	9	CFP	BPQ-VSF x Group	-0.007	-0.025	0.011	0.460	0.509	-0.001	0.532	No association
BPQ-VSF | EO | delta	9	CPO	BPQ-VSF x Group	-0.007	-0.029	0.015	0.509	0.509	-0.001	0.801	No association
BPQ-VSF | EC | theta	9	LF	BPQ-VSF x Group	0.005	-0.001	0.011	0.097	0.175	-0.001	0.246	No association
BPQ-VSF | EC | theta	9	RF	BPQ-VSF x Group	0.006	0.000	0.012	0.053	0.175	-0.001	0.269	No association
BPQ-VSF | EC | theta	9	LC	BPQ-VSF x Group	0.004	-0.002	0.009	0.169	0.217	0.000	0.391	No association
BPQ-VSF | EC | theta	9	RC	BPQ-VSF x Group	0.004	-0.001	0.010	0.123	0.185	0.000	0.475	No association
BPQ-VSF | EC | theta	9	LPO	BPQ-VSF x Group	0.005	-0.001	0.010	0.078	0.175	0.000	0.940	No association
BPQ-VSF | EC | theta	9	RPO	BPQ-VSF x Group	0.004	-0.001	0.009	0.081	0.175	0.000	0.993	No association
BPQ-VSF | EC | theta	9	CF	BPQ-VSF x Group	0.006	-0.001	0.013	0.074	0.175	-0.001	0.213	No association
BPQ-VSF | EC | theta	9	CFP	BPQ-VSF x Group	0.003	-0.003	0.008	0.313	0.313	-0.001	0.300	No association
BPQ-VSF | EC | theta	9	CPO	BPQ-VSF x Group	0.003	-0.002	0.007	0.280	0.313	0.000	0.925	No association
BPQ-VSF | EO | theta	9	LF	BPQ-VSF x Group	0.005	-0.001	0.010	0.086	0.332	-0.001	0.070	No association
BPQ-VSF | EO | theta	9	RF	BPQ-VSF x Group	0.005	0.000	0.011	0.065	0.332	-0.001	0.059	No association
BPQ-VSF | EO | theta	9	LC	BPQ-VSF x Group	0.003	-0.002	0.008	0.240	0.359	-0.001	0.064	No association
BPQ-VSF | EO | theta	9	RC	BPQ-VSF x Group	0.004	-0.001	0.009	0.114	0.332	-0.001	0.069	No association

FDR_family	FDR_family_size	Region	Interaction_term	Interaction_beta	CI_95_lower	CI_95_upper	*p*_interaction	*q*_FDR_interaction	Age_beta	Age_*p*	Evidence_level
BPQ-VSF | EO | theta	9	LPO	BPQ-VSF x Group	0.003	-0.002	0.007	0.282	0.363	-0.001	0.079	No association
BPQ-VSF | EO | theta	9	RPO	BPQ-VSF x Group	0.003	-0.001	0.007	0.185	0.332	-0.001	0.079	No association
BPQ-VSF | EO | theta	9	CF	BPQ-VSF x Group	0.004	-0.002	0.010	0.155	0.332	-0.001	0.047	No association
BPQ-VSF | EO | theta	9	CFP	BPQ-VSF x Group	0.002	-0.003	0.006	0.401	0.401	-0.001	0.043	No association
BPQ-VSF | EO | theta	9	CPO	BPQ-VSF x Group	0.002	-0.002	0.007	0.363	0.401	-0.001	0.119	No association
BPQ-VSF | EC | alpha	9	LF	BPQ-VSF x Group	0.003	-0.014	0.020	0.743	0.860	-0.003	0.074	No association
BPQ-VSF | EC | alpha	9	RF	BPQ-VSF x Group	0.005	-0.013	0.022	0.590	0.860	-0.003	0.097	No association
BPQ-VSF | EC | alpha	9	LC	BPQ-VSF x Group	0.006	-0.018	0.029	0.629	0.860	-0.003	0.150	No association
BPQ-VSF | EC | alpha	9	RC	BPQ-VSF x Group	0.009	-0.014	0.033	0.417	0.860	-0.003	0.134	No association
BPQ-VSF | EC | alpha	9	LPO	BPQ-VSF x Group	0.001	-0.029	0.032	0.936	0.936	-0.004	0.120	No association
BPQ-VSF | EC | alpha	9	RPO	BPQ-VSF x Group	0.007	-0.023	0.038	0.628	0.860	-0.004	0.144	No association
BPQ-VSF | EC | alpha	9	CF	BPQ-VSF x Group	0.003	-0.015	0.020	0.764	0.860	-0.003	0.101	No association
BPQ-VSF | EC | alpha	9	CFP	BPQ-VSF x Group	0.005	-0.018	0.029	0.652	0.860	-0.003	0.174	No association
BPQ-VSF | EC | alpha	9	CPO	BPQ-VSF x Group	0.005	-0.025	0.035	0.736	0.860	-0.004	0.157	No association
BPQ-VSF | EO | alpha	9	LF	BPQ-VSF x Group	0.002	-0.005	0.009	0.528	0.873	0.000	0.614	No association
BPQ-VSF | EO | alpha	9	RF	BPQ-VSF x Group	0.003	-0.003	0.010	0.329	0.873	0.000	0.423	No association

FDR_family	FDR_family_size	Region	Interaction_term	Interaction_beta	CI_95_lower	CI_95_upper	*p*_interaction	*q*_FDR_interaction	Age_beta	Age_*p*	Evidence_level
BPQ-VSF | EO | alpha	9	LC	BPQ-VSF x Group	0.002	-0.008	0.012	0.679	0.873	-0.001	0.503	No association
BPQ-VSF | EO | alpha	9	RC	BPQ-VSF x Group	0.003	-0.007	0.013	0.500	0.873	-0.001	0.384	No association
BPQ-VSF | EO | alpha	9	LPO	BPQ-VSF x Group	0.004	-0.011	0.020	0.593	0.873	-0.001	0.531	No association
BPQ-VSF | EO | alpha	9	RPO	BPQ-VSF x Group	0.005	-0.013	0.022	0.595	0.873	-0.001	0.470	No association
BPQ-VSF | EO | alpha	9	CF	BPQ-VSF x Group	0.001	-0.005	0.008	0.648	0.873	0.000	0.496	No association
BPQ-VSF | EO | alpha	9	CFP	BPQ-VSF x Group	0.000	-0.008	0.009	0.928	0.928	-0.001	0.342	No association
BPQ-VSF | EO | alpha	9	CPO	BPQ-VSF x Group	0.002	-0.012	0.016	0.794	0.893	-0.001	0.401	No association
BPQ-VSF | EC | beta	9	LF	BPQ-VSF x Group	0.013	0.001	0.026	0.038	0.080	0.003	0.016	Exploratory FDR-level $.05 \leq q < .10$
BPQ-VSF | EC | beta	9	RF	BPQ-VSF x Group	0.014	0.003	0.025	0.011	0.080	0.003	0.011	Exploratory FDR-level $.05 \leq q < .10$
BPQ-VSF | EC | beta	9	LC	BPQ-VSF x Group	0.010	-0.001	0.021	0.068	0.089	0.003	0.014	Exploratory FDR-level $.05 \leq q < .10$
BPQ-VSF | EC | beta	9	RC	BPQ-VSF x Group	0.010	0.000	0.020	0.045	0.080	0.002	0.008	Exploratory FDR-level $.05 \leq q < .10$
BPQ-VSF | EC | beta	9	LPO	BPQ-VSF x Group	0.009	0.001	0.018	0.038	0.080	0.002	0.013	Exploratory FDR-level $.05 \leq q < .10$
BPQ-VSF | EC | beta	9	RPO	BPQ-VSF x Group	0.007	-0.001	0.014	0.079	0.089	0.002	0.011	Exploratory FDR-level $.05 \leq q < .10$
BPQ-VSF | EC | beta	9	CF	BPQ-VSF x Group	0.013	0.002	0.024	0.020	0.080	0.003	0.008	Exploratory FDR-level $.05 \leq q < .10$
BPQ-VSF | EC | beta	9	CFP	BPQ-VSF x Group	0.008	-0.001	0.017	0.079	0.089	0.002	0.009	Exploratory FDR-level $.05 \leq q < .10$
BPQ-VSF | EC | beta	9	CPO	BPQ-VSF x Group	0.006	-0.002	0.013	0.127	0.127	0.002	0.013	No association

FDR_family	FDR_family_size	Region	Interaction_term	Interaction_beta	CI_95_lower	CI_95_upper	*p*_interaction	*q*_FDR_interaction	Age_beta	Age_*p*	Evidence_level
BPQ-VSF | EO | beta	9	LF	BPQ-VSF x Group	0.004	-0.012	0.021	0.578	0.578	0.003	0.038	No association
BPQ-VSF | EO | beta	9	RF	BPQ-VSF x Group	0.006	-0.007	0.020	0.365	0.550	0.003	0.013	No association
BPQ-VSF | EO | beta	9	LC	BPQ-VSF x Group	0.007	-0.006	0.021	0.270	0.550	0.003	0.028	No association
BPQ-VSF | EO | beta	9	RC	BPQ-VSF x Group	0.007	-0.004	0.018	0.176	0.550	0.003	0.004	No association
BPQ-VSF | EO | beta	9	LPO	BPQ-VSF x Group	0.005	-0.005	0.016	0.305	0.550	0.003	0.012	No association
BPQ-VSF | EO | beta	9	RPO	BPQ-VSF x Group	0.005	-0.004	0.014	0.291	0.550	0.003	0.003	No association
BPQ-VSF | EO | beta	9	CF	BPQ-VSF x Group	0.005	-0.008	0.018	0.464	0.550	0.003	0.008	No association
BPQ-VSF | EO | beta	9	CFP	BPQ-VSF x Group	0.004	-0.006	0.015	0.417	0.550	0.003	0.010	No association
BPQ-VSF | EO | beta	9	CPO	BPQ-VSF x Group	0.003	-0.006	0.012	0.489	0.550	0.002	0.009	No association
Not-Distracting | EC | delta	9	LF	Not-Distracting x Group	-0.017	-0.137	0.103	0.781	0.792	0.001	0.698	No association
Not-Distracting | EC | delta	9	RF	Not-Distracting x Group	-0.016	-0.136	0.104	0.792	0.792	0.001	0.623	No association
Not-Distracting | EC | delta	9	LC	Not-Distracting x Group	-0.020	-0.155	0.116	0.771	0.792	0.001	0.683	No association
Not-Distracting | EC | delta	9	RC	Not-Distracting x Group	-0.022	-0.156	0.111	0.736	0.792	0.002	0.551	No association
Not-Distracting | EC | delta	9	LPO	Not-Distracting x Group	-0.029	-0.176	0.119	0.697	0.792	0.003	0.408	No association

FDR_family	FDR_family_size	Region	Interaction_term	Interaction_beta	CI_95_lower	CI_95_upper	*p*_interaction	*q*_FDR_interaction	Age_beta	Age_*p*	Evidence_level
Not-Distracting | EC | delta	9	RPO	Not-Distracting x Group	-0.037	-0.181	0.106	0.600	0.792	0.003	0.377	No association
Not-Distracting | EC | delta	9	CF	Not-Distracting x Group	-0.015	-0.132	0.101	0.792	0.792	0.001	0.674	No association
Not-Distracting | EC | delta	9	CFP	Not-Distracting x Group	-0.028	-0.153	0.097	0.653	0.792	0.002	0.537	No association
Not-Distracting | EC | delta	9	CPO	Not-Distracting x Group	-0.034	-0.175	0.107	0.630	0.792	0.003	0.400	No association
Not-Distracting | EO | delta	9	LF	Not-Distracting x Group	-0.034	-0.128	0.060	0.468	0.619	-0.001	0.706	No association
Not-Distracting | EO | delta	9	RF	Not-Distracting x Group	-0.020	-0.114	0.073	0.660	0.660	-0.001	0.672	No association
Not-Distracting | EO | delta	9	LC	Not-Distracting x Group	-0.030	-0.122	0.062	0.512	0.619	-0.001	0.821	No association
Not-Distracting | EO | delta	9	RC	Not-Distracting x Group	-0.029	-0.118	0.060	0.515	0.619	-0.001	0.766	No association
Not-Distracting | EO | delta	9	LPO	Not-Distracting x Group	-0.036	-0.135	0.062	0.458	0.619	0.000	0.962	No association
Not-Distracting | EO | delta	9	RPO	Not-Distracting x Group	-0.033	-0.128	0.063	0.493	0.619	0.000	0.952	No association
Not-Distracting | EO | delta	9	CF	Not-Distracting x Group	-0.025	-0.108	0.059	0.551	0.619	-0.001	0.645	No association

FDR_family	FDR_family_size	Region	Interaction_term	Interaction_beta	CI_95_lower	CI_95_upper	*p*_interaction	*q*_FDR_interaction	Age_beta	Age_*p*	Evidence_level
Not-Distracting | EO | delta	9	CFP	Not-Distracting x Group	-0.029	-0.107	0.048	0.446	0.619	0.000	0.853	No association
Not-Distracting | EO | delta	9	CPO	Not-Distracting x Group	-0.030	-0.120	0.060	0.500	0.619	0.000	0.912	No association
Not-Distracting | EC | theta	9	LF	Not-Distracting x Group	-0.019	-0.044	0.006	0.137	0.184	0.000	0.909	No association
Not-Distracting | EC | theta	9	RF	Not-Distracting x Group	-0.020	-0.046	0.006	0.131	0.184	0.000	0.882	No association
Not-Distracting | EC | theta	9	LC	Not-Distracting x Group	-0.015	-0.036	0.006	0.164	0.184	0.000	0.876	No association
Not-Distracting | EC | theta	9	RC	Not-Distracting x Group	-0.018	-0.040	0.005	0.123	0.184	0.000	0.745	No association
Not-Distracting | EC | theta	9	LPO	Not-Distracting x Group	-0.011	-0.033	0.010	0.294	0.294	0.000	0.553	No association
Not-Distracting | EC | theta	9	RPO	Not-Distracting x Group	-0.015	-0.036	0.005	0.143	0.184	0.000	0.362	No association
Not-Distracting | EC | theta	9	CF	Not-Distracting x Group	-0.022	-0.049	0.006	0.115	0.184	0.000	0.873	No association
Not-Distracting | EC | theta	9	CFP	Not-Distracting x Group	-0.017	-0.039	0.004	0.108	0.184	0.000	0.870	No association
Not-Distracting | EC | theta	9	CPO	Not-Distracting x Group	-0.013	-0.032	0.005	0.155	0.184	0.000	0.391	No association

FDR_family	FDR_family_size	Region	Interaction_term	Interaction_beta	CI_95_lower	CI_95_upper	*p*_interaction	*q*_FDR_interaction	Age_beta	Age_*p*	Evidence_level
Not-Distracting | EO | theta	9	LF	Not-Distracting x Group	-0.014	-0.037	0.010	0.253	0.676	-0.001	0.334	No association
Not-Distracting | EO | theta	9	RF	Not-Distracting x Group	-0.018	-0.042	0.006	0.139	0.676	-0.001	0.336	No association
Not-Distracting | EO | theta	9	LC	Not-Distracting x Group	-0.006	-0.026	0.015	0.588	0.688	-0.001	0.222	No association
Not-Distracting | EO | theta	9	RC	Not-Distracting x Group	-0.009	-0.029	0.011	0.375	0.676	-0.001	0.282	No association
Not-Distracting | EO | theta	9	LPO	Not-Distracting x Group	-0.004	-0.024	0.016	0.711	0.711	-0.001	0.212	No association
Not-Distracting | EO | theta	9	RPO	Not-Distracting x Group	-0.005	-0.023	0.013	0.569	0.688	-0.001	0.236	No association
Not-Distracting | EO | theta	9	CF	Not-Distracting x Group	-0.016	-0.040	0.007	0.165	0.676	-0.001	0.273	No association
Not-Distracting | EO | theta	9	CFP	Not-Distracting x Group	-0.008	-0.027	0.010	0.364	0.676	-0.001	0.200	No association
Not-Distracting | EO | theta	9	CPO	Not-Distracting x Group	-0.005	-0.024	0.014	0.612	0.688	0.000	0.300	No association
Not-Distracting | EC | alpha	9	LF	Not-Distracting x Group	0.016	-0.055	0.087	0.651	0.651	-0.003	0.074	No association
Not-Distracting | EC | alpha	9	RF	Not-Distracting x Group	0.028	-0.043	0.099	0.429	0.638	-0.003	0.057	No association

**FDR_family**	**FDR_family_size**	**Region**	**Interaction_term**	**Interaction_beta**	**CI_95_lower**	**CI_95_upper**	***p*_interaction**	***q*_FDR_interaction**	**Age_beta**	**Age_*p***	**Evidence_level**
Not-Distracting | EC | alpha	9	LC	Not-Distracting x Group	0.026	-0.071	0.122	0.593	0.651	-0.004	0.145	No association
Not-Distracting | EC | alpha	9	RC	Not-Distracting x Group	0.034	-0.063	0.130	0.486	0.638	-0.004	0.087	No association
Not-Distracting | EC | alpha	9	LPO	Not-Distracting x Group	0.043	-0.083	0.168	0.496	0.638	-0.005	0.100	No association
Not-Distracting | EC | alpha	9	RPO	Not-Distracting x Group	0.059	-0.065	0.183	0.338	0.638	-0.005	0.082	No association
Not-Distracting | EC | alpha	9	CF	Not-Distracting x Group	0.028	-0.044	0.101	0.433	0.638	-0.003	0.059	No association
Not-Distracting | EC | alpha	9	CFP	Not-Distracting x Group	0.039	-0.058	0.137	0.418	0.638	-0.004	0.109	No association
Not-Distracting | EC | alpha	9	CPO	Not-Distracting x Group	0.050	-0.072	0.171	0.413	0.638	-0.005	0.108	No association
Not-Distracting | EO | alpha	9	LF	Not-Distracting x Group	-0.009	-0.036	0.019	0.525	0.966	0.000	0.933	No association
Not-Distracting | EO | alpha	9	RF	Not-Distracting x Group	-0.005	-0.033	0.023	0.739	0.966	0.000	0.674	No association
Not-Distracting | EO | alpha	9	LC	Not-Distracting x Group	0.001	-0.038	0.040	0.966	0.966	0.000	0.750	No association
Not-Distracting | EO | alpha	9	RC	Not-Distracting x Group	0.004	-0.037	0.045	0.850	0.966	-0.001	0.493	No association

FDR_family	FDR_family_size	Region	Interaction_term	Interaction_beta	CI_95_lower	CI_95_upper	*p*_interaction	*q*_FDR_interaction	Age_beta	Age_*p*	Evidence_level
Not-Distracting | EO | alpha	9	LPO	Not-Distracting x Group	0.010	-0.055	0.074	0.764	0.966	-0.001	0.517	No association
Not-Distracting | EO | alpha	9	RPO	Not-Distracting x Group	0.012	-0.058	0.083	0.729	0.966	-0.001	0.438	No association
Not-Distracting | EO | alpha	9	CF	Not-Distracting x Group	-0.006	-0.033	0.021	0.647	0.966	0.000	0.747	No association
Not-Distracting | EO | alpha	9	CFP	Not-Distracting x Group	-0.002	-0.037	0.032	0.892	0.966	-0.001	0.552	No association
Not-Distracting | EO | alpha	9	CPO	Not-Distracting x Group	0.007	-0.051	0.065	0.801	0.966	-0.001	0.409	No association
Not-Distracting | EC | beta	9	LF	Not-Distracting x Group	0.021	-0.035	0.077	0.455	0.938	0.002	0.138	No association
Not-Distracting | EC | beta	9	RF	Not-Distracting x Group	0.010	-0.041	0.060	0.698	0.938	0.002	0.111	No association
Not-Distracting | EC | beta	9	LC	Not-Distracting x Group	0.010	-0.040	0.060	0.685	0.938	0.002	0.099	No association
Not-Distracting | EC | beta	9	RC	Not-Distracting x Group	0.008	-0.036	0.052	0.712	0.938	0.002	0.073	No association
Not-Distracting | EC | beta	9	LPO	Not-Distracting x Group	-0.002	-0.042	0.039	0.934	0.938	0.002	0.070	No association
Not-Distracting | EC | beta	9	RPO	Not-Distracting x Group	-0.006	-0.039	0.028	0.742	0.938	0.002	0.037	No association

FDR_family	FDR_family_size	Region	Interaction_term	Interaction_beta	CI_95_lower	CI_95_upper	*p*_interaction	*q*_FDR_interaction	Age_beta	Age_*p*	Evidence_level
Not-Distracting | EC | beta	9	CF	Not-Distracting x Group	0.010	-0.041	0.062	0.685	0.938	0.002	0.074	No association
Not-Distracting | EC | beta	9	CFP	Not-Distracting x Group	0.007	-0.033	0.048	0.710	0.938	0.002	0.061	No association
Not-Distracting | EC | beta	9	CPO	Not-Distracting x Group	-0.001	-0.034	0.032	0.938	0.938	0.002	0.062	No association
Not-Distracting | EO | beta	9	LF	Not-Distracting x Group	0.057	-0.010	0.123	0.092	0.193	0.001	0.386	No association
Not-Distracting | EO | beta	9	RF	Not-Distracting x Group	0.044	-0.014	0.101	0.135	0.193	0.002	0.193	No association
Not-Distracting | EO | beta	9	LC	Not-Distracting x Group	0.035	-0.021	0.091	0.217	0.217	0.001	0.290	No association
Not-Distracting | EO | beta	9	RC	Not-Distracting x Group	0.035	-0.013	0.082	0.150	0.193	0.002	0.110	No association
Not-Distracting | EO | beta	9	LPO	Not-Distracting x Group	0.030	-0.014	0.074	0.170	0.193	0.002	0.141	No association
Not-Distracting | EO | beta	9	RPO	Not-Distracting x Group	0.026	-0.012	0.063	0.171	0.193	0.002	0.058	No association
Not-Distracting | EO | beta	9	CF	Not-Distracting x Group	0.048	-0.006	0.102	0.082	0.193	0.002	0.168	No association
Not-Distracting | EO | beta	9	CFP	Not-Distracting x Group	0.041	-0.006	0.087	0.084	0.193	0.001	0.195	No association

FDR_family	FDR_family_size	Region	Interaction_term	Interaction_beta	CI_95_lower	CI_95_upper	*p*_interaction	*q*_FDR_interaction	Age_beta	Age_*p*	Evidence_level
Not-Distracting | EO | beta	9	CPO	Not-Distracting x Group	0.028	-0.011	0.067	0.149	0.193	0.001	0.126	No association
Not-Worrying | EC | delta	9	LF	Not-Worrying x Group	0.031	-0.105	0.167	0.647	0.933	0.001	0.775	No association
Not-Worrying | EC | delta	9	RF	Not-Worrying x Group	0.016	-0.121	0.154	0.810	0.933	0.001	0.734	No association
Not-Worrying | EC | delta	9	LC	Not-Worrying x Group	0.021	-0.133	0.175	0.782	0.933	0.001	0.661	No association
Not-Worrying | EC | delta	9	RC	Not-Worrying x Group	0.027	-0.125	0.180	0.717	0.933	0.001	0.649	No association
Not-Worrying | EC | delta	9	LPO	Not-Worrying x Group	-0.025	-0.192	0.142	0.766	0.933	0.003	0.300	No association
Not-Worrying | EC | delta	9	RPO	Not-Worrying x Group	-0.024	-0.187	0.138	0.763	0.933	0.003	0.333	No association
Not-Worrying | EC | delta	9	CF	Not-Worrying x Group	0.002	-0.132	0.136	0.974	0.974	0.001	0.758	No association
Not-Worrying | EC | delta	9	CFP	Not-Worrying x Group	0.015	-0.128	0.158	0.829	0.933	0.002	0.585	No association
Not-Worrying | EC | delta	9	CPO	Not-Worrying x Group	-0.023	-0.183	0.137	0.773	0.933	0.003	0.320	No association
Not-Worrying | EO | delta	9	LF	Not-Worrying x Group	0.004	-0.104	0.112	0.944	0.994	-0.001	0.601	No association
Not-Worrying | EO | delta	9	RF	Not-Worrying x Group	0.016	-0.090	0.123	0.757	0.994	-0.001	0.596	No association
Not-Worrying | EO | delta	9	LC	Not-Worrying x Group	-0.004	-0.110	0.101	0.934	0.994	-0.001	0.801	No association
Not-Worrying | EO | delta	9	RC	Not-Worrying x Group	0.010	-0.092	0.112	0.846	0.994	-0.001	0.662	No association
Not-Worrying | EO | delta	9	LPO	Not-Worrying x Group	0.000	-0.113	0.112	0.994	0.994	0.000	0.965	No association
Not-Worrying | EO | delta	9	RPO	Not-Worrying x Group	0.003	-0.106	0.111	0.957	0.994	0.000	0.928	No association

FDR_family	FDR_family_size	Region	Interaction_term	Interaction_beta	CI_95_lower	CI_95_upper	*p*_interaction	*q*_FDR_interaction	Age_beta	Age_*p*	Evidence_level
Not-Worrying | EO | delta	9	CF	Not-Worrying x Group	-0.011	-0.107	0.086	0.825	0.994	-0.001	0.593	No association
Not-Worrying | EO | delta	9	CFP	Not-Worrying x Group	-0.010	-0.100	0.079	0.815	0.994	0.000	0.867	No association
Not-Worrying | EO | delta	9	CPO	Not-Worrying x Group	-0.014	-0.117	0.089	0.783	0.994	0.000	0.922	No association
Not-Worrying | EC | theta	9	LF	Not-Worrying x Group	-0.003	-0.032	0.026	0.823	0.959	-0.001	0.145	No association
Not-Worrying | EC | theta	9	RF	Not-Worrying x Group	0.003	-0.028	0.033	0.861	0.959	-0.001	0.158	No association
Not-Worrying | EC | theta	9	LC	Not-Worrying x Group	0.001	-0.023	0.025	0.909	0.959	-0.001	0.237	No association
Not-Worrying | EC | theta	9	RC	Not-Worrying x Group	0.008	-0.017	0.033	0.523	0.959	-0.001	0.274	No association
Not-Worrying | EC | theta	9	LPO	Not-Worrying x Group	-0.001	-0.025	0.023	0.959	0.959	0.000	0.888	No association
Not-Worrying | EC | theta	9	RPO	Not-Worrying x Group	-0.002	-0.026	0.021	0.833	0.959	0.000	0.987	No association
Not-Worrying | EC | theta	9	CF	Not-Worrying x Group	0.003	-0.028	0.035	0.825	0.959	-0.001	0.138	No association
Not-Worrying | EC | theta	9	CFP	Not-Worrying x Group	0.006	-0.018	0.031	0.601	0.959	-0.001	0.146	No association
Not-Worrying | EC | theta	9	CPO	Not-Worrying x Group	-0.001	-0.022	0.020	0.924	0.959	0.000	0.764	No association
Not-Worrying | EO | theta	9	LF	Not-Worrying x Group	-0.012	-0.040	0.016	0.391	0.923	-0.001	0.070	No association
Not-Worrying | EO | theta	9	RF	Not-Worrying x Group	-0.005	-0.034	0.023	0.719	0.923	-0.001	0.060	No association
Not-Worrying | EO | theta	9	LC	Not-Worrying x Group	-0.006	-0.029	0.017	0.584	0.923	-0.001	0.036	No association
Not-Worrying | EO | theta	9	RC	Not-Worrying x Group	-0.003	-0.026	0.019	0.772	0.923	-0.001	0.051	No association

FDR_family	FDR_family_size	Region	Interaction_term	Interaction_beta	CI_95_lower	CI_95_upper	*p*_interaction	*q*_FDR_interaction	Age_beta	Age_*p*	Evidence_level
Not-Worrying | EO | theta	9	LPO	Not-Worrying x Group	0.001	-0.021	0.024	0.903	0.923	-0.001	0.034	No association
Not-Worrying | EO | theta	9	RPO	Not-Worrying x Group	-0.001	-0.022	0.019	0.887	0.923	-0.001	0.048	No association
Not-Worrying | EO | theta	9	CF	Not-Worrying x Group	-0.003	-0.031	0.025	0.834	0.923	-0.001	0.040	No association
Not-Worrying | EO | theta	9	CFP	Not-Worrying x Group	0.001	-0.020	0.022	0.923	0.923	-0.001	0.018	No association
Not-Worrying | EO | theta	9	CPO	Not-Worrying x Group	0.002	-0.019	0.023	0.845	0.923	-0.001	0.050	No association
Not-Worrying | EC | alpha	9	LF	Not-Worrying x Group	-0.005	-0.087	0.077	0.900	0.971	-0.003	0.097	No association
Not-Worrying | EC | alpha	9	RF	Not-Worrying x Group	0.004	-0.078	0.086	0.919	0.971	-0.003	0.112	No association
Not-Worrying | EC | alpha	9	LC	Not-Worrying x Group	0.005	-0.107	0.116	0.933	0.971	-0.003	0.147	No association
Not-Worrying | EC | alpha	9	RC	Not-Worrying x Group	-0.011	-0.122	0.101	0.847	0.971	-0.003	0.155	No association
Not-Worrying | EC | alpha	9	LPO	Not-Worrying x Group	0.063	-0.080	0.206	0.375	0.971	-0.005	0.062	No association
Not-Worrying | EC | alpha	9	RPO	Not-Worrying x Group	0.063	-0.078	0.205	0.371	0.971	-0.005	0.077	No association
Not-Worrying | EC | alpha	9	CF	Not-Worrying x Group	0.014	-0.070	0.098	0.736	0.971	-0.003	0.108	No association
Not-Worrying | EC | alpha	9	CFP	Not-Worrying x Group	0.002	-0.110	0.114	0.971	0.971	-0.003	0.184	No association
Not-Worrying | EC | alpha	9	CPO	Not-Worrying x Group	0.056	-0.083	0.195	0.421	0.971	-0.005	0.092	No association
Not-Worrying | EO | alpha	9	LF	Not-Worrying x Group	-0.005	-0.037	0.026	0.741	0.978	0.000	0.643	No association
Not-Worrying | EO | alpha	9	RF	Not-Worrying x Group	-0.006	-0.038	0.026	0.723	0.978	0.000	0.467	No association

FDR_family	FDR_family_size	Region	Interaction_term	Interaction_beta	CI_95_lower	CI_95_upper	*p*_interaction	*q*_FDR_interaction	Age_beta	Age_*p*	Evidence_level
Not-Worrying | EO | alpha	9	LC	Not-Worrying x Group	0.002	-0.043	0.048	0.912	0.978	-0.001	0.445	No association
Not-Worrying | EO | alpha	9	RC	Not-Worrying x Group	-0.003	-0.050	0.044	0.898	0.978	-0.001	0.363	No association
Not-Worrying | EO | alpha	9	LPO	Not-Worrying x Group	-0.003	-0.077	0.070	0.929	0.978	-0.001	0.527	No association
Not-Worrying | EO | alpha	9	RPO	Not-Worrying x Group	0.001	-0.080	0.082	0.978	0.978	-0.001	0.457	No association
Not-Worrying | EO | alpha	9	CF	Not-Worrying x Group	-0.004	-0.035	0.028	0.815	0.978	0.000	0.515	No association
Not-Worrying | EO | alpha	9	CFP	Not-Worrying x Group	0.001	-0.039	0.041	0.966	0.978	-0.001	0.326	No association
Not-Worrying | EO | alpha	9	CPO	Not-Worrying x Group	0.008	-0.058	0.074	0.811	0.978	-0.001	0.365	No association
Not-Worrying | EC | beta	9	LF	Not-Worrying x Group	-0.022	-0.087	0.043	0.497	0.509	0.003	0.032	No association
Not-Worrying | EC | beta	9	RF	Not-Worrying x Group	-0.023	-0.080	0.035	0.430	0.509	0.003	0.028	No association
Not-Worrying | EC | beta	9	LC	Not-Worrying x Group	-0.025	-0.082	0.031	0.366	0.509	0.002	0.031	No association
Not-Worrying | EC | beta	9	RC	Not-Worrying x Group	-0.023	-0.073	0.026	0.347	0.509	0.002	0.021	No association
Not-Worrying | EC | beta	9	LPO	Not-Worrying x Group	-0.036	-0.081	0.009	0.111	0.334	0.002	0.027	No association
Not-Worrying | EC | beta	9	RPO	Not-Worrying x Group	-0.035	-0.072	0.001	0.057	0.334	0.002	0.011	No association
Not-Worrying | EC | beta	9	CF	Not-Worrying x Group	-0.019	-0.078	0.039	0.509	0.509	0.003	0.018	No association
Not-Worrying | EC | beta	9	CFP	Not-Worrying x Group	-0.022	-0.068	0.023	0.328	0.509	0.002	0.020	No association
Not-Worrying | EC | beta	9	CPO	Not-Worrying x Group	-0.030	-0.066	0.006	0.095	0.334	0.002	0.022	No association

FDR_family	FDR_family_size	Region	Interaction_term	Interaction_beta	CI_95_lower	CI_95_upper	*p*_interaction	*q*_FDR_interaction	Age_beta	Age_*p*	Evidence_level
Not-Worrying | EO | beta	9	LF	Not-Worrying x Group	0.014	-0.065	0.094	0.715	0.927	0.002	0.118	No association
Not-Worrying | EO | beta	9	RF	Not-Worrying x Group	-0.005	-0.073	0.063	0.885	0.927	0.003	0.049	No association
Not-Worrying | EO | beta	9	LC	Not-Worrying x Group	0.009	-0.057	0.075	0.788	0.927	0.002	0.091	No association
Not-Worrying | EO | beta	9	RC	Not-Worrying x Group	-0.003	-0.059	0.053	0.919	0.927	0.003	0.021	No association
Not-Worrying | EO | beta	9	LPO	Not-Worrying x Group	0.002	-0.049	0.054	0.927	0.927	0.002	0.046	No association
Not-Worrying | EO | beta	9	RPO	Not-Worrying x Group	-0.003	-0.047	0.041	0.907	0.927	0.002	0.012	No association
Not-Worrying | EO | beta	9	CF	Not-Worrying x Group	0.018	-0.047	0.082	0.583	0.927	0.003	0.042	No association
Not-Worrying | EO | beta	9	CFP	Not-Worrying x Group	0.009	-0.046	0.064	0.738	0.927	0.002	0.052	No association
Not-Worrying | EO | beta	9	CPO	Not-Worrying x Group	0.004	-0.041	0.050	0.853	0.927	0.002	0.038	No association
Emotional Awareness | EC | delta	9	LF	Emotional Awareness x Group	-0.040	-0.150	0.071	0.471	0.640	0.000	0.963	No association
Emotional Awareness | EC | delta	9	RF	Emotional Awareness x Group	-0.029	-0.140	0.083	0.606	0.640	0.000	0.948	No association
Emotional Awareness | EC | delta	9	LC	Emotional Awareness x Group	-0.059	-0.183	0.064	0.336	0.640	0.000	0.965	No association
Emotional Awareness | EC | delta	9	RC	Emotional Awareness x Group	-0.054	-0.178	0.069	0.376	0.640	0.001	0.846	No association
Emotional Awareness | EC | delta	9	LPO	Emotional Awareness x Group	-0.046	-0.182	0.091	0.501	0.640	0.002	0.591	No association

FDR_family	FDR_family_size	Region	Interaction_term	Interaction_beta	CI_95_lower	CI_95_upper	*p*_interaction	*q*_FDR_interaction	Age_beta	Age_*p*	Evidence_level
Emotional Awareness | EC | delta	9	RPO	Emotional Awareness x Group	-0.048	-0.181	0.085	0.471	0.640	0.002	0.637	No association
Emotional Awareness | EC | delta	9	CF	Emotional Awareness x Group	-0.025	-0.133	0.083	0.640	0.640	0.000	0.993	No association
Emotional Awareness | EC | delta	9	CFP	Emotional Awareness x Group	-0.054	-0.169	0.060	0.343	0.640	0.001	0.826	No association
Emotional Awareness | EC | delta	9	CPO	Emotional Awareness x Group	-0.036	-0.167	0.095	0.582	0.640	0.002	0.586	No association
Emotional Awareness | EO | delta	9	LF	Emotional Awareness x Group	0.001	-0.086	0.088	0.986	0.987	-0.002	0.339	No association
Emotional Awareness | EO | delta	9	RF	Emotional Awareness x Group	-0.016	-0.102	0.070	0.711	0.987	-0.002	0.370	No association
Emotional Awareness | EO | delta	9	LC	Emotional Awareness x Group	0.001	-0.085	0.086	0.987	0.987	-0.001	0.502	No association
Emotional Awareness | EO | delta	9	RC	Emotional Awareness x Group	-0.016	-0.099	0.067	0.702	0.987	-0.002	0.416	No association
Emotional Awareness | EO | delta	9	LPO	Emotional Awareness x Group	0.017	-0.075	0.110	0.704	0.987	-0.001	0.737	No association
Emotional Awareness | EO | delta	9	RPO	Emotional Awareness x Group	0.011	-0.078	0.101	0.800	0.987	-0.001	0.701	No association
Emotional Awareness | EO | delta	9	CF	Emotional Awareness x Group	0.002	-0.074	0.079	0.953	0.987	-0.002	0.309	No association

FDR_family	FDR_family_size	Region	Interaction_term	Interaction_beta	CI_95_lower	CI_95_upper	*p*_interaction	*q*_FDR_interaction	Age_beta	Age_*p*	Evidence_level
Emotional Awareness | EO | delta	9	CFP	Emotional Awareness x Group	-0.008	-0.079	0.064	0.830	0.987	-0.001	0.506	No association
Emotional Awareness | EO | delta	9	CPO	Emotional Awareness x Group	0.018	-0.066	0.102	0.661	0.987	-0.001	0.781	No association
Emotional Awareness | EC | theta	9	LF	Emotional Awareness x Group	0.015	-0.010	0.039	0.228	0.293	-0.001	0.282	No association
Emotional Awareness | EC | theta	9	RF	Emotional Awareness x Group	0.013	-0.012	0.038	0.308	0.308	-0.001	0.341	No association
Emotional Awareness | EC | theta	9	LC	Emotional Awareness x Group	0.014	-0.006	0.034	0.154	0.242	0.000	0.377	No association
Emotional Awareness | EC | theta	9	RC	Emotional Awareness x Group	0.015	-0.006	0.037	0.161	0.242	0.000	0.566	No association
Emotional Awareness | EC | theta	9	LPO	Emotional Awareness x Group	0.016	-0.003	0.035	0.096	0.242	0.000	0.920	No association
Emotional Awareness | EC | theta	9	RPO	Emotional Awareness x Group	0.014	-0.005	0.033	0.139	0.242	0.000	0.947	No association
Emotional Awareness | EC | theta	9	CF	Emotional Awareness x Group	0.014	-0.012	0.041	0.278	0.308	-0.001	0.297	No association
Emotional Awareness | EC | theta	9	CFP	Emotional Awareness x Group	0.016	-0.004	0.037	0.118	0.242	-0.001	0.318	No association
Emotional Awareness | EC | theta	9	CPO	Emotional Awareness x Group	0.017	0.000	0.034	0.050	0.242	0.000	0.914	No association

FDR_family	FDR_family_size	Region	Interaction_term	Interaction_beta	CI_95_lower	CI_95_upper	*p*_interaction	*q*_FDR_interaction	Age_beta	Age_*p*	Evidence_level
Emotional Awareness | EO | theta	9	LF	Emotional Awareness x Group	-0.003	-0.026	0.020	0.794	0.974	-0.001	0.054	No association
Emotional Awareness | EO | theta	9	RF	Emotional Awareness x Group	-0.002	-0.025	0.022	0.873	0.974	-0.001	0.073	No association
Emotional Awareness | EO | theta	9	LC	Emotional Awareness x Group	0.001	-0.018	0.020	0.918	0.974	-0.001	0.032	No association
Emotional Awareness | EO | theta	9	RC	Emotional Awareness x Group	0.001	-0.018	0.020	0.914	0.974	-0.001	0.054	No association
Emotional Awareness | EO | theta	9	LPO	Emotional Awareness x Group	-0.001	-0.019	0.018	0.948	0.974	-0.001	0.042	No association
Emotional Awareness | EO | theta	9	RPO	Emotional Awareness x Group	0.000	-0.017	0.017	0.974	0.974	-0.001	0.046	No association
Emotional Awareness | EO | theta	9	CF	Emotional Awareness x Group	-0.004	-0.027	0.019	0.697	0.974	-0.001	0.051	No association
Emotional Awareness | EO | theta	9	CFP	Emotional Awareness x Group	-0.001	-0.019	0.016	0.881	0.974	-0.001	0.024	No association
Emotional Awareness | EO | theta	9	CPO	Emotional Awareness x Group	-0.001	-0.019	0.017	0.889	0.974	-0.001	0.061	No association
Emotional Awareness | EC | alpha	9	LF	Emotional Awareness x Group	-0.013	-0.080	0.053	0.684	0.961	-0.003	0.101	No association
Emotional Awareness | EC | alpha	9	RF	Emotional Awareness x Group	-0.022	-0.088	0.045	0.515	0.961	-0.003	0.112	No association

FDR_family	FDR_family_size	Region	Interaction_term	Interaction_beta	CI_95_lower	CI_95_upper	*p*_interaction	*q*_FDR_interaction	Age_beta	Age_*p*	Evidence_level
Emotional Awareness | EC | alpha	9	LC	Emotional Awareness x Group	0.019	-0.072	0.109	0.678	0.961	-0.003	0.228	No association
Emotional Awareness | EC | alpha	9	RC	Emotional Awareness x Group	0.012	-0.079	0.103	0.793	0.961	-0.003	0.164	No association
Emotional Awareness | EC | alpha	9	LPO	Emotional Awareness x Group	0.003	-0.115	0.121	0.961	0.961	-0.004	0.162	No association
Emotional Awareness | EC | alpha	9	RPO	Emotional Awareness x Group	0.011	-0.106	0.128	0.849	0.961	-0.004	0.198	No association
Emotional Awareness | EC | alpha	9	CF	Emotional Awareness x Group	-0.027	-0.095	0.041	0.426	0.961	-0.003	0.112	No association
Emotional Awareness | EC | alpha	9	CFP	Emotional Awareness x Group	0.008	-0.084	0.100	0.861	0.961	-0.003	0.236	No association
Emotional Awareness | EC | alpha	9	CPO	Emotional Awareness x Group	-0.008	-0.122	0.106	0.890	0.961	-0.004	0.196	No association
Emotional Awareness | EO | alpha	9	LF	Emotional Awareness x Group	0.000	-0.026	0.025	0.992	0.992	0.000	0.831	No association
Emotional Awareness | EO | alpha	9	RF	Emotional Awareness x Group	0.001	-0.025	0.027	0.932	0.992	0.000	0.616	No association
Emotional Awareness | EO | alpha	9	LC	Emotional Awareness x Group	0.003	-0.034	0.040	0.880	0.992	0.000	0.637	No association
Emotional Awareness | EO | alpha	9	RC	Emotional Awareness x Group	0.005	-0.033	0.044	0.776	0.992	-0.001	0.506	No association

**FDR_family**	**FDR_family_size**	**Region**	**Interaction_term**	**Interaction_beta**	**CI_95_lower**	**CI_95_upper**	***p*_interaction**	***q*_FDR_interaction**	**Age_beta**	**Age_*p***	**Evidence_level**
Emotional Awareness | EO | alpha	9	LPO	Emotional Awareness x Group	-0.014	-0.074	0.046	0.639	0.992	-0.001	0.565	No association
Emotional Awareness | EO | alpha	9	RPO	Emotional Awareness x Group	-0.014	-0.080	0.052	0.668	0.992	-0.001	0.538	No association
Emotional Awareness | EO | alpha	9	CF	Emotional Awareness x Group	-0.002	-0.027	0.023	0.868	0.992	0.000	0.640	No association
Emotional Awareness | EO | alpha	9	CFP	Emotional Awareness x Group	0.005	-0.028	0.037	0.775	0.992	-0.001	0.501	No association
Emotional Awareness | EO | alpha	9	CPO	Emotional Awareness x Group	-0.015	-0.069	0.039	0.582	0.992	-0.001	0.478	No association
Emotional Awareness | EC | beta	9	LF	Emotional Awareness x Group	0.037	-0.013	0.087	0.145	0.231	0.003	0.007	No association
Emotional Awareness | EC | beta	9	RF	Emotional Awareness x Group	0.036	-0.009	0.080	0.113	0.231	0.003	0.008	No association
Emotional Awareness | EC | beta	9	LC	Emotional Awareness x Group	0.024	-0.020	0.069	0.280	0.280	0.003	0.009	No association
Emotional Awareness | EC | beta	9	RC	Emotional Awareness x Group	0.025	-0.014	0.064	0.205	0.231	0.003	0.005	No association
Emotional Awareness | EC | beta	9	LPO	Emotional Awareness x Group	0.024	-0.012	0.060	0.182	0.231	0.002	0.012	No association
Emotional Awareness | EC | beta	9	RPO	Emotional Awareness x Group	0.020	-0.010	0.050	0.177	0.231	0.002	0.006	No association

FDR_family	FDR_family_size	Region	Interaction_term	Interaction_beta	CI_95_lower	CI_95_upper	*p*_interaction	*q*_FDR_interaction	Age_beta	Age_*p*	Evidence_level
Emotional Awareness | EC | beta	9	CF	Emotional Awareness x Group	0.036	-0.009	0.081	0.111	0.231	0.003	0.004	No association
Emotional Awareness | EC | beta	9	CFP	Emotional Awareness x Group	0.027	-0.008	0.063	0.127	0.231	0.003	0.005	No association
Emotional Awareness | EC | beta	9	CPO	Emotional Awareness x Group	0.024	-0.005	0.053	0.098	0.231	0.002	0.010	No association
Emotional Awareness | EO | beta	9	LF	Emotional Awareness x Group	0.003	-0.060	0.066	0.932	0.932	0.003	0.034	No association
Emotional Awareness | EO | beta	9	RF	Emotional Awareness x Group	0.017	-0.038	0.072	0.535	0.932	0.003	0.017	No association
Emotional Awareness | EO | beta	9	LC	Emotional Awareness x Group	-0.004	-0.056	0.048	0.868	0.932	0.003	0.026	No association
Emotional Awareness | EO | beta	9	RC	Emotional Awareness x Group	0.009	-0.035	0.054	0.673	0.932	0.003	0.005	No association
Emotional Awareness | EO | beta	9	LPO	Emotional Awareness x Group	-0.002	-0.042	0.039	0.926	0.932	0.003	0.010	No association
Emotional Awareness | EO | beta	9	RPO	Emotional Awareness x Group	0.004	-0.031	0.038	0.833	0.932	0.003	0.002	No association
Emotional Awareness | EO | beta	9	CF	Emotional Awareness x Group	0.005	-0.047	0.056	0.855	0.932	0.003	0.010	No association
Emotional Awareness | EO | beta	9	CFP	Emotional Awareness x Group	0.004	-0.039	0.048	0.835	0.932	0.003	0.012	No association

FDR_family	FDR_family_size	Region	Interaction_term	Interaction_beta	CI_95_lower	CI_95_upper	*p*_interaction	*q*_FDR_interaction	Age_beta	Age_*p*	Evidence_level
Emotional Awareness | EO | beta	9	CPO	Emotional Awareness x Group	-0.002	-0.037	0.034	0.928	0.932	0.002	0.008	No association
Trusting | EC | delta	9	LF	Trusting x Group	-0.031	-0.129	0.067	0.528	0.601	0.000	0.959	No association
Trusting | EC | delta	9	RF	Trusting x Group	-0.025	-0.124	0.073	0.601	0.601	0.000	0.954	No association
Trusting | EC | delta	9	LC	Trusting x Group	-0.044	-0.155	0.066	0.421	0.601	0.000	0.986	No association
Trusting | EC | delta	9	RC	Trusting x Group	-0.032	-0.142	0.077	0.551	0.601	0.000	0.881	No association
Trusting | EC | delta	9	LPO	Trusting x Group	-0.057	-0.177	0.063	0.341	0.601	0.001	0.774	No association
Trusting | EC | delta	9	RPO	Trusting x Group	-0.037	-0.155	0.081	0.533	0.601	0.001	0.710	No association
Trusting | EC | delta	9	CF	Trusting x Group	-0.027	-0.122	0.068	0.567	0.601	0.000	0.988	No association
Trusting | EC | delta	9	CFP	Trusting x Group	-0.030	-0.132	0.072	0.556	0.601	0.001	0.823	No association
Trusting | EC | delta	9	CPO	Trusting x Group	-0.052	-0.167	0.063	0.368	0.601	0.001	0.765	No association
Trusting | EO | delta	9	LF	Trusting x Group	-0.044	-0.121	0.034	0.261	0.261	-0.003	0.269	No association
Trusting | EO | delta	9	RF	Trusting x Group	-0.048	-0.124	0.029	0.212	0.258	-0.003	0.262	No association
Trusting | EO | delta	9	LC	Trusting x Group	-0.061	-0.136	0.013	0.101	0.258	-0.003	0.239	No association
Trusting | EO | delta	9	RC	Trusting x Group	-0.052	-0.124	0.021	0.157	0.258	-0.003	0.241	No association
Trusting | EO | delta	9	LPO	Trusting x Group	-0.058	-0.138	0.022	0.152	0.258	-0.002	0.372	No association
Trusting | EO | delta	9	RPO	Trusting x Group	-0.047	-0.125	0.031	0.230	0.258	-0.002	0.420	No association

FDR_family	FDR_family_size	Region	Interaction_term	Interaction_beta	CI_95_lower	CI_95_upper	*p*_interaction	*q*_FDR_interaction	Age_beta	Age_*p*	Evidence_level
Trusting | EO | delta	9	CF	Trusting x Group	-0.043	-0.111	0.026	0.213	0.258	-0.002	0.243	No association
Trusting | EO | delta	9	CFP	Trusting x Group	-0.049	-0.112	0.014	0.126	0.258	-0.002	0.313	No association
Trusting | EO | delta	9	CPO	Trusting x Group	-0.064	-0.136	0.009	0.082	0.258	-0.002	0.338	No association
Trusting | EC | theta	9	LF	Trusting x Group	0.024	0.004	0.044	0.020	0.075	0.000	0.980	Exploratory FDR-level .05 ≤ *q* < .10
Trusting | EC | theta	9	RF	Trusting x Group	0.021	0.001	0.042	0.045	0.075	0.000	0.956	Exploratory FDR-level .05 ≤ *q* < .10
Trusting | EC | theta	9	LC	Trusting x Group	0.018	0.001	0.035	0.039	0.075	0.000	0.780	Exploratory FDR-level .05 ≤ *q* < .10
Trusting | EC | theta	9	RC	Trusting x Group	0.021	0.003	0.038	0.022	0.075	0.000	0.545	Exploratory FDR-level .05 ≤ *q* < .10
Trusting | EC | theta	9	LPO	Trusting x Group	0.011	-0.006	0.028	0.208	0.208	0.000	0.470	No association
Trusting | EC | theta	9	RPO	Trusting x Group	0.014	-0.003	0.030	0.108	0.121	0.001	0.317	No association
Trusting | EC | theta	9	CF	Trusting x Group	0.022	0.000	0.043	0.050	0.075	0.000	0.856	Exploratory FDR-level .05 ≤ *q* < .10
Trusting | EC | theta	9	CFP	Trusting x Group	0.019	0.001	0.036	0.037	0.075	0.000	0.917	Exploratory FDR-level .05 ≤ *q* < .10
Trusting | EC | theta	9	CPO	Trusting x Group	0.014	-0.002	0.029	0.080	0.103	0.000	0.362	No association
Trusting | EO | theta	9	LF	Trusting x Group	0.017	-0.003	0.036	0.086	0.119	-0.001	0.345	No association
Trusting | EO | theta	9	RF	Trusting x Group	0.017	-0.002	0.037	0.085	0.119	-0.001	0.352	No association
Trusting | EO | theta	9	LC	Trusting x Group	0.013	-0.003	0.030	0.115	0.119	-0.001	0.303	No association
Trusting | EO | theta	9	RC	Trusting x Group	0.014	-0.002	0.030	0.092	0.119	0.000	0.427	No association

FDR_family	FDR_family_size	Region	Interaction_term	Interaction_beta	CI_95_lower	CI_95_upper	*p*_interaction	*q*_FDR_interaction	Age_beta	Age_*p*	Evidence_level
Trusting | EO | theta	9	LPO	Trusting x Group	0.016	0.001	0.032	0.041	0.119	0.000	0.457	Nominal *p* < .05 only
Trusting | EO | theta	9	RPO	Trusting x Group	0.014	0.000	0.029	0.048	0.119	0.000	0.472	Nominal *p* < .05 only
Trusting | EO | theta	9	CF	Trusting x Group	0.016	-0.004	0.035	0.117	0.119	-0.001	0.232	No association
Trusting | EO | theta	9	CFP	Trusting x Group	0.012	-0.003	0.027	0.119	0.119	-0.001	0.211	No association
Trusting | EO | theta	9	CPO	Trusting x Group	0.017	0.002	0.031	0.028	0.119	0.000	0.593	Nominal *p* < .05 only
Trusting | EC | alpha	9	LF	Trusting x Group	-0.007	-0.066	0.053	0.825	0.971	-0.003	0.102	No association
Trusting | EC | alpha	9	RF	Trusting x Group	-0.013	-0.072	0.046	0.662	0.971	-0.003	0.096	No association
Trusting | EC | alpha	9	LC	Trusting x Group	-0.007	-0.087	0.073	0.865	0.971	-0.003	0.166	No association
Trusting | EC | alpha	9	RC	Trusting x Group	-0.011	-0.092	0.070	0.780	0.971	-0.004	0.130	No association
Trusting | EC | alpha	9	LPO	Trusting x Group	0.013	-0.091	0.118	0.798	0.971	-0.004	0.188	No association
Trusting | EC | alpha	9	RPO	Trusting x Group	-0.002	-0.106	0.102	0.971	0.971	-0.004	0.176	No association
Trusting | EC | alpha	9	CF	Trusting x Group	-0.012	-0.072	0.049	0.699	0.971	-0.003	0.109	No association
Trusting | EC | alpha	9	CFP	Trusting x Group	-0.009	-0.091	0.072	0.821	0.971	-0.003	0.180	No association
Trusting | EC | alpha	9	CPO	Trusting x Group	0.011	-0.091	0.112	0.831	0.971	-0.004	0.220	No association
Trusting | EO | alpha	9	LF	Trusting x Group	0.015	-0.006	0.037	0.154	0.417	0.000	0.792	No association
Trusting | EO | alpha	9	RF	Trusting x Group	0.014	-0.009	0.036	0.223	0.417	0.000	0.895	No association

FDR_family	FDR_family_size	Region	Interaction_term	Interaction_beta	CI_95_lower	CI_95_upper	*p*_interaction	*q*_FDR_interaction	Age_beta	Age_*p*	Evidence_level
Trusting | EO | alpha	9	LC	Trusting x Group	0.019	-0.013	0.050	0.232	0.417	0.000	0.965	No association
Trusting | EO | alpha	9	RC	Trusting x Group	0.021	-0.013	0.054	0.219	0.417	0.000	0.821	No association
Trusting | EO | alpha	9	LPO	Trusting x Group	0.015	-0.037	0.067	0.564	0.632	0.000	0.802	No association
Trusting | EO | alpha	9	RPO	Trusting x Group	0.014	-0.044	0.071	0.632	0.632	-0.001	0.712	No association
Trusting | EO | alpha	9	CF	Trusting x Group	0.010	-0.012	0.032	0.344	0.442	0.000	0.836	No association
Trusting | EO | alpha	9	CFP	Trusting x Group	0.020	-0.008	0.047	0.152	0.417	0.000	0.814	No association
Trusting | EO | alpha	9	CPO	Trusting x Group	0.024	-0.023	0.070	0.309	0.442	0.000	0.820	No association
Trusting | EC | beta	9	LF	Trusting x Group	0.012	-0.035	0.059	0.616	0.616	0.003	0.036	No association
Trusting | EC | beta	9	RF	Trusting x Group	0.016	-0.025	0.057	0.446	0.514	0.003	0.025	No association
Trusting | EC | beta	9	LC	Trusting x Group	0.031	-0.009	0.071	0.129	0.291	0.003	0.010	No association
Trusting | EC | beta	9	RC	Trusting x Group	0.021	-0.015	0.057	0.251	0.401	0.003	0.011	No association
Trusting | EC | beta	9	LPO	Trusting x Group	0.031	-0.001	0.063	0.059	0.265	0.003	0.007	No association
Trusting | EC | beta	9	RPO	Trusting x Group	0.023	-0.004	0.050	0.090	0.270	0.002	0.005	No association
Trusting | EC | beta	9	CF	Trusting x Group	0.016	-0.026	0.057	0.457	0.514	0.003	0.018	No association
Trusting | EC | beta	9	CFP	Trusting x Group	0.018	-0.015	0.051	0.267	0.401	0.003	0.014	No association
Trusting | EC | beta	9	CPO	Trusting x Group	0.025	-0.001	0.051	0.055	0.265	0.002	0.006	No association

FDR_family	FDR_family_size	Region	Interaction_term	Interaction_beta	CI_95_lower	CI_95_upper	*p*_interaction	*q*_FDR_interaction	Age_beta	Age_*p*	Evidence_level
Trusting | EO | beta	9	LF	Trusting x Group	0.010	-0.047	0.067	0.730	0.730	0.003	0.087	No association
Trusting | EO | beta	9	RF	Trusting x Group	0.016	-0.034	0.065	0.524	0.589	0.003	0.033	No association
Trusting | EO | beta	9	LC	Trusting x Group	0.028	-0.019	0.075	0.241	0.589	0.003	0.028	No association
Trusting | EO | beta	9	RC	Trusting x Group	0.016	-0.025	0.056	0.434	0.589	0.003	0.012	No association
Trusting | EO | beta	9	LPO	Trusting x Group	0.025	-0.012	0.061	0.181	0.589	0.003	0.011	No association
Trusting | EO | beta	9	RPO	Trusting x Group	0.017	-0.014	0.049	0.267	0.589	0.003	0.004	No association
Trusting | EO | beta	9	CF	Trusting x Group	0.016	-0.031	0.063	0.493	0.589	0.003	0.023	No association
Trusting | EO | beta	9	CFP	Trusting x Group	0.016	-0.024	0.055	0.433	0.589	0.003	0.028	No association
Trusting | EO | beta	9	CPO	Trusting x Group	0.021	-0.011	0.054	0.186	0.589	0.003	0.009	No association

Note. Interaction models included self-report score, group, self-report × group interaction, and age as predictors. The interaction term tested whether the self-report–RBP association differed between PD and HC after controlling for age. FDR correction was applied across the nine ROIs within each self-report construct, resting-state condition, and frequency band.

Supplementary Figure S1

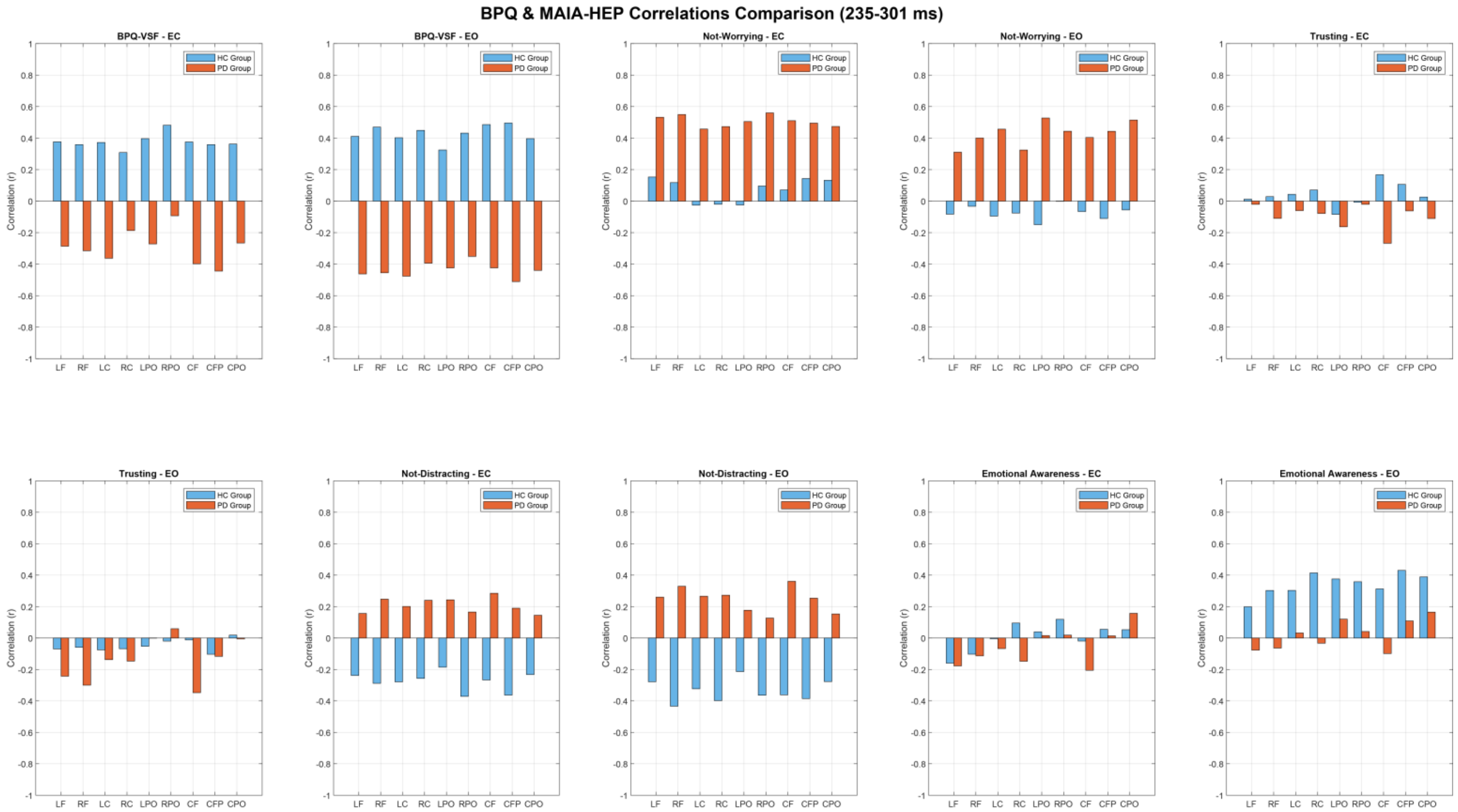

Supplementary Figure S1. Complete HEP correlation bar plots in the 235–301 ms time window. This figure shows Pearson correlation coefficients between self-reported interoceptive measures and mean HEP amplitudes across nine scalp ROIs for HC and PD under eyes-closed (EC) and eyes-open (EO) conditions. The displayed self-report constructs include BPQ-VSF and the MAIA dimensions selected for primary interpretation: Not-Worrying, Trusting, Not-Distracting, and Emotional Awareness. Bars indicate the direction and magnitude of Pearson correlations. Full statistics, including *r* values, 95% confidence intervals, *p* values, FDR-adjusted *q* values, Bayes factors, age-adjusted partial correlations, and group-interaction results, are provided in Supplementary Tables S4 and S5.

Supplementary Figure S2

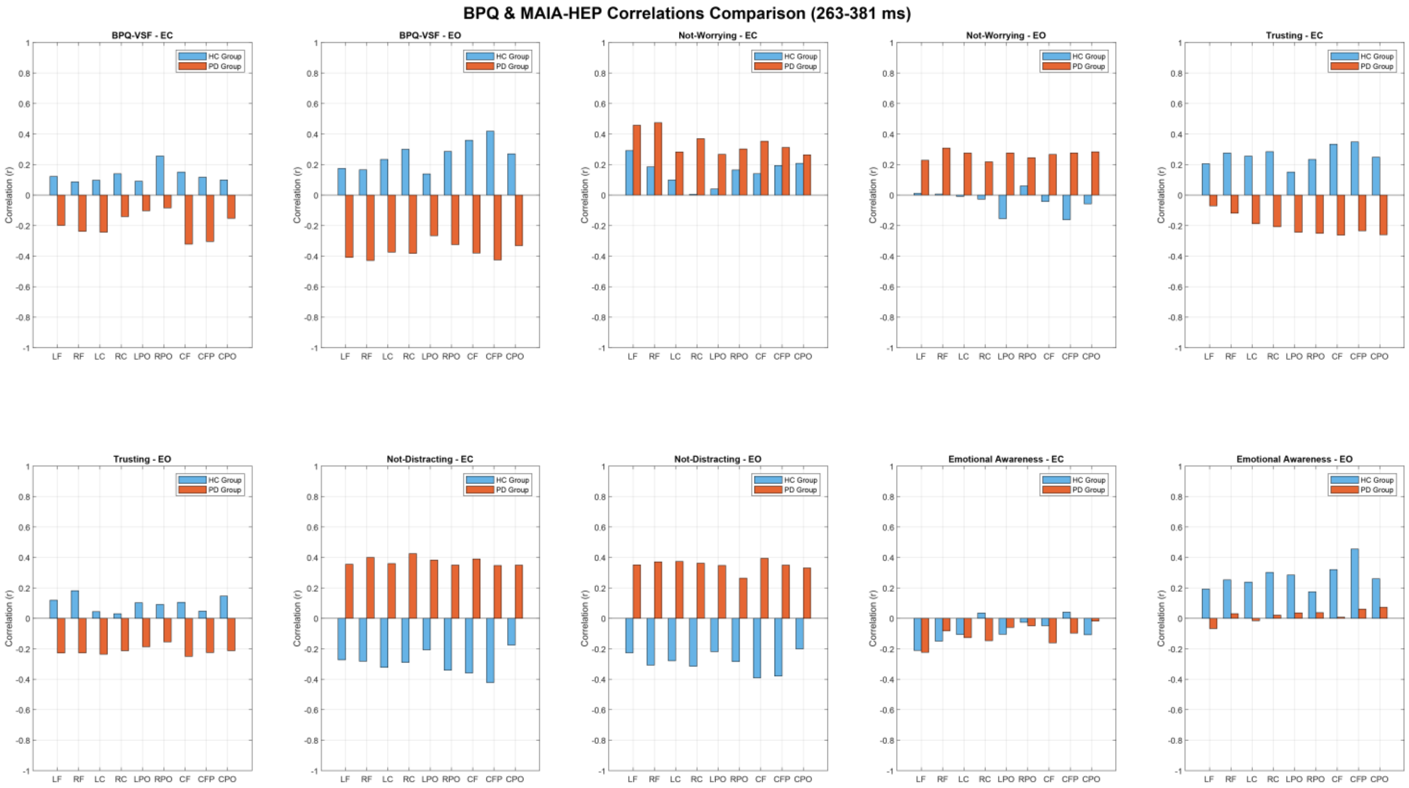

Supplementary Figure S2. Complete HEP correlation bar plots in the 263–381 ms time window. This later predefined HEP window is reported for completeness and was not the primary focus of the main-text interpretation. The figure shows Pearson correlation coefficients between self-reported interoceptive measures and mean HEP amplitudes across nine scalp ROIs for HC and PD under eyes-closed (EC) and eyes-open (EO) conditions. The displayed self-report constructs include BPQ-VSF and the MAIA dimensions selected for primary interpretation: Not-Worrying, Trusting, Not-Distracting, and Emotional Awareness. Full statistics, including *r* values, 95% confidence intervals, *p* values, FDR-adjusted *q* values, Bayes factors, age-adjusted partial correlations, and group-interaction results, are provided in Supplementary Tables S4 and S5.

Supplementary Figure S3

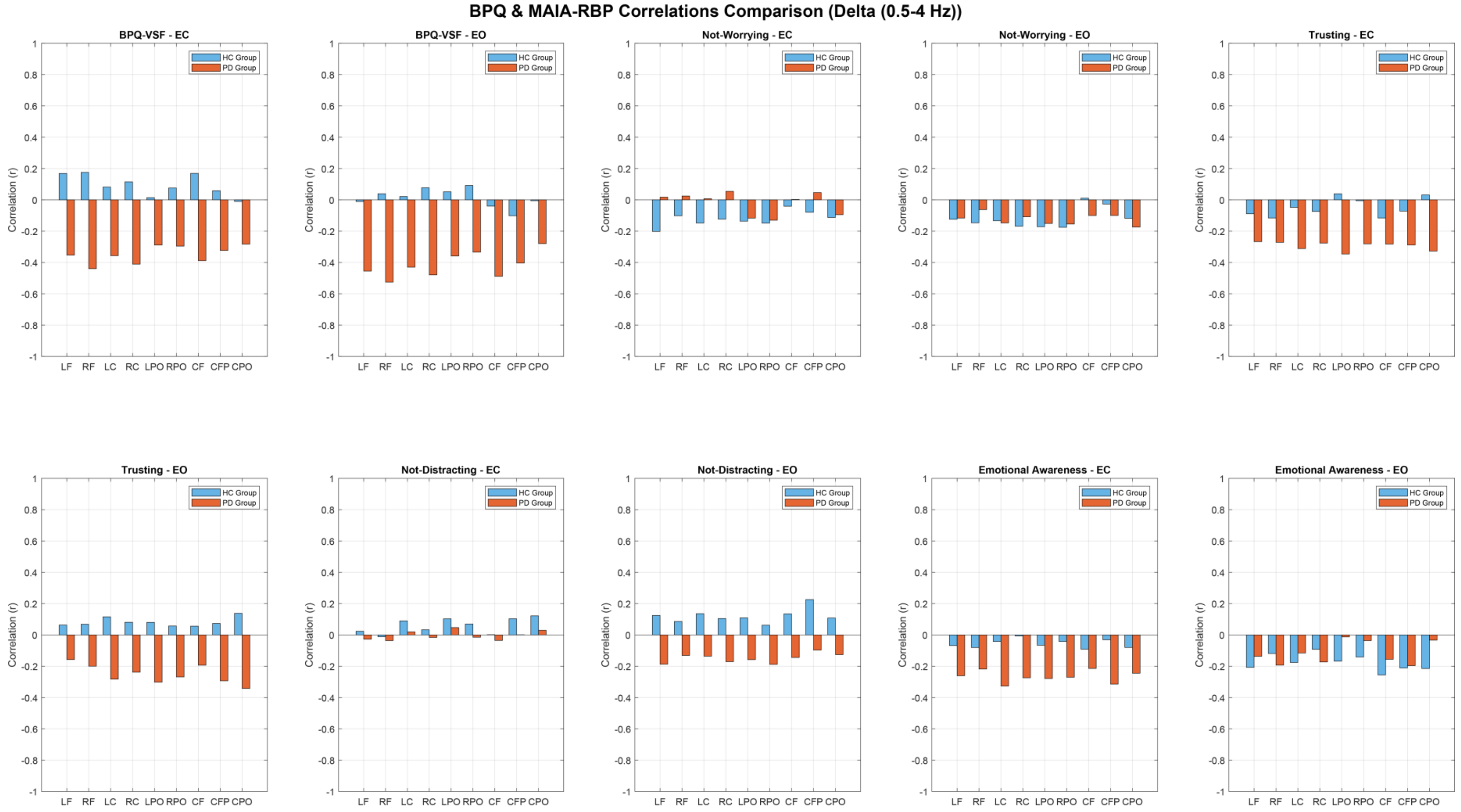

(A)

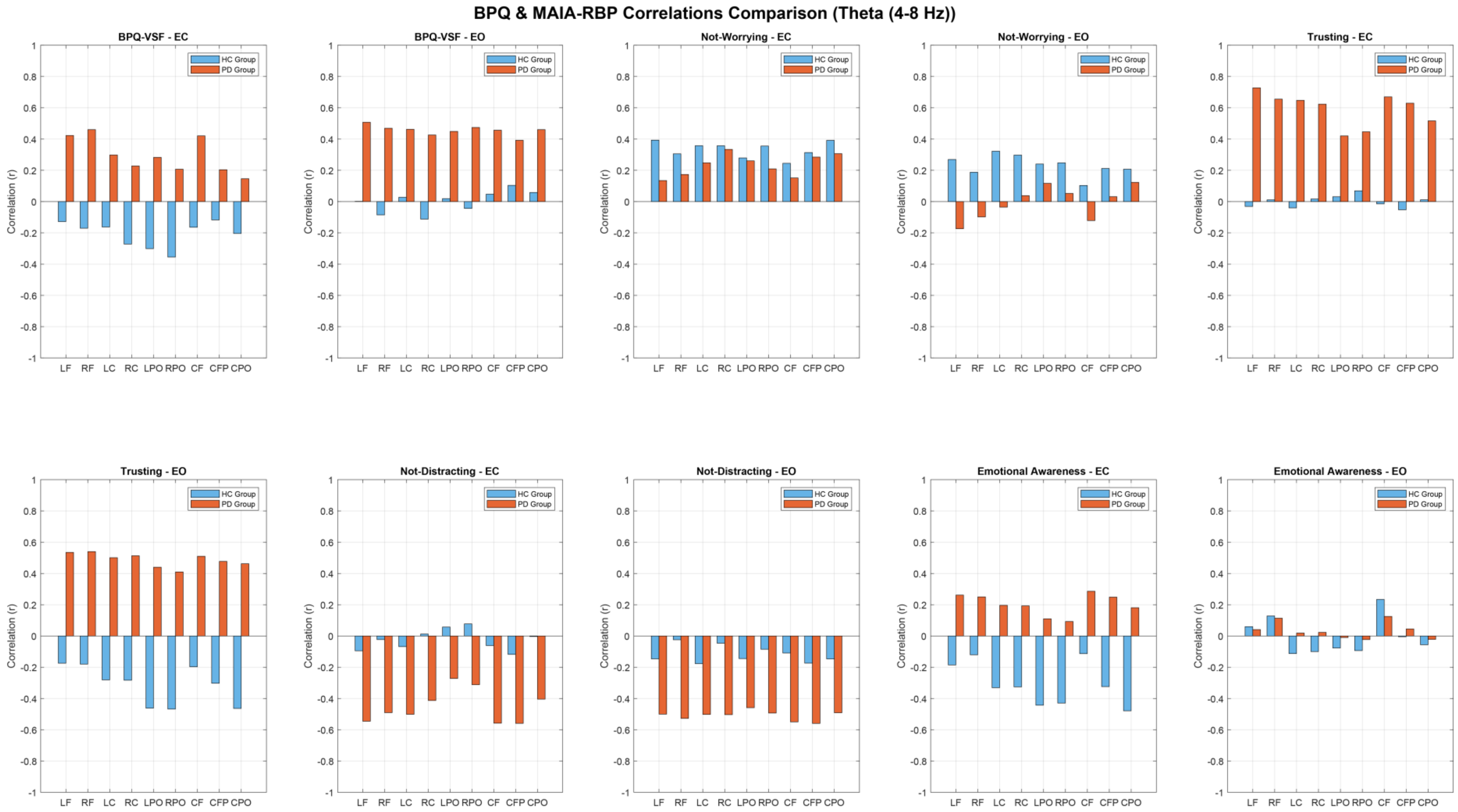
BPQ & MAIA-RBP Correlations Comparison (Theta (4-8 Hz))
BPQ-VSF - EC
BPQ-VSF - EO
Not-Worrying - EC
Not-Worrying - EO
Trusting - EC
Trusting - EO
Not-Distracting - EC
Not-Distracting - EO
Emotional Awareness - EC
Emotional Awareness - EO
HC Group
PD Group
Correlation (r)
LF RF LC RC LPO RPO CF CFP CPO

(B)

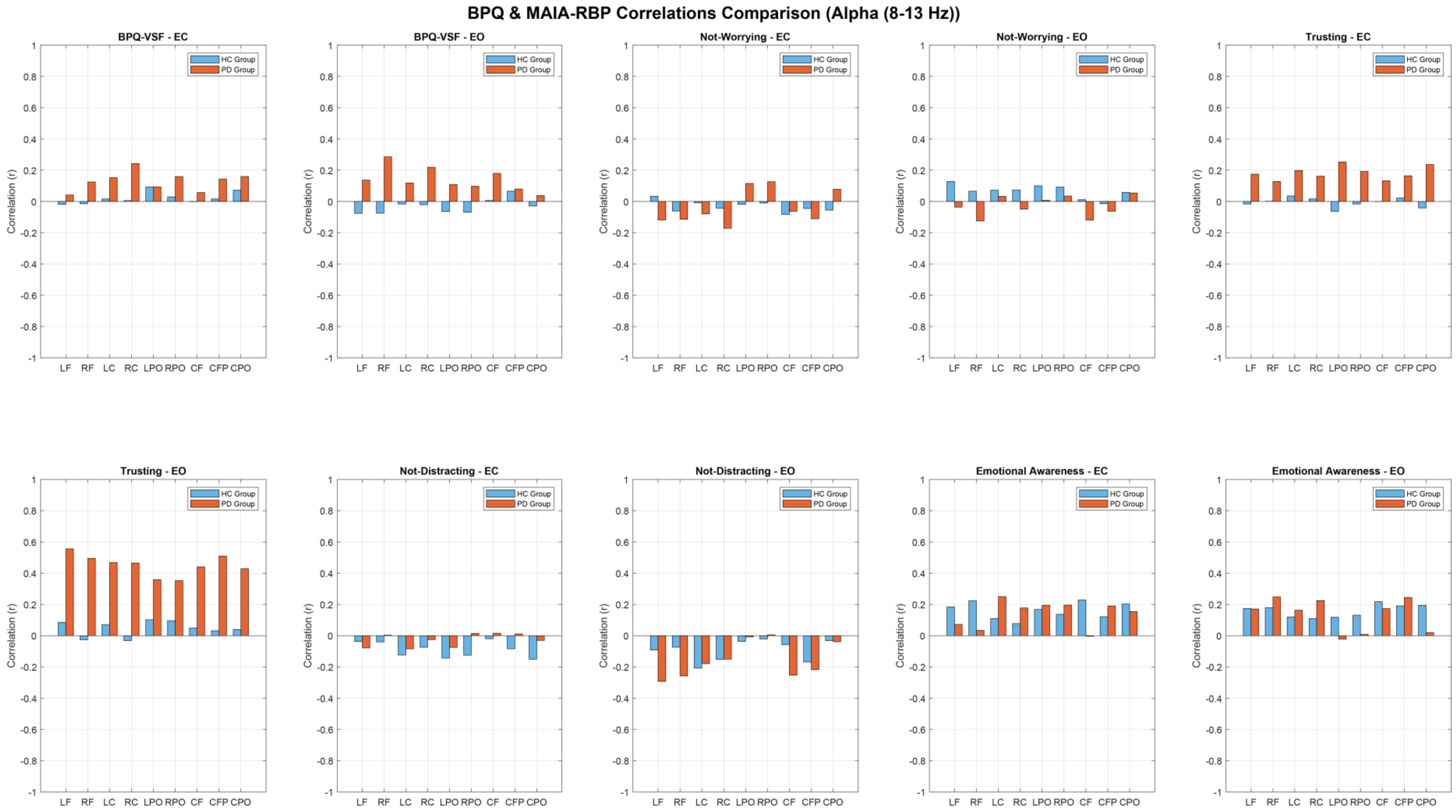
BPQ & MAIA-RBP Correlations Comparison (Alpha (8-13 Hz))
BPQ-VSF - EC
BPQ-VSF - EO
Not-Worrying - EC
Not-Worrying - EO
Trusting - EC
Trusting - EO
Not-Distracting - EC
Not-Distracting - EO
Emotional Awareness - EC
Emotional Awareness - EO
HC Group
PD Group
Correlation (r)
LF RF LC RC LPO RPO CF CFP CPO

(C)

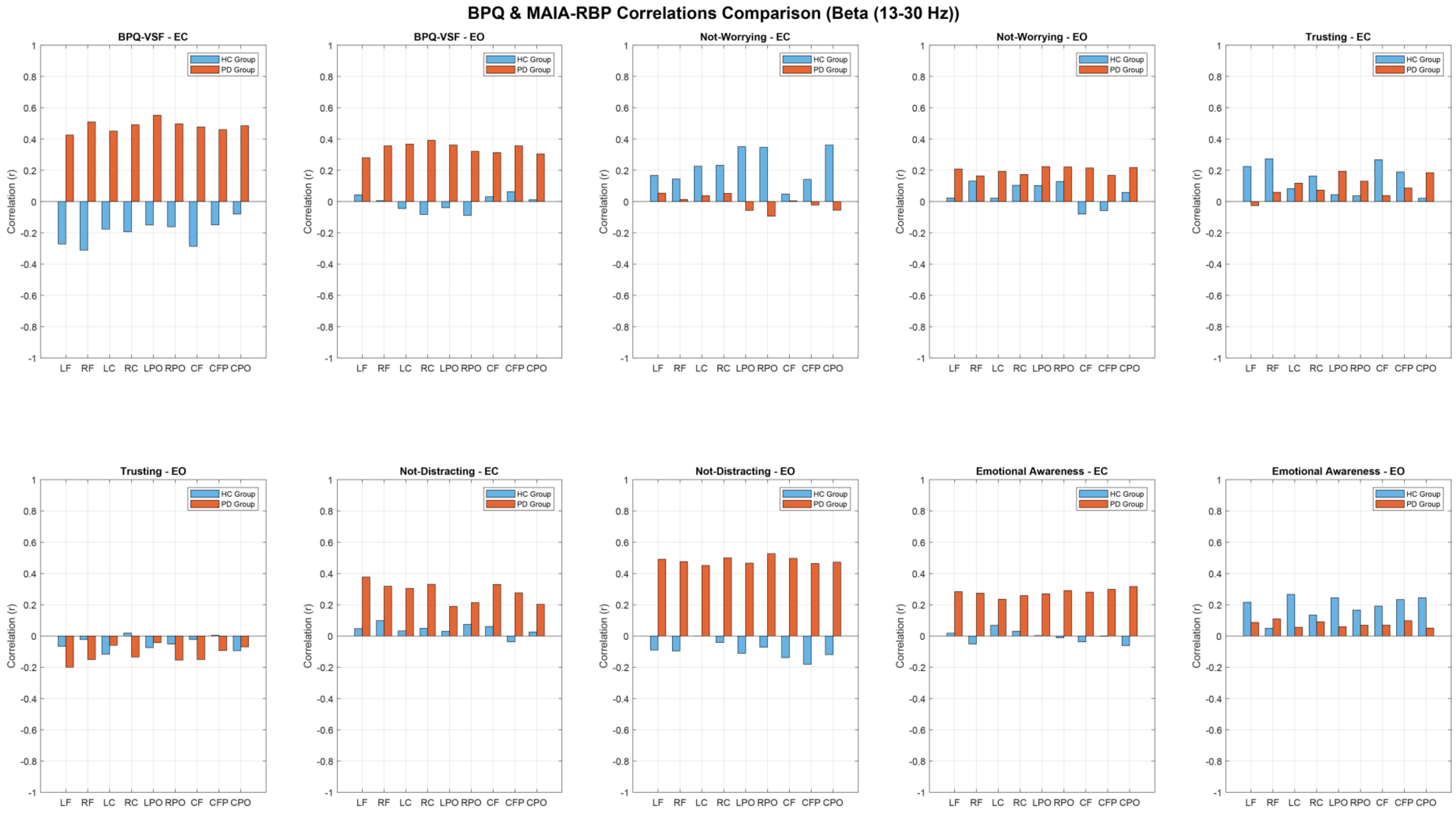
BPQ & MAIA-RBP Correlations Comparison (Beta (13-30 Hz))
BPQ-VSF - EC
BPQ-VSF - EO
Not-Worrying - EC
Not-Worrying - EO
Trusting - EC
Trusting - EO
Not-Distracting - EC
Not-Distracting - EO
Emotional Awareness - EC
Emotional Awareness - EO
HC Group
PD Group
Correlation (r)
LF RF LC RC LPO RPO CF CFP CPO

(D)

Supplementary Figure S3. Complete RBP correlation bar plots for the primary self-report constructs and frequency bands. (A) Delta-band results; (B) theta-band results; (C) alpha-band results; (D) beta-band results. Each panel shows Pearson correlation coefficients between self-reported interoceptive measures and relative band power across nine scalp ROIs for HC and PD under eyes-closed (EC) and eyes-open (EO) conditions. The displayed self-report constructs include BPQ-VSF and the MAIA dimensions selected for primary interpretation: Not-Worrying, Trusting, Not-Distracting, and Emotional Awareness. Bars indicate the direction and magnitude of Pearson correlations. Full statistics, including r values, 95% confidence intervals, p values, FDR-adjusted q values, Bayes factors, age-adjusted partial correlations, and group-interaction results, are provided in Supplementary Tables S6 and S7.
